# Modeling Host Genetic Regulation of Influenza Pathogenesis in the Collaborative Cross

**DOI:** 10.1371/journal.ppat.1003196

**Published:** 2013-02-28

**Authors:** Martin T. Ferris, David L. Aylor, Daniel Bottomly, Alan C. Whitmore, Lauri D. Aicher, Timothy A. Bell, Birgit Bradel-Tretheway, Janine T. Bryan, Ryan J. Buus, Lisa E. Gralinski, Bart L. Haagmans, Leonard McMillan, Darla R. Miller, Elizabeth Rosenzweig, William Valdar, Jeremy Wang, Gary A. Churchill, David W. Threadgill, Shannon K. McWeeney, Michael G. Katze, Fernando Pardo-Manuel de Villena, Ralph S. Baric, Mark T. Heise

**Affiliations:** 1 Carolina Vaccine Institute, University of North Carolina-Chapel Hill, Chapel Hill, North Carolina, United States of America; 2 Department of Genetics, University of North Carolina-Chapel Hill, Chapel Hill, North Carolina, United States of America; 3 Pacific Northwest Regional Center of Excellence for Biodefense and Emerging Infectious Diseases Research, Portland, Oregon, United States of America; 4 Oregon Clinical and Translational Research Institute, Oregon Health & Science University, Portland, Oregon, United States of America; 5 Department of Microbiology, School of Medicine, University of Washington, Seattle, Washington, United States of America; 6 Department of Epidemiology, University of North Carolina-Chapel Hill, Chapel Hill, North Carolina, United States of America; 7 Erasmus Medical Center, Rotterdam, the Netherlands; 8 Department of Computer Science, University of North Carolina–Chapel Hill, Chapel Hill, North Carolina, United States of America; 9 The Jackson Laboratory, Bar Harbor, Maine, United States of America; 10 Department of Genetics, North Carolina State University, Raleigh, North Carolina, United States of America; 11 Lineberger Comprehensive Cancer Center, University of North Carolina-Chapel Hill, Chapel Hill, North Carolina, United States of America; Fox Chase Cancer Center, United States of America

## Abstract

Genetic variation contributes to host responses and outcomes following infection by influenza A virus or other viral infections. Yet narrow windows of disease symptoms and confounding environmental factors have made it difficult to identify polymorphic genes that contribute to differential disease outcomes in human populations. Therefore, to control for these confounding environmental variables in a system that models the levels of genetic diversity found in outbred populations such as humans, we used incipient lines of the highly genetically diverse Collaborative Cross (CC) recombinant inbred (RI) panel (the pre-CC population) to study how genetic variation impacts influenza associated disease across a genetically diverse population. A wide range of variation in influenza disease related phenotypes including virus replication, virus-induced inflammation, and weight loss was observed. Many of the disease associated phenotypes were correlated, with viral replication and virus-induced inflammation being predictors of virus-induced weight loss. Despite these correlations, pre-CC mice with unique and novel disease phenotype combinations were observed. We also identified sets of transcripts (modules) that were correlated with aspects of disease. In order to identify how host genetic polymorphisms contribute to the observed variation in disease, we conducted quantitative trait loci (QTL) mapping. We identified several QTL contributing to specific aspects of the host response including virus-induced weight loss, titer, pulmonary edema, neutrophil recruitment to the airways, and transcriptional expression. Existing whole-genome sequence data was applied to identify high priority candidate genes within QTL regions. A key host response QTL was located at the site of the known anti-influenza *Mx1* gene. We sequenced the coding regions of *Mx1* in the eight CC founder strains, and identified a novel *Mx1* allele that showed reduced ability to inhibit viral replication, while maintaining protection from weight loss.

## Author Summary

Host responses to an infectious agent are highly variable across the human population, however, it is not entirely clear how various factors such as pathogen dose, demography, environment and host genetic polymorphisms contribute to variable host responses and infectious outcomes. In this study, a new *in vivo* experimental model was used that recapitulates many of the genetic characteristics of an outbred population, such as humans. By controlling viral dose, environment and demographic variables, we were able to focus on the role that host genetic variation plays in influenza virus infection. Both the range of disease phenotypes and the combinations of sets of disease phenotypes at 4 days post infection across this population exhibited a large amount of diversity, reminiscent of the variation seen across the human population. Multiple host genome regions were identified that contributed to different aspects of the host response to influenza infection. Taken together, these results emphasize the critical role of host genetics in the response to infectious diseases. Given the breadth of host responses seen within this population, several new models for unique host responses to infection were identified.

## Introduction

Influenza A virus (IAV) (*orthomyxoviridae*) is a negative sense RNA virus which causes severe, acute respiratory disease. Worldwide influenza infections cause several million cases annually, with severe pandemics (such as the 1918 pandemic) causing high levels of morbidity and mortality [1]. Among infected individuals there is significant variation in the clinical disease caused by IAV ranging from an asymptomatic infection to severe and acute respiratory distress syndrome [2]–[8]. Population-wide disease variation applies not only to clinical disease, but also to individual immune responses mounted in response to IAV infection [9], [10], as well as long-term complicating pathologies and co-infections [2], [11]–[13]. Despite the importance of understanding the underlying mechanisms of IAV-associated disease, the sources of the observed disease variation are unclear.

Like many viruses, IAV engages in a large number of complex interactions with various host proteins [14], [15]. It is less clear how polymorphisms in these and other host genes/proteins cause variation in the disease process following infection with IAV. A study of survival data from the 1918 IAV pandemic showed that host genetic variation [16] contributes to IAV disease variation. However, in contrast with other pathogens [17]–[21], human polymorphisms have not yet been identified that contribute to variable responses to IAV infection, although there have been some suggestions of polymorphisms in HLA contributing to IAV recovery [22], [23]. As IAV disease severity is likely due to a combination of viral, host, demographic and environmental factors [7], [13], [24]–[26], this complexity has interfered with reductionist approaches to evaluating the role that host genetic variation plays in regulating different IAV-associated disease outcomes across the population.

Mouse models of IAV infection have provided novel insights into the role of host genetics on IAV disease outcomes. This approach led to the discovery of the naturally polymorphic, interferon inducible *Mx1* gene, which inhibits IAV replication and limits disease [27]. Subsequently, most studies of host genetic contributions have used naturally defective *Mx1* mouse strains, such as C57BL/6J to study the effect of gene knock-outs on the host response to influenza. These studies have shown that many genes contribute to the host response [28]–[35] (reviewed in [36], [37]), and knock-outs often affect clinical disease primarily by altering the host inflammatory response [28], [29], [32], [35], [38]–[40]. Comparisons between inbred mouse strains [41]–[44] have confirmed that natural variation contributes to differential host responses. Given that most polymorphisms within the human population will be those that alter expression and/or function, rather than whole gene knock-outs, studies comparing naturally occurring polymorphisms are more relevant to human disease. Several recent studies [42], [45], [46] using classical recombinant inbred (RI) panels have identified a number of quantitative trait loci (QTL) contributing to host responses following IAV infection. However, traditional mouse genetics systems have limitations on their ability to accurately model the genetic structure and diversity of outbred populations, like humans [47], [48].

We developed a new model that captures host responses to IAV infection across a genetically diverse host population by using incipient lines from the Collaborative Cross (CC) octo-parental RI panel, known as the pre-CC population [49]–[51]. This population is highly genetically diverse (∼40 million single nucleotide polymorphisms (SNPs) evenly distributed across the genome), with up to eight functionally variant alleles at any given locus [52]. The pre-CC population exhibited a broad range of phenotypic outcomes, including unique combinations of disease phenotypes following infection, and we identified three novel QTL associated with multiple aspects of influenza induced disease. Furthermore, we identified a novel *Mx1* allele in the CAST/EiJ mouse strain and sequenced the associated haplotype. By integrating QTL mapping with whole genome sequence information, we significantly reduced the number of candidate genes within each QTL. Our findings provide a clarification of the importance of genetic variation in the host's response to IAV infection, and a foundation of support for the hypothesis that genetically complex mouse models such as the CC will provide a robust platform for studying the role of host genetic variation in regulating the host response to infection.

## Results

### Diverse IAV-associated phenotypic and transcriptome variation

We used 155 pre-CC mice, each from an independent, incipient CC line, as well as sets of mice (*n* = 5–11) from each of the eight CC founder strains. Mice were infected with a dose of the mouse adapted A/PR/8/34 (PR8) IAV that was known to cause severe disease in several of the CC founder strains (C57BL/6J, 129s1/SvImJ, A/J), and we assessed IAV-induced weight loss (measured as a percentage of starting weight) and clinical disease daily through four days post infection (D4), at which point the mice were euthanized and lung tissue assessed for viral replication, virus-induced inflammation and pathology, and (pre-CC mice only) transcriptional profiles by microarray analysis within the lungs (Table 1, Table S1, Dataset S1). This D4 timepoint was chosen to allow severe pathology to develop in susceptible lines, while minimizing the animals lost in this study due to humane euthanasia conditions. We examined the weight changes and clinical scores animals experienced through the course of this experiment, and found that weight loss and clinical scores of animals were highest at D4. We therefore limited our analysis of weight and clinical scores to this timepoint. Importantly, in analyzing the phenotypes of the pre-CC mice, we found no evidence for effects of age, generation of inbreeding, block effects or starting weights on gathered phenotypes, and therefore did not include these variables in our analysis.

**Table 1:** Phenotypes measured in the pre-CC and founder strains.

	Phenotype	Pre-CC	Founders
Clinical Disease	D4 weight	X	X
	D4 clinical score	X	X
	Hemorrhage	X	
	Gross Edema	X	
Viral Replication	Log Titer	X	X
	IHC score	X	
Virus-induced inflammation	Airway inflammation	X	X
	Airway neutrophils	X	
	Airway monocytes	X	
	Vascular inflammation	X	X
	Vascular neutrophils	X	
	Vascular monocytes	X	
	Alveolar inflammation	X	X
Pathology	Airway damage	X	X
	Alveolar damage	X	X
	Pulmonary edema	X	
	Fibrin deposition	X	
Transcription	D4 lung expression	X	

**Figure 1:**
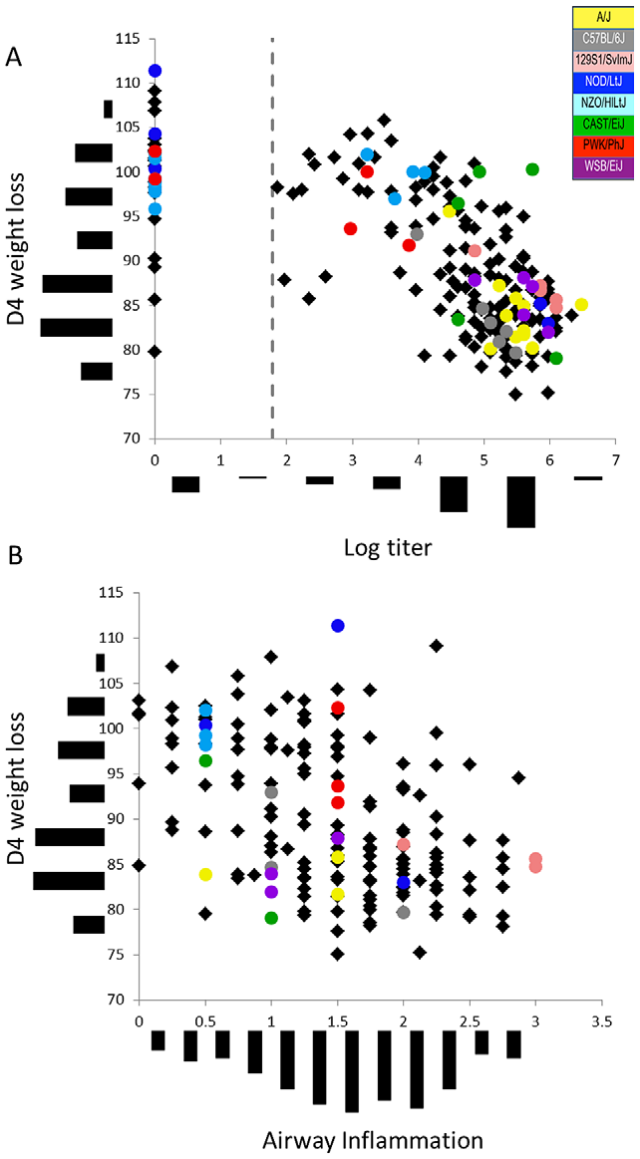
Diverse disease-associated phenotypes across the pre-CC population. Pre-CC mice showed a wide range of variation in phenotypes including D4 weight (Y-axis histogram, A and B), Log titer (X-axis histogram, A) and Airway Inflammation (X-axis histogram, B). In addition, strong correlations existed between D4 weight and both (A) Log titer and (B) Airway Inflammation across the pre-CC population (black diamonds). Despite these correlations, individual pre-CC mice showed unique combinations of disease phenotypes (e.g. low Log titer and severe D4 weight loss) not present in the founder strains of the CC (colored circles: A/J (n = 11) = yellow, C57BL/6J (n = 6) = grey, 129S1/SvImJ (n = 5) = pink, NOD/ShiLtJ (n = 5) = dk. Blue, NZO/HILtJ (n = 12) = lt. blue, CAST/EiJ (n = 5) = green, PWK/PhJ (n = 5) = red, WSB/EiJ (n = 5) = purple).

**Figure 2:**
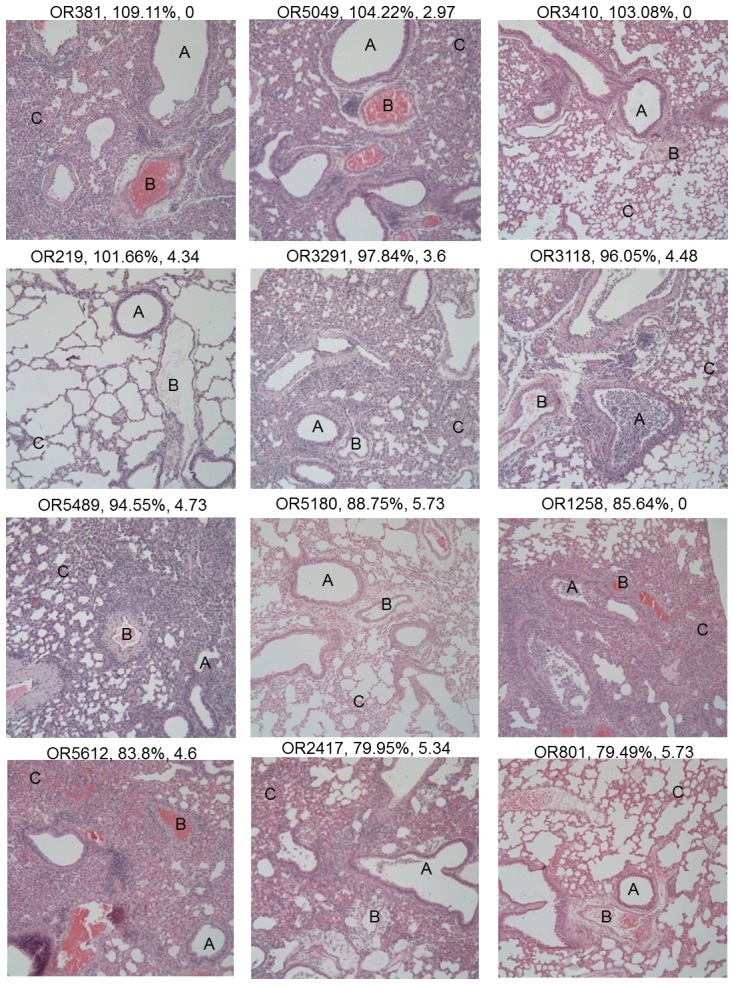
Diverse disease pathologies present across the pre-CC population. Histopathological examination of lung sections following IAV infection showed a diverse range of phenotypes. Each image is a single 100× magnification image of the lung section of a single pre-CC mouse (strain ID, D4 weight, and log titer (BDL = below detectable limit) are listed over each image). Disease phenotypes were scored for aspects of the damage to, and inflammatory cell infiltration around the airways (A), inflammatory cell infiltration around the vasculature (B), and damage and inflammatory cell infiltration in the alveolar spaces (C). Note that the image of OR219 shows a relatively healthy looking lung, and is useful as a baseline image.

The infected founder strains varied significantly for all measured phenotypes, including D4 weight, log titer, virus induced inflammation and pathology, except for variation in alveolar debris (p-values ranging from 0.15 to 1.37×10^−9^, Figure 1, Table S1). Founder strains could be grouped into susceptible (high viral titer, inflammation and weight loss) or resistant (low viral titer, little inflammation and weight loss) groups (Figure 1, Figure S1). As with the founders, many aspects of IAV associated disease were correlated with each other across the pre-CC population (correlation coefficients ranging from −0.78 to 0.78, Figure 1, Table S2), with the exception of alveolar immune cell infiltration as well as gross edema and hemorrhage at time of harvest, which were not strongly correlated with the rest of the host response to infection. Pre-CC mice often showed unique combinations of disease-associated phenotypes (e.g. high levels of viral replication but low inflammation and weight loss, no replication but significant weight loss, Figures 1 and 2). Therefore, though the pre-CC population recapitulated the range of variation within any given phenotype (Table S1), we observed new phenotypic combinations not seen in the parental lines.

The unique combinations of disease-associated phenotypes across the pre-CC population led us to investigate the relationships between viral replication and immune cell infiltration on weight loss, a long standing question within the IAV field. Since the large number of pre-CC mice we had in this study lacked the genetic structure of the founder strains, this population was uniquely positioned to evaluate the relationships between these disease parameters. Both log titer and airway inflammation (the cellular infiltrate most clearly related to infection status) were significant predictors (p-values<2.2×10^−16^ and 1.56×10^−9^, respectively) of D4 weight (Figure 1, Table S3). However, log titer and airway inflammation together were significantly better predictors of D4 weight than either variable alone (based on both partial F tests and Akaike Information Criterion (AIC), Table S3).

**Figure 3:**
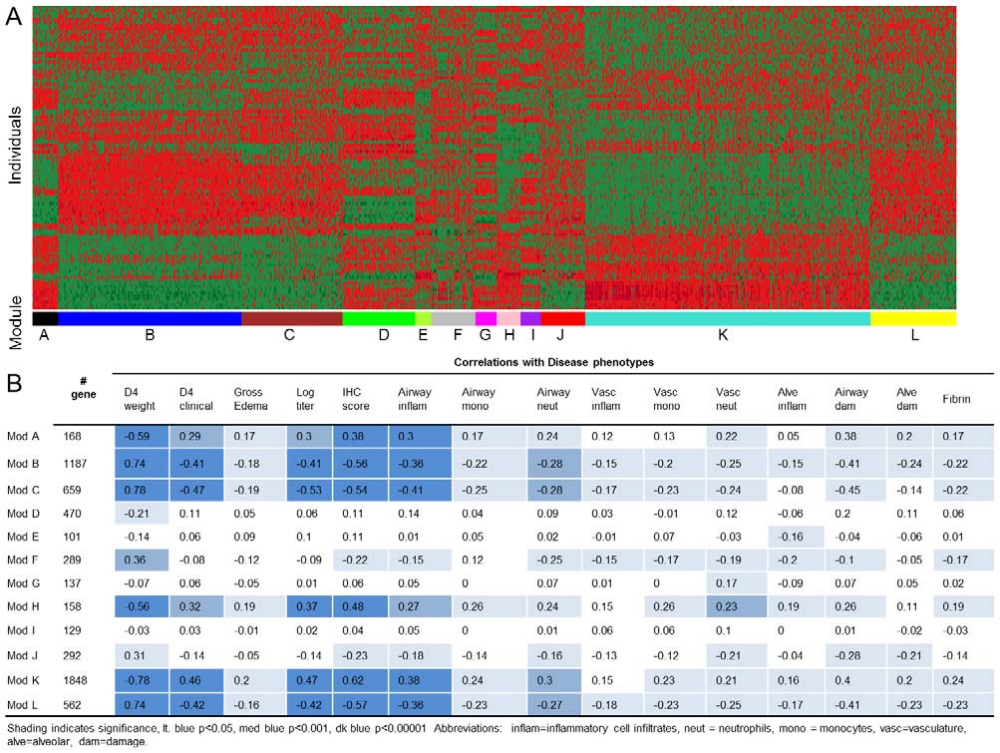
Transcriptional Modules across the pre-CC population. (A) highly variable transcripts from across the pre-CC population were grouped into modules (sets of transcripts that are connected and similarly expressed (shown here by heat map intensity) within individuals). Modules are given colored bars below them for visual clarity. (B) Modules were correlated with different disease phenotypes.

In addition to measuring disease associated phenotypes, we also assessed host transcript levels within the lungs at four days post infection. Of the 155 pre-CC mice used in this study, 99 had RNA of sufficient quality to use for RNA microarray analysis (see GEO, accession GSE30506 for full microarray dataset). A total of 11,700 genes passed quality control processing, and did not have a SNP across the eight founder lines which could impact their intensity on the array. Out of these 11,700, we identified the 6000 most variable and interconnected genes across this population and used weighted gene co-expression network analysis (WCGNA) to cluster these transcripts into twelve modules, labeled A–L (Figure 3, Table S4). Seven modules (B, D, F–I, K) were enriched for specific gene ontology (GO) terms (Table S5), including cellular signaling (module G), cell growth and biosynthesis (module D) and immune responses (module K). There was little to no overlap between the enriched categories across modules. We used the eigengene, an idealized representation of module transcription levels for each individual mouse, to correlate module expression levels with disease phenotypes as eigengene expression has been used previously to simply describe the sets of transcripts within a module [53]. We found that eigengene values for eight of the twelve modules (modules A–C, F, H, and J–L) were correlated with multiple disease-related phenotypes. Modules E and G correlated with aspects of Virus-induced inflammation and module D correlated with D4 weight (Figure 3). These results suggest that in this genetically diverse population severity of influenza infection is associated with wide-scale variation in a large number of biological processes within the lung.

### Genetic mapping reveals host genome regions controlling IAV-induced host responses

Given the variation in disease, virologic, inflammatory, pathologic, and transcriptional phenotypes observed in the pre-CC animals, we conducted QTL mapping (as in [49]) to identify host genome regions contributing to variation in IAV-induced phenotypes (Figures S2, S3, S4, Table 2). Previous studies [27] have identified a large effect IAV resistance gene, *Mx1*, on chromosome 16, and our *a priori* expectation (see below) was that we would identify a QTL over *Mx1*, validating our mapping approaches within the CC. Consistent with this, we identified a highly significant QTL on chromosome 16, *HrI1* (*H*ost *r*esponse to *I*nfluenza) that contributed to a number of these disease-associated phenotypes (D4 weight, D4 clinical, log titer, IHC score, airway inflammation and airway damage). *HrI1* explained 41.67% of the variation in weight loss (i.e. the adjusted R-squared for a model with all 8 strain effects), and similar amounts of variation in other phenotypes, and was located in a 0.71 Mb region (1.5 LOD interval: 97500418-98213493) annotated as containing 10 genes and one non-coding RNA, including the known anti-influenza gene *Mx1*. We also conducted QTL mapping on the eigengenes of the expression modules to identify mQTL (module QTL). Three modules (B, C, and K) had QTL that overlapped with *HrI1* (Table 2). These modules included ones enriched for a number of cell-adhesion and morphogenesis/development transcripts (module B) and immune system response phenotypes (module K), while module C was not enriched for any specific functional categories.

**Table 2:** QTL identified in the pre-CC population.

QTL	Phenotypes	Location (Megabases)
*HrI1*	D4 weight	16:97.5–98.2
	Log titer	
	IHC score	
	D4 clinical	
	Airway inflammation	
	Airway damage	
	Module B	
	Module C	
	Module K	
*HrI2*	D4 weight	7:89.1–96.7
*HrI3*	Pulmonary edema	1:21.7–29.0
*HrI4*	Airway neutrophils	15:77.4–86.6

**Figure 4:**
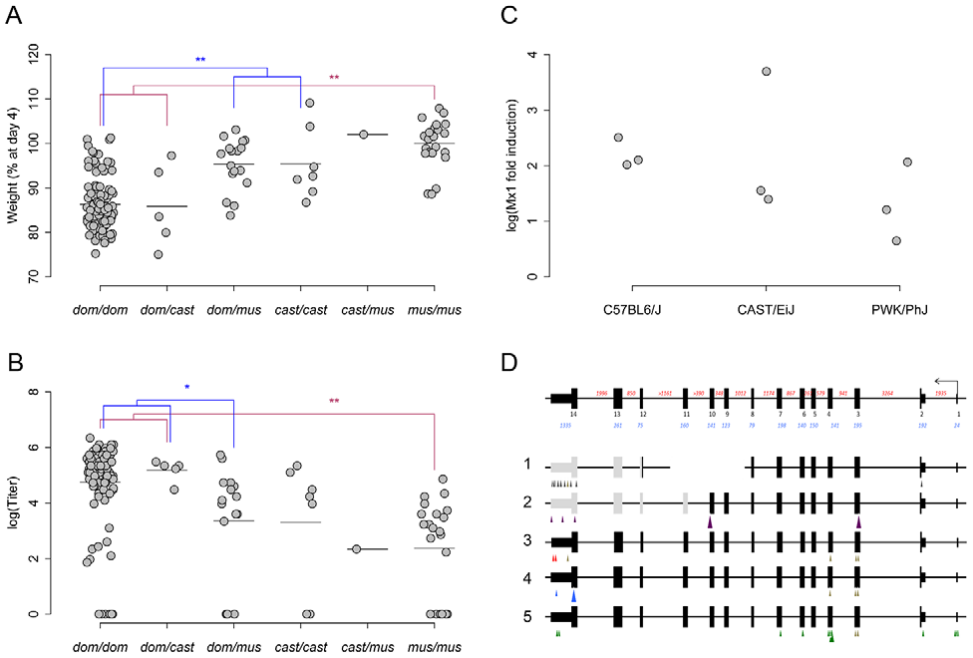
A novel *Mx1* allele differentially impacts host response to influenza. The founder strain alleles at *Mx1* were grouped based on their phenotypic effects into three functionally distinct classes corresponding to *domesticus* (*dom*: A/J, C57BL6/J, 129s1/SvImJ, NOD/HiLtJ and WSB/EiJ), *castaneus* (*cast*: CAST/EiJ) and *musculus* (*mus*: PWK/PhJ and NZO/ShILtJ). Points shown are individual pre-CC animals with these haplotypes, mean bars are shown for each class. These functionally distinct classes were separable based upon differences in (A) D4 weight and (B) Log titer, with the heterozygous classes showing intermediate phenotypes. Across the pre-CC population, homozygous *dom* animals had severe weight loss and high titers. Homozygous *mus* animals showed little weight loss and low titers. Homozygous *cast* animals showed little weight loss, but had intermediate viral titers. Brackets between groups represent significant differences (* = p<0.05, ** = p<0.003) based on Tukey's HSD. We found no difference by qPCR (C) in expression of *Mx1* at 2 days post-infection following influenza infection in a strain from each of these three functional classes. By sequencing *Mx1* (D), we were able to identify five haplotypes across the eight founder strains (Haplotype 1 = A/J, C57BL/6J, 129S1/SvImJ, NOD/HiLtJ; Haplotype 2 = WSB/EiJ; Haplotype 3 = PWK/PhJ; Haplotype 4 = NZO/HiLtJ; Haplotype 5 = CAST/EiJ). Arrows indicate locations of polymorphisms, with small arrows indicating non-coding changes, and large arrows indicating coding changes. Colors correspond to the founder strains having those polymorphisms (brown = multiple strains possess mutation). Grey exons indicate those not transcribed due to either deletion and frameshift, or insertion and early stop codon.

We grouped the eight founder alleles at *HrI1* by their estimated effects on each phenotype [49], [54], [55]. Alleles from five strains (A/J, C57BL/6J, 129S1/SvImJ, NOD/ShiLtJ and WSB/EiJ) affected the host response similarly and were associated with decreased influenza resistance (i.e. higher titers, higher weight loss, more pathology), increased module K and decreased expression of modules B and C. A/J, C57BL/6J and WSB/EiJ had previously been identified [27], [56] as having nonfunctional *Mx1* alleles. In contrast, the NZO/HILtJ and PWK/PhJ alleles within the pre-CC population shared similar effects and increased influenza resistance. Previously, CAST/EiJ was characterized as having a full length *Mx1* allele, based on analysis of portions of the *Mx1* locus [56]. Despite the presence of a full length transcript, the effect of the CAST/EiJ allele across the pre-CC population was intermediate in conferring resistance in our QTL models. Pre-CC mice with the CAST/EiJ allele showed low-to-intermediate weight loss. In contrast, these animals had viral titers that were intermediate between animals with nonfunctional *Mx1* alleles and those with a NZO/HILtJ or PWK/PhJ allele (Figure 4). These three functional groups held true when considering animals from the eight founder strains. Importantly, animals from the CAST/EiJ strain showed intermediate weight loss, but had viral titers no different than founder strains with nonfunctional *Mx1* alleles (Figure 1). We conclude that three *Mx1* alleles segregate in the pre-CC population, with the CAST/EiJ allele being functionally distinct from the classical protective *Mx1* allele, where this allele confers limited protection from viral replication, but does protect from virus-induced weight loss. We found no significant differences in *Mx1* mRNA gene expression in the lung at two days post-infection using one strain from each allele group (C57BL/6J, CAST/EiJ and PWK/PhJ, Figure 4). C57BL/6J had the highest mean level of up-regulation, with CAST/EiJ intermediate and PWK/PhJ having the lowest level of expression. This suggests that the differences between the CAST/EiJ and PWK/PhJ alleles are due to coding changes within the gene and not variation in gene expression.

The sequence variation at the *Mx1* locus in mouse is poorly understood despite its well-known role in influenza susceptibility. This is due in part to the presence of a deletion in the C57BL/6J strain (the mouse reference genome, see Materials and Methods) and the subsequent effect on the annotation of several of *Mx1* exons in the mouse assembly (mm9) and in the Sanger Institute's Mouse Genomes sequencing project [57]. Therefore, we identified the genetic variants in all *Mx1* exons in each of the eight founder strains by sequencing the *Mx1* exons from each strain (see Methods). We found five distinct haplotypes (Figure 4, Table S6). The most common haplotype in the CC contains a large (>2 kb) deletion that spans three coding exons (9, 10, and 11) that are highly conserved among placental mammals. As previously described [27], this deletion leads to a frame shift and early stop codon in exon 12. This haplotype results in the presence of the same non-functional *Mx1* gene in A/J, C57BL/6J, 129S1/SvImJ and NOD/ShiLtJ. We confirmed that WSB/EiJ also has a non-functional allele due to a nonsense mutation in exon 10 [56]. The other three strains have full length ORFs and each has a distinct protein sequence due to the different combinations of alleles at two non-synonymous SNPs. However, the genetic variants identified in our analysis and the regional assignment of sub-specific origin [47], [58] demonstrate that the CAST/EiJ strain has a divergent haplotype of *Mus musculus castaneus* origin while PWK/PhJ and NZO/HILtJ both have haplotypes of *M. m. musculus* origin. The three functional haplotypes are characterized largely by synonymous variation and variation in untranslated regions of the gene. There was a single amino acid substitution identified in NZO/HILtJ relative to PWK/PhJ and CAST/EiJ (Gly616Arg), and a single amino acid substitution identified in CAST/EiJ relative to NZO/HILtJ and PWK/PhJ (Gly83Arg). Although we cannot preclude transcriptional differences at different time points during infection from having a role in the functional differences between the three *Mx1* alleles, the nonsynonymous substitution that is unique to the CAST/EiJ haplotypes is a strong candidate to explain the intermediate phenotype of the CAST/EiJ *Mx1* allele. These results demonstrate our ability to identify: 1) a known IAV resistance locus with only one mouse per line, 2) the multiple phenotypes regulated by this locus, and 3) previously unidentified allelic variants due to the multiple alleles segregating within the pre-CC population.

We returned to our transcriptional data in an attempt to better understand how the CAST/EiJ *Mx1* allele could contribute to protection from weight loss while showing high titers and severe inflammatory responses. Of the 11,700 transcripts that passed QA/QC and were not SNP impacted, we determined that 2156 transcripts (18.4%) had their expression levels significantly impacted by genotype at the most significant *Mx1* marker (i.e. these transcripts had an expression, or eQTL at *Mx1*, Table S7), confirming the large role that *Mx1* has on regulating the response to IAV infection. We grouped these transcripts based on their allele effects, specifically looking for those transcripts where (a) CAST/EiJ *Mx1* alleles grouped with the resistant PWK/PhJ or NZO/HILtJ *Mx1* alleles, or (b) where CAST/EiJ allele grouped with susceptible A/J, C57BL/6J, 129S1/SvImJ, NOD/ShiLtJ and WSB/EiJ *Mx1* alleles. A total of 307 transcripts with an eQTL at *Mx1* (14.2%) had allele effects consistent with CAST/EiJ grouping with the resistant PWK/PhJ and NZO/HILtJ alleles, while 1207 transcripts with an eQTL at *Mx1* (55.9%) had allele effects consistent with CAST/EiJ grouping with the susceptible A/J, C57BL/6J, 129S1/SvImJ, NOD/ShiLtJ and WSB/EiJ alleles.

Those transcripts where CAST/EiJ grouped with the susceptible alleles showed significant enrichments for a large number of GO terms (Table S8), including biosynthesis and biogenesis processes (upregulated in the lines with a PWK/PhJ or NZO/HILtJ allele) and a highly diverse array of inflammatory, apoptotic, chemotactic, cell growth and hematologic-based terms (upregulated in the lines with A/J, C57BL/6J, 129S1/SvImJ, NOD/ShiLtJ, CAST/EiJ and WSB/EiJ alleles). In contrast, those transcripts where CAST/EiJ was grouped with the resistant PWK/PhJ and NZO/HILtJ alleles showed a much more limited enrichment, with mainly cytokine and T-cell processes (downregulated in lines with CAST/EiJ, PWK/PhJ and NZO/HILtJ alleles) being enriched.

Despite the large effect of *Mx1* on influenza response, there was large phenotypic variation within both the functional and non-functional *Mx1* allele classes. This suggested the presence of modifier alleles segregating in the pre-CC population. To find these modifiers, we conducted additional genome scans after accounting for genotype at the most significant *HrI1* marker (see methods), thereby controlling for *Mx1* allele. This model accounted for the large effect of *HrI1* and resulted in a single significant QTL, *HrI2*, on chromosome 7 (1.5 LOD interval: 89130587-96764352), that explained 9.7% of the variation in D4 weight (Figure S2). This region is annotated as containing 69 genes and 10 non-coding RNAs. Analysis of the allelic effects at *HrI2* suggests that animals with an A/J allele showed less weight loss than other animals, and animals with a 129S1/SvImJ allele showed more weight loss than other animals.

### Unique alleles contribute to disease phenotypes in a susceptible sub-population

To eliminate epistatic effects of the protective *Mx1* genotype, we analyzed those *Mx1*-/- individuals in our pre-CC population, where this group consisted of 99 mice defined as having two *Mx1* alleles coming from any of the A/J, C57BL6/J, 129S1/SvImJ, NOD/ShiLtJ or WSB/EiJ strains. Although this susceptible subpopulation still showed a wide range of phenotypes (Table S9), it was skewed towards increased disease-associated phenotypes. The correlations between weight loss, viral replication, pathology and aspects of the immune cell infiltrate were weaker than those seen across the whole population (Table S10). Specifically, while aspects of pathology and immune cell infiltrate remained correlated with each other, we observed reduced correlations between titer and pathology, titer and inflammation, and clinical disease and pathology. We also reexamined the relationship between titer, airway inflammation and weight loss, to determine if our earlier observation, which linked both titer and airway inflammation as significant predictors of weight loss was independent of *Mx1* status. Despite the reduced strength of relationships across the population, both titer and airway inflammation were still significant predictors of weight loss. Again, knowledge of both titer and airway inflammation was a better predictor of weight loss than either variable alone (based on both partial F tests and AIC, Table S3).

RNA of high quality was recovered from 60 mice within this *Mx1*-/- population, and we used WCGNA to cluster the 6,000 most variable transcripts across this population (4,933 of these 6,000 transcripts were also identified in the whole population analysis). Even in the absence of a large effect resistance gene, *Mx1*, we were able to group transcripts into functionally relevant co-expression modules. In total, this analysis identified eleven modules labeled M-W (Table S11). Again, modules were enriched for a wide range of functional terms, and showed little overlap between categories (Table S12). Eight modules (M-O, Q, T-W) were significantly correlated with clinical disease and/or viral replication, being enriched for T-cell processes (module M), inflammatory responses (module N), and signaling processes (module O). Module Q (enriched for cell cycle processes) was exclusively associated with some virus-induced inflammation, and two modules had no clear relationships with any phenotypes (enriched for sensory and neurological processes (module U) as well as metabolic and biosynthesis (module V), Table S13). Absence of an mQTL overlapping the *Mx1* region (see below) indicates that, as expected, the effect of the *Mx1* locus on the coexpression network has been ameliorated in this population. This indicates, along with the continued, albeit, weaker associations between the modules and phenotypes that performing a WGCNA analysis conditioning on the *Mx1* allele group provides a way to highlight additional diseases-associated genetic regulation of transcript expression.

When we conducted QTL mapping in the *Mx1*-/- subpopulation, we identified a significant QTL, *HrI3*, which explained 29.73% of the variation in Pulmonary Edema on chromosome 1 (7.31 Mb, 21767867-29085401) annotated as containing 24 genes and 11 non-coding RNA (Table 2, Figure S3). Additionally, we identified a suggestive QTL, *HrI4*, which explained 22.77% of the variation in airway neutrophils on chromosome 15 (77427235-86625488), a 9.19 Mb region annotated as containing 206 genes and 35 non-coding RNAs (Table 2, Figure S4). In contrast to our results with the whole pre-CC population, we were not able to identify any mQTL contributing to variation in module expression within this *Mx1*-/- subpopulation.

### Genetic variation underneath *HrI3* contributes to pulmonary edema

**Figure 5:**
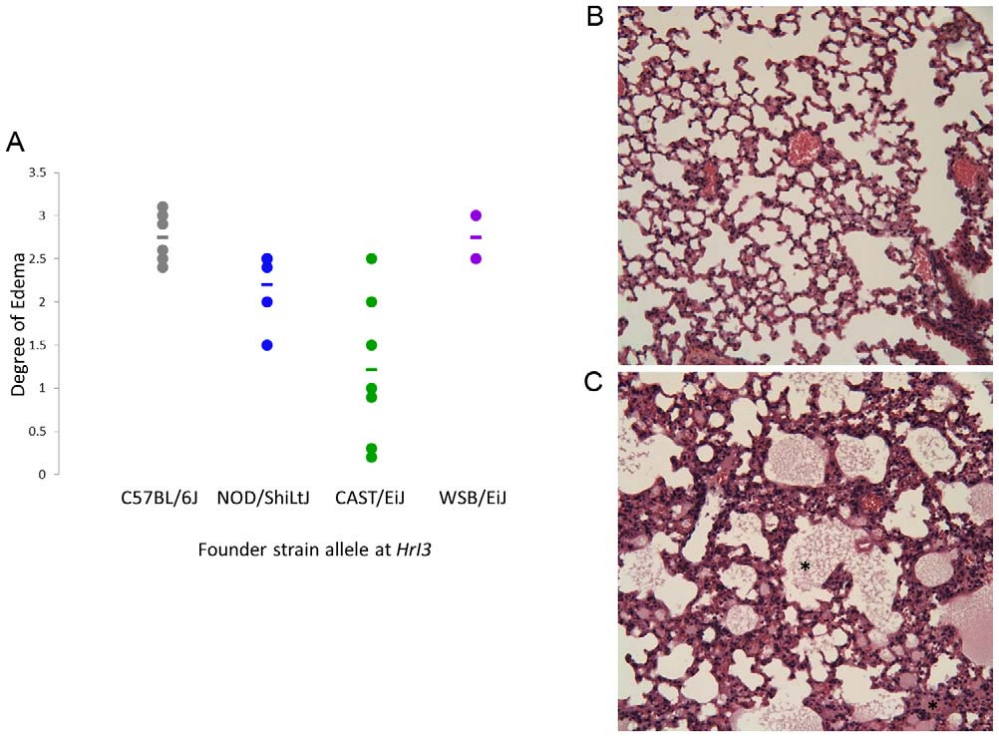
Genetic variation at *HrI3* contributes to variation in pulmonary edema in an independent set of Collaborative Cross lines. Following the identification of *HrI3*, we infected animals from fully inbred Collaborative Cross lines, where each line was homozygous for a single founder allele at *HrI3*. (A) We found a significant effect of genotype at *HrI3* on the extent and severity of pulmonary edema at four days post infection. Mild (B) and Severe (C) pulmonary edema can be seen at 200× magnification in animals from this experiment. Pulmonary edema was scored on the basis of evidence of transudates accumulating in the alveolar spaces (denoted by star marks in panel C).

In order to confirm the role of *HrI3* in contributing to control of pulmonary edema, we challenged a new set of female animals from a small set of completely inbred CC lines with IAV. These animals were homozygous for various founder alleles across the entire candidate region for *HrI3* and founder strain alleles were each represented by two CC lines (e.g. two lines that vary across the rest of their genome both share the WSB/EiJ allele at *HrI3*). We examined the severity of pulmonary edema in these animals at four days post infection. Founder strain alleles at *HrI3* significantly affected pulmonary edema (F_3,16_ = 8.48, p = 0.0013, Figure 5), validating the role of this genome region in the host response to IAV.

### Identification of candidate genes from whole-genome sequences

Having identified three novel QTL our next objective was to narrow down QTL regions to specific candidate genes/features. We used the estimated allele effects along with the whole genome sequences of the founder strains [57] to narrow the list of candidate genes within each interval (see methods).

When a particular allele underlies a QTL, it is due to a causal genome feature (e.g. SNP, insertion/deletion) corresponding to that allele, contrasted with the other alleles in the cross. In the simplest case of one allele contrasted with the other seven this means that a private genome feature in the single strain is causative for the QTL. Under more complex scenarios (e.g. two strains contrasted with six), because CC mice share common ancestry due to their natural history and the unique history of laboratory mice [47], [58], we assume that causal alleles are often shared across mouse strains. That is, if two strains are segregating from the other six, it is likely due to a common feature these two strains privately share. We used the allele effects plots (Figures S2, S3, S4) to group founder strains underneath QTL peaks into two groups based on the largest difference between groups (Note that for completely inbred lines, phenotype-by-genotype plots would provide similar information. For the incompletely inbred pre-CC mice, with up to 36 allele combinations at each locus, PxG plots are difficult to interpret). For each group, we identified the regions in which all strains were identical or nearly identical (≥98%). Then we excluded regions that were not unique to the allele group (e.g. where two causative alleles had different SNP patterns). Using this approach, we narrowed the candidate regions for *HrI3* (Pulmonary Edema: NZO/HILtJ and WSB/EiJ alleles reducing edema), from 7.31 Mb to 1.01 Mb, containing 10/24 genes and 1/10 annotated non-coding RNAs (Table 3). *HrI*4 (Airways Neutrophils: C57BL6/J, NZO/HILtJ and PWK/PhJ increasing infiltration) was similarly reduced from 9.19 Mb to 91 kb, including 12/206 genes and 2/35 non-coding RNAs (Table 3). *HrI*2 represented a case where a single founder allele associated with either increased resistance (A/J) or susceptibility (129S1/SvImJ) contrasted with the other six strains showing an intermediate phenotype. We were therefore looking for individual SNPs (and not regions of difference) that differentiated A/J or 129S1/SvImJ from the other strains. We identified 144 private A/J SNPs or small in/dels, and 611 private 129S1/SvImJ SNPs or small in/dels (out of a total of 106,684 SNPs or small in/dels in the region). These SNPs occur in or near 28 genes (7 genes unique to A/J, 13 unique to 129S1/SvImJ, and 8 overlapping between the two, Table 3).

**Table 3:** Candidate genes within QTL regions.

HrI2		HrI3	HrI4
9930013L23Rik (A)	Vmn2r73 (A)	4931308C20Rik	Atxn10
AC139576.1 (A)	1700026D08Rik (C)	Bai3	AW121686
AC156557.1	AC099601.2 (C)	Col19a1	Cacng2
AdamTsl3 (A)	AC111022.1 (C)	Fam135a	Card10
Ctsc (A)	AC161439.1 (C)	Gm5697	Cbx7
Folh1	Arnt2 (C)	Gm9884	Celsr1
Grm5	Eftud1 (C)	Gm11161	Enthd1
Il16	Fam108c (C)	Kcnq5	Grap2
Mesdc2	Fam154b (C)	Rims1	Lgals2
Nox4	Mex3b (C)	Smap1	Mirlet7c-2
Sh3gl3	Olfr301 (C)	SNORA17	Pdgfb
Tmc3	Tmem135 (C)		Sstr3
Tyr (A)	Vmn2r72.ps (C)		Syngr1
Vmn2r66 (A)	zfand6 (C)		

For *HrI2*, (A) refers to genes with a private A/J SNP, (C) refers to genes with a private 129S1/SvImJ SNP. Unmarked genes had both A/J and 129S1/SvImJ SNPs.

In all three of these cases, the high priority candidate genes we identified covered a range of biological functions, including a large number with no annotated functions. While no obvious candidates jump out with *HrI3*, *HrI4* includes *Grap2*, involved in leucocyte specific signaling [59]. Similarly, *HrI2* includes the chemoattractant/T-cell modulator *Il16* [60] as well as *Nox4*, which is potentially involved in production of reactive oxygen species and interacts with the TLR4 pathway [61].

## Discussion

The host response to infection represents a complex set of interacting phenotypes, where variation in these phenotypes is likely influenced by interactions between multiple polymorphic genes as well as other factors (specific virus-host interactions, environment, exposure, age). While reverse genetics approaches have afforded insight into the role of viral genes in infection [62]–[64], well defined models do not exist for understanding how polymorphic host genes interact to regulate host response to infection. A number of mouse models including gene specific knockouts and transgenic lines [28]–[30], [32], [34], [35], [39], [65], panels of genetically distinct mouse lines [44], [66], and classical RI panels [42], [45], [46], have been used to provide key insights into the role of specific genes in pathogenesis, however, these systems do not accurately reflect the situation in outbred populations. While these systems either interrogate the role of specific genes in the context of a single genetic background (e.g. knockouts) or analyze the impact of two variant alleles (e.g. classic RI panels) on disease pathogenesis, in genetically complex populations, such as humans, disease outcomes are likely determined by interactions between multiple polymorphic genes, with multiple polymorphic alleles at these loci. Therefore, we chose to use the pre-CC population to assess how genetic polymorphisms impact the host response to influenza infection in a population of animals that more closely represents the genetic diversity found in outbred populations. Our results show that even within the constraints of this pre-CC study (i.e. one animal/incipient line, single time point), we were able to uncover underlying relationships between host responses to infection, identify new disease phenotype combinations not present within the founder strains, and identify novel QTL impacting aspects of the host response to infection, suggesting that the CC panel represents a powerful system for studying pathogen interactions within genetically complex populations.

### Host genetic control of infectious disease responses

Host genetic polymorphisms have been shown to contribute to differential disease outcomes, and evidence exists for influenza [16], [22], [23], [67] that suggests host genetic variants are important regulators of influenza pathogenesis. The identification of a QTL of major effect sitting over the anti-influenza gene *Mx1* was not surprising. *Mx1* is known to strongly inhibit influenza virus replication, limiting the resultant IAV-induced disease symptoms in mice [68]. As was to be expected, within the pre-CC population, functional *Mx1* alleles reduced viral titers, weight loss and clinical disease, inflammation and pathology. *Mx1* also acted to influence the expression levels of a large number of transcripts. While it is unlikely a direct transcriptional regulator, *Mx1*'s potent ability to inhibit IAV replication likely alters the signaling environments and host response pathways triggered in response to infection.

Within human populations, it is possible for multiple alleles to exist at any given locus. Similarly, at any locus within the pre-CC population, up to eight distinct alleles exist. In addition to increasing the probability of having functionally variant alleles segregating within the population, multiple alleles at a locus can give rise to distinct phenotypic outcomes across the population. The effects of this allelic variation can clearly be seen when considering *Mx1*. A total of 5 distinct *Mx1* haplotypes exist in the pre-CC population, and they can be grouped into three functionally distinct alleles based on their effects during influenza infection. Of particular interest is the CAST/EiJ allele, which disassociates the effects of *Mx1* on control of viral replication from its' ability to protect from a clinical disease aspects. We utilized the large number of transcripts that had an eQTL at *Mx1* to better understand potential ways in which the CAST/EiJ allele might provide clinical protection while being unable to control IAV replication. We identified a set of transcripts that were significantly upregulated in those individuals with defective *Mx1*s, but were downregulated in individuals with CAST/EiJ, PWK/PhJ and NZO/HILtJ *Mx1* allele). These transcripts included sets of inflammatory and immune related transcripts, such as *SOCS3*, *Irf1*, and *Interferon-gamma*. GTPases, such as *Mx1*, are important in a number of signaling and protein production activities [69], [70], and it is possible that the signaling activities of *Mx1* are responsible for regulating specific transcripts, such as those mentioned above, in limiting clinical disease independent of *Mx1*'s previously described anti-IAV activities. However, additional studies are needed to better define whether the differential effect of the CAST/EiJ Mx1 allele are due to *Mx1*-associated signaling or more subtle effects on viral replication which subsequently affect inflammation and disease.

In a more general sense, these results illustrate the advantages provided by using a system such as the CC compared to using classical inbred strains such as the founder strains. Because of the inherent genome structure of the founder strains [47], it would be difficult to differentiate between the disassociated effects of the CAST/EiJ *Mx1* allele we found in the pre-CC population and the alternate hypothesis that CAST/EiJ's *Mx1* was completely non-functional, and that there was another polymorphic gene within CAST/EiJ that provided some protection from clinical disease. It is only through the recombination present within the CC, and the use of large populations of unique lines that can be evaluated within the CC that such hypothesis can be differentiated. This lack of structure across the genomes also allowed us to gain new insight into the relationships between coexpressed transcripts and disease outcomes, as well as the relationships between specific disease processes (see population-wide pattern section below).

Though the identification of *Mx1* served to validate our mapping study, the presence of a large effect allele within genetically variable populations can mask those with smaller effect sizes. Indeed, this genetic architecture is common to pathogen resistance, as several other large effect genes have been identified for viral (e.g. flaviviruses [71], mouse CMV [72], norovirus [20], HIV [18]), bacterial (NRAMP [73]) and parasitic (malaria [74]) diseases. Due to these genes of large effect, many studies of these pathogens have been conducted within susceptible models (e.g. mouse models of influenza infection are almost universally *Mx1*-/- models). It is likely that there are specific alleles influencing disease processes that only act in the context of the presence or absence of major resistance alleles (e.g. an allele that affected the degree of tissue repair would only act when there had been significant tissue damage, which a functional *Mx1* allele would prevent). To address this issue, we conducted further genome mapping while accounting for *Mx1* status using two complementary approaches: asking whether there are loci that act in addition to *Mx1* in regulating disease-associated phenotypes (*HrI2*), and also asking if there are loci that act only in the highly susceptible *Mx1* negative population (*HrI3, HrI4*). By doing so, we were able to identify three additional loci influencing weight loss, pulmonary edema and neutrophil infiltration into the airways, further validating the role of genetic variation at *HrI3* in contributing to pulmonary edema differences in a separate set of fully inbred CC animals. The development of pulmonary edema [6], [75], as well as neutrophilic infiltration [76] have been shown to contribute to disease severity and long-term lung disease in the human population. Our results show that host genetic variation not only contributes to direct responses to infection, but also to other aspects of the host response that can lead to long-term complications following infection.

In addition to allowing us to identify novel QTL that impacted the host response to IAV, our mapping using only *Mx1*-/- animals also allowed us to compare our study to other studies using QTL mapping within *Mx1*-/- panels [42], [45], [46]. While these studies all identified QTL contributing to IAV responses, there was no overlap between these QTL and the ones we discovered. This result is unsurprising given the differences in virus strains, phenotypes measured, and polymorphisms within the two panels. Nevertheless, the aggregate of these studies further strengthens the idea that virus-host interactions are highly complex, and that polymorphic host genes are critical for numerous responses. Our results further emphasize the unique genetic interactions that occur in specific sub-populations of infected individuals that regulate disease processes. While we limited our analysis to the *Mx1*-/- animals in the pre-CC population, due to sample size, it is likely that similar QTL can be identified in future work using animals with functional *Mx1*.

Ultimately, the goal of QTL mapping studies is to identify the causal polymorphic genes or genome features responsible for variation in disease processes. A variety of studies have used transcriptional data [42], [49] to narrow QTL regions into candidate genes. However, transcriptional analysis can be confounded by the dynamic nature of transcriptional responses, and can also be confounded by SNPs residing underneath the expression probes that can impact binding [77], [78]. An alternate approach, taking advantage of the allelic complexity of the CC is the use of the sequence of the founder strains [57] to interpret the mapping results and to prioritize candidate genes within QTL. This approach is quite powerful; as causative polymorphisms must be contained within QTL regions, and has been used effectively in other pre-CC studies [49], [55]. However, *Mx1* provides a cautionary note for regions of the genome in that there is a large in/del differentiating the eight CC founders. In fact, C57BL/6J, and thus the assembly, has the deletion. In such regions, functional genomic features may be misannotated and more importantly the genetic variants present in founders without the deletion is currently not annotated. This lack of annotation makes it difficult to conclusively dissect the polymorphisms within these regions that might cause phenotypic variation across the population, and currently requires more intensive sequencing efforts on a case-by-case basis. The *Mx1* result illustrates the importance of improving the annotation of genetic variants in the mouse genome. It also suggests that in addition of the processed lists of SNPs and in/dels available at the Mouse Genome Projects from the Sanger Institute, the analysis of the allele effect in QTL intervals should be analyzed by searching for signatures of structural variation that might be present in the raw reads. As further QTL analyses are undertaken within the CC system, approaches to narrow down onto candidate genes and polymorphisms will need to be further developed, likely integrating both transcriptional and refined analysis of sequence data to account for other potential causative genome features. These approaches will be facilitated by the interrogation of transcriptional activity at multiple time points in completely inbred CC lines.

### Population-wide patterns of host response to influenza infection

The pre-CC experiment also allowed us to identify specific CC lines that might be useful models for specific disease phenotypes (e.g. animals with high titers but little/no clinical disease as super-spreaders), as well as other interesting relationships between disease components across the pre-CC population. For example, while lung hemorrhage is a clinically important influenza-associated phenotype [79], we found that lung hemorrhage was only correlated with alveolar inflammation and not with other metrics of viral spread or disease severity. This result bears further study, but it suggests that while hemorrhage indicates a severe response to influenza infection, hemorrhage is governed by processes that are largely disassociated from those processes contributing to overall severity of infection at least through day 4. Results such as this further highlight the complexity of the host response to infection, and the need to consider genetically diverse populations when attempting to understand disease processes. While the individual pre-CC mice used within this study were insufficient to develop these new models of disease processes, as CC lines become increasingly available [52], utilization of specific CC lines with unique responses to infectious diseases might well become a critical resource for uncovering avenues of viral pathogenesis in specific subpopulations.

In addition, we identified transcriptional modules that correlated with overall disease severity, or with specific aspects of the host response to infection (e.g. inflammatory components, clinical disease). Recent efforts from a number of groups [10], [80] have focused on identifying markers associated with different disease states (e.g. protective vaccine responses, asymptomatic individuals) across human cohorts. While the nature of the pre-CC study (restricted to a single time-point) makes it difficult to draw broad conclusions about these results, it does suggest that there are unique transcriptional signatures relating to different aspects of the host response to infection. Future studies leveraging the full power of the CC (identical animals at different time points, compared to baseline transcriptional levels) will provide the opportunity for identification of molecular signatures of different disease-associated phenotypes, informing us both of the mechanisms through which these processes are occurring, as well as providing non-invasive diagnostic markers of various disease-related phenotypes.

The findings of this study also provide new insights into the relative contribution of viral replication versus virus-induced inflammation in the pathogenesis of influenza infection. There is conflicting evidence from a variety of *in vivo* studies as to the importance of virus-induced inflammation [28], [39], [64], [81], [82] and control of viral load [30], [39], [66] on disease severity. However, these studies have all used different mouse strains, influenza strains and experimental conditions, making direct comparisons difficult. The novel allele combinations in the pre-CC population allowed new insight by dissociating phenotypes that were correlated in the founder strains. This allowed us to assess the relative contribution of inflammation and viral replication on disease outcome. Consistent with the complex nature of virus-induced disease, we found that both viral replication and levels of inflammation were predictive of disease outcome independently of one another. Although the overall correlations between viral titer, weight loss and inflammation were reduced within the *Mx1*-/- subpopulation of pre-CC animals, we again found that both viral replication and levels of inflammation together were better predictors of disease outcome than either was alone. Consistent with this analysis, we identified CC lines that showed high levels of replication and weight loss, but little inflammation as well as lines with excessive inflammation and weight loss, but low viral titers suggesting that multiple pathways can lead to similar clinical disease outcomes across a genetically diverse population. Future studies utilizing this model with fully inbred CC lines, will allow us to more fully evaluate how the kinetics, magnitude, and duration of viral replication and inflammation contribute to disease outcome over the temporal course of the infection. These results illustrate the potential and the power of using genetically diverse mice to study the relative contribution of specific aspects of the pathogen and host response which together drive disease outcome.

### Summary

There is an increased appreciation for the role that host polymorphisms play in the host response to infectious diseases [83]. However, for a number of reasons, Genome Wide Association Studies (GWAS) of responses to acute infectious diseases within the human population have been difficult to conduct [84]. While future studies using the CC panel will allow for the evaluation of multiple animals/line and allow for integration of information across multiple timepoints, for this study we had access to only a single animal/line within the pre-CC population making it similar in design to GWAS and raising concerns about our ability to identify host response QTL within this study. However, the identification of several disease associated QTL, including a QTL containing the known IAV associated resistance gene *Mx1*, even when using a single mouse/time point, suggests that the CC lines represent a robust system for identifying polymorphic genes that regulate host responses to infectious diseases. As the CC can be recreated and manipulated, it will increasingly become a useful tool to a) identify candidate genes and pathways for more targeted association studies within human populations, and b) allow us to increase our understanding of how critical demographic and environmental factors, as well as specific genetic subpopulations impact some of the variability in GWAS studies of acute infectious diseases in humans.

Host genetic variation clearly plays an important role in regulating differential response phenotypes to infectious disease progression. Herein, we provide proof of concept and a framework for identifying the role of polymorphic genes on microbial pathogenesis using a genetically diverse population: underlying relationships between different disease phenotypes, genetic control of phenotypes following infection (both those of large effect, as well as those that modulate the host response), and transcriptional profiles that related to specific disease-associated phenotypes. In summary, this study shows that a genetically complex *in vivo* model represents a useful system for modeling pathogen interactions within genetically diverse populations and identifying novel genetic loci controlling multiple aspects of disease pathogenesis. Though this study had clear limitations, the pre-CC population provided the appropriate framework to develop the methodological approaches that resulted in the identification and prioritization of genes within novel disease loci. These results strongly support the hypothesis that studies using the fully inbred CC lines, with the use of replicate animals and evaluation of phenotypic variation during influenza infection over time, will be even more successful in identifying polymorphic genes that regulate multiple disease associated phenotypes including those phenotypes associated with adaptive immune responses and disease recovery. Furthermore, through careful selection of CC lines, studies can be designed to specifically investigate how interactions between allelic variants in two or more genes interact to influence complex phenotypic outcomes during infection.

## Methods

### Ethics statement

Mouse studies were performed in strict accordance with the recommendations in the Guide for the Care and Use of Laboratory Animals of the National Institutes of Health. All mouse studies were performed at the University of North Carolina (Animal Welfare Assurance # A3410-01) using protocols approved by the UNC Institutional Animal Care and Use Committee (IACUC). All studies were performed in a manner designed to minimize pain and suffering in infected animals, and any animals that exhibited severe disease signs was euthanized immediately in accordance with IACUC approved endpoints.

### Animals

8–16 week old female animals from the 8 founder strains (A/J, C57BL/6J, 129S1/SvImJ, NOD/ShiLtJ, NZO/HILtJ, CAST/EiJ, PWK/PhJ, and WSB/EiJ) were derived from the Jackson labs (jax.org), and bred at UNC Chapel Hill under specific pathogen free conditions. 8–12 week old female Pre-CC mice were bred at Oak Ridge National Laboratories under specific pathogen free conditions, and transferred directly into a BSL-3 containment laboratory at UNC Chapel Hill. Inbred CC mice were bred at UNC Chapel Hill under specific pathogen free conditions. All experiments were approved by the UNC Chapel Hill Institutional Animal Care and Use Committee.

### Virus and cell lines

The mouse adapted influenza A strain A/PR/8/34 (H1N1) was used for all infection studies. A/PR/8/34 stocks were made by infection of 10-day old embryonated chicken eggs. MDCK cells grown in high glucose Dulbecco's modified Eagle's medium (10% FBS, 1% Penicillin-Streptomycin) were used for titering virus.

### Infections

Animals were lightly anesthetized via inhalation with Isoflurane (Piramal, Bethlehem, Pa). Following anesthesia, animals were infected intranasally with 5×10∧2 pfu of PR8 in 50 µL of phosphate buffered saline (PBS), while mock infected animals received only 50 µL of PBS. Animals were assayed daily for morbidity (determined as % weight loss), mortality and clinical disease scores. At 4 days post infection, animals were euthanized via Isoflurane overdose and cardiac puncture, animals were assessed for gross pathology (lung hemorrhage and edema) and tissues were taken for various assays.

### TCID_50_ assay

MDCK cells were seeded into 96 well plates at a density of 1.5×10∧5 cells/well in DMEM (10% FBS, 1% Pen-strep) and incubated at 37 degrees overnight. Cells were washed 2 times with PBS, before addition of 100 µL of DMEM to each well. Media was removed from all wells in the 1^st^ column of the plate, and 146 uL of lung homogenate in DMEM was added to these wells (each biological sample was added to 4 wells). Serial dilutions of 46 µL (0.5 log dilutions) were carried out across the plate. Plates were incubated at 37 degrees C for 1 hour, inoculum was removed and 150 µL of serum free DMEM with 1 µg/mL of trypsin was added to each well. Plates were then incubated at 37oC for 3 days. Media was then removed, and wells were stained with a 1% Crystal Violet solution. The stain was washed off with water. Titer is determined as follows:
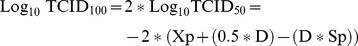
(1)Where Xp is the last dilution where all of the replicates of a given sample are positive, D is the serial dilution log and Sp is the sum of the proportion of replicates at all dilutions where positives are seen (starting with the Xp dilution).

### Histopathological analysis

The right lung was removed and submerged in 10% buffered formalin (Fischer) without inflation for 1 week before being submitted to the UNC Linberger Comprehensive Cancer Center histopathology core for processing. Two 5 micron thick Hematoxylin and Eosin stained lung sections (step-separated by 100 microns) were blind-scored by microscopic evaluation performed by two independent scorers for a variety of metrics relating to the extent and severity of immune cell infiltration and pathological damage on a 0–3 (none, mild, moderate, severe) scale.

### Immunohistochemical analysis of viral replication

For detection of influenza virus antigen, we used serial sections from formalin-fixed, paraffin-embedded lung samples. After deparaffinization and rehydration, antigen retrieval was performed using 0.1% protease (10 min at 37°C). Endogenous peroxidase was blocked with 3% hydrogen peroxide and slides were briefly washed with phosphate-buffered saline (PBS)/0.05% Tween 20. Mouse anti- influenza virus nucleoprotein (clone Hb65, ATCC) and horseradish peroxidase labeled goat anti-mouse IgG2a were used for 1 h at room temperature. Peroxidase activity was revealed by incubating slides in 3-amino-9-ethylcarbazole (AEC, Sigma) for 10 minutes, resulting in a bright red precipitate, followed by counterstaining with hematoxylin. Tissue sections from non-infected BALB/c mice and mouse IgG2a isotype antibody (R&D) were used as negative controls. The extent of influenza viral antigen spread across these slides was then scored in a blinded fashion on a 0–3 scale.

### RNA preparation and oligonucleotide microarray processing

At 4 days after infection, mice were killed and lung tissue harvested and placed in RNAlater (Applied Biosystems/Ambion, Austin, TX) and stored at −80°. The tissues were subsequently homogenized, and RNA extracted as previously described [85]. RNA samples were spectroscopically verified for purity, and the quality of the intact RNA was assessed using an Agilent 2100 Bioanalyzer. cRNA probes were generated from each sample by the use of an Agilent one-color Low Input Quick Amp Labeling Kit (Agilent Technologies, Santa Clara, CA). Individual cRNA samples were hybridized to Agilent mouse whole-genome oligonucleotide 4×44 microarrays according to manufacturer instructions. Samples from individual mice were evaluated to enable examination of animal-to-animal variation as part of the data analysis. Slides were scanned with an Agilent DNA microarray scanner, and the resulting images were analyzed using Agilent Feature Extractor version 8.1.1.1. The Agilent Feature Extractor software was used to perform image analysis, including significance of signal and spatial detrending and to apply a universal error model. For these hybridizations, the most conservative error model was applied. Raw data were then loaded into a custom-designed laboratory information management system (LIMS). Data were warehoused in a Labkey system (Labkey, Inc., Seattle, WA). Raw array data are available from GEO with accession GSE30506.

The Agilent arrays were background corrected by applying the Normal-Exponential convolution model [86] and normalized using quantile normalization [87] with the Agi4×44PreProcess Bioconductor package (www.bioconductor.org). The probes were filtered requiring that all probes meet specific QC requirements (probe intensity had to be found, well above background, not saturated, and not be nonuniformity or population outliers as defined by the standard parameters in Agi4×44PreProcess package) for all samples. Differential expression analysis was performed using the LIMMA Bioconductor package [88], and the false discovery rate was calculated using the qvalue Bioconductor package [89]. Probes were mapped to the mm9 genome using BLAT [90] requiring at least 98% identity. Probes that did not map, mapped to multiple locations equally well, or contained a high confidence single nucleotide polymorphism (SNP) from one of the eight progenitor strains from the Sanger Institute/Wellcome Trust mouse sequencing project [57] in the probe sequence were excluded from analysis. There were 11,700 probes passing QC and not potentially impacted by a SNP. The Gene Ontology (GO) analysis was performed using the standard hypergeometric test from the Gostats Bioconductor package [91] with a universe consisting of the unique genes from the probes entered into the DE analysis. Only the Biological Process subset of the Gene Ontology was used for testing. The Benjamini and Yekutieli false discovery rate (FDR) [92] was computed for the *P*-value distribution for this analysis to address dependencies inherent from the hierarchical/nested structure of the GO categories.

### De-novo network (module) analysis

For both the full analysis and the Mx1-/- analysis, six thousand probes were chosen to be entered into the analysis based on both high variability across samples as well as a measure of how connected they were [93]. Arrays were preprocessed separately for both analyses. These probes were used for the formation of coexpression modules through the weighted gene coexpression network analysis (WGCNA) procedure [93], [94]. Module formation was signed [95] and was carried out using the dynamicTreeCut R package [96] with pruning carried out based only on the dendrogram. All modules were checked for statistical significance through a permutation procedure whereby the mean topological overlap of those probes within a module was compared to the mean topological overlap of 10,000 random modules of the same size chosen from the initial set of 6,000 probes. Using the WGCNA package the module eigengene (first principle component of the expression matrix) for each module was computed [97]. The module eigengene can be viewed as the representative profile that summarizes the module expression profile. The module eigengene was first correlated with the clinical traits using Pearson's correlation with P-values provided as Student's asymptotic P-value. The module QTL scan was carried out similar to below but using the eigengene for each module as a phenotype. Specifically, each eigengene was regressed on the expected haplotype contribution from each of the eight founding inbred strains. Significance was assessed using the –log10 P-values (using a Bonferroni type correction (α = 0.05)) from the model and support intervals were computed using the 1.5 LOD drop method [54]. This method of defining an mQTL is essentially the same as a previous study using F2 intercrosses [53]. A related approach looking at overrepresentation of eQTLs in a module [98] could potentially be sensitive to significance cutoffs and module size and necessitates a full eQTL scan.

### Genotyping and haplotype reconstruction

Genotyping and haplotype reconstruction were done as described in [49]. Briefly, each pre-CC animal was genotyped using Mouse Diversity [47] test A-array at 181,752 well performing SNPs which were polymorphic across the founder strains. Once genotypes were determined (Dataset S2), founder strain haplotype probabilities were computed for all genotyped loci using the HAPPY algorithm [99]. Genetic map positions were based on the integrated mouse genetic map using mouse genome build 37 [100].

### Genome scans

Genome scans were run as described in [49]. Briefly, QTL mapping was conducted using the BAGPIPE package [101] to regress each phenotype on the computed haplotypes in the interval between adjacent genotype markers, producing a LOD score in each interval to evaluate significance. Genome-wide significance was determined by permutation test, with 250 permutations conducted per scan.

A more complex model was also used to control for *Mx1* status, whereby the null model included the haplotype information from the most significant marker at the *Mx1* locus (JAX00072951). LOD scores are then computed for each haplotype interval based on the increase in fit of genotype to phenotype when Mx1 haplotype is already taken into account.

### Identifying candidate regions

For the likely regions of identified QTL peaks, SNP data for the eight founder strains from the Sanger mouse genomes project was downloaded, and filtered to include only homozygous calls. In the case where a single founder strain allele drives a QTL peak, all private SNPs for that strain are candidates for the observed phenotype. In the case where multiple founder strains drive a QTL peak, the most likely hypothesis is that the causative polymorphism exists in a region of shared ancestry between these founder strains. SNPs were categorized into 3 classes: Consistent with a shared ancestry (SNPs where the driver strains share a private SNP), Inconsistent with a shared ancestry (SNPs where the driver strains share different alleles with other strains), and Uninformative with regards to ancestry (SNPs private to a single strain and SNPs shared by driver strains as well as others). Candidate regions were defined as regions containing at least one consistent SNP, and were bounded by the 1^st^ nucleotide after the last inconsistent SNP until the last nucleotide before the next inconsistent SNP, and also had to exceed 100 base pairs in length. We identified all annotated genes and non-coding RNAs that were within 500 bases of, or in consistent regions and classify these as our likely candidates.

### *Mx1* gene structure

To characterize the genetic variation at the *Mx1* locus we combined data from the mouse genome assembly, the Sanger Institute's Mouse Genomes sequencing project and a mouse full-length *Mx1* cDNA clone (CT010406). By aligning the cDNA to the *Mx1* genomic locus we identified three missing exons (exons 9, 10 and 11). We used this information to design primers to amplify and sequence every exon and the span the deletion boundaries in each strain (Table S14). The deletion occurs between positions 97674078 and 97674079 of the reference on chromosome 10. The deletion is 921 bp upstream from exon 12 and 302 bp downstream of exon 8. Chromosome walking was used to partially sequence the introns missing in the assembly. All sequence variants have been submitted to NCBI (GenBank Accession numbers: JQ860141-JQ860220).

### Real-time PCR analysis

Whole lung RNA from 8 week old female C57BL/6J, CAST/EiJ, and PWK/PhJ that had been either mock or flu infected were isolated using Trizol (Invitrogen, Carlsbad CA), and following their protocol. One microgram of total isolated RNA from each sample was reverse transcribed using MMLV-RT (Promega, Madison WI), and following their protocol. We ran TaqMan real time PCR with two primer-probe pairs (Applied Biosystems Foster City, CA): Mm01217999_m1 to amplify the 5′ gene region of Mx1 transcripts, and Hs03928985_g1 to amplify 18s mRNA. Fold-induction was calculated as the difference in expression levels for infected animals as compared to their strain-matched mock animals.

## Supporting Information

Dataset S1 **Phenotypes of Pre-CC animals.**
(CSV)

Dataset S2 **Genotypes of Pre-CC animals.**
(ZIP)

Figure S1 **Variation in weight loss curves across the eight founder strains of the Collaborative Cross.** Following infection with IAV, n = 5–12 animals from each of the eight founder strains had their weights recorded through four days post infection. Shown are weight loss curves (with standard deviations) for these eight strains (A/J (n = 11) = yellow, C57BL/6J (n = 6) = grey, 129S1/SvImJ (n = 5) = pink, NOD/ShiLtJ (n = 5) = dk. Blue, NZO/HILtJ (n = 12) = lt. blue, CAST/EiJ (n = 5) = green, PWK/PhJ (n = 5) = red, WSB/EiJ (n = 5) = purple).). The high variation present within NOD/ShiLtJ and CAST/EiJ was due to bimodal weight loss responses in each strain, and repeated in multiple experiments,
(TIF)

Figure S2 **QTL underlying weight loss following influenza infection.** (A) QTL scans showing LOD score (Y-axis) and genome position (X-axis) for weight loss. *HrI1* on chromosome 16 (dark peak) was identified and influenced many disease phenotypes We remapped weight loss after accounting for the large effect of *HrI1* in our QTL model, and identified another QTL, *HrI2* on chromosome 7 (light grey peak). Allele effects plot for (B) *HrI1* and (C) *HrI2*, showing the estimated contributions of each founder strain allele (y-axis) across the likely region for these loci (position in locus, x-axis) on weight loss. A phenotype by haplotype plot (D) showing the weight loss phenotypes for those animals that were homozygous for founder strain alleles at *HrI2* (A/J = yellow, C57BL/6J = grey, 129S1/SvImJ = pink, NOD/ShiLtJ = dk. blue, NZO/HILtJ = lt. blue, CAST/EiJ = green, PWK/PhJ = red, WSB/EiJ = purple).
(TIF)

Figure S3 **QTL underlying pulmonary edema following influenza infection.** (A) QTL scans within the *Mx1* -/- subpopulation (light grey line) identified a QTL, *HrI3* on chromosome 1, influencing pulmonary edema that was not detectable within the whole pre-CC population (black line). (B) Allele effects plot for *HrI3*, showing the estimated contributions of each founder strain allele across this locus on pulmonary edema. A phenotype by haplotype plot (C) showing the pulmonary edema score for those animals that were homozygous for founder strain alleles at *HrI3* (A/J = yellow, C57BL/6J = grey, 129S1/SvImJ = pink, NOD/ShiLtJ = dk. blue, NZO/HILtJ = lt. blue, CAST/EiJ = green, PWK/PhJ = red, WSB/EiJ = purple).
(TIF)

Figure S4 **QTL underlying neutrophil infiltration following influenza infection.** (A) QTL scans within the *Mx1* -/- subpopulation (light grey line) identified a QTL, *HrI4* on chromosome 15, influencing neutrophil infiltration that was not detectable within the whole pre-CC population (black line). (B) Allele effects plot for *HrI4*, showing the estimated contributions of each founder strain allele across this locus on neutrophil infiltration. A phenotype by haplotype plot (C) showing the neutrophil infiltrate score for those animals that were homozygous for founder strain alleles at *HrI4* (A/J = yellow, C57BL/6J = grey, 129S1/SvImJ = pink, NOD/ShiLtJ = dk. blue, NZO/HILtJ = lt. blue, CAST/EiJ = green, PWK/PhJ = red, WSB/EiJ = purple).
(TIF)

Table S1 **Mean (range) of IAV associated phenotypes comparing founder strains and the Pre-CC.**
(DOCX)

Table S2 **Phenotypic correlations across the pre-CC population.**
(DOCX)

Table S3 **Significance of predictors of D4 weight.**
(DOCX)

Table S4 **Transcripts within expression modules.**
(DOCX)

Table S5 **GO-term enrichment by module.**
(DOCX)

Table S6 ***Mx1*** **sequence variants.**
(DOCX)

Table S7 **Transcripts with an eQTL at** ***Mx1***.
(DOCX)

Table S8 **GO-Term enrichment of eQTL that differentiate the CAST/EiJ** ***Mx1*** **allele's effects.**
(DOCX)

Table S9 **Phenotypic mean (ranges) between entire pre-CC population and the** ***Mx1*** **-/- subpopulation.**
(DOCX)

Table S10 **Phenotypic correlations across the** ***Mx1*** **-/- pre-CC subpopulation.**
(DOCX)

Table S11 **Transcripts within modules in the** ***Mx1*** **-/- subpopulation.**
(DOCX)

Table S12 **GO-term enrichment by module in** ***Mx1*** **-/- subpopulation.**
(DOCX)

Table S13 **Transcriptional module correlates with influenza phenotypes in the** ***Mx1*** **-/- subpopulation.**
(DOCX)

Table S14 **Primers used in sequencing** ***Mx1*** **.**
(DOCX)
