# Supplementary figures and images for: Modeling Host Genetic Regulation of Influenza Pathogenesis in the Collaborative Cross
Source: PLoS Pathog. 2013 Feb 28;9(2):e1003196. doi: 10.1371/journal.ppat.1003196 (PMC3585141; doi:10.1371/journal.ppat.1003196)

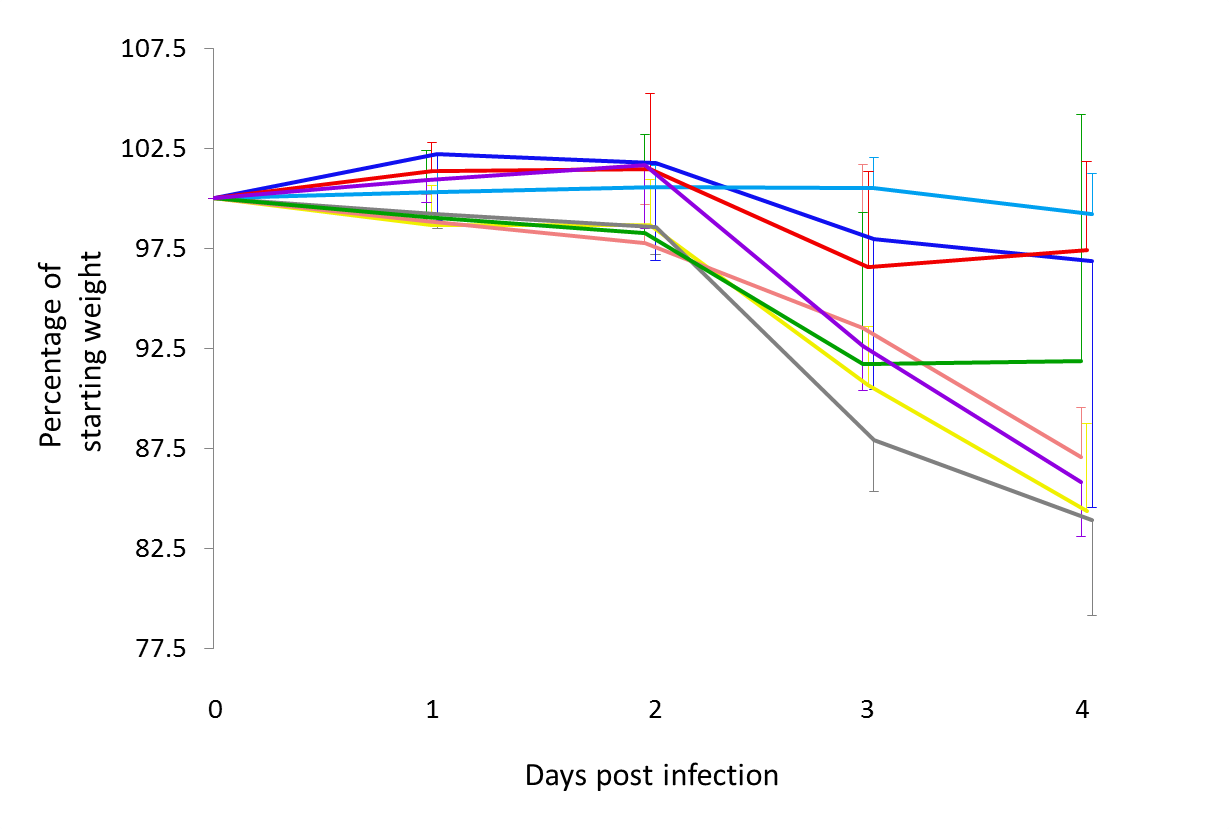

Supplement: Figure S1 — Variation in weight loss curves across the eight founder strains of the Collaborative Cross. Following infection with IAV, n = 5–12 animals from each of the eight founder strains had their weights recorded through four days post infection. Shown are weight loss curves (with standard deviations) for these eight strains (A/J (n = 11) = yellow, C57BL/6J (n = 6) = grey, 129S1/SvImJ (n = 5) = pink, NOD/ShiLtJ (n = 5) = dk. Blue, NZO/HILtJ (n = 12) = lt. blue, CAST/EiJ (n = 5) = green, PWK/PhJ (n = 5) = red, WSB/EiJ (n = 5) = purple).). The high variation present within NOD/ShiLtJ and CAST/EiJ was due to bimodal weight loss responses in each strain, and repeated in multiple experiments, (TIF) [file ppat.1003196.s003.tif]

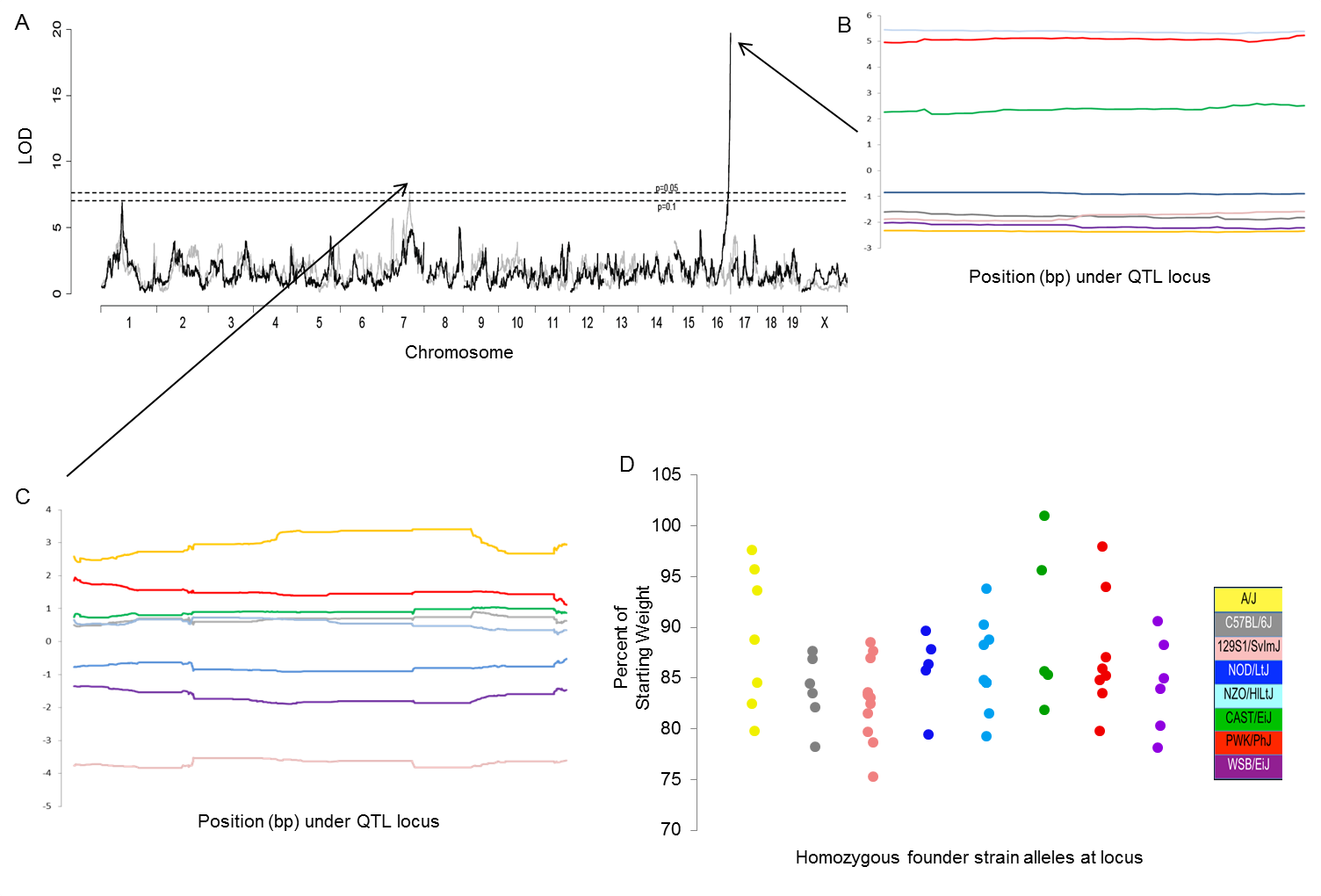

Supplement: Figure S2 — QTL underlying weight loss following influenza infection. (A) QTL scans showing LOD score (Y-axis) and genome position (X-axis) for weight loss. HrI1 on chromosome 16 (dark peak) was identified and influenced many disease phenotypes We remapped weight loss after accounting for the large effect of HrI1 in our QTL model, and identified another QTL, HrI2 on chromosome 7 (light grey peak). Allele effects plot for (B) HrI1 and (C) HrI2, showing the estimated contributions of each founder strain allele (y-axis) across the likely region for these loci (position in locus, x-axis) on weight loss. A phenotype by haplotype plot (D) showing the weight loss phenotypes for those animals that were homozygous for founder strain alleles at HrI2 (A/J = yellow, C57BL/6J = grey, 129S1/SvImJ = pink, NOD/ShiLtJ = dk. blue, NZO/HILtJ = lt. blue, CAST/EiJ = green, PWK/PhJ = red, WSB/EiJ = purple). (TIF) [file ppat.1003196.s004.tif]

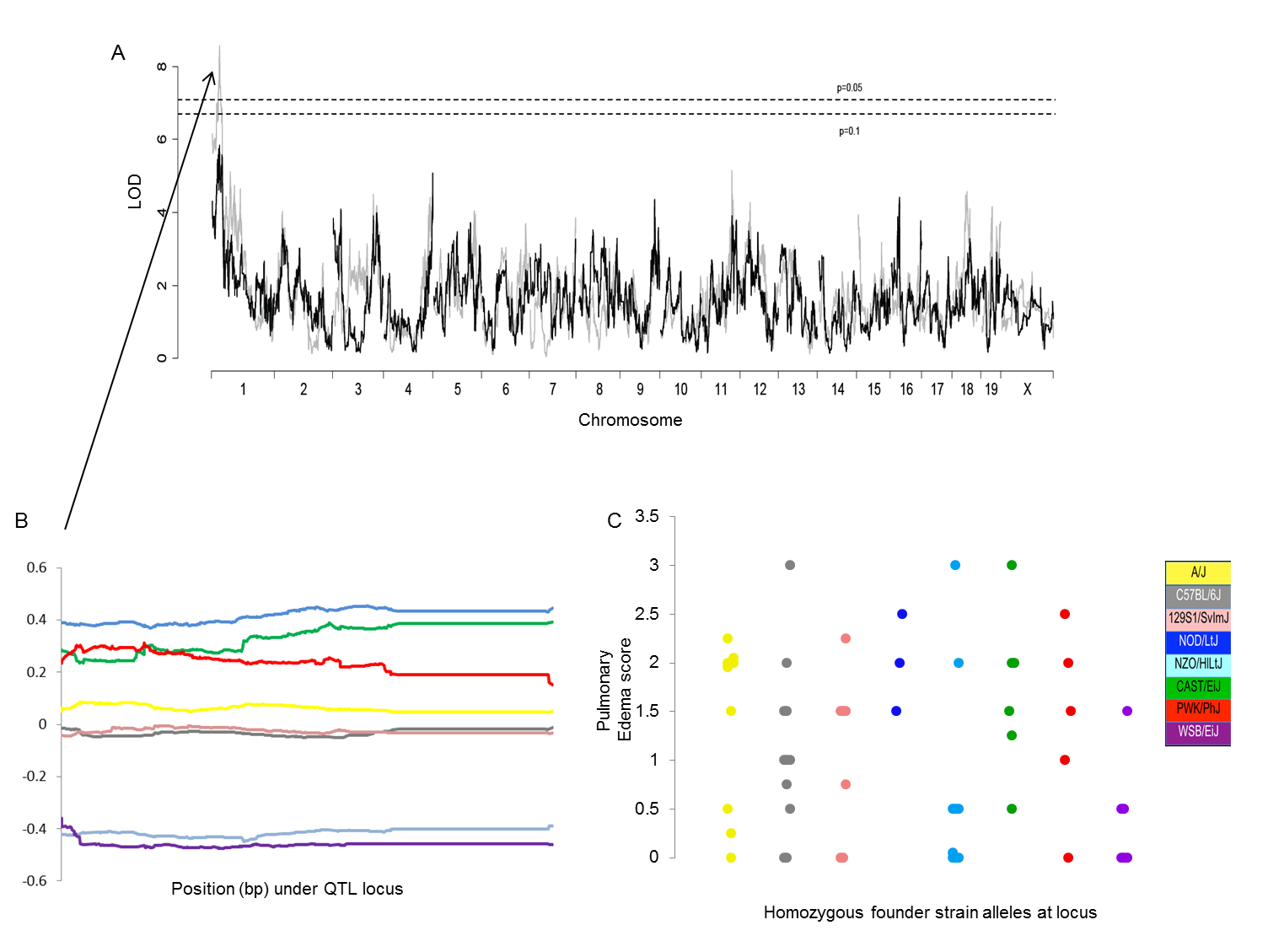

Supplement: Figure S3 — QTL underlying pulmonary edema following influenza infection. (A) QTL scans within the Mx1 -/- subpopulation (light grey line) identified a QTL, HrI3 on chromosome 1, influencing pulmonary edema that was not detectable within the whole pre-CC population (black line). (B) Allele effects plot for HrI3, showing the estimated contributions of each founder strain allele across this locus on pulmonary edema. A phenotype by haplotype plot (C) showing the pulmonary edema score for those animals that were homozygous for founder strain alleles at HrI3 (A/J = yellow, C57BL/6J = grey, 129S1/SvImJ = pink, NOD/ShiLtJ = dk. blue, NZO/HILtJ = lt. blue, CAST/EiJ = green, PWK/PhJ = red, WSB/EiJ = purple). (TIF) [file ppat.1003196.s005.tif]

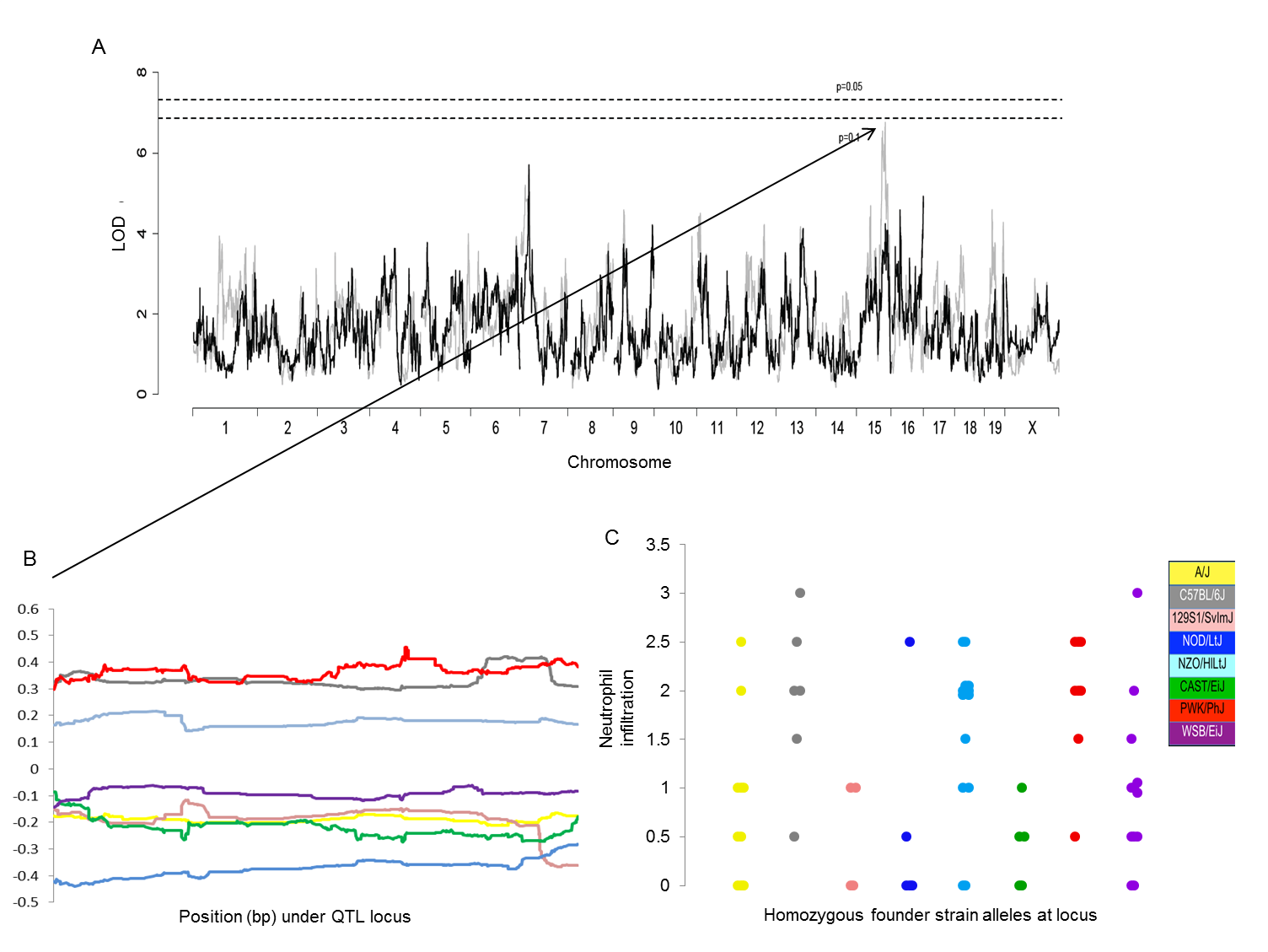

Supplement: Figure S4 — QTL underlying neutrophil infiltration following influenza infection. (A) QTL scans within the Mx1 -/- subpopulation (light grey line) identified a QTL, HrI4 on chromosome 15, influencing neutrophil infiltration that was not detectable within the whole pre-CC population (black line). (B) Allele effects plot for HrI4, showing the estimated contributions of each founder strain allele across this locus on neutrophil infiltration. A phenotype by haplotype plot (C) showing the neutrophil infiltrate score for those animals that were homozygous for founder strain alleles at HrI4 (A/J = yellow, C57BL/6J = grey, 129S1/SvImJ = pink, NOD/ShiLtJ = dk. blue, NZO/HILtJ = lt. blue, CAST/EiJ = green, PWK/PhJ = red, WSB/EiJ = purple). (TIF) [file ppat.1003196.s006.tif]
