# Supplementary material for: Modeling Host Genetic Regulation of Influenza Pathogenesis in the Collaborative Cross
Source: PLoS Pathog. 2013 Feb 28;9(2):e1003196. doi: 10.1371/journal.ppat.1003196 (PMC3585141; doi:10.1371/journal.ppat.1003196)
Supplement: Table S1 — Mean (range) of IAV associated phenotypes comparing founder strains and the Pre-CC. (DOCX) [file ppat.1003196.s007.docx]

| **Table S1. Mean (Range) of IAV associated phenotypes comparing founder strains and the Pre-CC** | | | | | | | | |
| --- | --- | --- | --- | --- | --- | --- | --- | --- |
|  | D4 weight^***^ | D4 clinical^***^ | Log titer^***^ | Airway infiltration^*^ | Airway debris^**^ | Vascular  infiltration^***^ | Alveolar infiltration^***^ | Alveolar debris |
| A/J | 85.7 (80.14-100.14) | 1.14  (0-2.5) | 5  (BDL^#^-6.47) | 1.17  (0.5-1.5) | 2 | 1.17  (0.5-1.5) | 1 | 0.5  (0-1) |
| C57BL6/J | 83.91 (79.72-93.0) | 0.5  (0-1) | 5.01 (3.97-5.48) | 1 (0.5-2) | 1.33 (0.5-2) | 1.33 (0.5-2) | 0.17 (0-0.5) | 0 |
| 129S1/SvImJ | 87.09 (84.76-91.14) | 1.1  (0-2) | 5.75 (4.85-6.1) | 2.67 (2-3) | 2.67 (2.5-3) | 1.67 (1.5-2) | 1.33 (0.5-2) | 0.67 (0.5-1) |
| NOD/SHiLtJ | 96.86 (82.99-111.39) | 0.3  (0-1) | 2.36 (BDL-5.97) | 1.33  (0.5-2) | 0.33  (0-1) | 0.67 (0.5-1) | 0.67 (0.5-1) | 0.33 (0-0.5) |
| NZO/HiLtJ | 99.22 (95.85-102.16) | 0.08  (0-0.5) | 1.06 (BDL-4.1) | 0.33  (0-0.5) | 0.33  (0-0.5) | 0.33 (0-0.5) | 0 | 0 |
| CAST/EiJ | 91.87 (79.05-100.33) | 2.3  (0.5-3) | 5.19 (4.6-6.1) | 0.67  (0.5-1) | 0.67 (0.5-1) | 1 (0.5-1.5) | 1.33 (1-1.5) | 0.33 (0-0.5) |
| PWK/PhJ | 97.39 (91.8-102.33) | 0 | 2.01 (BDL-3.86) | 1.5 | 0.67 (0.5-1) | 1.33 (1-1.5) | 0.33 (0-1) | 0.17(0-0.5) |
| WSB/EiJ | 85.83 (81.98-89.11) | 3  (2.5-4) | 5.55 (4.86-5.97) | 1.17(1-1.5) | 1.33 (0.5-2) | 0.5 | 0.17(0-0.5) | 0.17(0-0.5) |
| Pre-CC | 89.97 (75.02-109.11) | 1.4  (0-3.5) | 4.18 (BDL-6.34) | 1.48  (0-2.875) | 1.41  (0-3) | 1.58 (0-3) | 0.83(0-3) | 0.5(0-2) |
| Star next to phenotype=significant variation amongst founder strains in phenotype, ^*^p=0.1, ^**^p=0.05, ^***^p<0.01 (ANOVA for D4 weight and Log titer, Kruskal-Wallace for other phenotypes)  ^#^Below Detectable Limit (Samples with a titer lower than log_10_ 1.87 were undetectable) | | | | | | | | |
