# Supplementary material for: Modeling Host Genetic Regulation of Influenza Pathogenesis in the Collaborative Cross
Source: PLoS Pathog. 2013 Feb 28;9(2):e1003196. doi: 10.1371/journal.ppat.1003196 (PMC3585141; doi:10.1371/journal.ppat.1003196)
Supplement: Table S2 — Phenotypic correlations across the pre-CC population. (DOCX) [file ppat.1003196.s008.docx]

| **Table S2. Phenotypic correlations across the pre-CC popluation** | | | | | | | | | | | | | | | | |
| --- | --- | --- | --- | --- | --- | --- | --- | --- | --- | --- | --- | --- | --- | --- | --- | --- |
|  | Clinical Disease | | | | Viral replication | | Inflammatory cell  infiltrates | | | | | | | Pathology | | |
|  | D4 weight | D4 clinical | Hem | Gross Edema | Log titer | IHC score | Airway inflam | Airway neut | Airway mono | Vasc inflam | Vasc neut | Vasc mono | Alve inflam | Airway damage | Alve damage | Pulm  Edema |
| Fibrin | -0.15 | 0.12 | 0.05 | 0.07 | 0.24 | 0.31 | 0.19 | 0.17 | 0.17 | 0.09 | 0.08 | 0.2 | 0.07 | 0.15 | 0.14 | 0.27 |
| Pulm Edema | -0.16 | 0.12 | - 0.01 | -0.01 | 0.1 | 0.18 | 0.16 | 0.23 | 0.18 | 0.09 | 0.2 | 0.2 | 0.07 | 0.22 | 0.1 |  |
| Alve Damage | -0.16 | 0.12 | -0.03 | 0.1 | 0.08 | 0.23 | 0.26 | 0.17 | 0.1 | 0.17 | 0.18 | 0.06 | 0.4 | 0.22 |  |  |
| Airway Damage | -0.48 | 0.34 | -0.01 | 0.22 | 0.39 | 0.45 | 0.55 | 0.4 | 0.46 | 0.31 | 0.33 | 0.42 | 0.08 |  |  |  |
| Alve Inflam | -0.1 | 0.05 | 0.18 | -0.04 | 0.07 | 0.18 | 0.18 | 0.23 | 0.21 | 0.18 | 0.17 | 0.18 |  |  |  |  |
| Vasc Mono | -0.22 | 0.17 | 0 | 0.06 | 0.3 | 0.28 | 0.4 | 0.42 | 0.89 | 0.22 | 0.4 |  |  |  |  |  |
| Vasc Neut | -0.25 | 0.19 | -0.04 | 0.03 | 0.15 | 0.23 | 0.38 | 0.72 | 0.4 | 0.26 |  |  |  |  |  |  |
| Vasc Inflam | -0.17 | 0.06 | 0.05 | -0.08 | 0.09 | 0.2 | 0.43 | 0.26 | 0.21 |  |  |  |  |  |  |  |
| Airway Mono | -0.22 | 0.13 | 0 | 0.12 | 0.28 | 0.28 | 0.46 | 0.43 |  |  |  |  |  |  |  |  |
| Airway Neut | -0.31 | 0.18 | -0.05 | 0.1 | 0.2 | 0.27 | 0.44 |  |  |  |  |  |  |  |  |  |
| Airway inflam | -0.33 | 0.27 | 0.02 | 0.16 | 0.3 | 0.43 |  |  |  |  |  |  |  |  |  |  |
| IHC  score | -0.6 | 0.5 | 0.06 | 0.19 | 0.55 |  |  |  |  |  |  |  |  |  |  |  |
| Log titer | -0.62 | 0.45 | 0.1 | 0.13 |  |  |  |  |  |  |  |  |  |  |  |  |
| Gross Edema | -0.18 | 0.15 | -0.01 |  |  |  |  |  |  |  |  |  |  |  |  |  |
| Hem | -0.09 | 0.07 |  |  |  |  |  |  |  |  |  |  |  |  |  |  |
| D4 clinical | -0.54 |  |  |  |  |  |  |  |  |  |  |  |  |  |  |  |
| Correlations are Spearman’s rho. Blue shading indicate significance, lt. blue p<0.05, med blue p<0.001, dk blue p<0.00001 Abbreviations: inflam=inflammatory cell infiltrates, neut = neutrophils, mono = monocytes, hemorrhage=hem, pulm=pulmonary, alve=alveolar, vasc=vascular | | | | | | | | | | | | | | | | |
