# Supplementary material for: Modeling Host Genetic Regulation of Influenza Pathogenesis in the Collaborative Cross
Source: PLoS Pathog. 2013 Feb 28;9(2):e1003196. doi: 10.1371/journal.ppat.1003196 (PMC3585141; doi:10.1371/journal.ppat.1003196)
Supplement: Table S3 — Significance of predictors of D4 weight. (DOCX) [file ppat.1003196.s009.docx]

| **Table S3. Significance of predictors of D4 weight** | | | | |
| --- | --- | --- | --- | --- |
|  | Model | Significance | AIC | Full model better fit? |
| Full population | D4 weight = Log titer | <2.2e-16 | 995.85 | p=0.0003854 |
|  | D4 weight =Airway inflammation | 1.556e-9 | 1035.3 | p=8.588e-13 |
|  | D4 weight = Log titer + Airway inflammation | ^NA^ | 984.95 | ^NA^ |
| *Mx1*-/- subpopulation | D4 weight = Log titer | 3.939e-05 | 619.17 | p=0.01242 |
|  | D4 weight = Airway inflammation | 0.001418 | 626.07 | p=0.0003287 |
|  | D4 weight = Log titer + Airway inflammation | ^NA^ | 614.7 | ^NA^ |
