# Supplementary material for: Modeling Host Genetic Regulation of Influenza Pathogenesis in the Collaborative Cross
Source: PLoS Pathog. 2013 Feb 28;9(2):e1003196. doi: 10.1371/journal.ppat.1003196 (PMC3585141; doi:10.1371/journal.ppat.1003196)
Supplement: Table S4 — Transcripts within expression modules. (DOCX) [file ppat.1003196.s010.docx]

| **Table S4. Transcripts within expression modules** | |
| --- | --- |
| **Module** | **Transcript** |
| A | 2410042D21Rik |
| A | 2610020O08Rik |
| A | 2610207I05Rik |
| A | 3110001A13Rik |
| A | 4632404H12Rik |
| A | 4933407C03Rik |
| A | A230046K03Rik |
| A | A430106J12Rik |
| A | A630018P17Rik |
| A | Abcb7 |
| A | Abl2 |
| A | Abt1 |
| A | Adamts1 |
| A | AK035139 |
| A | AK037061 |
| A | AK044844 |
| A | AK050842 |
| A | AK051007 |
| A | AK081596 |
| A | Akap13 |
| A | Ankib1 |
| A | Ap4e1 |
| A | Apbb1ip |
| A | Arl6ip6 |
| A | B230369F24Rik |
| A | BC031781 |
| A | BC071254 |
| A | BF228116 |
| A | Blzf1 |
| A | Bzw1 |
| A | C030046E11Rik |
| A | C920006C10Rik |
| A | Cald1 |
| A | Ccdc25 |
| A | Ccdc55 |
| A | Cd44 |
| A | Cdkl3 |
| A | Cgn |
| A | Chd1 |
| A | Chm |
| A | Crk |
| A | Crkrs |
| A | Cul5 |
| A | Cwf19l1 |
| A | D030022P06Rik |
| A | Dcun1d3 |
| A | Ddhd1 |
| A | Ddx21 |
| A | Dnajc2 |
| A | Dnajc2 |
| A | Dnm2 |
| A | E130014J05Rik |
| A | E2f3 |
| A | E330009E22Rik |
| A | E430025E21Rik |
| A | Eea1 |
| A | EG245436 |
| A | Eif2ak1 |
| A | Eif3s1 |
| A | Eif4enif1 |
| A | Eif4g1 |
| A | Esf1 |
| A | Fgd4 |
| A | Fkbp15 |
| A | Flnb |
| A | Gabpb1 |
| A | Gcnt2 |
| A | Gltscr2 |
| A | Gmeb1 |
| A | Gnas |
| A | Gosr1 |
| A | Gprk6 |
| A | Gripap1 |
| A | Gtf2h1 |
| A | Gtpbp4 |
| A | Gtpbp4 |
| A | Heatr1 |
| A | Hipk1 |
| A | Hk1 |
| A | Hnrpdl |
| A | Hspa9 |
| A | Jarid2 |
| A | Keap1 |
| A | Klhl18 |
| A | Kpna4 |
| A | Lypla1 |
| A | Map3k1 |
| A | Mapk6 |
| A | Mapk9 |
| A | Mdm4 |
| A | Mpp1 |
| A | Mpp6 |
| A | Mtpn |
| A | NAP045236-1 |
| A | NAP060490-1 |
| A | Nfkbil1 |
| A | Nfyb |
| A | Nol10 |
| A | Nrbp1 |
| A | Nudt4 |
| A | Nupl1 |
| A | Nupl1 |
| A | Osbpl8 |
| A | Pa2g4 |
| A | Pcf11 |
| A | Pctk2 |
| A | Pdlim5 |
| A | Pfkfb3 |
| A | Picalm |
| A | Pik3c2a |
| A | Pitpnm2 |
| A | Plekha1 |
| A | Pmm2 |
| A | Prdm4 |
| A | Prpf38b |
| A | Pscdbp |
| A | Ptk2b |
| A | Pus3 |
| A | Rab22a |
| A | Rbm26 |
| A | Riok1 |
| A | Rkhd2 |
| A | Rmrp |
| A | Rnf111 |
| A | Rnf12 |
| A | Rnf24 |
| A | Rnf38 |
| A | Rnf4 |
| A | Rrbp1 |
| A | Rrp1b |
| A | Rsl1d1 |
| A | Rtn4 |
| A | Rufy1 |
| A | Sat1 |
| A | Scamp2 |
| A | Sh3kbp1 |
| A | Sirpa |
| A | Skil |
| A | Skil |
| A | Slc4a7 |
| A | Smad1 |
| A | Smg7 |
| A | Socs7 |
| A | Spats2 |
| A | Spred1 |
| A | Spred1 |
| A | Ssr3 |
| A | Stat3 |
| A | Stx2 |
| A | Syt11 |
| A | Taf15 |
| A | Taf7 |
| A | Tbc1d23 |
| A | Tgs1 |
| A | Thex1 |
| A | Ube1l2 |
| A | Ube1x |
| A | Usp12 |
| A | Usp38 |
| A | Utp14a |
| A | Vps54 |
| A | Wdr37 |
| A | Wdr43 |
| A | Wsb1 |
| A | Zc3h7a |
| A | Zcchc6 |
| A | Zfp597 |
| A | Zubr1 |
| B | 6-Mar |
| B | 4-Sep |
| B | 11-Sep |
| B | 0610007C21Rik |
| B | 0610009O20Rik |
| B | 1110018J18Rik |
| B | 1110031B06Rik |
| B | 1110031B06Rik |
| B | 1110051M20Rik |
| B | 1110054M08Rik |
| B | 1190002N15Rik |
| B | 1200009O22Rik |
| B | 1200016B10Rik |
| B | 1300010F03Rik |
| B | 1500005A01Rik |
| B | 1500041B16Rik |
| B | 1700012H05Rik |
| B | 1700019E19Rik |
| B | 1700020I14Rik |
| B | 1700040I03Rik |
| B | 1700055M20Rik |
| B | 1810011O10Rik |
| B | 1810021J13Rik |
| B | 1810026B05Rik |
| B | 1810037C20Rik |
| B | 2010003O02Rik |
| B | 2010100O12Rik |
| B | 2210011C24Rik |
| B | 2310014D11Rik |
| B | 2310022B05Rik |
| B | 2310040A07Rik |
| B | 2310046K01Rik |
| B | 2310067B10Rik |
| B | 2410081M15Rik |
| B | 2510003E04Rik |
| B | 2510048L02Rik |
| B | 2510048L02Rik |
| B | 2610003J06Rik |
| B | 2610019F03Rik |
| B | 2610034N15Rik |
| B | 2610204K14Rik |
| B | 2610301F02Rik |
| B | 2700049A03Rik |
| B | 2700049A03Rik |
| B | 2700089E24Rik |
| B | 2810025M15Rik |
| B | 2810432L12Rik |
| B | 2810482I07Rik |
| B | 2810488G03Rik |
| B | 2900002H16Rik |
| B | 2900009J20Rik |
| B | 2900024O10Rik |
| B | 2900024O10Rik |
| B | 2900041A09Rik |
| B | 3110004L20Rik |
| B | 3110004L20Rik |
| B | 3321401G04Rik |
| B | 3526401B18Rik |
| B | 3732413I11Rik |
| B | 4631416L12Rik |
| B | 4631427C17Rik |
| B | 4732479N06Rik |
| B | 4921511K06Rik |
| B | 4921517N04Rik |
| B | 4930535B03Rik |
| B | 4930570C03Rik |
| B | 4932442K08Rik |
| B | 4933404M19Rik |
| B | 4933406E20Rik |
| B | 4933413A10Rik |
| B | 4933439F18Rik |
| B | 5330417C22Rik |
| B | 5330421C15Rik |
| B | 5730403B10Rik |
| B | 5730406M06Rik |
| B | 5730446D14Rik |
| B | 5730601F06Rik |
| B | 5830428H23Rik |
| B | 5830434P21Rik |
| B | 5830454D03Rik |
| B | 6720458D17Rik |
| B | 8430408J07Rik |
| B | 8430408O14 |
| B | 8430415E04Rik |
| B | 8430416G17Rik |
| B | 9030607L17Rik |
| B | 9030612M13Rik |
| B | 9130221D24Rik |
| B | 9430020K01Rik |
| B | 9430079B08Rik |
| B | 9630050M13Rik |
| B | 9630058J23Rik |
| B | 9830163H01Rik |
| B | 9930024M15Rik |
| B | A_51_P486046 |
| B | A_52_P549754 |
| B | A_52_P836776 |
| B | A530089I17Rik |
| B | A730055C05Rik |
| B | A930008G19Rik |
| B | Aass |
| B | Abca1 |
| B | Abca2 |
| B | Abca3 |
| B | Abca3 |
| B | Abca5 |
| B | Abcc5 |
| B | Abcd3 |
| B | Abcd3 |
| B | Abi2 |
| B | Ablim1 |
| B | Ablim3 |
| B | Acbd4 |
| B | Acbd5 |
| B | Acot1 |
| B | Acox1 |
| B | Acp6 |
| B | Acss2 |
| B | Acvr2a |
| B | Acvr2b |
| B | Adamts10 |
| B | Adamtsl5 |
| B | Adarb1 |
| B | Adarb1 |
| B | Adcy9 |
| B | Add3 |
| B | Adk |
| B | Adrbk2 |
| B | Aes |
| B | AF143539 |
| B | Afap1l1 |
| B | Agbl3 |
| B | Agbl3 |
| B | Ager |
| B | Agrn |
| B | Agtr1a |
| B | Agtrl1 |
| B | Ahnak |
| B | Ahnak |
| B | AI464131 |
| B | AI851716 |
| B | AK011803 |
| B | AK020422 |
| B | AK028004 |
| B | AK031258 |
| B | AK034355 |
| B | AK040606 |
| B | AK042559 |
| B | AK043317 |
| B | AK045702 |
| B | AK048091 |
| B | AK049403 |
| B | AK051672 |
| B | AK051961 |
| B | AK052002 |
| B | AK054054 |
| B | AK054507 |
| B | AK078322 |
| B | AK078885 |
| B | AK079732 |
| B | AK080164 |
| B | AK082505 |
| B | AK085773 |
| B | AK086814 |
| B | AK087708 |
| B | AK089858 |
| B | AK142427 |
| B | Akap11 |
| B | Akap8l |
| B | Aktip |
| B | Aktip |
| B | Alas1 |
| B | Aldh2 |
| B | Aldh3a2 |
| B | Aldh6a1 |
| B | Als2cr13 |
| B | Als2cr2 |
| B | Amph |
| B | Anapc11 |
| B | Angptl2 |
| B | Ank3 |
| B | Ank3 |
| B | Ankrd12 |
| B | Ankrd15 |
| B | Ankrd25 |
| B | Ankrd25 |
| B | Ankrd40 |
| B | Ankrd44 |
| B | Ankrd46 |
| B | Ankrd47 |
| B | Ankrd50 |
| B | Anks1 |
| B | Antxr2 |
| B | Aox1 |
| B | Aox3 |
| B | Apc |
| B | App |
| B | Appl1 |
| B | Aprin |
| B | Aqp1 |
| B | Araf |
| B | Arhgap12 |
| B | Arhgap21 |
| B | Arhgap29 |
| B | Arhgap6 |
| B | Arhgef12 |
| B | Arhgef15 |
| B | Arhgef18 |
| B | Arhgef6 |
| B | Arid4b |
| B | Arl2bp |
| B | Arl3 |
| B | Arrdc3 |
| B | Arvcf |
| B | Asb1 |
| B | Asgr1 |
| B | Atbf1 |
| B | Atg16l2 |
| B | Atp1b1 |
| B | Atpif1 |
| B | Atrn |
| B | Atrnl1 |
| B | Atxn1 |
| B | AU040829 |
| B | AU042671 |
| B | Auh |
| B | AV154513 |
| B | AW061290 |
| B | AW061290 |
| B | AW456874 |
| B | Axin2 |
| B | Aytl2 |
| B | B230219D22Rik |
| B | B230333C21Rik |
| B | B230339M05Rik |
| B | B230380D07Rik |
| B | B930008K04Rik |
| B | B930037P14Rik |
| B | Bace1 |
| B | Bahcc1 |
| B | Bambi |
| B | BB128963 |
| B | Bbs9 |
| B | BC010787 |
| B | BC021381 |
| B | BC030336 |
| B | BC031353 |
| B | BC031748 |
| B | BC037121 |
| B | BC038156 |
| B | BC039093 |
| B | BC043118 |
| B | BC054438 |
| B | BC059842 |
| B | BC060632 |
| B | Bcas3 |
| B | Bcl2l2 |
| B | BE650457 |
| B | Bex4 |
| B | Bhlhb9 |
| B | Bmf |
| B | Bmpr1a |
| B | Bmpr2 |
| B | Bnip3l |
| B | Bpgm |
| B | Braf |
| B | Braf |
| B | Brwd1 |
| B | Btbd3 |
| B | Btbd3 |
| B | Btbd6 |
| B | Btd |
| B | BU530502 |
| B | BU531328 |
| B | BU554808 |
| B | BU557601 |
| B | C030015A19Rik |
| B | C130074G19Rik |
| B | C1qtnf2 |
| B | C1qtnf7 |
| B | C230078M08Rik |
| B | C230091D08Rik |
| B | C230094B09Rik |
| B | C85492 |
| B | Cab39l |
| B | Cacnb2 |
| B | Cadm1 |
| B | Cadm1 |
| B | Calcoco1 |
| B | Calcrl |
| B | Calcrl |
| B | Cand1 |
| B | Car4 |
| B | Card10 |
| B | Cars2 |
| B | Casd1 |
| B | Cat |
| B | Cav1 |
| B | Cav2 |
| B | CB845695 |
| B | Cbfa2t3h |
| B | Cbfa2t3h |
| B | Cbr2 |
| B | Cbx6 |
| B | Cbx6 |
| B | Cbx6 |
| B | Cbx7 |
| B | Ccdc117 |
| B | Ccni |
| B | Ccpg1 |
| B | Ccpg1 |
| B | Ccpg1 |
| B | Ccpg1 |
| B | Cd36 |
| B | Cd81 |
| B | Cd9 |
| B | Cd97 |
| B | Cdadc1 |
| B | Cdadc1 |
| B | Cdc25b |
| B | Cdc42bpa |
| B | Cdc42ep3 |
| B | Cdh13 |
| B | Cds2 |
| B | Centb2 |
| B | Centd3 |
| B | Ces3 |
| B | Chd3 |
| B | Chd6 |
| B | Clec14a |
| B | Clec14a |
| B | Clic3 |
| B | Clic5 |
| B | Clstn1 |
| B | Clstn2 |
| B | Cobl |
| B | Cobll1 |
| B | Col4a3bp |
| B | Col4a4 |
| B | Cplx2 |
| B | Cpm |
| B | Crbn |
| B | Crebl2 |
| B | Creg1 |
| B | Creg1 |
| B | Crim1 |
| B | Crip1 |
| B | Crip2 |
| B | Cryl1 |
| B | Csad |
| B | Csad |
| B | Csde1 |
| B | Cst3 |
| B | Ctdspl |
| B | Ctf1 |
| B | Ctnnb1 |
| B | Ctnnd1 |
| B | Cttnbp2 |
| B | Cugbp2 |
| B | Cxcl15 |
| B | Cxx1c |
| B | Cyb5b |
| B | Cyb5r3 |
| B | Cybrd1 |
| B | Cyhr1 |
| B | Cyp2b9 |
| B | Cyp2b9 |
| B | Cyp2d22 |
| B | Cyp39a1 |
| B | Cys1 |
| B | D10Ertd610e |
| B | D12Ertd647e |
| B | D14Ertd436e |
| B | D19Wsu12e |
| B | D1Ertd161e |
| B | D1Ertd161e |
| B | D230025D16Rik |
| B | D430015B01Rik |
| B | D4Bwg0951e |
| B | D4Wsu53e |
| B | D630014A15Rik |
| B | D730040F13Rik |
| B | D830024N08Rik |
| B | D930005D10Rik |
| B | Daam2 |
| B | Dab2ip |
| B | Dach1 |
| B | Dag1 |
| B | Dag1 |
| B | Dchs1 |
| B | Dcun1d2 |
| B | Ddc |
| B | Ddi2 |
| B | Ddr2 |
| B | Ddx26b |
| B | Deb1 |
| B | Decr1 |
| B | Dennd2a |
| B | Dgka |
| B | Dguok |
| B | Dhrs3 |
| B | Dip2b |
| B | Dixdc1 |
| B | Dmd |
| B | Dmn |
| B | Dnajb4 |
| B | Dnajc3a |
| B | Dnase2a |
| B | Dnase2a |
| B | Dnm3 |
| B | Dock7 |
| B | Dpp4 |
| B | Dpp4 |
| B | Dpyd |
| B | Dsp |
| B | Dst |
| B | Dstn |
| B | Dtna |
| B | Dync1li2 |
| B | Dynll2 |
| B | Dynll2 |
| B | Dynlt3 |
| B | E130308A19Rik |
| B | E130308A19Rik |
| B | E330009J07Rik |
| B | Ecm2 |
| B | Ednrb |
| B | Efemp1 |
| B | EG639396 |
| B | EG639426 |
| B | EG665123 |
| B | Egflam |
| B | Ehbp1 |
| B | Ehd4 |
| B | Eif2c1 |
| B | Eif2c4 |
| B | Eif2c4 |
| B | Eif2c4 |
| B | Eif4a2 |
| B | Eif4ebp2 |
| B | Elovl1 |
| B | Elovl5 |
| B | Eml1 |
| B | Emp2 |
| B | Enpep |
| B | ENSMUSG00000052439 |
| B | ENSMUSG00000054178 |
| B | ENSMUSG00000073981 |
| B | ENSMUST00000030142 |
| B | ENSMUST00000032357 |
| B | ENSMUST00000035300 |
| B | ENSMUST00000050697 |
| B | ENSMUST00000054524 |
| B | ENSMUST00000094652 |
| B | ENSMUST00000100305 |
| B | Epas1 |
| B | Epb4.1 |
| B | Epb4.1l4a |
| B | Epb4.1l5 |
| B | Epha1 |
| B | Ephb4 |
| B | Epm2aip1 |
| B | Epn2 |
| B | Epn2 |
| B | Eps15 |
| B | Eps8l1 |
| B | Erbb3 |
| B | Erg |
| B | Es22 |
| B | Etl4 |
| B | Etv1 |
| B | Etv5 |
| B | Evc2 |
| B | Evi1 |
| B | Faah |
| B | Fads1 |
| B | Fahd2a |
| B | Fasn |
| B | Fat4 |
| B | Fbxl16 |
| B | Fbxo10 |
| B | Fbxo10 |
| B | Fbxo25 |
| B | Fbxo3 |
| B | Fbxo8 |
| B | Fbxw8 |
| B | Fcgrt |
| B | Fchsd2 |
| B | Fcmd |
| B | Fech |
| B | Fgf1 |
| B | Fgf18 |
| B | Fgfr2 |
| B | Fgfr3 |
| B | Fgfr3 |
| B | Fgfr4 |
| B | Fgfr4 |
| B | Fhl1 |
| B | Figf |
| B | Fign |
| B | Fmo3 |
| B | Fmo5 |
| B | Fnbp1l |
| B | Fnta |
| B | Foxo1 |
| B | Foxp1 |
| B | Foxp1 |
| B | Foxp2 |
| B | Frat1 |
| B | Fxyd1 |
| B | Fyco1 |
| B | Fzd2 |
| B | Fzd3 |
| B | Fzd7 |
| B | Gabarapl1 |
| B | Garnl1 |
| B | Gcap14 |
| B | Gcat |
| B | Gcc2 |
| B | Gcc2 |
| B | Gdpd1 |
| B | Ggcx |
| B | Git2 |
| B | Gkap1 |
| B | Glb1l |
| B | Glcci1 |
| B | Glcci1 |
| B | Glt28d2 |
| B | Glt8d3 |
| B | Gm114 |
| B | Gng11 |
| B | Gnmt |
| B | Gnpat |
| B | Gpc4 |
| B | Gpd1l |
| B | Gpm6a |
| B | Gpr125 |
| B | Gpr126 |
| B | Gpr155 |
| B | Gpr175 |
| B | Gpr30 |
| B | Gprasp1 |
| B | Gpsn2 |
| B | Gramd1c |
| B | Grcc10 |
| B | Grem2 |
| B | Grtp1 |
| B | Gstp1 |
| B | Gstt2 |
| B | Gstt3 |
| B | Gtf2i |
| B | Gtf3c1 |
| B | Gucy1a3 |
| B | Gucy1b3 |
| B | Gucy1b3 |
| B | Gulp1 |
| B | Hbp1 |
| B | Hcfc1r1 |
| B | Hdac5 |
| B | Hdac7a |
| B | Hdac8 |
| B | Heca |
| B | Heca |
| B | Helz |
| B | Helz |
| B | Herpud1 |
| B | Hey1 |
| B | Hfe |
| B | Higd1b |
| B | Hipk2 |
| B | Hipk3 |
| B | Hisppd2a |
| B | Hkdc1 |
| B | Hlcs |
| B | Hltf |
| B | Hmbox1 |
| B | Hmcn1 |
| B | Hmg20a |
| B | Hnrph3 |
| B | Hp1bp3 |
| B | Hrsp12 |
| B | Hs2st1 |
| B | Hsd11b1 |
| B | Hsd17b4 |
| B | Ica1 |
| B | Ica1 |
| B | Icam2 |
| B | Id4 |
| B | Ift20 |
| B | Ift20 |
| B | Igf1r |
| B | Il11ra1 |
| B | Il11ra1 |
| B | Il17re |
| B | Impdh1 |
| B | Ing3 |
| B | Inpp5a |
| B | Iqsec1 |
| B | Iqsec1 |
| B | Irs1 |
| B | Itfg1 |
| B | Itfg1 |
| B | Itga1 |
| B | Itga8 |
| B | Itga8 |
| B | Itm2b |
| B | Itm2b |
| B | Itpr1 |
| B | Itsn1 |
| B | Ivd |
| B | Jak1 |
| B | Kcna2 |
| B | Kdelc2 |
| B | Kdelc2 |
| B | Kdr |
| B | Kif13a |
| B | Kif16b |
| B | Kif1c |
| B | Kif26a |
| B | Kif26b |
| B | Kifap3 |
| B | Kitl |
| B | Klf9 |
| B | Klf9 |
| B | Klhdc8b |
| B | Klhl13 |
| B | Klhl17 |
| B | Klhl24 |
| B | Klhl7 |
| B | Klhl8 |
| B | Klk8 |
| B | Lama3 |
| B | Lama4 |
| B | Lamb2 |
| B | Lamp3 |
| B | Lamp3 |
| B | Lamp3 |
| B | Lbx2 |
| B | Lims2 |
| B | Lmbr1 |
| B | LOC636537 |
| B | Lonp2 |
| B | Lphn1 |
| B | Lpin1 |
| B | Lrba |
| B | Lrp2 |
| B | Lrp2 |
| B | Lrp5 |
| B | Lrrfip2 |
| B | Lrrk2 |
| B | Ltbp3 |
| B | Ly6g6d |
| B | Lycat |
| B | Lyrm4 |
| B | Lztr1 |
| B | Macf1 |
| B | Mageh1 |
| B | Magi1 |
| B | Magi3 |
| B | Mamdc2 |
| B | Maml2 |
| B | Map1lc3a |
| B | Map1lc3b |
| B | Map3k4 |
| B | Map4k2 |
| B | Mapk12 |
| B | Mapk1ip1 |
| B | Mapkbp1 |
| B | Mapre2 |
| B | Mapt |
| B | Mapt |
| B | Mbnl2 |
| B | Mbtps1 |
| B | Mbtps1 |
| B | Mcee |
| B | Mcf2l |
| B | Med25 |
| B | Megf9 |
| B | Mett5d1 |
| B | Mgll |
| B | Mgst1 |
| B | Mgst1 |
| B | Mib1 |
| B | Mid1ip1 |
| B | Mid2 |
| B | Mir16 |
| B | Mlc1 |
| B | Mllt6 |
| B | Mmaa |
| B | Mmd |
| B | Mme |
| B | Mmp11 |
| B | Mmp28 |
| B | Mobkl2b |
| B | Mospd2 |
| B | Mphosph9 |
| B | Mpp5 |
| B | Mpp7 |
| B | Mpped2 |
| B | Mpv17 |
| B | Mpv17l |
| B | Mrpl14 |
| B | Mtac2d1 |
| B | Mtap4 |
| B | Mtfr1 |
| B | Mtss1 |
| B | Mtus1 |
| B | Mtx3 |
| B | Muc1 |
| B | Mum1 |
| B | Mxd4 |
| B | Mxi1 |
| B | Mxra8 |
| B | Myh10 |
| B | Myh7 |
| B | Mylk |
| B | Mylk |
| B | Myo1b |
| B | Myo1b |
| B | Myo1d |
| B | Myo6 |
| B | Myo9a |
| B | Myst4 |
| B | NAP033385-1 |
| B | NAP101459-1 |
| B | NAP122883-1 |
| B | NAP123201-1 |
| B | Nap1l1 |
| B | Nap1l3 |
| B | Napsa |
| B | Nbeal1 |
| B | Nbeal1 |
| B | Nck1 |
| B | Ndst1 |
| B | Ndst1 |
| B | Ndufb2 |
| B | Nebl |
| B | Nebl |
| B | Nek1 |
| B | Nelf |
| B | Neo1 |
| B | Nfat5 |
| B | Nfia |
| B | Nfia |
| B | Nfib |
| B | Nfib |
| B | Nfib |
| B | Nhlrc2 |
| B | Nipa1 |
| B | Nipbl |
| B | Nisch |
| B | Nisch |
| B | Nme3 |
| B | Notch4 |
| B | Nox4 |
| B | Npnt |
| B | Npnt |
| B | Npr3 |
| B | Npr3 |
| B | Nr3c1 |
| B | Nrbp2 |
| B | Nrp1 |
| B | Nuak1 |
| B | Nucb1 |
| B | Numa1 |
| B | Oaz2 |
| B | Oaz2 |
| B | Ociad2 |
| B | Ogn |
| B | Ogt |
| B | Omd |
| B | Osbpl2 |
| B | Osbpl5 |
| B | OTTMUSG00000003947 |
| B | Otud1 |
| B | Oxct1 |
| B | Oxr1 |
| B | Paip2 |
| B | Palmd |
| B | Paqr4 |
| B | Pard3 |
| B | Park2 |
| B | Parva |
| B | Patz1 |
| B | Pbx3 |
| B | Pcdh12 |
| B | Pcdha6 |
| B | Pcdha9 |
| B | Pcdha9 |
| B | Pcdhga7 |
| B | Pcmtd2 |
| B | Pcolce2 |
| B | Pcsk6 |
| B | Pcx |
| B | Pcyox1 |
| B | Pcyt2 |
| B | Pdcd4 |
| B | Pdcd6ip |
| B | Pde4d |
| B | Pde8b |
| B | Pdgfd |
| B | Pdlim2 |
| B | Pea15a |
| B | Pex26 |
| B | Pftk1 |
| B | Pgm2l1 |
| B | Phactr1 |
| B | Phactr1 |
| B | Phf17 |
| B | Phkb |
| B | Pias2 |
| B | Pigp |
| B | Pik3ca |
| B | Pik3ca |
| B | Pink1 |
| B | Pir |
| B | Pitpnc1 |
| B | Pitpnc1 |
| B | Pja1 |
| B | Pkia |
| B | Pkp4 |
| B | Pkp4 |
| B | Plagl1 |
| B | Plcg1 |
| B | Plekha6 |
| B | Plekhb1 |
| B | Plekhc1 |
| B | Pltp |
| B | Plxdc2 |
| B | Plxna1 |
| B | Pmp22 |
| B | Pon1 |
| B | Por |
| B | Postn |
| B | Ppfibp2 |
| B | Ppfibp2 |
| B | Ppid |
| B | Ppm1b |
| B | Ppm1l |
| B | Ppp1cb |
| B | Ppp1r12b |
| B | pPtp4a3 |
| B | Prelp |
| B | Prickle1 |
| B | Prickle1 |
| B | Prickle2 |
| B | Prkaa2 |
| B | Prkce |
| B | Prkcn |
| B | Prkg1 |
| B | Prr8 |
| B | Prss36 |
| B | Pscd3 |
| B | Psd3 |
| B | Psd3 |
| B | Ptch1 |
| B | Pten |
| B | Ptgfr |
| B | Ptk2 |
| B | Ptov1 |
| B | Ptplad1 |
| B | Ptpn13 |
| B | Ptpn14 |
| B | Ptprd |
| B | Ptprd |
| B | Ptprf |
| B | Ptprf |
| B | Ptprf |
| B | Ptprm |
| B | Ptprr |
| B | Ptprs |
| B | Ptrf |
| B | Ptrf |
| B | Pts |
| B | Purg |
| B | Pvrl3 |
| B | Pvrl3 |
| B | Pxmp2 |
| B | Pxmp4 |
| B | Pxmp4 |
| B | Qser1 |
| B | Rab11fip3 |
| B | Rab2b |
| B | Rab3d |
| B | Rabgap1l |
| B | Rabgap1l |
| B | Rad51l3 |
| B | Ramp2 |
| B | Rap1gap |
| B | Rapgef4 |
| B | Rasgef1a |
| B | Rasgrp2 |
| B | Rasl12 |
| B | Rb1cc1 |
| B | Rbbp6 |
| B | Rbl2 |
| B | Rbms3 |
| B | Rbms3 |
| B | Rbmx |
| B | Rbp1 |
| B | Reck |
| B | Rere |
| B | Rftn2 |
| B | Rgs3 |
| B | Rhebl1 |
| B | Ripk5 |
| B | Rmnd5a |
| B | Rmnd5a |
| B | Rnase4 |
| B | Rnase4 |
| B | Rnf141 |
| B | Rnf141 |
| B | Rnf144 |
| B | Rnf43 |
| B | Rnf5 |
| B | Robo2 |
| B | Rora |
| B | Rora |
| B | Rora |
| B | RP23-136K12.4 |
| B | RP23-157O10.7 |
| B | RP23-195K8.6 |
| B | Rpl22 |
| B | Rsu1 |
| B | Rtn1 |
| B | Rtn1 |
| B | Rtn3 |
| B | Rtn4rl1 |
| B | Rusc2 |
| B | S100a1 |
| B | Sash1 |
| B | Scamp1 |
| B | Scarf2 |
| B | Scn7a |
| B | Scnn1b |
| B | Scrn3 |
| B | Scrn3 |
| B | Scube2 |
| B | Scx |
| B | Sdc2 |
| B | Sdk2 |
| B | Sdpr |
| B | Sec14l1 |
| B | Sec14l2 |
| B | Sec14l3 |
| B | Sec14l3 |
| B | Sec14l3 |
| B | Sec63 |
| B | Sel1l |
| B | Sema3b |
| B | Sema3g |
| B | Sema6a |
| B | Serinc5 |
| B | Sesn1 |
| B | Sestd1 |
| B | Setd3 |
| B | Setd7 |
| B | Setd7 |
| B | Sft2d2 |
| B | Sftpa1 |
| B | Sftpa1 |
| B | Sftpb |
| B | Sgce |
| B | Sgsh |
| B | Sh3bgrl |
| B | Sh3md4 |
| B | Sh3rf1 |
| B | Sh3rf1 |
| B | Sh3tc2 |
| B | Shroom1 |
| B | Sigirr |
| B | Siglecf |
| B | Sipa1l2 |
| B | Six5 |
| B | Slain2 |
| B | Slc12a2 |
| B | Slc12a5 |
| B | Slc13a4 |
| B | Slc16a2 |
| B | Slc16a9 |
| B | Slc1a4 |
| B | Slc24a3 |
| B | Slc25a35 |
| B | Slc25a36 |
| B | Slc25a36 |
| B | Slc29a1 |
| B | Slc2a13 |
| B | Slc30a9 |
| B | Slc34a2 |
| B | Slc34a2 |
| B | Slc38a5 |
| B | Slc7a4 |
| B | Slc9a3r2 |
| B | Slc9a6 |
| B | Slit2 |
| B | Slitrk6 |
| B | Smarca2 |
| B | Smarce1 |
| B | Smurf2 |
| B | Smurf2 |
| B | Snai2 |
| B | Sned1 |
| B | Snrpn |
| B | Snx13 |
| B | Snx21 |
| B | Snx25 |
| B | Sod1 |
| B | Sord |
| B | Sort1 |
| B | Sort1 |
| B | Sort1 |
| B | Sox13 |
| B | Sox18 |
| B | Specc1l |
| B | Spg20 |
| B | Spna2 |
| B | Spnb2 |
| B | Spnb2 |
| B | Spock2 |
| B | Spock2 |
| B | Spry1 |
| B | Sstr4 |
| B | St5 |
| B | St6galnac6 |
| B | Stard9 |
| B | Stard9 |
| B | Stard9 |
| B | Stk36 |
| B | Stmn2 |
| B | Stx17 |
| B | Suhw4 |
| B | Syne1 |
| B | Syne2 |
| B | Synj2 |
| B | Sytl4 |
| B | Tanc1 |
| B | Tbc1d19 |
| B | Tbc1d8b |
| B | Tbcel |
| B | Tbx3 |
| B | Tbx6 |
| B | TC1605426 |
| B | TC1672661 |
| B | Tcp11l2 |
| B | Tcp11l2 |
| B | Tek |
| B | Tenc1 |
| B | Tfdp2 |
| B | Tgfb3 |
| B | Thbd |
| B | Thbs3 |
| B | Thrb |
| B | Thsd4 |
| B | Tie1 |
| B | Timp2 |
| B | Tinag |
| B | Tjp1 |
| B | Tlcd1 |
| B | Tle2 |
| B | Tle6 |
| B | Tm2d2 |
| B | Tmcc3 |
| B | Tmco1 |
| B | Tmco3 |
| B | Tmeff1 |
| B | Tmem100 |
| B | Tmem106b |
| B | Tmem109 |
| B | Tmem110 |
| B | Tmem117 |
| B | Tmem123 |
| B | Tmem14a |
| B | Tmem150 |
| B | Tmem16a |
| B | Tmem18 |
| B | Tmem24 |
| B | Tmem32 |
| B | Tmem4 |
| B | Tmem41a |
| B | Tmem42 |
| B | Tmem50b |
| B | Tmem59 |
| B | Tmem63b |
| B | Tmem64 |
| B | Tmem64 |
| B | Tmem98 |
| B | Tmod1 |
| B | Tnfrsf19 |
| B | Tns1 |
| B | Tns3 |
| B | Tom1l2 |
| B | Tpcn1 |
| B | Trim2 |
| B | Trim3 |
| B | Trim3 |
| B | Trim32 |
| B | Trim35 |
| B | Trim44 |
| B | Trp53bp2 |
| B | Trp53inp2 |
| B | Tsc22d1 |
| B | Tspan12 |
| B | Tspan13 |
| B | Tspan15 |
| B | Tspan18 |
| B | Tspan2 |
| B | Tspan7 |
| B | Tspan9 |
| B | Ttbk2 |
| B | Ttc23 |
| B | Ttc28 |
| B | Ttc28 |
| B | Ttc3 |
| B | Twsg1 |
| B | Ube2h |
| B | Ubqln1 |
| B | Ubr1 |
| B | Ubr1 |
| B | Ubr2 |
| B | Ulk1 |
| B | Ulk2 |
| B | Unc84a |
| B | Usp33 |
| B | Usp33 |
| B | Usp46 |
| B | Vamp4 |
| B | Vamp5 |
| B | Vcl |
| B | Vegfa |
| B | Vegfa |
| B | Vgll4 |
| B | Vkorc1 |
| B | Vldlr |
| B | Vps13a |
| B | Vps13b |
| B | Vps13d |
| B | Vtn |
| B | Vwf |
| B | Wbp1 |
| B | Wdfy3 |
| B | Wdr42a |
| B | Wdr45 |
| B | Wdr47 |
| B | Wdr6 |
| B | Wnt3a |
| B | Wrb |
| B | Wscd1 |
| B | Wwp1 |
| B | Wwp1 |
| B | Wwtr1 |
| B | Wwtr1 |
| B | Xpc |
| B | Xpc |
| B | Xpr1 |
| B | Yap1 |
| B | Ypel1 |
| B | Ypel3 |
| B | Yy1 |
| B | Zbtb10 |
| B | Zbtb20 |
| B | Zbtb20 |
| B | Zbtb4 |
| B | Zc3h7b |
| B | Zeb1 |
| B | Zfp101 |
| B | Zfp106 |
| B | Zfp2 |
| B | Zfp219 |
| B | Zfp219 |
| B | Zfp277 |
| B | Zfp354a |
| B | Zfp422-rs1 |
| B | Zfp579 |
| B | Zfp592 |
| B | Zfp612 |
| B | Zfp637 |
| B | Zfp664 |
| B | Zfp704 |
| B | Zfp740 |
| B | Zfyve21 |
| B | Zfyve27 |
| B | Zhx1 |
| B | Zkscan14 |
| B | Zmat3 |
| B | Zmym2 |
| B | Zmym3 |
| B | Zrsr1 |
| C | 4-Mar |
| C | 0610011F06Rik |
| C | 0610040J01Rik |
| C | 1110001A16Rik |
| C | 1110004E09Rik |
| C | 1110013L07Rik |
| C | 1110017D15Rik |
| C | 1110032A03Rik |
| C | 1190002A17Rik |
| C | 1190002J23Rik |
| C | 1190007F08Rik |
| C | 1190007I07Rik |
| C | 1500011H22Rik |
| C | 1500015O10Rik |
| C | 1600021P15Rik |
| C | 1600029D21Rik |
| C | 1700001L19Rik |
| C | 1700003M02Rik |
| C | 1700009P17Rik |
| C | 1700010A17Rik |
| C | 1700016K19Rik |
| C | 1700020I14Rik |
| C | 1700021K14Rik |
| C | 1700025K23Rik |
| C | 1700026D08Rik |
| C | 1700026L06Rik |
| C | 1700027N10Rik |
| C | 1700030J22Rik |
| C | 1700088E04Rik |
| C | 1700094D03Rik |
| C | 1700123D08Rik |
| C | 1810007P19Rik |
| C | 1810008I18Rik |
| C | 1810019J16Rik |
| C | 1810048J11Rik |
| C | 2010007H06Rik |
| C | 2010300C02Rik |
| C | 2310005E10Rik |
| C | 2310007A19Rik |
| C | 2310016C08Rik |
| C | 2310016C08Rik |
| C | 2310030G06Rik |
| C | 2310045A20Rik |
| C | 2310057J16Rik |
| C | 2410187C16Rik |
| C | 2510009E07Rik |
| C | 2610015P09Rik |
| C | 2610020H08Rik |
| C | 2610028H24Rik |
| C | 2610110G12Rik |
| C | 2700094K13Rik |
| C | 2810002I04Rik |
| C | 2810002I04Rik |
| C | 2810008D09Rik |
| C | 2810410M20Rik |
| C | 2810453I06Rik |
| C | 2900006F19Rik |
| C | 2900042B11Rik |
| C | 2900046G09Rik |
| C | 3010026O09Rik |
| C | 3110003A22Rik |
| C | 3110009E18Rik |
| C | 3110050N22Rik |
| C | 3300002A11Rik |
| C | 4632417N05Rik |
| C | 4732415M23Rik |
| C | 4732474O15Rik |
| C | 4833401D15Rik |
| C | 4833436C18Rik |
| C | 4921509J17Rik |
| C | 4921513D23Rik |
| C | 4921537I17Rik |
| C | 4930430E16Rik |
| C | 4930430E16Rik |
| C | 4930431B11Rik |
| C | 4930444P10Rik |
| C | 4930455F23Rik |
| C | 4930535E21Rik |
| C | 4930562C15Rik |
| C | 4930588N13Rik |
| C | 4931408A02Rik |
| C | 4931408A02Rik |
| C | 4932425I24Rik |
| C | 4933404M02Rik |
| C | 4933404M02Rik |
| C | 4933430H15Rik |
| C | 4933439F18Rik |
| C | 5330417C22Rik |
| C | 5330431N19Rik |
| C | 5430414B12Rik |
| C | 5730494M16Rik |
| C | 5830404H04Rik |
| C | 6330403L08Rik |
| C | 6330439K17Rik |
| C | 6330505N24Rik |
| C | 6430537H07Rik |
| C | 6530401C20Rik |
| C | 6720401G13Rik |
| C | 6720473M08Rik |
| C | 6820408C15Rik |
| C | 9030612M13Rik |
| C | 9330101J02Rik |
| C | 9630019K15Rik |
| C | 9630023C09Rik |
| C | 9830169C18Rik |
| C | AA536717 |
| C | Abcc5 |
| C | Abcf3 |
| C | Acaa1b |
| C | Acad10 |
| C | Acsl3 |
| C | Acsl4 |
| C | Acy3 |
| C | Adam19 |
| C | Aga |
| C | Agbl2 |
| C | Ahctf1 |
| C | AI427122 |
| C | Aim1l |
| C | Air |
| C | AK030494 |
| C | AK033818 |
| C | AK036787 |
| C | AK044272 |
| C | AK047347 |
| C | AK047616 |
| C | AK048657 |
| C | AK051988 |
| C | AK053596 |
| C | AK075660 |
| C | AK076876 |
| C | AK085706 |
| C | Ak7 |
| C | Akap14 |
| C | Akr1b3 |
| C | Alad |
| C | Aldh1a7 |
| C | Aldh3b1 |
| C | Alg14 |
| C | Alox5 |
| C | Angptl6 |
| C | Ankrd26 |
| C | Ankrd42 |
| C | Ankrd44 |
| C | Ankrd5 |
| C | Ankrd5 |
| C | Ap1m2 |
| C | Apcdd1 |
| C | Apln |
| C | Apln |
| C | Apoa1bp |
| C | Arhgap18 |
| C | Arhgdig |
| C | Arhgef4 |
| C | Aspa |
| C | Atg4b |
| C | Atp2c2 |
| C | Atxn2 |
| C | AU020772 |
| C | AV249152 |
| C | AV249152 |
| C | Azi1 |
| C | B230120H23Rik |
| C | B230208H17Rik |
| C | B3gnt4 |
| C | Banp |
| C | Bbs5 |
| C | Bbs5 |
| C | BC004853 |
| C | BC006662 |
| C | BC007180 |
| C | BC013529 |
| C | BC019943 |
| C | BC020535 |
| C | BC038167 |
| C | BC051019 |
| C | BC060267 |
| C | BC062650 |
| C | BC064078 |
| C | Bcas1 |
| C | BF642829 |
| C | Bmp5 |
| C | Bmpr2 |
| C | Bphl |
| C | Bzrap1 |
| C | C230082I21Rik |
| C | C230096C10Rik |
| C | C730043O17 |
| C | C77370 |
| C | C77370 |
| C | Cacybp |
| C | Calml4 |
| C | Camk1 |
| C | Camk1 |
| C | Camkk1 |
| C | Capsl |
| C | Car11 |
| C | Cbr3 |
| C | Ccdc108 |
| C | Ccdc13 |
| C | Ccdc14 |
| C | Ccdc34 |
| C | Ccdc40 |
| C | Ccdc46 |
| C | Ccdc52 |
| C | Ccdc67 |
| C | Ccdc85a |
| C | Ccdc85a |
| C | Ccdc92 |
| C | Ccnt2 |
| C | Cd24a |
| C | Cd59b |
| C | Cd99l2 |
| C | Cdc14a |
| C | Cdc14a |
| C | Cds1 |
| C | Cds2 |
| C | Celsr1 |
| C | Centg3 |
| C | Cep164 |
| C | Cep70 |
| C | Cetn2 |
| C | Cetn2 |
| C | Chchd6 |
| C | Chordc1 |
| C | Chordc1 |
| C | Cldn3 |
| C | Cldn7 |
| C | Clmn |
| C | Cnn1 |
| C | Cnnm2 |
| C | Col16a1 |
| C | Col8a2 |
| C | Cpt1c |
| C | Crip3 |
| C | Csnk1g2 |
| C | Csnk1g2 |
| C | Cspp1 |
| C | Ctxn1 |
| C | Cxx1b |
| C | Cyp2a4 |
| C | Cyp2a5 |
| C | Cyp2s1 |
| C | Cyp3a11 |
| C | D030011O10Rik |
| C | D0H4S114 |
| C | D0H4S114 |
| C | D10Bwg1070e |
| C | D130043K22Rik |
| C | D16Bwg1494e |
| C | D19Ertd652e |
| C | D230014K01Rik |
| C | D330028D13Rik |
| C | D330050I23Rik |
| C | D430042O09Rik |
| C | D630039A03Rik |
| C | Dact2 |
| C | Dalrd3 |
| C | Dalrd3 |
| C | Dbndd1 |
| C | Dcp1b |
| C | Ddit4l |
| C | Dgkg |
| C | Dhx29 |
| C | Dleu2 |
| C | Dmrt2 |
| C | Dnahc11 |
| C | Dnahc2 |
| C | Dnahc6 |
| C | Dnahc9 |
| C | Dnaja1 |
| C | Dnaja1 |
| C | Dnaja1 |
| C | Dnaja4 |
| C | Dnajb1 |
| C | Dnajb1 |
| C | Dnajc12 |
| C | Dnalc4 |
| C | Dock8 |
| C | Dpt |
| C | Dusp14 |
| C | Dusp14 |
| C | Dync2h1 |
| C | Dync2h1 |
| C | Dynlrb2 |
| C | Dynlrb2 |
| C | Dynlt3 |
| C | Dyx1c1 |
| C | Dzip1 |
| C | E030013G06Rik |
| C | E130303B06Rik |
| C | E130319B15Rik |
| C | E230008N13Rik |
| C | Ebf1 |
| C | Ecgf1 |
| C | Efcab1 |
| C | Efhb |
| C | EG432995 |
| C | EG621324 |
| C | EG621431 |
| C | Endog |
| C | ENSMUSG00000050599 |
| C | ENSMUSG00000052188 |
| C | ENSMUST00000035915 |
| C | ENSMUST00000036576 |
| C | ENSMUST00000050829 |
| C | Eppb9 |
| C | Eps8l2 |
| C | Exoc4 |
| C | Exosc7 |
| C | Eya1 |
| C | F2r |
| C | F8a |
| C | Faah |
| C | Fat1 |
| C | Fbxo36 |
| C | Fhad1 |
| C | Fhod1 |
| C | Fnbp1 |
| C | Foxj1 |
| C | Fsip1 |
| C | G0s2 |
| C | Gadd45gip1 |
| C | Galnt3 |
| C | Gart |
| C | Gas1 |
| C | Gats |
| C | Ggt6 |
| C | Gja7 |
| C | Gja7 |
| C | Gm1060 |
| C | Gm166 |
| C | Gm166 |
| C | Gna11 |
| C | Gna14 |
| C | Gng13 |
| C | Golph2 |
| C | Gpr22 |
| C | Gprc5c |
| C | Gpx2 |
| C | Grb10 |
| C | Grhl2 |
| C | Grip1 |
| C | Gtl2 |
| C | Gtl2 |
| C | Gtl3 |
| C | Hes6 |
| C | Hgf |
| C | Hgfac |
| C | Hirip3 |
| C | Hist1h2ba |
| C | Hmgcll1 |
| C | Hmgn3 |
| C | Hmgn3 |
| C | Hoxb3 |
| C | Hoxb5 |
| C | Hpn |
| C | Hpn |
| C | Hsp110 |
| C | Hsp90ab1 |
| C | Hspa1a |
| C | Hspa1a |
| C | Hspa4l |
| C | Hspa4l |
| C | Hspa4l |
| C | Hspb1 |
| C | Ift140 |
| C | Ift80 |
| C | Ift81 |
| C | Ift81 |
| C | Ift88 |
| C | Ildr1 |
| C | Iqca |
| C | Iqcg |
| C | Irx2 |
| C | Kcnk3 |
| C | Kcnk3 |
| C | Kif1b |
| C | Kif21a |
| C | Klc3 |
| C | Klf4 |
| C | Klhdc7a |
| C | Kndc1 |
| C | Kremen1 |
| C | Krtcap3 |
| C | Krtcap3 |
| C | Ldhb |
| C | Lef1 |
| C | Lgtn |
| C | Liph |
| C | Lnx1 |
| C | Lnx1 |
| C | LOC384261 |
| C | Lphn2 |
| C | Lphn3 |
| C | Lpin2 |
| C | Lrba |
| C | Lrig1 |
| C | Lrrc23 |
| C | Lrrc51 |
| C | Lrrc51 |
| C | Lrrc56 |
| C | Lrrc58 |
| C | Lrrc8e |
| C | Lrsam1 |
| C | Lxn |
| C | Lynx1 |
| C | Magi2 |
| C | Mak |
| C | Mansc1 |
| C | Map3k12 |
| C | Mapk15 |
| C | Mbip |
| C | Mdh1b |
| C | Met |
| C | Mgat3 |
| C | Mgat5 |
| C | Mgst2 |
| C | Mib2 |
| C | Mipep |
| C | Mlf1 |
| C | Mmrn1 |
| C | Morn3 |
| C | Mospd2 |
| C | Mrc2 |
| C | Mrgprf |
| C | Mrps6 |
| C | Mrps6 |
| C | Mtap6 |
| C | Mtap6 |
| C | Mtap7 |
| C | Mtap7 |
| C | Mtss1 |
| C | Muc1 |
| C | Muc4 |
| C | Muc5ac |
| C | Mxd4 |
| C | Myb |
| C | Mycbp |
| C | Myh11 |
| C | Myl9 |
| C | NAP006594-001 |
| C | NAP027922-1 |
| C | NAP029500-1 |
| C | NAP123115-1 |
| C | Naprt1 |
| C | Nek1 |
| C | Neurl |
| C | Nfrkb |
| C | Ngef |
| C | Ngfrap1 |
| C | Nipsnap1 |
| C | Nkd1 |
| C | Nme5 |
| C | Nme5 |
| C | Nme7 |
| C | Nnt |
| C | Nphp3 |
| C | Nr2c2 |
| C | Nup210 |
| C | Olfml1 |
| C | Oplah |
| C | Osbpl10 |
| C | OTTMUSG00000003456 |
| C | Ovol2 |
| C | P4ha1 |
| C | Pamci |
| C | Pcdh18 |
| C | Pcp4l1 |
| C | Peli3 |
| C | Per2 |
| C | Pfn2 |
| C | Phlda2 |
| C | Pigz |
| C | Pih1d2 |
| C | Pitpnb |
| C | Pkd2 |
| C | Pkp2 |
| C | Plcb3 |
| C | Plch2 |
| C | Plekhg6 |
| C | Pmm1 |
| C | Polr3k |
| C | Pomt2 |
| C | Pon2 |
| C | Porcn |
| C | Ppp1r16a |
| C | Prdx6 |
| C | Prkcz |
| C | Ptgis |
| C | Rab15 |
| C | Rab25 |
| C | Rabl2a |
| C | Rage |
| C | Rage |
| C | Ralgps1 |
| C | Rasl11a |
| C | Rassf6 |
| C | Rassf7 |
| C | Rbm35a |
| C | Rbm35b |
| C | Rec8L1 |
| C | Reep6 |
| C | Rfk |
| C | Ribc1 |
| C | Ribc2 |
| C | Ripk4 |
| C | Rnf128 |
| C | Rnf186 |
| C | Rnf32 |
| C | Rnf32 |
| C | Ropn1l |
| C | Rpgr |
| C | Rps11 |
| C | Rragd |
| C | Sccpdh |
| C | Scrn2 |
| C | Sec31b |
| C | Serpina9 |
| C | Serpinb1a |
| C | Serpinf1 |
| C | Sertad4 |
| C | Sesn3 |
| C | Sft2d3 |
| C | Sh3glb2 |
| C | Shank3 |
| C | Siae |
| C | Siah2 |
| C | Six1 |
| C | Slc12a7 |
| C | Slc22a17 |
| C | Slc22a8 |
| C | Slc23a1 |
| C | Slc25a10 |
| C | Slc25a17 |
| C | Slc35d1 |
| C | Slc39a4 |
| C | Slc43a3 |
| C | Slc44a1 |
| C | Slc44a2 |
| C | Slc46a1 |
| C | Slc5a3 |
| C | Smarcd3 |
| C | Smo |
| C | Sobp |
| C | Sorbs3 |
| C | Sox17 |
| C | Sp5 |
| C | Spa17 |
| C | Spag16 |
| C | Spag17 |
| C | Spata1 |
| C | Spata7 |
| C | Spef1 |
| C | Spint1 |
| C | Spint1 |
| C | Ssbp3 |
| C | St8sia4 |
| C | St8sia4 |
| C | Stau2 |
| C | Stip1 |
| C | Stk36 |
| C | Stox2 |
| C | Strbp |
| C | Strbp |
| C | Stx19 |
| C | Stxbp5 |
| C | Sult5a1 |
| C | Swap70 |
| C | Syn2 |
| C | Tagln |
| C | Tagln |
| C | Taok2 |
| C | TC1610785 |
| C | TC1620718 |
| C | TC1643662 |
| C | TC1671899 |
| C | TC1676032 |
| C | TC1682954 |
| C | TC1690652 |
| C | TC1697186 |
| C | Tcea3 |
| C | Tcf2 |
| C | Tekt1 |
| C | Tekt2 |
| C | Tekt4 |
| C | Tjp3 |
| C | Tlcd1 |
| C | Tle1 |
| C | Tmc4 |
| C | Tmc4 |
| C | Tmc5 |
| C | Tmcc3 |
| C | Tmem107 |
| C | Tmem16k |
| C | Tmem29 |
| C | Tmem54 |
| C | Tmem77 |
| C | Tmtc2 |
| C | Tnk1 |
| C | Tom1l1 |
| C | Tpm2 |
| C | Traf3ip1 |
| C | Traf3ip1 |
| C | Trmt12 |
| C | Tro |
| C | Tro |
| C | Trp73 |
| C | Tspan33 |
| C | Tspyl4 |
| C | Ttc12 |
| C | Ttc13 |
| C | Ttc18 |
| C | Ttc18 |
| C | Ttc21b |
| C | Ttc30a1 |
| C | Ttc7b |
| C | Tyro3 |
| C | Tyro3 |
| C | Ube2b |
| C | Ubfd1 |
| C | Ubr1 |
| C | Ubxd5 |
| C | Upk3b |
| C | Usp54 |
| C | Vapb |
| C | Vars2 |
| C | Vpreb3 |
| C | Wbp5 |
| C | Wdr35 |
| C | Wdr35 |
| C | Wdr52 |
| C | Wdr54 |
| C | Wdr60 |
| C | Wdr63 |
| C | Wdr67 |
| C | Wdr78 |
| C | Wdr90 |
| C | Whrn |
| C | Wnk4 |
| C | X12807 |
| C | Xpot |
| C | Xrcc4 |
| C | Yipf2 |
| C | Yipf2 |
| C | Yipf2 |
| C | Yme1l1 |
| C | Zdhhc2 |
| C | Zdhhc2 |
| C | Zfp398 |
| C | Zfp40 |
| C | Zfp422 |
| C | Zfp474 |
| C | Zfp61 |
| C | Zfp750 |
| C | Zmynd10 |
| C | Zmynd12 |
| C | Zswim3 |
| C | Zxda |
| D | 7-Mar |
| D | 7-Mar |
| D | 10-Sep |
| D | 0610007P08Rik |
| D | 1110033M05Rik |
| D | 1110037F02Rik |
| D | 1700001J11Rik |
| D | 1700081L11Rik |
| D | 1700124K17Rik |
| D | 2210417D09Rik |
| D | 2310003C23Rik |
| D | 2610024E20Rik |
| D | 2610028A01Rik |
| D | 2610101N10Rik |
| D | 2610101N10Rik |
| D | 2610207I05Rik |
| D | 2610507B11Rik |
| D | 2610507B11Rik |
| D | 2810006K23Rik |
| D | 2900053A13Rik |
| D | 2900083I11Rik |
| D | 3110031B13Rik |
| D | 4121402D02Rik |
| D | 4921505C17Rik |
| D | 4921524J17Rik |
| D | 4930402H24Rik |
| D | 4930422G04Rik |
| D | 4930422G04Rik |
| D | 4930506M07Rik |
| D | 4930535B03Rik |
| D | 4930539N22Rik |
| D | 4932438A13Rik |
| D | 5830415L20Rik |
| D | 9030416H16Rik |
| D | A_52_P860487 |
| D | A430041B07Rik |
| D | A430041B07Rik |
| D | A630047E20Rik |
| D | A830080L01Rik |
| D | AA536743 |
| D | AA987161 |
| D | Abcc1 |
| D | Abce1 |
| D | Acbd3 |
| D | Acin1 |
| D | Actb |
| D | Adam17 |
| D | AF315352 |
| D | Agpat6 |
| D | AI504432 |
| D | AK032729 |
| D | AK036484 |
| D | AK040891 |
| D | AK042230 |
| D | AK045163 |
| D | AK046154 |
| D | AK047888 |
| D | AK050569 |
| D | AK051319 |
| D | AK051522 |
| D | AK080115 |
| D | AK081910 |
| D | AK084172 |
| D | AK087406 |
| D | AK087421 |
| D | AK088646 |
| D | Akap10 |
| D | Akap13 |
| D | Akt1 |
| D | Arhgap10 |
| D | Arhgap26 |
| D | Arhgef6 |
| D | Arl6ip2 |
| D | Atad2b |
| D | Atf7ip |
| D | Atg12 |
| D | Atp11c |
| D | Atp2b1 |
| D | Atp6v0a1 |
| D | AU017455 |
| D | AU040320 |
| D | AY036118 |
| D | B230325K18Rik |
| D | Baz1b |
| D | Baz2a |
| D | Bbx |
| D | Bbx |
| D | BC005537 |
| D | BC082312 |
| D | BC082312 |
| D | Bclaf1 |
| D | Bhlhb8 |
| D | Braf |
| D | Brd4 |
| D | Brwd1 |
| D | C030048B08Rik |
| D | C030048B08Rik |
| D | C130022K22Rik |
| D | C130022K22Rik |
| D | C130039O16Rik |
| D | C230029D21Rik |
| D | C230081A13Rik |
| D | C430003P19Rik |
| D | C730024G19Rik |
| D | C80913 |
| D | C920006C10Rik |
| D | Cabin1 |
| D | Camkk2 |
| D | Canx |
| D | Cast |
| D | Cbll1 |
| D | Ccdc100 |
| D | Ccdc131 |
| D | Ccnk |
| D | Cd164 |
| D | Cd2ap |
| D | Cd47 |
| D | Cdc27 |
| D | Cdc2l5 |
| D | Cdc37l1 |
| D | Cdk5rap2 |
| D | Cebpg |
| D | Cebpz |
| D | Centb2 |
| D | Centb2 |
| D | Cep250 |
| D | Chd6 |
| D | Chd9 |
| D | Chuk |
| D | Clasp1 |
| D | Cldnd1 |
| D | Clip1 |
| D | Clip4 |
| D | Cmtm1 |
| D | Cnot3 |
| D | Col5a1 |
| D | Cpeb4 |
| D | Crebzf |
| D | Crkrs |
| D | Csde1 |
| D | Cyld |
| D | D030022P06Rik |
| D | D030074E01Rik |
| D | D14Ertd436e |
| D | D1Pas1 |
| D | D330037H05Rik |
| D | D5Wsu178e |
| D | Dars |
| D | Ddx1 |
| D | Ddx10 |
| D | Ddx23 |
| D | Ddx24 |
| D | Ddx25 |
| D | Ddx3x |
| D | Ddx42 |
| D | Ddx46 |
| D | Dek |
| D | Dgcr8 |
| D | Dis3 |
| D | Dnajc10 |
| D | Dnajc5 |
| D | Dnmt3a |
| D | Dph4 |
| D | Dst |
| D | Dzip1l |
| D | E030024N20Rik |
| D | E130014J05Rik |
| D | E130102H24Rik |
| D | EG382421 |
| D | EG385454 |
| D | Eif3s6 |
| D | Eif3s8 |
| D | Eif3s8 |
| D | Eif4a2 |
| D | Eif4b |
| D | Eif4e3 |
| D | Eif4g2 |
| D | Eif5b |
| D | Ell |
| D | Ensa |
| D | ENSMUST00000015595 |
| D | ENSMUST00000055843 |
| D | ENSMUST00000078779 |
| D | ENSMUST00000088251 |
| D | Ep400 |
| D | Epm2aip1 |
| D | Epn1 |
| D | Erbb2ip |
| D | Erlin2 |
| D | Etnk1 |
| D | Exoc6b |
| D | Fat1 |
| D | Fbxw2 |
| D | Fhl3 |
| D | Fmr1 |
| D | Fnip1 |
| D | Frmd4b |
| D | Fus |
| D | Fyco1 |
| D | Gabbr2 |
| D | Ganab |
| D | Gata4 |
| D | Gbf1 |
| D | Gls |
| D | Golgb1 |
| D | Gpatch2 |
| D | Gzf1 |
| D | H2afz |
| D | Hbs1l |
| D | Hel308 |
| D | Herc1 |
| D | Herc4 |
| D | Hira |
| D | Hist1h1e |
| D | Hist1h1t |
| D | Hlx1 |
| D | Hmg20b |
| D | Hmgb1-rs17 |
| D | Hnrpul2 |
| D | Hook3 |
| D | Hs1bp3 |
| D | Hspa4 |
| D | Htatsf1 |
| D | Huwe1 |
| D | Huwe1 |
| D | Ifnz |
| D | Igfbp4 |
| D | Ilf3 |
| D | Ing5 |
| D | Inhbb |
| D | Inoc1 |
| D | Inpp5f |
| D | Ints6 |
| D | Itgb1 |
| D | Jarid2 |
| D | Kctd11 |
| D | Kctd17 |
| D | Kdelc1 |
| D | Khdrbs1 |
| D | Kif2a |
| D | Klf3 |
| D | Klf6 |
| D | Klhl9 |
| D | Krtap6-3 |
| D | L3mbtl2 |
| D | Larp5 |
| D | Lars |
| D | Las1l |
| D | Lemd3 |
| D | Leng8 |
| D | Lifr |
| D | Lims1 |
| D | Lmbrd2 |
| D | LOC236598 |
| D | Lpp |
| D | Lrrc29 |
| D | Lrrc8b |
| D | Luzp1 |
| D | Maml2 |
| D | Marcks |
| D | Mbtps1 |
| D | Mdm4 |
| D | Mdn1 |
| D | Mef2c |
| D | Memo1 |
| D | Mgea6 |
| D | Mier1 |
| D | Mier1 |
| D | Mitf |
| D | Mkl2 |
| D | Mll2 |
| D | Mll3 |
| D | Mll3 |
| D | Mnt |
| D | Mobkl1a |
| D | Morf4l1 |
| D | Msn |
| D | Mtap4 |
| D | Mtif2 |
| D | Mtr |
| D | Myadm |
| D | Myo9a |
| D | Myo9b |
| D | Mysm1 |
| D | Myst3 |
| D | NAP028427-1 |
| D | NAP042178-1 |
| D | NAP052136-1 |
| D | NAP061200-1 |
| D | NAP102845-1 |
| D | NAP107478-1 |
| D | NAP112463-1 |
| D | NAP123498-1 |
| D | Nap1l4 |
| D | Ncoa6 |
| D | Nedd1 |
| D | Nfat5 |
| D | Nfat5 |
| D | Nfatc4 |
| D | Nfic |
| D | Nol8 |
| D | Npat |
| D | Nr1i3 |
| D | Nr2f2 |
| D | Nr3c1 |
| D | Nrg1 |
| D | Nsbp1 |
| D | Nsmaf |
| D | Nufip2 |
| D | Nup153 |
| D | Nup50 |
| D | Olfr32 |
| D | Osbpl1a |
| D | Otud7b |
| D | Pabpc2 |
| D | Paip2b |
| D | Papd5 |
| D | Pard6b |
| D | Pcdhga7 |
| D | Pcyt1a |
| D | Pdcd11 |
| D | Pdcl |
| D | Pde2a |
| D | Pdpk1 |
| D | Pdpk1 |
| D | Pdxk |
| D | Phf3 |
| D | Phip |
| D | Phip |
| D | Phldb2 |
| D | Piga |
| D | Pik3cb |
| D | Pik3r1 |
| D | Pip5k2b |
| D | Pkp3 |
| D | Pla2g2f |
| D | Plekha7 |
| D | Plxnb2 |
| D | Plxnc1 |
| D | Pom121 |
| D | Pou2f1 |
| D | Ppm1k |
| D | Ppme1 |
| D | Ppp1r12a |
| D | Ppp1r12b |
| D | Ppp2cb |
| D | Prdm2 |
| D | Prei4 |
| D | Prkrir |
| D | Prpf4b |
| D | Prr8 |
| D | Psme4 |
| D | Ptcd3 |
| D | Ptms |
| D | Ptpn11 |
| D | Pum2 |
| D | R3hdm1 |
| D | Rab12 |
| D | Rab43 |
| D | Rad21 |
| D | Rai14 |
| D | Ranbp6 |
| D | Rassf4 |
| D | Rbm24 |
| D | Rbm27 |
| D | Rfxdc2 |
| D | Rin2 |
| D | Rnf103 |
| D | Rnf146 |
| D | Rnf214 |
| D | Rnf214 |
| D | Rnf6 |
| D | Rock1 |
| D | Rock2 |
| D | Rsrc1 |
| D | Sbf2 |
| D | Sbno1 |
| D | Sbno1 |
| D | Senp2 |
| D | Setd8 |
| D | Setx |
| D | Sf3b3 |
| D | Sfrs10 |
| D | Sfrs11 |
| D | Sfrs12 |
| D | Shprh |
| D | Slc38a2 |
| D | Slc4a7 |
| D | Slc4a8 |
| D | Slc6a6 |
| D | Slmap |
| D | Smarca5 |
| D | Snap23 |
| D | Snap23 |
| D | Son |
| D | Sp4 |
| D | Spag9 |
| D | Spop |
| D | Srbd1 |
| D | Stk3 |
| D | Stra6 |
| D | Strn3 |
| D | Suhw4 |
| D | Suv420h1 |
| D | Suz12 |
| D | Taf1 |
| D | Taf1 |
| D | TC1602578 |
| D | TC1619269 |
| D | TC1659888 |
| D | TC1665684 |
| D | TC1676462 |
| D | TC1735696 |
| D | Terf2ip |
| D | Tex21 |
| D | Tgfbr1 |
| D | Tgm5 |
| D | Tgoln2 |
| D | Tmem161b |
| D | Tnks |
| D | Tnks1bp1 |
| D | Tnks2 |
| D | Tnrc15 |
| D | Tob2 |
| D | Tob2 |
| D | Traf4 |
| D | Tram2 |
| D | Txndc2 |
| D | Ubap2l |
| D | Upf3b |
| D | Usp14 |
| D | Usp15 |
| D | Usp29 |
| D | Usp36 |
| D | Usp42 |
| D | Vamp2 |
| D | Vcl |
| D | Vps13c |
| D | Vps35 |
| D | Wac |
| D | Wwp2 |
| D | Xpo4 |
| D | Xpr1 |
| D | Zbtb11 |
| D | Zbtb7a |
| D | Zc3h13 |
| D | Zc3h6 |
| D | Zc3h6 |
| D | Zcchc11 |
| D | Zcchc8 |
| D | Zeb2 |
| D | Zfp106 |
| D | Zfp146 |
| D | Zfp236 |
| D | Zfp318 |
| D | Zfp397 |
| D | Zfp426 |
| D | Zfp451 |
| D | Zfp609 |
| D | Zfp644 |
| D | Zfp668 |
| D | Zfp748 |
| D | Zfp75 |
| D | Zfp91 |
| D | Zfr |
| D | Zfr |
| D | Zfy1 |
| D | Zfy2 |
| D | Zhx1 |
| D | Zranb2 |
| E | 0610038F07Rik |
| E | 1110049F12Rik |
| E | 1700020O03Rik |
| E | 2310047M10Rik |
| E | 2410018C17Rik |
| E | 2610019A05Rik |
| E | 2610024G14Rik |
| E | 2610039C10Rik |
| E | 2610206B13Rik |
| E | A_51_P305350 |
| E | A230046K03Rik |
| E | AA408296 |
| E | Abcd1 |
| E | Abcd1 |
| E | Acn9 |
| E | Aifm1 |
| E | Anapc4 |
| E | Arcn1 |
| E | Arl6 |
| E | Atg3 |
| E | B4galt2 |
| E | Bat5 |
| E | Ccdc98 |
| E | Cd302 |
| E | Cdc42ep5 |
| E | Cdc42ep5 |
| E | Cdk4 |
| E | Cdk7 |
| E | Chd4 |
| E | Cops3 |
| E | Cpeb2 |
| E | Crlf3 |
| E | Crsp8 |
| E | D14Ertd581e |
| E | Ddx19a |
| E | Dhx15 |
| E | Dnajb9 |
| E | E2f5 |
| E | Eid2 |
| E | Eif3s2 |
| E | Enoph1 |
| E | ENSMUST00000085365 |
| E | Gpaa1 |
| E | Gpr19 |
| E | Ints4 |
| E | Josd1 |
| E | Kif18a |
| E | Klf3 |
| E | Lmo4 |
| E | Lypla1 |
| E | Mesdc2 |
| E | Mfn1 |
| E | Mrpl3 |
| E | Mrpl44 |
| E | Mvk |
| E | NAP025822-1 |
| E | NAP057020-1 |
| E | Ndfip2 |
| E | Nsg1 |
| E | Nt5c2 |
| E | Nt5c3l |
| E | Nup205 |
| E | Pacs1 |
| E | Parp2 |
| E | Pigl |
| E | Plrg1 |
| E | Praf2 |
| E | Prpf31 |
| E | Psme4 |
| E | Psmf1 |
| E | Ptplb |
| E | Qtrtd1 |
| E | Rab5a |
| E | Rabif |
| E | Rars2 |
| E | Rpl29 |
| E | Sec13 |
| E | Sec13 |
| E | Siah1a |
| E | Slc6a6 |
| E | Snx27 |
| E | Spata6 |
| E | Strn4 |
| E | TC1638264 |
| E | TC1648248 |
| E | Tln1 |
| E | Tmem177 |
| E | Tmem186 |
| E | Tmem39b |
| E | Tmem55b |
| E | Trpc4ap |
| E | Txndc14 |
| E | Ufd1l |
| E | Unk |
| E | Vbp1 |
| E | Vps26b |
| E | Wdr8 |
| E | Zfp142 |
| E | Zfp54 |
| E | Zfp655 |
| E | Zkscan5 |
| F | 2-Mar |
| F | 0610030E20Rik |
| F | 1110001J03Rik |
| F | 1110012N22Rik |
| F | 1500032D16Rik |
| F | 1700081L11Rik |
| F | 2310002J21Rik |
| F | 2310076L09Rik |
| F | 2410015M20Rik |
| F | 2600005O03Rik |
| F | 2600005O03Rik |
| F | 2610002D18Rik |
| F | 2610029G23Rik |
| F | 2810417H13Rik |
| F | 2900062L11Rik |
| F | 3110009E18Rik |
| F | 4921517D21Rik |
| F | 4930535E21Rik |
| F | 4931406C07Rik |
| F | 5730553K21 |
| F | 9430025N12Rik |
| F | 9430025N12Rik |
| F | 9530058B02Rik |
| F | A_51_P327496 |
| F | A_52_P1197466 |
| F | A530082C11Rik |
| F | A730081D07Rik |
| F | Abcb8 |
| F | Acad11 |
| F | Acadvl |
| F | Acat1 |
| F | Aco2 |
| F | Acsl1 |
| F | Acsl1 |
| F | Actc1 |
| F | Actn2 |
| F | Adamtsl3 |
| F | Adcy6 |
| F | Adprhl1 |
| F | Agmat |
| F | AK038328 |
| F | AK042636 |
| F | AK161939 |
| F | AK199677 |
| F | Akap6 |
| F | Aldh5a1 |
| F | Ank |
| F | Ank1 |
| F | Ankrd1 |
| F | Anln |
| F | Aoc3 |
| F | Asb2 |
| F | Asf1b |
| F | Atp2a2 |
| F | AW822216 |
| F | B230317C12Rik |
| F | B430006D22Rik |
| F | BC038822 |
| F | BI738048 |
| F | Birc5 |
| F | Brca1 |
| F | Brip1 |
| F | Bub1b |
| F | BY439412 |
| F | C030006K11Rik |
| F | C230094A16Rik |
| F | Casc5 |
| F | Ccdc117 |
| F | Ccna2 |
| F | Ccnb1 |
| F | Ccnf |
| F | Cdc20 |
| F | Cdc45l |
| F | Cdca2 |
| F | Cdca3 |
| F | Cdca5 |
| F | Cdca8 |
| F | Cdkn3 |
| F | Cdr2 |
| F | Cenpa |
| F | Cenpe |
| F | Cenph |
| F | Cep55 |
| F | Chaf1a |
| F | Chid1 |
| F | Chpt1 |
| F | Cit |
| F | Cks1b |
| F | Clu |
| F | Clybl |
| F | Cox7a1 |
| F | Cox8b |
| F | Creb3l3 |
| F | Cs |
| F | Csrp3 |
| F | Cyc1 |
| F | D10Jhu81e |
| F | D16H22S680E |
| F | D930010J01Rik |
| F | Dbi |
| F | Dci |
| F | Ddhd2 |
| F | Doc2g |
| F | Dph1 |
| F | Dtl |
| F | Dut |
| F | Dut |
| F | E130306D19Rik |
| F | E2f7 |
| F | Ect2 |
| F | Elf4 |
| F | Eme1 |
| F | Eno3 |
| F | ENSMUST00000063037 |
| F | ENSMUST00000103507 |
| F | Ercc6l |
| F | Espl1 |
| F | Fabp3 |
| F | Fahd1 |
| F | Fbxo5 |
| F | Fhl2 |
| F | Fhod3 |
| F | Frmd5 |
| F | Gale |
| F | Gm1418 |
| F | Gmpr |
| F | Gpd1 |
| F | Gpd1 |
| F | Gpx7 |
| F | Grit |
| F | Gstz1 |
| F | H2afx |
| F | Hhatl |
| F | Hibch |
| F | Hist1h1b |
| F | Hist1h2aa |
| F | Hist3h2a |
| F | Hmga2 |
| F | Htra3 |
| F | Id1 |
| F | Id1 |
| F | Idh2 |
| F | Idh3a |
| F | Ikzf4 |
| F | Itgb1bp2 |
| F | Kcna1 |
| F | Kif1c |
| F | Kif20a |
| F | Kif22 |
| F | Kif23 |
| F | Kif4 |
| F | Kifc1 |
| F | Klf13 |
| F | Klf7 |
| F | Klf7 |
| F | Krt80 |
| F | Ldha |
| F | Lmod3 |
| F | LOC627563 |
| F | Lrrc8a |
| F | Lyrm5 |
| F | Mad2l1 |
| F | Mapk10 |
| F | Mastl |
| F | Mb |
| F | Mccc1 |
| F | Mccc1 |
| F | Mcm5 |
| F | Mcm6 |
| F | Mcm8 |
| F | Mdh1 |
| F | Mdh1 |
| F | Mfn2 |
| F | Mki67 |
| F | Mki67 |
| F | Mkln1 |
| F | Mlxipl |
| F | Muc4 |
| F | Myl4 |
| F | Myom2 |
| F | Myoz2 |
| F | NAP028620-1 |
| F | NAP058291-1 |
| F | Ncaph |
| F | Ndufa10 |
| F | Ndufab1 |
| F | Ndufb10 |
| F | Ndufc1 |
| F | Ndufs2 |
| F | Nexn |
| F | Nsl1 |
| F | Nudt4 |
| F | Numb |
| F | Nusap1 |
| F | Odz4 |
| F | Olfr821 |
| F | Pacsin3 |
| F | Paf1 |
| F | Pafah1b3 |
| F | Pcca |
| F | Pcp2 |
| F | Pde4dip |
| F | Pdha1 |
| F | Pdlim5 |
| F | Pfkm |
| F | Pgam2 |
| F | Phf19 |
| F | Plk1 |
| F | Plxna2 |
| F | Plxna2 |
| F | Plxna2 |
| F | Pofut2 |
| F | Pola1 |
| F | Ppara |
| F | Ppargc1a |
| F | Ppargc1a |
| F | Ppp1r13l |
| F | Prdx2 |
| F | Prim1 |
| F | Prkag2 |
| F | Ptn |
| F | Pygb |
| F | Pygm |
| F | Racgap1 |
| F | Rad51 |
| F | Rad51l3 |
| F | Rcn3 |
| F | Rcn3 |
| F | Retnla |
| F | Rin3 |
| F | Rtbdn |
| F | S100b |
| F | S3-12 |
| F | Sgcg |
| F | Sh2b3 |
| F | Sh2d3c |
| F | Shcbp1 |
| F | Slc14a2 |
| F | Slc22a1 |
| F | Slc25a19 |
| F | Slc25a3 |
| F | Slc25a35 |
| F | Slc25a4 |
| F | Slc25a4 |
| F | Slc38a2 |
| F | Slc4a3 |
| F | Slc4a3 |
| F | Sln |
| F | Smc2 |
| F | Smc2 |
| F | Snrp70 |
| F | Spag5 |
| F | Stard10 |
| F | Suclg1 |
| F | Tanc1 |
| F | Tbx4 |
| F | TC1633516 |
| F | TC1675885 |
| F | Tcap |
| F | Terc |
| F | Tfdp1 |
| F | Tmem33 |
| F | Tmem38a |
| F | Tmem48 |
| F | Tnni3 |
| F | Tnnt2 |
| F | Tnrc6b |
| F | Top2a |
| F | Tpm1 |
| F | Tpm1 |
| F | Tra2a |
| F | Trip13 |
| F | Ttn |
| F | Ttn |
| F | Ttn |
| F | Tubb5 |
| F | Tubb5 |
| F | Tyms |
| F | Tyms |
| F | Tyms-ps |
| F | Ubfd1 |
| F | Ugt2b34 |
| F | Ung |
| F | Uqcr |
| F | Wdhd1 |
| F | Yipf7 |
| F | Zcd1 |
| F | Zfp383 |
| F | Zfp9 |
| G | 1110006O24Rik |
| G | 1110014J01Rik |
| G | 1200015N20Rik |
| G | 1700019G17Rik |
| G | 1700105P06Rik |
| G | 1810049H19Rik |
| G | 2410004P03Rik |
| G | 3110048L19Rik |
| G | 4933400C05Rik |
| G | 9430010O03Rik |
| G | A_51_P451508 |
| G | A030003K02Rik |
| G | A630055G03Rik |
| G | Aak1 |
| G | Adra1d |
| G | AK006309 |
| G | AK034319 |
| G | AK035470 |
| G | AK043486 |
| G | AK050700 |
| G | AK085302 |
| G | Atp7b |
| G | AU020772 |
| G | AW536275 |
| G | B430319H21Rik |
| G | BC051212 |
| G | BC051628 |
| G | BC068233 |
| G | BG084650 |
| G | Bloc1s3 |
| G | Braf |
| G | Bzrpl1 |
| G | C1rl |
| G | Camk1g |
| G | Card14 |
| G | Cd3e |
| G | Celsr3 |
| G | Cntnap1 |
| G | Cplx2 |
| G | Crim2 |
| G | Csnk1a1 |
| G | Cyp17a1 |
| G | Cyp46a1 |
| G | Drd3 |
| G | Dyrk1b |
| G | Dyrk1b |
| G | E230015B07Rik |
| G | Ecel1 |
| G | EG240038 |
| G | EG631624 |
| G | ENSMUSG00000044227 |
| G | Fthl17 |
| G | Gabrb2 |
| G | Gal |
| G | Gdap1l1 |
| G | Gnai1 |
| G | Gpr173 |
| G | Gpr37l1 |
| G | Gpr44 |
| G | Gprc5d |
| G | Gscl |
| G | Gtrgeo22 |
| G | Hcn4 |
| G | Hhat |
| G | Hip1 |
| G | Iqcf1 |
| G | Kif26a |
| G | Klf8 |
| G | Krtap16-10 |
| G | Lalba |
| G | Lce1l |
| G | Lhfpl4 |
| G | Lhx4 |
| G | Ly6g6d |
| G | Mapk8ip2 |
| G | Mc4r |
| G | MGC117846 |
| G | NAP005803-002 |
| G | NAP018688-001 |
| G | NAP025478-001 |
| G | NAP039199-1 |
| G | NAP047434-1 |
| G | NAP067058-1 |
| G | NAP070939-1 |
| G | NAP071025-1 |
| G | NAP105744-1 |
| G | NAP108144-1 |
| G | NAP121938-001 |
| G | Nfe2l3 |
| G | Npal3 |
| G | Odf4 |
| G | Og9x |
| G | Olfr1030 |
| G | Olfr1344 |
| G | Olfr1393 |
| G | Olfr1444 |
| G | Olfr457 |
| G | Olfr48 |
| G | OTTMUSG00000008584 |
| G | Pcsk1n |
| G | Pknox2 |
| G | Plcg1 |
| G | Plcl1 |
| G | Plec1 |
| G | Prkcc |
| G | Prss3 |
| G | Psd |
| G | Ptk7 |
| G | Raver2 |
| G | Rex2 |
| G | Sars2 |
| G | Scn10a |
| G | Sema5b |
| G | Slc20a1 |
| G | Slit1 |
| G | Slmo1 |
| G | Speg |
| G | Sprn |
| G | Sprr2g |
| G | Strbp |
| G | Taar4 |
| G | Tacr1 |
| G | TC1625496 |
| G | TC1666853 |
| G | TC1672155 |
| G | TC1679528 |
| G | TC1686545 |
| G | TC1708273 |
| G | TC1721634 |
| G | Tdh |
| G | Tle3 |
| G | Tlx2 |
| G | Trim45 |
| G | Tulp1 |
| G | V1ra4 |
| G | Wasf1 |
| G | Zfp179 |
| H | 3-Mar |
| H | 1110054O05Rik |
| H | 1700029F09Rik |
| H | 1700029F09Rik |
| H | 2310008M10Rik |
| H | 2310008M10Rik |
| H | 2410001C21Rik |
| H | 2410019A14Rik |
| H | 2810452K22Rik |
| H | 3110082I17Rik |
| H | 9630042H07Rik |
| H | Abhd10 |
| H | AI848100 |
| H | AK042960 |
| H | Anxa2 |
| H | Ap4e1 |
| H | Armet |
| H | Atf4 |
| H | BC025546 |
| H | BC049807 |
| H | Blcap |
| H | Bxdc2 |
| H | Ccdc49 |
| H | Ccdc94 |
| H | Ccnc |
| H | Cct3 |
| H | Cdc34 |
| H | Cdc42ep4 |
| H | Cfl1 |
| H | Chmp6 |
| H | Cnih4 |
| H | Coq10b |
| H | Coro1c |
| H | Crebl1 |
| H | Cry1 |
| H | Cwf19l1 |
| H | Cxcr7 |
| H | D11Ertd497e |
| H | D1Ertd622e |
| H | D230037D09Rik |
| H | Dars |
| H | Derl1 |
| H | Dkc1 |
| H | Dtwd1 |
| H | Dus4l |
| H | Ecd |
| H | Edg5 |
| H | Eif2s1 |
| H | Eif5a |
| H | ENSMUST00000075235 |
| H | Exosc1 |
| H | Exosc1 |
| H | Farsa |
| H | Farsb |
| H | Fbn1 |
| H | Fbn1 |
| H | Fbxl5 |
| H | Fkbp1a |
| H | Fubp1 |
| H | Ghitm |
| H | Glmn |
| H | Gmps |
| H | Iars |
| H | Itgb4bp |
| H | Iws1 |
| H | Lepre1 |
| H | Loh12cr1 |
| H | Lrrc40 |
| H | Lsm2 |
| H | Lsm2 |
| H | Mars |
| H | Mars2 |
| H | Mbtps2 |
| H | Med8 |
| H | Moap1 |
| H | Mrpl15 |
| H | Mrpl37 |
| H | Mrps18b |
| H | Msn |
| H | NAP026555-1 |
| H | NAP057019-1 |
| H | NAP093832-001 |
| H | NAP103789-1 |
| H | NAP104272-1 |
| H | Nkrf |
| H | Nol14 |
| H | Nola1 |
| H | Npm1 |
| H | Nsmce2 |
| H | Nudt5 |
| H | Nup37 |
| H | Nup43 |
| H | Pdcd6ip |
| H | Pemt |
| H | Pgm1 |
| H | Pla2g4a |
| H | Plaa |
| H | Pold2 |
| H | Pole4 |
| H | Polr3d |
| H | Ppp1r2 |
| H | Ppp1r2 |
| H | Ppp2r5c |
| H | Prmt1 |
| H | Psmb2 |
| H | Psmd14 |
| H | Pus3 |
| H | Rab11a |
| H | Rabggtb |
| H | Rabggtb |
| H | Rap1b |
| H | Rassf1 |
| H | Rbm22 |
| H | Rcl1 |
| H | Rcl1 |
| H | Rg9mtd1 |
| H | Ripk1 |
| H | Rpl29 |
| H | Rtn4 |
| H | Scamp4 |
| H | Sec61a1 |
| H | Sfrs2 |
| H | Shq1 |
| H | Snrpa |
| H | Snrpa1 |
| H | Snx12 |
| H | Socs4 |
| H | Spry4 |
| H | Srfbp1 |
| H | Srp9 |
| H | Stoml2 |
| H | Surf4 |
| H | Tardbp |
| H | Tbc1d7 |
| H | TC1660970 |
| H | Tinagl |
| H | Tmem5 |
| H | Tpm3 |
| H | Tprkb |
| H | Trim27 |
| H | Tsc22d2 |
| H | Ttc1 |
| H | Twistnb |
| H | U2af1 |
| H | Ube2a |
| H | Ube2d3 |
| H | Ube2d3 |
| H | Ube2f |
| H | Ube2g2 |
| H | Ube2j2 |
| H | Uchl4 |
| H | Wdr18 |
| H | Wdr70 |
| H | Wdr77 |
| H | Xbp1 |
| H | Yrdc |
| H | Yrdc |
| H | Zdhhc13 |
| I | 1700020C07Rik |
| I | 1700049J03Rik |
| I | 4921530G04Rik |
| I | 9030025P20Rik |
| I | A630089N07Rik |
| I | Aff4 |
| I | AI118078 |
| I | AI987944 |
| I | AK006604 |
| I | AK082070 |
| I | AK082839 |
| I | Aldoa-ps1 |
| I | Ankrd11 |
| I | B230315N10Rik |
| I | BC002059 |
| I | BC029127 |
| I | BC029127 |
| I | BC029127 |
| I | BC031441 |
| I | BC048507 |
| I | BC050196 |
| I | C330013J21Rik |
| I | C77080 |
| I | C87436 |
| I | Cacna2d2 |
| I | Cdk5r2 |
| I | D330037H05Rik |
| I | D5Ertd135e |
| I | Daf2 |
| I | Dot1l |
| I | Dpp9 |
| I | E130304I02Rik |
| I | E2f4 |
| I | EG434179 |
| I | EG435336 |
| I | EG435337 |
| I | EG435366 |
| I | EG435970 |
| I | EG546361 |
| I | EG627782 |
| I | EG666606 |
| I | ENSMUSG00000066507 |
| I | ENSMUSG00000069586 |
| I | ENSMUSG00000070586 |
| I | ENSMUST00000074313 |
| I | ENSMUST00000075032 |
| I | Esrrg |
| I | Fbs1 |
| I | Fkbp11 |
| I | Foxh1 |
| I | Grin2d |
| I | Ifrg15 |
| I | Irs4 |
| I | Jph4 |
| I | Lhb |
| I | Lmtk2 |
| I | LOC383707 |
| I | LOC619975 |
| I | LOC632900 |
| I | LOC638058 |
| I | LOC670626 |
| I | LOC675815 |
| I | Ly6g5b |
| I | Ly6g6c |
| I | M17518 |
| I | Matk |
| I | Mchr1 |
| I | Mdga2 |
| I | Nab2 |
| I | NAP000001-064 |
| I | NAP020770-001 |
| I | NAP020861-001 |
| I | NAP026611-1 |
| I | NAP026710-1 |
| I | NAP027922-1 |
| I | NAP030950-1 |
| I | NAP059572-1 |
| I | NAP062835-1 |
| I | NAP071006-1 |
| I | NAP071064-1 |
| I | NAP096498-001 |
| I | NAP102079-1 |
| I | NAP102441-1 |
| I | NAP102548-1 |
| I | NAP102683-1 |
| I | NAP103757-1 |
| I | NAP113018-1 |
| I | Ngb |
| I | Olfr1170 |
| I | Olfr1263 |
| I | Olfr1350 |
| I | Olfr1459 |
| I | Olfr313 |
| I | Osbpl7 |
| I | OTTMUSG00000005065 |
| I | OTTMUSG00000015730 |
| I | OTTMUSG00000015743 |
| I | OTTMUSG00000015743 |
| I | Pcbp1 |
| I | Pex14 |
| I | Pou4f1 |
| I | Ppp3r1 |
| I | Ppp5c |
| I | Prp2 |
| I | Rbak |
| I | Rhbdl3 |
| I | RP23-406N5.2 |
| I | Rps21 |
| I | Rps21 |
| I | Samd10 |
| I | Scrt2 |
| I | Set |
| I | Sox15 |
| I | Spata21 |
| I | Spint2 |
| I | Ssb |
| I | Syn1 |
| I | Synpo |
| I | Tbrg4 |
| I | TC1631533 |
| I | TC1637123 |
| I | Tmem165 |
| I | Tmem174 |
| I | Ubxd2 |
| I | Ybx2 |
| I | Zfp273 |
| I | Zfp53 |
| I | Zfp74 |
| I | Zfp759 |
| J | 1190002H23Rik |
| J | 2310004I24Rik |
| J | 2310028N02Rik |
| J | 2310045N01Rik |
| J | 2310061J03Rik |
| J | 2610027H17Rik |
| J | 2810055G20Rik |
| J | 3110067C02Rik |
| J | 4631426J05Rik |
| J | 4631427C17Rik |
| J | 4921533L14Rik |
| J | 4930431B09Rik |
| J | 6030446N20Rik |
| J | 6530404N21Rik |
| J | 6720467C03Rik |
| J | A830080D01Rik |
| J | A930025D01Rik |
| J | AA438147 |
| J | Abcb7 |
| J | Abcg2 |
| J | Abcg2 |
| J | Acaca |
| J | Acaca |
| J | Acly |
| J | Adal |
| J | Adrb2 |
| J | AJ237917 |
| J | AK030738 |
| J | AK032764 |
| J | AK040092 |
| J | AK041471 |
| J | AK041753 |
| J | AK043743 |
| J | AK045982 |
| J | AK048514 |
| J | AK079806 |
| J | AK082620 |
| J | AK084144 |
| J | AK084349 |
| J | AK085783 |
| J | Akap2 |
| J | Akt3 |
| J | Aldh9a1 |
| J | Arhgef7 |
| J | Arid4a |
| J | Arl4d |
| J | Atf2 |
| J | Atf7 |
| J | Atg10 |
| J | Atpbd1c |
| J | Bat3 |
| J | Bbs7 |
| J | BC024479 |
| J | BC024694 |
| J | BC043301 |
| J | BC046331 |
| J | BC067068 |
| J | Bckdhb |
| J | Bckdhb |
| J | Bcl11b |
| J | Bcl11b |
| J | Bgn |
| J | Brwd2 |
| J | Btaf1 |
| J | C330016O10Rik |
| J | C87436 |
| J | Calm3 |
| J | Canx |
| J | Carf |
| J | Cbx8 |
| J | Cd47 |
| J | Cdc91l1 |
| J | Cdr2l |
| J | Cdyl2 |
| J | Cenpc1 |
| J | Chn1 |
| J | Ckb |
| J | Clcc1 |
| J | Clcn3 |
| J | Clock |
| J | Col6a1 |
| J | Col7a1 |
| J | Cpt1a |
| J | Ctbp2 |
| J | Ctdspl2 |
| J | Ctnna1 |
| J | Cttnbp2nl |
| J | D830012I24Rik |
| J | Dbndd2 |
| J | Dcbld2 |
| J | Ddef1 |
| J | Dennd3 |
| J | Dhrs1 |
| J | Dhrs7b |
| J | Dirc2 |
| J | Dlg5 |
| J | Dll4 |
| J | Dsp |
| J | Dtx1 |
| J | Edc3 |
| J | Edg6 |
| J | Eea1 |
| J | Elk4 |
| J | Elmo1 |
| J | ENSMUSG00000075516 |
| J | ENSMUST00000039827 |
| J | ENSMUST00000057459 |
| J | ENSMUST00000103201 |
| J | Epb4.1l2 |
| J | Ergic3 |
| J | Exdl2 |
| J | Exoc3l |
| J | Fastkd1 |
| J | Fbxo45 |
| J | Fdps |
| J | Fgf7 |
| J | Gdap5 |
| J | Gdi1 |
| J | Gemin4 |
| J | Ghr |
| J | Ghr |
| J | Ghr |
| J | Gimap1 |
| J | Git2 |
| J | Gle1l |
| J | Gle1l |
| J | Gmps |
| J | Gnas |
| J | Gnl3l |
| J | Golgb1 |
| J | Gprk5 |
| J | H1f0 |
| J | Hace1 |
| J | Hes1 |
| J | Hpcal1 |
| J | Ift57 |
| J | Ift57 |
| J | Ift57 |
| J | Igh-6 |
| J | Ikbkg |
| J | Il7 |
| J | Irf3 |
| J | Kctd12 |
| J | Klf2 |
| J | Lancl1 |
| J | Laptm4a |
| J | Laptm4b |
| J | Lmbrd1 |
| J | Lmcd1 |
| J | Maged2 |
| J | Mark3 |
| J | Matr3 |
| J | Mertk |
| J | Mfsd1 |
| J | Mfsd8 |
| J | Mical2 |
| J | Mrps25 |
| J | Mrps7 |
| J | Msi2 |
| J | Mtch1 |
| J | Mtm1 |
| J | Mutyh |
| J | Mxra7 |
| J | Myrip |
| J | Nqo2 |
| J | Nr2c1 |
| J | Nsdhl |
| J | Nsdhl |
| J | Ntan1 |
| J | Ntrk3 |
| J | Numb |
| J | Ocln |
| J | Olfml3 |
| J | ORF61 |
| J | Otud6b |
| J | Pcaf |
| J | Pcdhb12 |
| J | Pcdhgb4 |
| J | Pde8a |
| J | Pde9a |
| J | Pdlim2 |
| J | Pex3 |
| J | Phf16 |
| J | Pias1 |
| J | Pias3 |
| J | Pigg |
| J | Pja1 |
| J | Plcb1 |
| J | Plcb1 |
| J | Plekhf1 |
| J | Polr2e |
| J | Ppap2a |
| J | Ppp2r1b |
| J | Prdx4 |
| J | Prdx6 |
| J | Prkag1 |
| J | Prkch |
| J | Prpf4b |
| J | Prpf8 |
| J | Rab34 |
| J | Rab3gap2 |
| J | Rabgap1 |
| J | Raf1 |
| J | Ralgps2 |
| J | Ranbp6 |
| J | Rbpms |
| J | RP23-136K12.4 |
| J | Rpa3 |
| J | Ryk |
| J | S100a16 |
| J | Saps3 |
| J | Sbds |
| J | Sec24a |
| J | Sema6d |
| J | Sfn |
| J | Sgms1 |
| J | Shroom2 |
| J | Slc10a6 |
| J | Slc24a5 |
| J | Slc25a39 |
| J | Slc30a9 |
| J | Slc31a1 |
| J | Slc35a5 |
| J | Slc6a8 |
| J | Smad7 |
| J | Smpd2 |
| J | Snf1lk2 |
| J | Snhg8 |
| J | Socs4 |
| J | Spire1 |
| J | Sppl3 |
| J | St3gal3 |
| J | Stk3 |
| J | Strn |
| J | Svep1 |
| J | Taf9b |
| J | Tal1 |
| J | Tbcel |
| J | Tbx3 |
| J | TC1605825 |
| J | TC1615264 |
| J | TC1655572 |
| J | TC1677116 |
| J | Tcn2 |
| J | Tcta |
| J | Tex261 |
| J | Tex261 |
| J | Tm2d3 |
| J | Tmem176a |
| J | Tmem62 |
| J | Tmem80 |
| J | Tmod3 |
| J | Tnrc6c |
| J | Tpt1 |
| J | Trappc3 |
| J | Trp53inp1 |
| J | Tsc1 |
| J | Tsc22d3 |
| J | Tsc22d3 |
| J | Tspan32 |
| J | Ttc15 |
| J | Ttl |
| J | Ttll1 |
| J | Tuba-rs1 |
| J | Tubb2c |
| J | Tusc3 |
| J | Tusc5 |
| J | Ube2h |
| J | Usp32 |
| J | Utp6 |
| J | Vamp1 |
| J | Vav3 |
| J | Vps29 |
| J | Wasl |
| J | Wdr32 |
| J | Wwc2 |
| J | Wwox |
| J | X83328 |
| J | Yipf1 |
| J | Yipf1 |
| J | Yipf3 |
| J | Zbtb40 |
| J | Zeb2 |
| J | Zfp248 |
| J | Zfp292 |
| J | Zfp444 |
| J | Zfp462 |
| J | Zfp462 |
| J | Zfp609 |
| J | Zfp68 |
| J | Zfp817 |
| J | Zik1 |
| K | 1-Mar |
| K | 5-Mar |
| K | 5-Mar |
| K | 6-Mar |
| K | 9-Sep |
| K | 0610010F05Rik |
| K | 0610012D17Rik |
| K | 0910001A06Rik |
| K | 0910001A06Rik |
| K | 0910001A06Rik |
| K | 1110007C09Rik |
| K | 1110007M04Rik |
| K | 1110007M04Rik |
| K | 1110018G07Rik |
| K | 1110018G07Rik |
| K | 1110032O16Rik |
| K | 1110038B12Rik |
| K | 1200015F23Rik |
| K | 1300018I05Rik |
| K | 1500001M20Rik |
| K | 1500002O20Rik |
| K | 1500003O03Rik |
| K | 1500012F01Rik |
| K | 1500034J01Rik |
| K | 1600014C10Rik |
| K | 1600016N20Rik |
| K | 1600027N09Rik |
| K | 1700001K19Rik |
| K | 1700003O08Rik |
| K | 1700012H17Rik |
| K | 1700017B05Rik |
| K | 1700019N12Rik |
| K | 1700027J05Rik |
| K | 1700041G16Rik |
| K | 1700049L16Rik |
| K | 1810009K13Rik |
| K | 1810054D07Rik |
| K | 2010002N04Rik |
| K | 2010003J03Rik |
| K | 2010012C16Rik |
| K | 2010012O05Rik |
| K | 2010106G01Rik |
| K | 2010106G01Rik |
| K | 2010106G01Rik |
| K | 2010109K11Rik |
| K | 2010209O12Rik |
| K | 2010305A19Rik |
| K | 2010305A19Rik |
| K | 2010310D06Rik |
| K | 2010316F05Rik |
| K | 2210008N01Rik |
| K | 2210403K04Rik |
| K | 2210408I21Rik |
| K | 2310008H09Rik |
| K | 2310009B15Rik |
| K | 2310011J03Rik |
| K | 2310014H01Rik |
| K | 2310016F22Rik |
| K | 2310022A10Rik |
| K | 2310040C09Rik |
| K | 2310043J07Rik |
| K | 2310044G17Rik |
| K | 2310047O13Rik |
| K | 2410001C21Rik |
| K | 2410012H22Rik |
| K | 2410014A08Rik |
| K | 2410014A08Rik |
| K | 2410127E18Rik |
| K | 2610028A01Rik |
| K | 2610203C20Rik |
| K | 2610203E10Rik |
| K | 2610208M17Rik |
| K | 2610524H06Rik |
| K | 2700007P21Rik |
| K | 2700007P21Rik |
| K | 2700019D07Rik |
| K | 2700029M09Rik |
| K | 2700094F01Rik |
| K | 2810002O09Rik |
| K | 2810022L02Rik |
| K | 2810403A07Rik |
| K | 2810439F02Rik |
| K | 2810474O19Rik |
| K | 3110001I22Rik |
| K | 3110001K24Rik |
| K | 3110003A17Rik |
| K | 3110003A17Rik |
| K | 3110005G23Rik |
| K | 3200002M19Rik |
| K | 3200002M19Rik |
| K | 3300001M20Rik |
| K | 4632417K18Rik |
| K | 4632434I11Rik |
| K | 4732429D16Rik |
| K | 4833446K15Rik |
| K | 4833446K15Rik |
| K | 4921517L17Rik |
| K | 4930471M23Rik |
| K | 4933403F05Rik |
| K | 4933426M11Rik |
| K | 4933426M11Rik |
| K | 4933440N22Rik |
| K | 5031439G07Rik |
| K | 5133401H06Rik |
| K | 5530401N12Rik |
| K | 5730458M16Rik |
| K | 5730508B09Rik |
| K | 5730528L13Rik |
| K | 5730559C18Rik |
| K | 5830400J07Rik |
| K | 5830416P10Rik |
| K | 5830417C01Rik |
| K | 5830418K08Rik |
| K | 5830443L24Rik |
| K | 5830472M02Rik |
| K | 5830482F20Rik |
| K | 5830482F20Rik |
| K | 6030408C04Rik |
| K | 6330407G11Rik |
| K | 6330409N04Rik |
| K | 6330411E07Rik |
| K | 6330416G13Rik |
| K | 6330578E17Rik |
| K | 6430511F03 |
| K | 6720456H09Rik |
| K | 8030431J09Rik |
| K | 9030425E11Rik |
| K | 9130017C17Rik |
| K | 9130017N09Rik |
| K | 9130227C08Rik |
| K | 9430008C03Rik |
| K | 9430034N14Rik |
| K | 9430038I01Rik |
| K | 9430098F02Rik |
| K | 9530028C05 |
| K | 9530068E07Rik |
| K | 9630055N22Rik |
| K | 9830124H08Rik |
| K | 9930023K05Rik |
| K | 9930023K05Rik |
| K | A_51_P462771 |
| K | A_52_P1013432 |
| K | A_52_P981179 |
| K | A030007L17Rik |
| K | A130099P19Rik |
| K | A230050P20Rik |
| K | A230050P20Rik |
| K | A230097K15Rik |
| K | A430057L12Rik |
| K | A430084P05Rik |
| K | A430107D22Rik |
| K | A530032D15Rik |
| K | A630082K20Rik |
| K | A730011C13Rik |
| K | A930001N09Rik |
| K | AA407452 |
| K | AA409316 |
| K | AA960436 |
| K | Aars |
| K | Aars |
| K | Abcb1b |
| K | Abcb1b |
| K | Abcc3 |
| K | Abhd12 |
| K | Abhd2 |
| K | Abhd2 |
| K | Abi3 |
| K | Abl2 |
| K | Ablim1 |
| K | Abr |
| K | Abtb2 |
| K | Acp2 |
| K | Acsbg1 |
| K | Acvr1b |
| K | Adam15 |
| K | Adam21 |
| K | Adamts2 |
| K | Adamts3 |
| K | Adar |
| K | Adm |
| K | Adora2b |
| K | Adora3 |
| K | Adrbk1 |
| K | Adrbk1 |
| K | Adrbk1 |
| K | Aff1 |
| K | Aftph |
| K | Agpat5 |
| K | Ahnak |
| K | Ahnak |
| K | Ahrr |
| K | AI447904 |
| K | AI447904 |
| K | AI451617 |
| K | AI481105 |
| K | Aif1 |
| K | AK008862 |
| K | AK012844 |
| K | AK013113 |
| K | AK013239 |
| K | AK013903 |
| K | AK030647 |
| K | AK032625 |
| K | AK035243 |
| K | AK036874 |
| K | AK037475 |
| K | AK038388 |
| K | AK038845 |
| K | AK039806 |
| K | AK040334 |
| K | AK040628 |
| K | AK041551 |
| K | AK041801 |
| K | AK042092 |
| K | AK043151 |
| K | AK045158 |
| K | AK046873 |
| K | AK047447 |
| K | AK048085 |
| K | AK048147 |
| K | AK050084 |
| K | AK050741 |
| K | AK050867 |
| K | AK051012 |
| K | AK053561 |
| K | AK054376 |
| K | AK076354 |
| K | AK076900 |
| K | AK079230 |
| K | AK079518 |
| K | AK080372 |
| K | AK081327 |
| K | AK082068 |
| K | AK082409 |
| K | AK082956 |
| K | AK084024 |
| K | AK084575 |
| K | AK085307 |
| K | AK087246 |
| K | AK087356 |
| K | AK087372 |
| K | AK087429 |
| K | AK087625 |
| K | AK087943 |
| K | AK089714 |
| K | AK089832 |
| K | AK134580 |
| K | AK139506 |
| K | AK149247 |
| K | AK160276 |
| K | AK169992 |
| K | Ak2 |
| K | Akp2 |
| K | Akt3 |
| K | Alg9 |
| K | Alkbh3 |
| K | Als2 |
| K | Als2 |
| K | Als2cr13 |
| K | Als2cr13 |
| K | Amdhd2 |
| K | Amica1 |
| K | Ankrd15 |
| K | Ankrd17 |
| K | Ankrd49 |
| K | Ankrd57 |
| K | Anp32e |
| K | Anxa1 |
| K | Aoah |
| K | Aoc2 |
| K | Ap2b1 |
| K | Ap3s1 |
| K | Apaf1 |
| K | Apbb1ip |
| K | Apc |
| K | Apex1 |
| K | Apob48r |
| K | Apobec1 |
| K | Areg |
| K | Arf5 |
| K | Arf6 |
| K | Arf6 |
| K | Arg2 |
| K | Arhgap15 |
| K | Arhgap25 |
| K | Arhgap29 |
| K | Arhgap30 |
| K | Arhgap30 |
| K | Arhgef10 |
| K | Arhgef6 |
| K | Arid3a |
| K | Arid5a |
| K | Arl13b |
| K | Arl4c |
| K | Arl5c |
| K | Arl8a |
| K | Armc7 |
| K | Armcx5 |
| K | Arpc4 |
| K | Arrb2 |
| K | Arsi |
| K | Arts1 |
| K | Asb13 |
| K | Asb13 |
| K | Asb13 |
| K | Asb4 |
| K | Ascl3 |
| K | Asprv1 |
| K | Asxl2 |
| K | Atf1 |
| K | Atf1 |
| K | Atf3 |
| K | Atf6 |
| K | Atp13a3 |
| K | Atp2b1 |
| K | Atp6v1b2 |
| K | Atp7a |
| K | Atp8b1 |
| K | Atp8b2 |
| K | Atp8b2 |
| K | Atp8b4 |
| K | Atrn |
| K | Atrn |
| K | Atxn1 |
| K | Atxn7l1 |
| K | Atxn7l3 |
| K | Auh |
| K | AW108044 |
| K | AW112010 |
| K | AW122171 |
| K | AW552889 |
| K | Axl |
| K | Axud1 |
| K | AY078069 |
| K | Azi2 |
| K | B230217C12Rik |
| K | B230219D22Rik |
| K | B230312A22Rik |
| K | B2m |
| K | B3gnt2 |
| K | B3gnt2 |
| K | B430306N03Rik |
| K | B4galt1 |
| K | B930006L02Rik |
| K | Bai1 |
| K | Bak1 |
| K | Basp1 |
| K | Baz1a |
| K | BB160563 |
| K | BC004022 |
| K | BC004022 |
| K | BC005537 |
| K | BC005764 |
| K | BC013481 |
| K | BC013672 |
| K | BC013712 |
| K | BC016423 |
| K | BC016423 |
| K | BC019134 |
| K | BC023892 |
| K | BC025833 |
| K | BC027057 |
| K | BC027231 |
| K | BC032204 |
| K | BC033915 |
| K | BC033915 |
| K | BC035295 |
| K | BC037034 |
| K | BC051230 |
| K | Bcar1 |
| K | Bcl11a |
| K | Bcl2a1b |
| K | Bcl2l1 |
| K | Bcl2l1 |
| K | Bcl2l1 |
| K | Bcl2l11 |
| K | Bcl2l2 |
| K | Bcl3 |
| K | Bcl6b |
| K | Bcl7c |
| K | Bean |
| K | Bin3 |
| K | Birc2 |
| K | Birc3 |
| K | Blm |
| K | Bloc1s2 |
| K | Blvra |
| K | Bmper |
| K | Bmper |
| K | Brd2 |
| K | Brd3 |
| K | Bri3bp |
| K | Brms1 |
| K | Bst2 |
| K | Btbd4 |
| K | Btbd9 |
| K | Btg1 |
| K | BX529199 |
| K | Bysl |
| K | C130032J12Rik |
| K | C1qa |
| K | C1qbp |
| K | C1qc |
| K | C1qtnf6 |
| K | C1r |
| K | C2 |
| K | C2 |
| K | C230075M21Rik |
| K | C3 |
| K | C330006P03Rik |
| K | C330023M02Rik |
| K | C330023M02Rik |
| K | C330023M02Rik |
| K | C3ar1 |
| K | C530007A02Rik |
| K | Calm3 |
| K | Camk2d |
| K | Camk2d |
| K | Camk2n2 |
| K | Cant1 |
| K | Capg |
| K | Capn5 |
| K | Capza1 |
| K | Capza2 |
| K | Capza2 |
| K | Capzb |
| K | Car13 |
| K | Cars |
| K | Cars |
| K | Casp3 |
| K | Casp8 |
| K | Cbwd1 |
| K | Cbwd1 |
| K | Cbx1 |
| K | Ccdc109b |
| K | Ccdc134 |
| K | Ccdc23 |
| K | Ccdc59 |
| K | Ccdc86 |
| K | Ccdc88 |
| K | Ccdc9 |
| K | Ccdc90a |
| K | Ccl12 |
| K | Ccl2 |
| K | Ccl5 |
| K | Ccl8 |
| K | Ccng1 |
| K | Ccnl1 |
| K | Ccr2 |
| K | Ccrl2 |
| K | Cct6b |
| K | Cd14 |
| K | Cd244 |
| K | Cd244 |
| K | Cd248 |
| K | Cd248 |
| K | Cd300lf |
| K | Cd37 |
| K | Cd3eap |
| K | Cd44 |
| K | Cd52 |
| K | Cd68 |
| K | Cd7 |
| K | Cd86 |
| K | Cd93 |
| K | Cdk5r1 |
| K | Cdk8 |
| K | Cdt1 |
| K | Cebpb |
| K | Cebpb |
| K | Cenpq |
| K | Centa2 |
| K | Centb1 |
| K | Cep192 |
| K | CF747846 |
| K | Cflar |
| K | Cflar |
| K | Cfp |
| K | Ch25h |
| K | Chac1 |
| K | Chac2 |
| K | Chd7 |
| K | Chd7 |
| K | Chic2 |
| K | Chit1 |
| K | Chl1 |
| K | Chmp4b |
| K | Chmp4b |
| K | Cirh1a |
| K | Cish |
| K | Cklf |
| K | Cklf |
| K | Clasp1 |
| K | Clcn5 |
| K | Clcn6 |
| K | Cldn4 |
| K | Clec4a2 |
| K | Clec5a |
| K | Clic1 |
| K | Clic4 |
| K | Clip2 |
| K | Cln3 |
| K | Cln8 |
| K | Cndp2 |
| K | Cnih2 |
| K | Cnnm4 |
| K | Cog8 |
| K | Col1a1 |
| K | Col1a2 |
| K | Col23a1 |
| K | Col2a1 |
| K | Col3a1 |
| K | Col4a2 |
| K | Col5a1 |
| K | Col5a2 |
| K | Coro1a |
| K | Coro1a |
| K | Coro1b |
| K | Coro2a |
| K | Coro2a |
| K | Coro7 |
| K | Cotl1 |
| K | Cox10 |
| K | Cox15 |
| K | Cpeb3 |
| K | Cpsf2 |
| K | Cpsf4 |
| K | Creb3l1 |
| K | Creb5 |
| K | Crem |
| K | Crem |
| K | Crem |
| K | Crispld2 |
| K | Cryba4 |
| K | Csf1r |
| K | Csf2ra |
| K | Csf2rb2 |
| K | Csf3r |
| K | Csk |
| K | Csk |
| K | Csnk1d |
| K | Ctdp1 |
| K | Ctps |
| K | Ctsc |
| K | Ctsw |
| K | Ctsz |
| K | Cttnbp2nl |
| K | Cul2 |
| K | Cul5 |
| K | Cutc |
| K | Cxcl13 |
| K | Cxcl16 |
| K | Cxcr3 |
| K | Cxcr4 |
| K | Cyba |
| K | Cyp2d26 |
| K | Cyp4f18 |
| K | Cyp7b1 |
| K | Cyr61 |
| K | Cysltr1 |
| K | Cysltr1 |
| K | D030011O10Rik |
| K | D10Ertd438e |
| K | D13Wsu177e |
| K | D14Abb1e |
| K | D15Wsu75e |
| K | D17Wsu92e |
| K | D4Ertd22e |
| K | D5Ertd585e |
| K | D630023B12Rik |
| K | Dapk3 |
| K | Darc |
| K | Daxx |
| K | Dbn1 |
| K | Dbnl |
| K | Dbr1 |
| K | Dcakd |
| K | Dclre1c |
| K | Dcp2 |
| K | Dcp2 |
| K | Dcun1d4 |
| K | Ddx28 |
| K | Ddx49 |
| K | Ddx52 |
| K | Ddx58 |
| K | Ddx6 |
| K | Dennd1a |
| K | Dennd1a |
| K | Dennd1c |
| K | Depdc7 |
| K | Dfna5h |
| K | Dhfr |
| K | Dhrs9 |
| K | Dhx33 |
| K | Dhx38 |
| K | Disc1 |
| K | Disc1 |
| K | Dlgap2 |
| K | Dll1 |
| K | Dnajb5 |
| K | Dnajb9 |
| K | Dnajc16 |
| K | Dnajc18 |
| K | Dnmt1 |
| K | Dnmt3a |
| K | Dock11 |
| K | Dock11 |
| K | Dock2 |
| K | Dock4 |
| K | Dok1 |
| K | Dok3 |
| K | Donson |
| K | Dph2 |
| K | Dpy19l1 |
| K | Dpy19l1 |
| K | Dscr1 |
| K | Dst |
| K | Dtnbp1 |
| K | Dtx4 |
| K | Dus2l |
| K | Dusp16 |
| K | Dusp2 |
| K | Dusp3 |
| K | Dym |
| K | Dync1li1 |
| K | Dync2h1 |
| K | E030010A14Rik |
| K | E130106K03Rik |
| K | E430028B21Rik |
| K | Eaf1 |
| K | Ebi3 |
| K | Ecm1 |
| K | Edem1 |
| K | Edg3 |
| K | Eea1 |
| K | Eef1g |
| K | Efcbp2 |
| K | Efhd2 |
| K | EG238395 |
| K | EG240327 |
| K | EG382448 |
| K | EG408191 |
| K | EG432555 |
| K | EG435337 |
| K | EG545306 |
| K | EG619750 |
| K | EG622339 |
| K | EG629581 |
| K | EG629591 |
| K | EG630499 |
| K | EG634588 |
| K | EG634650 |
| K | EG668139 |
| K | Egr3 |
| K | Eif2ak1 |
| K | Eif2ak2 |
| K | Eif2s2 |
| K | Eif4a1 |
| K | Eif4e2 |
| K | Eif4e2 |
| K | Eif5 |
| K | Ell2 |
| K | Elmod2 |
| K | Emb |
| K | Emilin2 |
| K | Emilin2 |
| K | Emp3 |
| K | Emr1 |
| K | Enc1 |
| K | Endogl1 |
| K | Enpp4 |
| K | Enpp4 |
| K | ENSMUST00000024982 |
| K | ENSMUST00000042143 |
| K | ENSMUST00000043741 |
| K | ENSMUST00000053562 |
| K | ENSMUST00000056717 |
| K | ENSMUST00000073088 |
| K | ENSMUST00000074789 |
| K | ENSMUST00000085410 |
| K | ENSMUST00000092045 |
| K | ENSMUST00000098637 |
| K | ENSMUST00000101511 |
| K | ENSMUST00000103291 |
| K | Entpd3 |
| K | Entpd7 |
| K | Ep300 |
| K | Epha2 |
| K | Ercc1 |
| K | Erg |
| K | Ero1l |
| K | Esrra |
| K | Etf1 |
| K | Ethe1 |
| K | Etnk1 |
| K | Etv6 |
| K | Evi2a |
| K | Evi2b |
| K | Evi2b |
| K | Evl |
| K | Exoc3 |
| K | Exosc2 |
| K | Exosc8 |
| K | Exosc9 |
| K | F10 |
| K | F830004M19Rik |
| K | Fasl |
| K | Fblim1 |
| K | Fbxl14 |
| K | Fbxl5 |
| K | Fbxl5 |
| K | Fbxo42 |
| K | Fbxo6 |
| K | Fbxw11 |
| K | Fbxw17 |
| K | Fbxw2 |
| K | Fcer1g |
| K | Fcgr2b |
| K | Fcgr4 |
| K | Fcho1 |
| K | Fem1c |
| K | Fem1c |
| K | Fes |
| K | Ffar2 |
| K | Fgd3 |
| K | Fgl2 |
| K | Fgr |
| K | Fkbp5 |
| K | Fkbp5 |
| K | Flad1 |
| K | Flnb |
| K | Flnb |
| K | Fmn1 |
| K | Fnbp1l |
| K | Fndc3a |
| K | Fndc3a |
| K | Fos |
| K | Fosb |
| K | Fosl2 |
| K | Foxg1 |
| K | Foxk1 |
| K | Foxk2 |
| K | Foxred2 |
| K | Fpgs |
| K | Fpr-rs2 |
| K | Fry |
| K | Fryl |
| K | Fst |
| K | Ftsj3 |
| K | Furin |
| K | Fxc1 |
| K | G3bp2 |
| K | G6pdx |
| K | Gab2 |
| K | Gadd45b |
| K | Gadd45b |
| K | Gadd45g |
| K | Galns |
| K | Galnt1 |
| K | Galnt10 |
| K | Galnt6 |
| K | Galnt7 |
| K | Gan |
| K | Garnl1 |
| K | Garnl4 |
| K | Gars |
| K | Gas2l3 |
| K | Gas7 |
| K | Gba |
| K | Gbp4 |
| K | Gca |
| K | Gcc2 |
| K | Gch1 |
| K | Gcnt1 |
| K | Gcnt2 |
| K | Gem |
| K | Gfi1 |
| K | Gfpt1 |
| K | Gfpt2 |
| K | Ggps1 |
| K | Gimap9 |
| K | Git1 |
| K | Gla |
| K | Glipr1 |
| K | Glipr2 |
| K | Glis2 |
| K | Glod5 |
| K | Gm1966 |
| K | Gm71 |
| K | Gmfb |
| K | Gmip |
| K | Gmppb |
| K | Gnb1 |
| K | Gnb4 |
| K | Gng12 |
| K | Gng2 |
| K | Gnl3 |
| K | Gnptab |
| K | Gnrh1 |
| K | Golph3 |
| K | Golph4 |
| K | Gorasp2 |
| K | Gosr2 |
| K | Gosr2 |
| K | Gpr119 |
| K | Gpr160 |
| K | Gpr65 |
| K | Gprk6 |
| K | Gramd3 |
| K | Grap2 |
| K | Grin3b |
| K | Grina |
| K | Gsg2 |
| K | Gtf2f1 |
| K | Gtf2f2 |
| K | Gtf2f2 |
| K | Gtpbp2 |
| K | Gtpbp2 |
| K | Gtpbp8 |
| K | Gtpbp9 |
| K | Gyk |
| K | Gzmb |
| K | H13 |
| K | H2afy |
| K | H2afy |
| K | H2-D1 |
| K | H2-K1 |
| K | H2-K1 |
| K | H2-Q8 |
| K | H2-Q9 |
| K | H2-T22 |
| K | H2-T23 |
| K | H3f3b |
| K | H3f3b |
| K | Hal |
| K | Hand2 |
| K | Has1 |
| K | Has2 |
| K | Hat1 |
| K | Havcr2 |
| K | Hbegf |
| K | Hck |
| K | Hcls1 |
| K | Hcst |
| K | Hdh |
| K | Heatr5a |
| K | Herc5 |
| K | Hhex |
| K | Hhex |
| K | Hif1a |
| K | Hipk2 |
| K | Hivep3 |
| K | Hk1 |
| K | Hmbox1 |
| K | Hmgcr |
| K | Hmha1 |
| K | Hnrpa1 |
| K | Hnrpd |
| K | Hnrpdl |
| K | Hpdl |
| K | Hpse |
| K | Hpxn |
| K | Hsd17b7 |
| K | Hsd17b7 |
| K | Hsh2d |
| K | Hspbap1 |
| K | Ibrdc1 |
| K | Icam1 |
| K | Icam5 |
| K | Icosl |
| K | Id2 |
| K | Id2 |
| K | Ier2 |
| K | Ier3 |
| K | Ier5 |
| K | Ier5l |
| K | Ifi203 |
| K | Ifi204 |
| K | Ifi204 |
| K | Ifi30 |
| K | Ifi35 |
| K | Ifit2 |
| K | Ifitm1 |
| K | Ifitm2 |
| K | Ifitm2 |
| K | Ifitm7 |
| K | Ifnar2 |
| K | Ifng |
| K | Ifrg15 |
| K | Igf1 |
| K | Igf2bp2 |
| K | Igsf6 |
| K | Igsf9 |
| K | Ihpk1 |
| K | Ihpk1 |
| K | Ikzf1 |
| K | Ikzf3 |
| K | Il11 |
| K | Il12rb1 |
| K | Il13ra1 |
| K | Il15ra |
| K | Il17ra |
| K | Il17ra |
| K | Il18bp |
| K | Il18bp |
| K | Il18rap |
| K | Il1r2 |
| K | Il1r2 |
| K | Il20rb |
| K | Il24 |
| K | Il2rb |
| K | Il2rg |
| K | Il4ra |
| K | Ing4 |
| K | Inhba |
| K | Inhbb |
| K | Inpp5d |
| K | Ipo4 |
| K | Irf1 |
| K | Irf2 |
| K | Irf5 |
| K | Irgm |
| K | Irgm |
| K | Isg20 |
| K | Isgf3g |
| K | Itga3 |
| K | Itga3 |
| K | Itga4 |
| K | Itga4 |
| K | Itgal |
| K | Itgav |
| K | Itgax |
| K | Itgb2 |
| K | Itgb7 |
| K | Itpa |
| K | Itpkc |
| K | Ivns1abp |
| K | Jak2 |
| K | Jarid1b |
| K | Jmjd3 |
| K | Josd3 |
| K | Josd3 |
| K | Josd3 |
| K | Junb |
| K | Kars |
| K | Katna1 |
| K | Kbtbd2 |
| K | Kif16b |
| K | Kif1b |
| K | Kif5c |
| K | Klhl15 |
| K | Klhl6 |
| K | Klhl6 |
| K | Klk1 |
| K | Klk1 |
| K | Klra21 |
| K | Klra23 |
| K | Kmo |
| K | Kpna3 |
| K | Kpna3 |
| K | Kpna4 |
| K | Kpnb1 |
| K | Kremen1 |
| K | Krt15 |
| K | Krt17 |
| K | Krt18 |
| K | Krt85 |
| K | Lactb |
| K | Lair1 |
| K | Lair1 |
| K | Lamp2 |
| K | Lap3 |
| K | Lap3 |
| K | Larp1 |
| K | Larp1 |
| K | Lass6 |
| K | Lats1 |
| K | Lck |
| K | Ldlrap1 |
| K | Leprotl1 |
| K | Leprotl1 |
| K | Lgals1 |
| K | Lgals3bp |
| K | Lgals8 |
| K | Lhfpl2 |
| K | Lilrb4 |
| K | Lincr |
| K | Lipg |
| K | Litaf |
| K | Lmna |
| K | Lmna |
| K | Lmna |
| K | Lmnb1 |
| K | Lmnb2 |
| K | Lnpep |
| K | LOC194985 |
| K | LOC219106 |
| K | LOC545342 |
| K | LOC546100 |
| K | LOC667370 |
| K | LOC670106 |
| K | LOC671973 |
| K | LOC672716 |
| K | LOC673370 |
| K | Lonrf3 |
| K | Loxl1 |
| K | Lpp |
| K | Lrba |
| K | Lrch1 |
| K | Lrch4 |
| K | Lrg1 |
| K | Lrig2 |
| K | Lrp1 |
| K | Lrp12 |
| K | Lrrc4 |
| K | Lrrc59 |
| K | Lrrc8c |
| K | Lsm12 |
| K | Ltb4r1 |
| K | Ly6e |
| K | Ly96 |
| K | Lyl1 |
| K | Lyn |
| K | Lysmd3 |
| K | Mad2l2 |
| K | Maf |
| K | Mafb |
| K | Maff |
| K | Mafk |
| K | Malt1 |
| K | Malt1 |
| K | Map2k1 |
| K | Map2k3 |
| K | Map3k14 |
| K | Map4k1 |
| K | Map4k4 |
| K | Mapk11 |
| K | Mapk6 |
| K | Mapk7 |
| K | Mapkapk2 |
| K | Mapkapk3 |
| K | Marcksl1 |
| K | Marcksl1 |
| K | Mare |
| K | Mars |
| K | Mbc2 |
| K | Mbd2 |
| K | Mbd2 |
| K | Mcl1 |
| K | Mcm9 |
| K | Mdk |
| K | Mdm4 |
| K | Mefv |
| K | Megf9 |
| K | Mgat2 |
| K | Mgat4a |
| K | Midn |
| K | Mif |
| K | Mitd1 |
| K | Mitd1 |
| K | Mizf |
| K | Mki67ip |
| K | Mkrn1 |
| K | Mlkl |
| K | Mll3 |
| K | Mllt1 |
| K | Mllt11 |
| K | Mmp19 |
| K | Mmp3 |
| K | Mms19l |
| K | Mobkl2a |
| K | Mobkl2c |
| K | Mocs1 |
| K | Morc3 |
| K | Mospd4 |
| K | Mov10 |
| K | Mpeg1 |
| K | Mpo |
| K | Mrgprg |
| K | Mrpl17 |
| K | Mrpl30 |
| K | Mrpl35 |
| K | Mrpl40 |
| K | Mrpl40 |
| K | Mrps22 |
| K | Mrto4 |
| K | Ms4a4b |
| K | Ms4a4d |
| K | Ms4a4d |
| K | Ms4a6c |
| K | Msh3 |
| K | Msn |
| K | Mt1 |
| K | Mt1 |
| K | Mtf1 |
| K | Mthfd2 |
| K | Mtpn |
| K | Mx1 |
| K | Mxd1 |
| K | Mybbp1a |
| K | Myc |
| K | Myc |
| K | Myo10 |
| K | Myo1f |
| K | Myo5a |
| K | Myohd1 |
| K | NAP000727-001 |
| K | NAP007796-001 |
| K | NAP019557-001 |
| K | NAP022641-001 |
| K | NAP025806-1 |
| K | NAP037326-1 |
| K | NAP043943-1 |
| K | NAP046244-1 |
| K | NAP049188-1 |
| K | NAP050738-1 |
| K | NAP055718-1 |
| K | NAP057030-1 |
| K | NAP058998-1 |
| K | NAP061805-1 |
| K | NAP066348-1 |
| K | NAP070973-1 |
| K | NAP097934-001 |
| K | NAP099819-001 |
| K | NAP102462-1 |
| K | NAP107236-1 |
| K | NAP113293-1 |
| K | NAP120816-001 |
| K | NAP122876-1 |
| K | Nasp |
| K | Nat13 |
| K | Nat5 |
| K | Nat5 |
| K | Ncf1 |
| K | Ncf4 |
| K | Ncl |
| K | Ncoa3 |
| K | Nedd9 |
| K | Nek1 |
| K | Nek6 |
| K | Neud4 |
| K | Nfam1 |
| K | Nfatc3 |
| K | Nfil3 |
| K | Nfil3 |
| K | Nfkb2 |
| K | Nfkbib |
| K | Nfkbiz |
| K | Nfs1 |
| K | Nfxl1 |
| K | Nip7 |
| K | Nipa2 |
| K | Nkg7 |
| K | Nkg7 |
| K | Nkiras2 |
| K | Nktr |
| K | Nle1 |
| K | Nln |
| K | Nlrp3 |
| K | Nmt1 |
| K | Nmt2 |
| K | Noc3l |
| K | Noc4l |
| K | Nol12 |
| K | Nol5 |
| K | Nol6 |
| K | Nola2 |
| K | Nos3 |
| K | Nras |
| K | Nrg1 |
| K | Nrip3 |
| K | Nsf |
| K | Nt5c3 |
| K | Nubp1 |
| K | Nudcd1 |
| K | Nudt21 |
| K | Nudt22 |
| K | Nufip1 |
| K | Nufip1 |
| K | Nup155 |
| K | Nup50 |
| K | Nup54 |
| K | Nup98 |
| K | Nupl2 |
| K | Nupr1 |
| K | Nvl |
| K | Nxn |
| K | Nxn |
| K | Oaf |
| K | Oas1a |
| K | Oas3 |
| K | Oasl2 |
| K | Obfc2a |
| K | Odf2 |
| K | Ogfr |
| K | Ogfrl1 |
| K | Olfm1 |
| K | Olfr433 |
| K | Olfr53 |
| K | Olfr741 |
| K | Omp |
| K | Oraov1 |
| K | ORF5 |
| K | Orm1 |
| K | Osgin1 |
| K | Osmr |
| K | Otof |
| K | OTTMUSG00000015282 |
| K | Otub1 |
| K | Otub2 |
| K | Otud3 |
| K | Otud7b |
| K | Oxsm |
| K | Oxsr1 |
| K | P2ry14 |
| K | P2ry6 |
| K | Palb2 |
| K | Panx1 |
| K | Pappa |
| K | Pard6b |
| K | Pard6b |
| K | Parl |
| K | Parp14 |
| K | Parp14 |
| K | Parp3 |
| K | Parp8 |
| K | Pbef1 |
| K | Pbef1 |
| K | Pcgf5 |
| K | Pde1a |
| K | Pde1a |
| K | Pdlim7 |
| K | Pdlim7 |
| K | Pdss1 |
| K | Pdzrn3 |
| K | Pecam1 |
| K | Peli1 |
| K | Perq1 |
| K | Pex1 |
| K | Pex11a |
| K | Pfdn4 |
| K | Pfkfb2 |
| K | Pfkfb3 |
| K | Pfkp |
| K | Pfn1 |
| K | Pfpl |
| K | Pftk1 |
| K | Pgd |
| K | Phc2 |
| K | Phca |
| K | Phf11 |
| K | Phf15 |
| K | Phf15 |
| K | Phf23 |
| K | Phf3 |
| K | Phf6 |
| K | Phlda1 |
| K | Phr1 |
| K | Picalm |
| K | Pign |
| K | Pigw |
| K | Pik3cd |
| K | Pilrb1 |
| K | Pim1 |
| K | Pim2 |
| K | Pim2 |
| K | Pip5k1a |
| K | Pip5k1a |
| K | Pitpna |
| K | Pitpnm1 |
| K | Pitpnm2 |
| K | Pla1a |
| K | Pla2g4c |
| K | Plaa |
| K | Plat |
| K | Plat |
| K | Plau |
| K | Plaur |
| K | Plaur |
| K | Plcg2 |
| K | Pld4 |
| K | Plec1 |
| K | Plek |
| K | Plek |
| K | Plekhb2 |
| K | Plekho1 |
| K | Plekhq1 |
| K | Plk2 |
| K | Plk3 |
| K | Plod3 |
| K | Plscr3 |
| K | Pmf1 |
| K | Pno1 |
| K | Pnp |
| K | Pnpla7 |
| K | Pnpt1 |
| K | Pnpt1 |
| K | Pofut2 |
| K | Pola2 |
| K | Pols |
| K | Pom121 |
| K | Ppa1 |
| K | Ppif |
| K | Ppp1r12c |
| K | Ppp1r15b |
| K | Ppp2r5c |
| K | Ppp3cc |
| K | Ppp3cc |
| K | Ppt1 |
| K | Pram1 |
| K | Prdx5 |
| K | Prickle2 |
| K | Prkaa1 |
| K | Prkab2 |
| K | Prkcb1 |
| K | Prkcb1 |
| K | Prkcd |
| K | Prkcd |
| K | Prmt3 |
| K | Procr |
| K | Prpf38a |
| K | Prr16 |
| K | Prrc1 |
| K | Prrx1 |
| K | Prrx1 |
| K | Prrx1 |
| K | Prtn3 |
| K | Pscd4 |
| K | Psd4 |
| K | Psma4 |
| K | Psmb10 |
| K | Psmb8 |
| K | Psmc4 |
| K | Psmd12 |
| K | Psmd12 |
| K | Psmd12 |
| K | Psmd14 |
| K | Psmd3 |
| K | Psmd7 |
| K | Psmd8 |
| K | Psme1 |
| K | Psme2 |
| K | Psme3 |
| K | Pstpip1 |
| K | Ptcd1 |
| K | Ptgir |
| K | Ptp4a1 |
| K | Ptpn1 |
| K | Ptpn14 |
| K | Ptpn2 |
| K | Ptpn22 |
| K | Ptpn6 |
| K | Ptpn6 |
| K | Ptpn7 |
| K | Ptprc |
| K | Ptprcap |
| K | Pus1 |
| K | Pus7 |
| K | Pvr |
| K | Pvr |
| K | Pvr |
| K | Pvrl2 |
| K | Pvrl4 |
| K | Pwp2 |
| K | Pwp2 |
| K | Pxn |
| K | Pycard |
| K | Pycr1 |
| K | Qscn6 |
| K | Qscn6 |
| K | Rab10 |
| K | Rab10 |
| K | Rab11fip5 |
| K | Rab12 |
| K | Rab20 |
| K | Rab22a |
| K | Rab37 |
| K | Rab3il1 |
| K | Rab43 |
| K | Rab43 |
| K | Rab5c |
| K | Rab8b |
| K | Rabl3 |
| K | Ramp2 |
| K | Ranbp3 |
| K | Ranbp5 |
| K | Ranbp5 |
| K | Rap2c |
| K | Rars |
| K | Rasd1 |
| K | Rasgef1b |
| K | Raver1 |
| K | Raver1 |
| K | Rb1cc1 |
| K | Rbl1 |
| K | Rbm13 |
| K | Rbm18 |
| K | Rbm19 |
| K | Rbm22 |
| K | Rbm34 |
| K | Rbm43 |
| K | Rbm9 |
| K | Rcc2 |
| K | Rcor3 |
| K | Reep3 |
| K | Refbp2 |
| K | Relb |
| K | Retnlg |
| K | Rexo1 |
| K | Rffl |
| K | Rg9mtd2 |
| K | Rgl1 |
| K | Rgs19 |
| K | Rhbdf2 |
| K | Rhbdf2 |
| K | Rhcg |
| K | Rhoc |
| K | Rhog |
| K | Rhoh |
| K | Rhou |
| K | Riok3 |
| K | Ripk1 |
| K | Ripk3 |
| K | Rmnd5a |
| K | Rnase6 |
| K | Rnd3 |
| K | Rnd3 |
| K | Rnf12 |
| K | Rnf135 |
| K | Rnf139 |
| K | Rnf149 |
| K | Rnf149 |
| K | Rnf157 |
| K | Rnf183 |
| K | Rnf213 |
| K | Rnf31 |
| K | Rnf31 |
| K | Rnh1 |
| K | Rod1 |
| K | RP23-336J1.4 |
| K | Rp2h |
| K | Rpl7l1 |
| K | Rpo1-4 |
| K | Rpp38 |
| K | Rps6ka3 |
| K | Rrad |
| K | Rras2 |
| K | Rras2 |
| K | Rrbp1 |
| K | Rrbp1 |
| K | Rrbp1 |
| K | Rrp9 |
| K | Rsad2 |
| K | Rsad2 |
| K | Rufy3 |
| K | Rufy3 |
| K | Runx2 |
| K | Runx3 |
| K | S100a9 |
| K | Saa1 |
| K | Saa3 |
| K | Saal1 |
| K | Samd8 |
| K | Samd9l |
| K | Samhd1 |
| K | Samhd1 |
| K | Sap30 |
| K | Sap30bp |
| K | Sar1a |
| K | Sars |
| K | Sass6 |
| K | Sat1 |
| K | Sbno2 |
| K | Scamp2 |
| K | Scamp4 |
| K | Scand1 |
| K | Sclt1 |
| K | Sco1 |
| K | Scotin |
| K | Sct |
| K | Sdad1 |
| K | Sdc3 |
| K | Sdcbp2 |
| K | Sdk1 |
| K | Seh1l |
| K | Sell |
| K | Selp |
| K | Selplg |
| K | Sema4a |
| K | Sema4c |
| K | Sema4d |
| K | Sema6b |
| K | Sema7a |
| K | Senp5 |
| K | Serpine1 |
| K | Sertad1 |
| K | Sesn2 |
| K | Set |
| K | Setd8 |
| K | Sfmbt1 |
| K | Sfrp1 |
| K | Sfrs2 |
| K | Sgcb |
| K | Sgk |
| K | Sgk3 |
| K | Sgms1 |
| K | Sgpl1 |
| K | Sh2b3 |
| K | Sh2d1a |
| K | Sh3d19 |
| K | Sh3glb1 |
| K | Sh3rf1 |
| K | Siglec1 |
| K | Sirpa |
| K | Sirpa |
| K | Slamf6 |
| K | Slamf7 |
| K | Slamf9 |
| K | Slc11a1 |
| K | Slc12a9 |
| K | Slc16a10 |
| K | Slc16a10 |
| K | Slc16a3 |
| K | Slc16a6 |
| K | Slc20a1 |
| K | Slc20a2 |
| K | Slc23a3 |
| K | Slc25a20 |
| K | Slc25a25 |
| K | Slc25a30 |
| K | Slc25a37 |
| K | Slc25a43 |
| K | Slc25a43 |
| K | Slc26a2 |
| K | Slc26a4 |
| K | Slc2a1 |
| K | Slc2a3 |
| K | Slc30a1 |
| K | Slc30a7 |
| K | Slc35c2 |
| K | Slc35d2 |
| K | Slc39a1 |
| K | Slc39a14 |
| K | Slc3a2 |
| K | Slc6a13 |
| K | Slc7a6 |
| K | Slc7a7 |
| K | Slc8a1 |
| K | Slco4a1 |
| K | Slfn2 |
| K | Slfn3 |
| K | Slfn5 |
| K | Smap1l |
| K | Smcr8 |
| K | Smek1 |
| K | Smg7 |
| K | Smn1 |
| K | Smndc1 |
| K | Smpd3 |
| K | Smpd3 |
| K | Smpdl3b |
| K | Smurf1 |
| K | Snag1 |
| K | Snai1 |
| K | Snap29 |
| K | Snapc3 |
| K | Snrpd1 |
| K | Snx10 |
| K | Snx2 |
| K | Snx5 |
| K | Snx8 |
| K | Socs1 |
| K | Socs3 |
| K | Sod2 |
| K | Sp100 |
| K | Sp100 |
| K | Sp100 |
| K | Sp4 |
| K | Spag9 |
| K | Spata13 |
| K | Spata2 |
| K | Spata20 |
| K | Sphk1 |
| K | Sphk1 |
| K | Spic |
| K | Spn |
| K | Spn |
| K | Spna2 |
| K | Spry2 |
| K | Spsb1 |
| K | Sptlc2 |
| K | Sptlc2 |
| K | Spty2d1 |
| K | Srcrb4d |
| K | Srfbp1 |
| K | Srgn |
| K | Srm |
| K | Srxn1 |
| K | St3gal4 |
| K | St6galnac4 |
| K | St7 |
| K | Stab1 |
| K | Stac2 |
| K | Stac3 |
| K | Stam2 |
| K | Stard3 |
| K | Stat1 |
| K | Stat1 |
| K | Stat2 |
| K | Stat3 |
| K | Stat4 |
| K | Steap4 |
| K | Stk19 |
| K | Stk19 |
| K | Stk19 |
| K | Stk40 |
| K | Stoml1 |
| K | Stoml1 |
| K | Stx16 |
| K | Stx3 |
| K | Stxbp5 |
| K | Surf5 |
| K | Susd3 |
| K | Sv2a |
| K | Svil |
| K | Syk |
| K | Syncrip |
| K | Synj1 |
| K | Synj1 |
| K | Tacc2 |
| K | Taf4b |
| K | Taf5 |
| K | Taf5 |
| K | Taf6l |
| K | Taf7 |
| K | Tbc1d1 |
| K | Tbc1d9 |
| K | Tbc1d9 |
| K | Tbc1d9b |
| K | Tbk1 |
| K | Tbl1xr1 |
| K | Tbl3 |
| K | Tbrg4 |
| K | TC1628823 |
| K | TC1634227 |
| K | TC1645771 |
| K | TC1651824 |
| K | TC1653266 |
| K | TC1677080 |
| K | TC1688109 |
| K | TC1709674 |
| K | TC1717672 |
| K | Tceb3 |
| K | Tceb3 |
| K | Tcfec |
| K | Tcirg1 |
| K | Tcp11l1 |
| K | Tdrd7 |
| K | Tead1 |
| K | Tec |
| K | Tep1 |
| K | Tgfbr1 |
| K | Tgs1 |
| K | Thap6 |
| K | Thbs1 |
| K | Thbs1 |
| K | Thbs1 |
| K | Thoc6 |
| K | Thrap2 |
| K | Thumpd3 |
| K | Thyn1 |
| K | Timeless |
| K | Timm10 |
| K | Tiparp |
| K | Tjp1 |
| K | Tle3 |
| K | Tlk2 |
| K | Tlk2 |
| K | Tlr3 |
| K | Tlr7 |
| K | Tm6sf1 |
| K | Tmbim4 |
| K | Tmco4 |
| K | Tmem106a |
| K | Tmem127 |
| K | Tmem128 |
| K | Tmem142a |
| K | Tmem167 |
| K | Tmem185b |
| K | Tmem37 |
| K | Tmem40 |
| K | Tmem40 |
| K | Tmem49 |
| K | Tmem67 |
| K | Tmepai |
| K | Tnc |
| K | Tnfaip3 |
| K | Tnfaip8l2 |
| K | Tnfrsf12a |
| K | Tnfrsf14 |
| K | Tnfrsf1b |
| K | Tnfrsf4 |
| K | Tnfsf10 |
| K | Tnip1 |
| K | Tnks1bp1 |
| K | Tnpo2 |
| K | Tnrc6b |
| K | Tomm20 |
| K | Tomm70a |
| K | Tor1aip1 |
| K | Tor3a |
| K | Tpcn2 |
| K | Tpd52 |
| K | Tpd52 |
| K | Trabd |
| K | Trafd1 |
| K | Trafd1 |
| K | Tram1 |
| K | Trem2 |
| K | Trem3 |
| K | Trex1 |
| K | Trib1 |
| K | Trim14 |
| K | Trim16 |
| K | Trim21 |
| K | Trim26 |
| K | Trim27 |
| K | Trim30 |
| K | Tsc22d2 |
| K | Tspan4 |
| K | Tspo |
| K | Tspo |
| K | Tsr1 |
| K | Tssc1 |
| K | Ttbk2 |
| K | Ttc7 |
| K | Ttc9c |
| K | Ttc9c |
| K | Ttf2 |
| K | Ttll4 |
| K | Ttll4 |
| K | Tubb6 |
| K | Tuft1 |
| K | Txndc5 |
| K | Txnrd1 |
| K | Tyk2 |
| K | Tyki |
| K | Tyrobp |
| K | U11274 |
| K | Ubap1 |
| K | Ubap1 |
| K | Ubap2l |
| K | Ube1x |
| K | Ube2d1 |
| K | Ube2f |
| K | Ube2j1 |
| K | Ube2l6 |
| K | Ube2m |
| K | Ube2m |
| K | Ubqln1 |
| K | Ubr2 |
| K | Ubtd1 |
| K | Ubtd2 |
| K | Ubtd2 |
| K | Uchl4 |
| K | Uck2 |
| K | Uck2 |
| K | Ugcg |
| K | Ugcg |
| K | Ugcg |
| K | Umps |
| K | Unc93b1 |
| K | Usp16 |
| K | Usp25 |
| K | Usp33 |
| K | Usp42 |
| K | Utp18 |
| K | Utrn |
| K | Vac14 |
| K | Vasp |
| K | Vasp |
| K | Vav1 |
| K | Vav1 |
| K | Vcan |
| K | Vcpip1 |
| K | Vcpip1 |
| K | Vcpip1 |
| K | Vcpip1 |
| K | Vps33a |
| K | Vps37b |
| K | Vps54 |
| K | Vrk2 |
| K | Vta1 |
| K | Vti1a |
| K | W15861 |
| K | Wars2 |
| K | Was |
| K | Was |
| K | Wdfy2 |
| K | Wdr33 |
| K | Wdr4 |
| K | Wdr40c |
| K | Wdr41 |
| K | Wdr5 |
| K | Wdr60 |
| K | Wdr73 |
| K | Wibg |
| K | Wipf1 |
| K | Wisp2 |
| K | Wsb1 |
| K | Wwp2 |
| K | Wwp2 |
| K | Xdh |
| K | Xrn2 |
| K | Yars |
| K | Ykt6 |
| K | Ykt6 |
| K | Ypel5 |
| K | Ythdf1 |
| K | Zap70 |
| K | Zbtb7a |
| K | Zbtb7b |
| K | Zc3h12a |
| K | Zc3h12c |
| K | Zc3h15 |
| K | Zc3hav1 |
| K | Zcchc11 |
| K | Zcchc2 |
| K | Zcchc2 |
| K | Zdhhc24 |
| K | Zfand5 |
| K | Zfp143 |
| K | Zfp148 |
| K | Zfp26 |
| K | Zfp26 |
| K | Zfp281 |
| K | Zfp296 |
| K | Zfp36 |
| K | Zfp36 |
| K | Zfp36l2 |
| K | Zfp384 |
| K | Zfp385 |
| K | Zfp629 |
| K | Zfp809 |
| K | Zkscan3 |
| K | Zkscan3 |
| K | Znhit1 |
| K | Zwint |
| L | 2-Mar |
| L | 1110028C15Rik |
| L | 1110059E24Rik |
| L | 1110067D22Rik |
| L | 1190003M12Rik |
| L | 1700034H14Rik |
| L | 1810020D17Rik |
| L | 1810030O07Rik |
| L | 1810063B07Rik |
| L | 2210010B09Rik |
| L | 2210021J22Rik |
| L | 2210408K08 |
| L | 2300002D11Rik |
| L | 2310001A20Rik |
| L | 2310002J21Rik |
| L | 2310010J17Rik |
| L | 2310016C16Rik |
| L | 2310033P09Rik |
| L | 2310061C15Rik |
| L | 2410005O16Rik |
| L | 2410127L17Rik |
| L | 2610110G12Rik |
| L | 2700081O15Rik |
| L | 2700097O09Rik |
| L | 2810417J12Rik |
| L | 3110001I20Rik |
| L | 3110001I20Rik |
| L | 3110001I20Rik |
| L | 4732457N14 |
| L | 4832420A03Rik |
| L | 4833428M15Rik |
| L | 4833442J19Rik |
| L | 4930502E18Rik |
| L | 4930504E06Rik |
| L | 4930523C07Rik |
| L | 4930570C03Rik |
| L | 4930571C24Rik |
| L | 4931406P16Rik |
| L | 4932416N17Rik |
| L | 5330426P16Rik |
| L | 5730593F17Rik |
| L | 6330416L11Rik |
| L | 6330419J24Rik |
| L | 6720489N17Rik |
| L | 9430028L06Rik |
| L | 9530004P13Rik |
| L | 9630025I21Rik |
| L | 9630041N07Rik |
| L | A_52_P517668 |
| L | A230062I15Rik |
| L | A230092J17Rik |
| L | A430090L17Rik |
| L | A430107O13Rik |
| L | A630007B06Rik |
| L | A930001N09Rik |
| L | A930041I02Rik |
| L | Aasdhppt |
| L | Abcb9 |
| L | Abcd3 |
| L | Ablim1 |
| L | Ablim2 |
| L | Abtb1 |
| L | Acot11 |
| L | Acvrl1 |
| L | Adcy2 |
| L | Adcy3 |
| L | Add3 |
| L | Agtr1a |
| L | Ahsa2 |
| L | AI314976 |
| L | AI314976 |
| L | AJ237917 |
| L | AK005483 |
| L | AK031532 |
| L | AK031552 |
| L | AK033087 |
| L | AK034320 |
| L | AK034805 |
| L | AK035307 |
| L | AK037381 |
| L | AK038076 |
| L | AK038230 |
| L | AK041851 |
| L | AK043497 |
| L | AK044903 |
| L | AK045692 |
| L | AK047905 |
| L | AK048751 |
| L | AK051949 |
| L | AK052617 |
| L | AK054073 |
| L | AK078794 |
| L | AK079007 |
| L | AK079264 |
| L | AK080781 |
| L | AK085332 |
| L | AK086969 |
| L | AK087084 |
| L | AK087326 |
| L | Alcam |
| L | Aldh1l1 |
| L | Alg2 |
| L | Als2cr4 |
| L | Anapc1 |
| L | Ang1 |
| L | Ang2 |
| L | Ang4 |
| L | Ankrd24 |
| L | Arhgef12 |
| L | Armc9 |
| L | Arrb1 |
| L | Atbf1 |
| L | Atbf1 |
| L | Atg4c |
| L | Atp1b2 |
| L | Atp9a |
| L | AW124722 |
| L | AW215868 |
| L | Azin1 |
| L | B330016D10Rik |
| L | Bace2 |
| L | BB333095 |
| L | BC008155 |
| L | BC011209 |
| L | BC024868 |
| L | BC038613 |
| L | BC048507 |
| L | Bcam |
| L | BI100969 |
| L | BI408134 |
| L | Bmp1 |
| L | Bmpr2 |
| L | Bmyc |
| L | Brd3 |
| L | Bsg |
| L | Btbd2 |
| L | C030044C12Rik |
| L | Cab39l |
| L | Calm1 |
| L | Calm1 |
| L | Camta1 |
| L | Caprin2 |
| L | Carkl |
| L | Cars2 |
| L | Casz1 |
| L | Casz1 |
| L | Cbx3 |
| L | Ccdc85b |
| L | Ccny |
| L | Ccr6 |
| L | Cd300lg |
| L | Cd59a |
| L | Cdc14b |
| L | Cdc2l6 |
| L | Cdc42bpa |
| L | Cdh11 |
| L | Cdh11 |
| L | Cdkl2 |
| L | Cdkn1b |
| L | Cdkn2c |
| L | Cdkn2c |
| L | Cebpa |
| L | Cep70 |
| L | Cfh |
| L | Cfh |
| L | Cfh |
| L | Cfh |
| L | Cgnl1 |
| L | Chd6 |
| L | Ches1 |
| L | Chia |
| L | Chst1 |
| L | Chst2 |
| L | Chst8 |
| L | CJ288729 |
| L | Clk4 |
| L | Cobll1 |
| L | Cobll1 |
| L | Col13a1 |
| L | Col4a6 |
| L | Colec12 |
| L | Colec12 |
| L | Coq7 |
| L | Coq7 |
| L | Cox4i2 |
| L | Cpd |
| L | Cr2 |
| L | Crebl2 |
| L | Crim1 |
| L | Ctnnb1 |
| L | Cyp2d10 |
| L | D030011O10Rik |
| L | D130059P03Rik |
| L | D130061D10Rik |
| L | D14Abb1e |
| L | D14Ertd500e |
| L | D230004J03Rik |
| L | D430007A19Rik |
| L | D5Ertd593e |
| L | D630040G17Rik |
| L | D6Wsu176e |
| L | D730040F13Rik |
| L | D930030O05Rik |
| L | D9Ertd402e |
| L | Dapk1 |
| L | Dbp |
| L | Ddx5 |
| L | Deb1 |
| L | Dirc2 |
| L | Dkk3 |
| L | Dmpk |
| L | Dnalc1 |
| L | Dock1 |
| L | Dock1 |
| L | Dock4 |
| L | Dock4 |
| L | Dock4 |
| L | Dock6 |
| L | Dohh |
| L | Dpysl2 |
| L | Dtnb |
| L | DV651670 |
| L | Dynll1 |
| L | Dyrk1b |
| L | E030026E10Rik |
| L | Ece1 |
| L | Echdc3 |
| L | Ednra |
| L | Efna1 |
| L | Efnb1 |
| L | Eif2c1 |
| L | ENSMUSG00000071543 |
| L | ENSMUST00000035340 |
| L | ENSMUST00000069187 |
| L | ENSMUST00000072587 |
| L | Erbb3 |
| L | Ercc5 |
| L | Fancb |
| L | Fancc |
| L | Farp2 |
| L | Fbln1 |
| L | Fbxl12 |
| L | Fbxo30 |
| L | Fcho2 |
| L | Fdx1 |
| L | Fdx1 |
| L | Fech |
| L | Fgd5 |
| L | Fibp |
| L | Foxo1 |
| L | Foxo6 |
| L | Frmd6 |
| L | Frmd6 |
| L | Frmd6 |
| L | Fusip1 |
| L | Fzd6 |
| L | Fzd7 |
| L | Fzd8 |
| L | Gabbr1 |
| L | Galntl4 |
| L | Gata2 |
| L | Gata2 |
| L | Gata3 |
| L | Gfra2 |
| L | Glg1 |
| L | Glg1 |
| L | Glis2 |
| L | Glul |
| L | Gm684 |
| L | Gmcl1 |
| L | Gnpda2 |
| L | Gpihbp1 |
| L | Gpm6a |
| L | Gpm6b |
| L | Gpsm1 |
| L | Grap |
| L | Grin2c |
| L | Hdac8 |
| L | Hectd3 |
| L | Heg1 |
| L | Hhip |
| L | Hint2 |
| L | Hlf |
| L | Hoxa1 |
| L | Hoxa2 |
| L | Hoxa7 |
| L | Hsp90b1 |
| L | Hspa12a |
| L | Hspa4 |
| L | Ick |
| L | Id3 |
| L | Igf1r |
| L | Igf1r |
| L | Igf2 |
| L | Igfbp2 |
| L | Igsf3 |
| L | Inpp4b |
| L | Irf2bp2 |
| L | Irs1 |
| L | Irx1 |
| L | Irx1 |
| L | Jmy |
| L | Jup |
| L | Kcnb1 |
| L | Kctd1 |
| L | Kctd15 |
| L | Kctd7 |
| L | Kif13a |
| L | Kif13b |
| L | Kif1b |
| L | Kifap3 |
| L | Kitl |
| L | Kras |
| L | Krt23 |
| L | L01776 |
| L | L01776 |
| L | l7Rn6 |
| L | Lama3 |
| L | Lama4 |
| L | Lamb3 |
| L | Lass4 |
| L | Lbh |
| L | Ldb2 |
| L | Lifr |
| L | Lmo7 |
| L | LOC669793 |
| L | Lphn1 |
| L | Lrdd |
| L | M77174 |
| L | Maml2 |
| L | Mapk8ip1 |
| L | Mapre3 |
| L | Mast1 |
| L | Mat2a |
| L | Mccc2 |
| L | Mcf2l |
| L | Me2 |
| L | Meis1 |
| L | Meox2 |
| L | Met |
| L | Mfap2 |
| L | Mfap4 |
| L | Mfhas1 |
| L | Mgll |
| L | Mllt3 |
| L | Mllt3 |
| L | Mme |
| L | Mpdz |
| L | Mpdz |
| L | Mpzl1 |
| L | Mrg1 |
| L | Mrg1 |
| L | Msr2 |
| L | Msra |
| L | Mthfd1 |
| L | Mum1l1 |
| L | Mxd4 |
| L | Myo1b |
| L | NAP014312-001 |
| L | NAP026388-1 |
| L | NAP046120-1 |
| L | Nat6 |
| L | Nbeal1 |
| L | Nbl1 |
| L | Ncam1 |
| L | Ncapd3 |
| L | Ncor1 |
| L | Nebl |
| L | Nedd4l |
| L | Nfib |
| L | Nid2 |
| L | Npal2 |
| L | Npal2 |
| L | Nphp3 |
| L | Npr1 |
| L | Nr1d1 |
| L | Nr1d2 |
| L | Nr3c2 |
| L | Nrarp |
| L | Nrp2 |
| L | Ntn2l |
| L | Ogdh |
| L | Ogdh |
| L | Olfr414 |
| L | Osbpl3 |
| L | Pabpn1 |
| L | Papss1 |
| L | Pard3b |
| L | Pard6g |
| L | Pbx1 |
| L | Pcdhb6 |
| L | Pcm1 |
| L | Pcnt |
| L | Pcnt |
| L | Pde4d |
| L | Pde7a |
| L | Pdgfa |
| L | Pdlim3 |
| L | Pdlim3 |
| L | Pecam1 |
| L | Peg12 |
| L | Pex10 |
| L | Pex11c |
| L | Pex13 |
| L | Pgm5 |
| L | Phldb1 |
| L | Phpt1 |
| L | Pi4k2b |
| L | Pja2 |
| L | Pkd1 |
| L | Pla2g4f |
| L | Plcl1 |
| L | Pnrc1 |
| L | Polr3g |
| L | Pon3 |
| L | Pon3 |
| L | Ppm1a |
| L | Ppp1r13b |
| L | Ppp1r13b |
| L | Ppp3ca |
| L | Pqlc3 |
| L | Prelp |
| L | Prosapip1 |
| L | Prpf40b |
| L | Prps2 |
| L | Prrt1 |
| L | Pscd3 |
| L | Ptch1 |
| L | Ptpn3 |
| L | Ptprk |
| L | Ptrf |
| L | Pum1 |
| L | Rab4a |
| L | Rab6b |
| L | Rab6b |
| L | Rabgap1l |
| L | Rasal2 |
| L | Rasgrp2 |
| L | Rbm12b |
| L | Recql |
| L | Rgl2 |
| L | Rgmb |
| L | Rhobtb1 |
| L | Rnf123 |
| L | Rnf144 |
| L | Rogdi |
| L | Rprml |
| L | Rsrc1 |
| L | Rutbc1 |
| L | Rutbc3 |
| L | Sall2 |
| L | Scgb1a1 |
| L | Scnn1g |
| L | Scrn1 |
| L | Sdha |
| L | Sema3e |
| L | Sh3d19 |
| L | Shroom4 |
| L | Sirt4 |
| L | Sirt5 |
| L | Skp1a |
| L | Slc1a5 |
| L | Slc22a12 |
| L | Slc2a8 |
| L | Slc6a2 |
| L | Slc6a4 |
| L | Slc6a9 |
| L | Smarca2 |
| L | Sms |
| L | Snca |
| L | Sntb1 |
| L | Snx26 |
| L | Sparc |
| L | Sparcl1 |
| L | Sparcl1 |
| L | Specc1l |
| L | Spg3a |
| L | Spnb2 |
| L | Spnb2 |
| L | Spnb3 |
| L | Sppl3 |
| L | Spr |
| L | Srebf1 |
| L | Srpx |
| L | Ssbp3 |
| L | Ssh2 |
| L | Stard13 |
| L | Stau2 |
| L | Stra13 |
| L | Suv420h2 |
| L | Synj2bp |
| L | Synj2bp |
| L | Tacc2 |
| L | Tbc1d17 |
| L | Tbc1d9b |
| L | Tbcel |
| L | Tbxa2r |
| L | TC1614162 |
| L | TC1632790 |
| L | TC1779078 |
| L | Tcf3 |
| L | Tead2 |
| L | Tef |
| L | Terf2ip |
| L | Tgoln1 |
| L | Thap2 |
| L | Tln2 |
| L | Tmcc2 |
| L | Tmeff2 |
| L | Tmem9 |
| L | Tnik |
| L | Tnrc15 |
| L | Tns1 |
| L | Tns1 |
| L | Tpp1 |
| L | Traf5 |
| L | Trappc2 |
| L | Trappc6a |
| L | Trib2 |
| L | Trim37 |
| L | Trp53bp2 |
| L | Trpc3 |
| L | Trpt1 |
| L | Tspan3 |
| L | Tspan3 |
| L | Tspan6 |
| L | Tspan6 |
| L | Ttc14 |
| L | Ttll7 |
| L | Txnrd3 |
| L | Ube2a |
| L | Ube3b |
| L | Uhrf2 |
| L | Usp20 |
| L | Usp33 |
| L | Utrn |
| L | Utrn |
| L | Utrn |
| L | Vamp2 |
| L | Vegfa |
| L | Vegfb |
| L | Vps13b |
| L | Wdfy3 |
| L | Wdr40a |
| L | Wdr47 |
| L | Wdr51b |
| L | Wipi1 |
| L | Wtip |
| L | Wwp1 |
| L | X99384 |
| L | Zdhhc3 |
| L | Zfp26 |
| L | Zfp291 |
| L | Zfp367 |
| L | Zfp521 |
| L | Zfp532 |
| L | Zfp647 |
| L | Zfp652 |
| L | Zfp75 |
| L | Zhx3 |
| L | Zmym6 |
| L | Zxdc |
