# Supplementary material for: Modeling Host Genetic Regulation of Influenza Pathogenesis in the Collaborative Cross
Source: PLoS Pathog. 2013 Feb 28;9(2):e1003196. doi: 10.1371/journal.ppat.1003196 (PMC3585141; doi:10.1371/journal.ppat.1003196)
Supplement: Table S5 — GO-term enrichment by module. (DOCX) [file ppat.1003196.s011.docx]

| **Table S5. GO-term enrichment by module** | |  |
| --- | --- | --- |
| **Module** | **GO Term** | **By FDR** |
| B | enzyme linked receptor protein signaling pathway | 0.00866964 |
|  | cell adhesion | 0.01643939 |
|  | biological adhesion | 0.01643939 |
|  | anatomical structure morphogenesis | 0.02107207 |
|  | anatomical structure development | 0.02408286 |
| D | cellular macromolecule metabolic process | 5.15E-07 |
|  | gene expression | 1.12E-06 |
|  | macromolecule metabolic process | 2.89E-05 |
|  | chromatin organization | 0.00010248 |
|  | chromosome organization | 0.0002603 |
|  | regulation of gene expression | 0.0005095 |
|  | cellular macromolecule biosynthetic process | 0.00053771 |
|  | nucleobase, nucleoside, nucleotide and nucleic acid metabolic process | 0.00097163 |
|  | chromatin modification | 0.00111599 |
|  | macromolecule biosynthetic process | 0.00115608 |
|  | regulation of macromolecule biosynthetic process | 0.00177174 |
|  | cellular metabolic process | 0.00178579 |
|  | primary metabolic process | 0.00180447 |
|  | regulation of transcription | 0.00469911 |
|  | regulation of cellular biosynthetic process | 0.0068335 |
|  | regulation of biosynthetic process | 0.00704794 |
|  | nitrogen compound metabolic process | 0.01008717 |
|  | regulation of macromolecule metabolic process | 0.0171706 |
|  | transcription | 0.01815349 |
|  | cellular biosynthetic process | 0.01921811 |
|  | RNA metabolic process | 0.02034319 |
|  | regulation of nucleobase, nucleoside, nucleotide and nucleic acid metabolic process | 0.02454501 |
|  | biosynthetic process | 0.02953021 |
|  | regulation of nitrogen compound metabolic process | 0.03064416 |
| F | M phase | 1.21E-15 |
|  | cell cycle phase | 8.59E-15 |
|  | cell cycle process | 6.46E-14 |
|  | M phase of mitotic cell cycle | 8.61E-13 |
|  | nuclear division | 8.61E-13 |
|  | mitosis | 8.61E-13 |
|  | organelle fission | 1.76E-12 |
|  | generation of precursor metabolites and energy | 2.24E-12 |
|  | cell division | 8.73E-12 |
|  | cell cycle | 1.58E-11 |
|  | mitotic cell cycle | 2.03E-11 |
|  | organelle organization | 3.44E-05 |
|  | chromosome segregation | 4.48E-05 |
|  | cellular respiration | 7.83E-05 |
|  | energy derivation by oxidation of organic compounds | 9.46E-05 |
|  | small molecule catabolic process | 0.00018186 |
|  | carbohydrate catabolic process | 0.00020392 |
|  | electron transport chain | 0.00020392 |
|  | hexose metabolic process | 0.00024456 |
|  | DNA metabolic process | 0.0002737 |
|  | monosaccharide metabolic process | 0.0005095 |
|  | DNA replication | 0.0005095 |
|  | cellular carbohydrate catabolic process | 0.0005095 |
|  | tricarboxylic acid cycle | 0.001301 |
|  | acetyl-CoA catabolic process | 0.001301 |
|  | DNA conformation change | 0.00132091 |
|  | glucose metabolic process | 0.00176235 |
|  | oxidation reduction | 0.00287247 |
|  | alcohol catabolic process | 0.00325003 |
|  | acetyl-CoA metabolic process | 0.0068335 |
|  | aerobic respiration | 0.0068335 |
|  | coenzyme catabolic process | 0.0068335 |
|  | DNA packaging | 0.00725141 |
|  | glycolysis | 0.00833389 |
|  | cellular carbohydrate metabolic process | 0.00952369 |
|  | cofactor catabolic process | 0.01230111 |
|  | glucose catabolic process | 0.01236647 |
|  | hexose catabolic process | 0.01236647 |
|  | monosaccharide catabolic process | 0.01236647 |
|  | meiosis | 0.03134898 |
|  | meiotic cell cycle | 0.03134898 |
|  | M phase of meiotic cell cycle | 0.03134898 |
|  | carbohydrate metabolic process | 0.04195691 |
|  | DNA-dependent DNA replication | 0.04195691 |
|  | chromosome condensation | 0.04195691 |
| G | G-protein coupled receptor protein signaling pathway | 5.01E-09 |
|  | cell surface receptor linked signaling pathway | 9.60E-06 |
|  | signaling pathway | 4.39E-05 |
|  | sensory perception of chemical stimulus | 0.00177174 |
|  | sensory perception of smell | 0.00630682 |
|  | signal transduction | 0.00668945 |
|  | signaling | 0.02091363 |
|  | signaling process | 0.03073672 |
|  | signal transmission | 0.03073672 |
|  | sensory perception | 0.0442678 |
| H | ncRNA metabolic process | 1.12E-06 |
|  | RNA processing | 5.96E-05 |
|  | ribonucleoprotein complex biogenesis | 0.0002737 |
|  | tRNA metabolic process | 0.00054221 |
|  | cellular macromolecule metabolic process | 0.00098834 |
|  | ribosome biogenesis | 0.00116326 |
|  | macromolecule metabolic process | 0.00186363 |
|  | translation | 0.00399031 |
|  | cellular metabolic process | 0.00608565 |
|  | ncRNA processing | 0.00739885 |
|  | gene expression | 0.01868173 |
|  | tRNA aminoacylation for protein translation | 0.02408286 |
|  | amino acid activation | 0.02408286 |
|  | tRNA aminoacylation | 0.02408286 |
|  | primary metabolic process | 0.02930784 |
|  | RNA splicing | 0.03314845 |
| I | sensory perception of smell | 0.02380626 |
|  | neurological system process | 0.04279572 |
| K | immune response | 8.03E-15 |
|  | immune system process | 1.49E-14 |
|  | defense response | 6.03E-14 |
|  | inflammatory response | 4.55E-07 |
|  | positive regulation of immune system process | 7.79E-07 |
|  | regulation of immune system process | 1.12E-06 |
|  | response to wounding | 1.25E-06 |
|  | cell activation | 1.55E-06 |
|  | regulation of response to stimulus | 2.14E-06 |
|  | regulation of immune response | 2.41E-06 |
|  | leukocyte activation | 1.24E-05 |
|  | cytokine production | 1.55E-05 |
|  | response to stimulus | 2.69E-05 |
|  | response to external stimulus | 6.04E-05 |
|  | lymphocyte activation | 9.13E-05 |
|  | immune effector process | 9.67E-05 |
|  | positive regulation of response to stimulus | 0.00018916 |
|  | regulation of cytokine production | 0.00023775 |
|  | response to stress | 0.00030175 |
|  | leukocyte mediated immunity | 0.00030175 |
|  | regulation of cell activation | 0.0006758 |
|  | T cell activation | 0.00107144 |
|  | adaptive immune response | 0.0012034 |
|  | adaptive immune response based on somatic recombination of immune receptors built from immunoglobulin superfamily domains | 0.0012034 |
|  | positive regulation of immune response | 0.0012034 |
|  | regulation of leukocyte activation | 0.00236158 |
|  | regulation of leukocyte mediated immunity | 0.0036272 |
|  | positive regulation of cell activation | 0.00423679 |
|  | regulation of immune effector process | 0.00704794 |
|  | immune response-regulating signaling pathway | 0.0080241 |
|  | antigen processing and presentation | 0.0080241 |
|  | response to other organism | 0.00863287 |
|  | regulation of lymphocyte activation | 0.0086939 |
|  | lymphocyte mediated immunity | 0.0086939 |
|  | innate immune response | 0.0086939 |
|  | positive regulation of cytokine production | 0.01230111 |
|  | cell activation during immune response | 0.01230111 |
|  | leukocyte activation during immune response | 0.01230111 |
|  | positive regulation of leukocyte activation | 0.01643939 |
|  | activation of immune response | 0.0168412 |
|  | defense response to bacterium | 0.01715852 |
|  | immune response-activating signal transduction | 0.0171706 |
|  | positive regulation of developmental process | 0.01868173 |
|  | B cell activation | 0.02930784 |
|  | response to biotic stimulus | 0.03314845 |
|  | regulation of adaptive immune response | 0.03663163 |
|  | regulation of adaptive immune response based on somatic recombination of immune receptors built from immunoglobulin superfamily domains | 0.03663163 |
|  | immune system development | 0.04105504 |
|  | programmed cell death | 0.04309409 |
|  | cell death | 0.0441013 |
|  | mononuclear cell proliferation | 0.04897404 |
|  | lymphocyte proliferation | 0.04897404 |

Both the Gene Ontology term (GO Term) and the Benjamini and Yekutieli false discovery rate (BY FDR) are shown for modules that had significant representation in GO-terms. 5 modules (A, C, E, J, and L) were not significantly enriched for any GO terms.
