# Supplementary material for: Modeling Host Genetic Regulation of Influenza Pathogenesis in the Collaborative Cross
Source: PLoS Pathog. 2013 Feb 28;9(2):e1003196. doi: 10.1371/journal.ppat.1003196 (PMC3585141; doi:10.1371/journal.ppat.1003196)
Supplement: Table S6 — Mx1 sequence variants. (DOCX) [file ppat.1003196.s012.docx]

**Table S6. *Mx1* sequence variants**

**Position(b37) Exon A B C D E F G H Type strand**

97684505 1 G G G G G C G G 5’UTR 5-3

97684501 1 A A A A A G A A 5’UTR 5-3

97684494 1 A A A A A T A A 5’UTR 5-3

97682477 2 AGG AGG AGG AGG AGG --- AGG AGG 5’UTR 5-3

97682366 2 T T T T C C C C 5’UTR 5-3

97679069 3 C C C C C C C T E → L 5-3

97678962 3 A A A A T T T A Silent 5-3

97678947 3 C C C C A A A C Silent 5-3

97677916 4 C C C C C T C C G → R 5-3

97677878 4 A A A A G T G A Silent 5-3

97677836 4 A A A A A G A A Silent 5-3

97676770 6 G G G G G A G G Silent 5-3

97675794 7 G G G G G A G G Silent 5-3

na 9,10,11 - - - - 9,10,11 9,10,11 9,10,11 9,10,11 Deletion

na 10 T T T T T T T A STOP codon 5-3

97669894 14 G G G G A A A A T → M 5-3

97669824 14 C C C C C C C T Silent 5-3

97669806 14 C C C C T C C C G → R 5-3

97669680 14 G G G G A A A A 3’UTR 5-3

97669583 14 T T T T C C T C 3’UTR 5-3

97669531 14 ---- ---- ---- ---- AGTT AGTT AGTT AGTT 3’UTR 5-3

97669430 14 C C C C C C C T 3’UTR 5-3

97669409 14 G G G G A A A A 3’UTR 5-3

97669294 14 G G G G G A G G 3’UTR 5-3

97669267 14 T T T T C C C C 3’UTR 5-3

97669162 14 G G G G G A G G 3’UTR 5-3

97669128 14 G G G G A G G G 3’UTR 5-3

97669006 14 A A A A A A G A 3’UTR 5-3

97668998 14 T T T T C C C C 3’UTR 5-3

97668882 14 T T T T C C C C 3’UTR 5-3

97668700 14 AGTT AGTT AGTT AGTT AGTT AGTT ---- AGTT 3’UTR 5-3

97668684 14 G G G G T T T T 3’UTR 5-3

97668670 14 G G G G G G G A 3’UTR 5-3

A-H refer to founder strains. A=A/J, B=C57BL6/J, C=129S1/SvImJ, D=NOD/ShiLtJ, E=NZO/HILtJ, F=CAST/EiJ, G=PWK/PhJ, H=WSB/EiJ
