# Supplementary material for: Modeling Host Genetic Regulation of Influenza Pathogenesis in the Collaborative Cross
Source: PLoS Pathog. 2013 Feb 28;9(2):e1003196. doi: 10.1371/journal.ppat.1003196 (PMC3585141; doi:10.1371/journal.ppat.1003196)
Supplement: Table S7 — Transcripts with an eQTL at Mx1. (DOCX) [file ppat.1003196.s013.docx]

| **Table S7. Transcripts with an eQTL at *Mx1*** | |  |
| --- | --- | --- |
| **Transcript** | **High expression alleles** | **Low expression alleles** |
| Glt28d2 | ABCDEFG | H |
| Arl13b | ABCDEFG | H |
| Dyrk1a | ABCDEFH | G |
| Synj1 | ABCDEFH | G |
| Picalm | ABCDEFH | G |
| AK038388 | ABCDEFH | G |
| Dcp2 | ABCDEFH | G |
| Snag1 | ABCDEFH | G |
| Kbtbd2 | ABCDEFH | G |
| Clec5a | ABCDEFH | G |
| 5530401N12Rik | ABCDEFH | G |
| Gtf2f1 | ABCDEFH | G |
| Itpkc | ABCDEFH | G |
| Sdccag10 | ABCDEFH | G |
| Tgs1 | ABCDEFH | G |
| Nat5 | ABCDEFH | G |
| Nlrp3 | ABCDEFH | G |
| Sfrs2 | ABCDEFH | G |
| Fem1c | ABCDEFH | G |
| Slc20a1 | ABCDEFH | G |
| Lincr | ABCDEFH | G |
| Timeless | ABCDEFH | G |
| 5830400J07Rik | ABCDEFH | G |
| Ccr2 | ABCDEFH | G |
| Nup54 | ABCDEFH | G |
| Tmem185b | ABCDEFH | G |
| 9430008C03Rik | ABCDEFH | G |
| Sp100 | ABCDEFH | G |
| AA960436 | ABCDEFH | G |
| 2700007P21Rik | ABCDEFH | G |
| Slc35d2 | ABCDEFH | G |
| Chic2 | ABCDEFH | G |
| Osmr | ABCDEFH | G |
| Als2 | ABCDEFH | G |
| Gyk | ABCDEFH | G |
| 3300001M20Rik | ABCDEFH | G |
| Gtpbp4 | ABCDEFH | G |
| Pdzrn3 | ABCDEFH | G |
| Tgs1 | ABCDEFH | G |
| Gmfb | ABCDEFH | G |
| Hbegf | ABCDEFH | G |
| Bin3 | ABCDEFH | G |
| Zdhhc5 | ABCDEFH | G |
| Atf1 | ABCDEFH | G |
| CF747846 | ABCDEFH | G |
| Trps1 | ABCDEFH | G |
| Sar1a | ABCDEFH | G |
| Pycard | ABCDEFH | G |
| Cnih2 | ABCDEFH | G |
| Rps6ka3 | ABCDEFH | G |
| Donson | ABCDEFH | G |
| Adamts1 | ABCDEFH | G |
| NAP060490-1 | ABCDEFH | G |
| Hivep3 | ABCDEFH | G |
| Flnb | ABCDEFH | G |
| Cysltr1 | ABCDEFH | G |
| NAP049188-1 | ABCDEFH | G |
| Srfbp1 | ABCDEFH | G |
| A_52_P1013432 | ABCDEFH | G |
| Marcks | ABCDEFH | G |
| Wsb1 | ABCDEFH | G |
| Trim26 | ABCDEFH | G |
| 4833446K15Rik | ABCDEFH | G |
| Capzb | ABCDEFH | G |
| Hk1 | ABCDEFH | G |
| Dclre1c | ABCDEFH | G |
| Sp100 | ABCDEFH | G |
| NAP096720-001 | ABCDEFH | G |
| Vti1a | ABCDEFH | G |
| Vasp | ABCDEFH | G |
| Cbfb | ABCDEFH | G |
| Sap30bp | ABCDEFH | G |
| Rhoc | ABCDEFH | G |
| Ubp1 | ABCDEFH | G |
| Rnf4 | ABCDEFH | G |
| Syncrip | ABCDEFH | G |
| Nek6 | ABCDEFH | G |
| Cd3eap | ABCDEFH | G |
| Larp1 | ABCDEFH | G |
| Mt1 | ABCDEFH | G |
| 4933435A13Rik | ABCDEFH | G |
| Ranbp3 | ABCDEFH | G |
| Foxk2 | ABCDEFH | G |
| Cysltr1 | ABCDEFH | G |
| Riok3 | ABCDEFH | G |
| ENSMUST00000053562 | ABCDEFH | G |
| Rnf12 | ABCDEFH | G |
| Kpna3 | ABCDEFH | G |
| Col4a2 | ABCDEFH | G |
| Pak1ip1 | ABCDEFH | G |
| Ranbp5 | ABCDEFH | G |
| Mki67ip | ABCDEFH | G |
| Mif | ABCDEFH | G |
| Srp72 | ABCDEFH | G |
| Kcmf1 | ABCDEFH | G |
| Scamp4 | ABCDEFH | G |
| Nsf | ABCDEFH | G |
| Sdcbp | ABCDEFH | G |
| Nsf | ABCDEFH | G |
| 1110007M04Rik | ABCDEFH | G |
| 1500002O20Rik | ABCDEFH | G |
| Bysl | ABCDEFH | G |
| Setd4 | ABCDEGH | F |
| 2810055G20Rik | ABCDEGH | F |
| Ets2 | ABCDEH | FG |
| Dscr1 | ABCDFGH | E |
| Gripap1 | ABCDFGH | E |
| Eif5 | ABCDFGH | E |
| Inoc1 | ABCDFGH | E |
| Grin3b | ABCDFGH | E |
| Gng12 | ABCDFGH | E |
| Mxd1 | ABCDFH | EG |
| Icam5 | ABCDFH | EG |
| Srgn | ABCDFH | EG |
| Hif1a | ABCDFH | EG |
| Marcksl1 | ABCDFH | EG |
| Arid5a | ABCDFH | EG |
| Igsf6 | ABCDFH | EG |
| Cdk5r1 | ABCDFH | EG |
| Rnd3 | ABCDFH | EG |
| Marcksl1 | ABCDFH | EG |
| Enc1 | ABCDFH | EG |
| Litaf | ABCDFH | EG |
| Slc39a14 | ABCDFH | EG |
| AK008862 | ABCDFH | EG |
| Mefv | ABCDFH | EG |
| Rnf149 | ABCDFH | EG |
| Dok3 | ABCDFH | EG |
| 8030431J09Rik | ABCDFH | EG |
| Axud1 | ABCDFH | EG |
| Pram1 | ABCDFH | EG |
| Tnfrsf1b | ABCDFH | EG |
| Rhcg | ABCDFH | EG |
| Csf3r | ABCDFH | EG |
| Il17ra | ABCDFH | EG |
| Serpine1 | ABCDFH | EG |
| Plk3 | ABCDFH | EG |
| 0910001A06Rik | ABCDFH | EG |
| Trib1 | ABCDFH | EG |
| Etv6 | ABCDFH | EG |
| Myc | ABCDFH | EG |
| Gcnt2 | ABCDFH | EG |
| Myc | ABCDFH | EG |
| 2310014H01Rik | ABCDFH | EG |
| Il17ra | ABCDFH | EG |
| Rnf149 | ABCDFH | EG |
| Adm | ABCDFH | EG |
| Atf3 | ABCDFH | EG |
| Sbno2 | ABCDFH | EG |
| Plekhq1 | ABCDFH | EG |
| Rnd3 | ABCDFH | EG |
| Zc3h12a | ABCDFH | EG |
| Slc2a1 | ABCDFH | EG |
| BC004022 | ABCDFH | EG |
| C330023M02Rik | ABCDFH | EG |
| Jak2 | ABCDFH | EG |
| Ripk3 | ABCDFH | EG |
| Vcan | ABCDFH | EG |
| Cd300lf | ABCDFH | EG |
| Pstpip1 | ABCDFH | EG |
| Bcl2l11 | ABCDFH | EG |
| 1810009K13Rik | ABCDFH | EG |
| 0910001A06Rik | ABCDFH | EG |
| Tnip1 | ABCDFH | EG |
| Slc16a3 | ABCDFH | EG |
| Daxx | ABCDFH | EG |
| Ltb4r1 | ABCDFH | EG |
| Ptpn1 | ABCDFH | EG |
| Tnfaip8l2 | ABCDFH | EG |
| Ier5l | ABCDFH | EG |
| BC013712 | ABCDFH | EG |
| Sell | ABCDFH | EG |
| Plek | ABCDFH | EG |
| Ccdc88 | ABCDFH | EG |
| Mgat4a | ABCDFH | EG |
| Abtb2 | ABCDFH | EG |
| AI447904 | ABCDFH | EG |
| Irf5 | ABCDFH | EG |
| Selp | ABCDFH | EG |
| Zfp36 | ABCDFH | EG |
| Dennd1c | ABCDFH | EG |
| Chmp4b | ABCDFH | EG |
| Slfn2 | ABCDFH | EG |
| 1-Mar | ABCDFH | EG |
| Rab20 | ABCDFH | EG |
| Junb | ABCDFH | EG |
| C330023M02Rik | ABCDFH | EG |
| BC004022 | ABCDFH | EG |
| C330023M02Rik | ABCDFH | EG |
| Evi2a | ABCDFH | EG |
| Il13ra1 | ABCDFH | EG |
| Slc25a25 | ABCDFH | EG |
| Mlkl | ABCDFH | EG |
| Mapk6 | ABCDFH | EG |
| Lnpep | ABCDFH | EG |
| Fes | ABCDFH | EG |
| Mafb | ABCDFH | EG |
| Il4ra | ABCDFH | EG |
| Slc11a1 | ABCDFH | EG |
| Hcls1 | ABCDFH | EG |
| Slfn5 | ABCDFH | EG |
| Tspan4 | ABCDFH | EG |
| Arhgap30 | ABCDFH | EG |
| Plek | ABCDFH | EG |
| 9130017C17Rik | ABCDFH | EG |
| AK013903 | ABCDFH | EG |
| Vav1 | ABCDFH | EG |
| Was | ABCDFH | EG |
| Slamf6 | ABCDFH | EG |
| Vcpip1 | ABCDFH | EG |
| Rhoh | ABCDFH | EG |
| 2700019D07Rik | ABCDFH | EG |
| H3f3b | ABCDFH | EG |
| Ms4a4d | ABCDFH | EG |
| Sertad1 | ABCDFH | EG |
| Abcb1b | ABCDFH | EG |
| AK042092 | ABCDFH | EG |
| Was | ABCDFH | EG |
| Ms4a6c | ABCDFH | EG |
| Zc3hav1 | ABCDFH | EG |
| Ptpn2 | ABCDFH | EG |
| Hck | ABCDFH | EG |
| Dusp2 | ABCDFH | EG |
| A530032D15Rik | ABCDFH | EG |
| Trex1 | ABCDFH | EG |
| Rsad2 | ABCDFH | EG |
| Sema7a | ABCDFH | EG |
| Fgr | ABCDFH | EG |
| Plec1 | ABCDFH | EG |
| Arhgap30 | ABCDFH | EG |
| 1600014C10Rik | ABCDFH | EG |
| Ncf4 | ABCDFH | EG |
| Eif2s2 | ABCDFH | EG |
| A030007L17Rik | ABCDFH | EG |
| Pdlim7 | ABCDFH | EG |
| Picalm | ABCDFH | EG |
| Wdr4 | ABCDFH | EG |
| Nfkbib | ABCDFH | EG |
| Nt5c3 | ABCDFH | EG |
| Snap29 | ABCDFH | EG |
| Spag9 | ABCDFH | EG |
| Gla | ABCDFH | EG |
| Mcl1 | ABCDFH | EG |
| Gpr65 | ABCDFH | EG |
| AK043151 | ABCDFH | EG |
| Havcr2 | ABCDFH | EG |
| Ppp1r15b | ABCDFH | EG |
| Evi2b | ABCDFH | EG |
| Pdlim7 | ABCDFH | EG |
| Inhba | ABCDFH | EG |
| P2ry6 | ABCDFH | EG |
| Trafd1 | ABCDFH | EG |
| Il12rb1 | ABCDFH | EG |
| Maff | ABCDFH | EG |
| Chd7 | ABCDFH | EG |
| Slc25a37 | ABCDFH | EG |
| Rsad2 | ABCDFH | EG |
| Clic4 | ABCDFH | EG |
| Csf2rb2 | ABCDFH | EG |
| Eif4a1 | ABCDFH | EG |
| Tor1aip1 | ABCDFH | EG |
| Ier5 | ABCDFH | EG |
| Efhd2 | ABCDFH | EG |
| Pim1 | ABCDFH | EG |
| Fosl2 | ABCDFH | EG |
| LOC545342 | ABCDFH | EG |
| Fcer1g | ABCDFH | EG |
| Nktr | ABCDFH | EG |
| Dok1 | ABCDFH | EG |
| Dbn1 | ABCDFH | EG |
| Evi2b | ABCDFH | EG |
| Pbef1 | ABCDFH | EG |
| 2010012C16Rik | ABCDFH | EG |
| Oxsr1 | ABCDFH | EG |
| Azi2 | ABCDFH | EG |
| Ythdf1 | ABCDFH | EG |
| Snx2 | ABCDFH | EG |
| Socs1 | ABCDFH | EG |
| Psma4 | ABCDFH | EG |
| Birc3 | ABCDFH | EG |
| Tnc | ABCDFH | EG |
| 9430034N14Rik | ABCDFH | EG |
| 4933426M11Rik | ABCDFH | EG |
| Vcpip1 | ABCDFH | EG |
| Tmem49 | ABCDFH | EG |
| Pnp | ABCDFH | EG |
| Ykt6 | ABCDFH | EG |
| Slc39a1 | ABCDFH | EG |
| Ykt6 | ABCDFH | EG |
| Dcp2 | ABCDFH | EG |
| Amica1 | ABCDFH | EG |
| Aoah | ABCDFH | EG |
| Vav1 | ABCDFH | EG |
| Uck2 | ABCDFH | EG |
| Aftph | ABCDFH | EG |
| Rrbp1 | ABCDFH | EG |
| Pscd4 | ABCDFH | EG |
| Trafd1 | ABCDFH | EG |
| Tubb6 | ABCDFH | EG |
| Ms4a4b | ABCDFH | EG |
| Cd244 | ABCDFH | EG |
| Cflar | ABCDFH | EG |
| Coro1a | ABCDFH | EG |
| Zcchc2 | ABCDFH | EG |
| Hpse | ABCDFH | EG |
| Nat5 | ABCDFH | EG |
| TC1634227 | ABCDFH | EG |
| Cd86 | ABCDFH | EG |
| 1500012F01Rik | ABCDFH | EG |
| Spty2d1 | ABCDFH | EG |
| Myo1f | ABCDFH | EG |
| 2610208M17Rik | ABCDFH | EG |
| Stat3 | ABCDFH | EG |
| Ppp4c | ABCDFH | EG |
| Tbc1d1 | ABCDFH | EG |
| 1700017B05Rik | ABCDFH | EG |
| Rbm34 | ABCDFH | EG |
| Etf1 | ABCDFH | EG |
| Slamf7 | ABCDFH | EG |
| AI447904 | ABCDFH | EG |
| Lyn | ABCDFH | EG |
| Wdr43 | ABCDFH | EG |
| Skap2 | ABCDFH | EG |
| Ifit2 | ABCDFH | EG |
| Cd52 | ABCDFH | EG |
| Noc3l | ABCDFH | EG |
| Pbef1 | ABCDFH | EG |
| Katna1 | ABCDFH | EG |
| Ifi204 | ABCDFH | EG |
| Rpp38 | ABCDFH | EG |
| Csf2ra | ABCDFH | EG |
| Ubtd1 | ABCDFH | EG |
| Emilin2 | ABCDFH | EG |
| Tcirg1 | ABCDFH | EG |
| Bak1 | ABCDFH | EG |
| AY078069 | ABCDFH | EG |
| Ccl5 | ABCDFH | EG |
| Rbm13 | ABCDFH | EG |
| Aif1 | ABCDFH | EG |
| BC032204 | ABCDFH | EG |
| Usp25 | ABCDFH | EG |
| Rrbp1 | ABCDFH | EG |
| Grina | ABCDFH | EG |
| 5-Mar | ABCDFH | EG |
| Sgk3 | ABCDFH | EG |
| Vcpip1 | ABCDFH | EG |
| Rrp9 | ABCDFH | EG |
| 0910001A06Rik | ABCDFH | EG |
| Runx3 | ABCDFH | EG |
| Rap2c | ABCDFH | EG |
| Ccl2 | ABCDFH | EG |
| Tmem127 | ABCDFH | EG |
| Ccrl2 | ABCDFH | EG |
| Rars | ABCDFH | EG |
| Fst | ABCDFH | EG |
| Josd3 | ABCDFH | EG |
| Noc4l | ABCDFH | EG |
| Ptpn6 | ABCDFH | EG |
| Pnpt1 | ABCDFH | EG |
| Capzb | ABCDFH | EG |
| Capza2 | ABCDFH | EG |
| Gm1966 | ABCDFH | EG |
| Il15ra | ABCDFH | EG |
| Zfand5 | ABCDFH | EG |
| AK087356 | ABCDFH | EG |
| A430084P05Rik | ABCDFH | EG |
| Gch1 | ABCDFH | EG |
| Rhog | ABCDFH | EG |
| Cyp4f18 | ABCDFH | EG |
| Smap1l | ABCDFH | EG |
| Nfkb2 | ABCDFH | EG |
| Slc12a9 | ABCDFH | EG |
| Pcgf5 | ABCDFH | EG |
| 2010106G01Rik | ABCDFH | EG |
| Glipr2 | ABCDFH | EG |
| Syk | ABCDFH | EG |
| Darc | ABCDFH | EG |
| Sgpl1 | ABCDFH | EG |
| Psmd12 | ABCDFH | EG |
| Rab43 | ABCDFH | EG |
| Ogfr | ABCDFH | EG |
| Dcp1a | ABCDFH | EG |
| Casp8 | ABCDFH | EG |
| Lair1 | ABCDFH | EG |
| Gmip | ABCDFH | EG |
| A_52_P981179 | ABCDFH | EG |
| Eif4e2 | ABCDFH | EG |
| Spata13 | ABCDFH | EG |
| Tyki | ABCDFH | EG |
| Gosr2 | ABCDFH | EG |
| Rhbdf2 | ABCDFH | EG |
| TC1645771 | ABCDFH | EG |
| Rhbdf2 | ABCDFH | EG |
| Sdad1 | ABCDFH | EG |
| Fcgr2b | ABCDFH | EG |
| Csnk1d | ABCDFH | EG |
| Zcchc2 | ABCDFH | EG |
| Samhd1 | ABCDFH | EG |
| Apob48r | ABCDFH | EG |
| Sap30 | ABCDFH | EG |
| Leprotl1 | ABCDFH | EG |
| Aoc2 | ABCDFH | EG |
| Mpeg1 | ABCDFH | EG |
| TC1688109 | ABCDFH | EG |
| Wipf1 | ABCDFH | EG |
| Cpsf2 | ABCDFH | EG |
| F10 | ABCDFH | EG |
| C3ar1 | ABCDFH | EG |
| P2ry14 | ABCDFH | EG |
| Emilin2 | ABCDFH | EG |
| AK040628 | ABCDFH | EG |
| Bcl3 | ABCDFH | EG |
| Ppp1r15b | ABCDFH | EG |
| Ifi35 | ABCDFH | EG |
| Wsb1 | ABCDFH | EG |
| Tlk2 | ABCDFH | EG |
| Coro1a | ABCDFH | EG |
| Josd3 | ABCDFH | EG |
| Slfn3 | ABCDFH | EG |
| Nufip1 | ABCDFH | EG |
| 1110038B12Rik | ABCDFH | EG |
| Plaur | ABCDFH | EG |
| EG668139 | ABCDFH | EG |
| Tnfrsf12a | ABCDFH | EG |
| Tpd52 | ABCDFH | EG |
| Lgals8 | ABCDFH | EG |
| Cutc | ABCDFH | EG |
| NAP107236-1 | ABCDFH | EG |
| Hipk2 | ABCDFH | EG |
| Has2 | ABCDFH | EG |
| Fgl2 | ABCDFH | EG |
| ENSMUST00000073088 | ABCDFH | EG |
| Tomm20 | ABCDFH | EG |
| AK089832 | ABCDFH | EG |
| Spsb1 | ABCDFH | EG |
| Atp8b4 | ABCDFH | EG |
| Mrto4 | ABCDFH | EG |
| Irf2 | ABCDFH | EG |
| Atp8b1 | ABCDFH | EG |
| Ncoa3 | ABCDFH | EG |
| Dock2 | ABCDFH | EG |
| D630023B12Rik | ABCDFH | EG |
| Sp100 | ABCDFH | EG |
| Rbm19 | ABCDFH | EG |
| Asb13 | ABCDFH | EG |
| Zfp36 | ABCDFH | EG |
| Vps37b | ABCDFH | EG |
| Ugcg | ABCDFH | EG |
| Fndc3a | ABCDFH | EG |
| 3110043O21Rik | ABCDFH | EG |
| Vps54 | ABCDFH | EG |
| Cdv3 | ABCDFH | EG |
| BC016423 | ABCDFH | EG |
| Gosr2 | ABCDFH | EG |
| BC016423 | ABCDFH | EG |
| Rbm9 | ABCDFH | EG |
| Ptgir | ABCDFH | EG |
| Gzmb | ABCDFH | EG |
| Arhgap25 | ABCDFH | EG |
| Mizf | ABCDFH | EG |
| AK081327 | ABCDFH | EG |
| Ankrd49 | ABCDFH | EG |
| 5830443L24Rik | ABCDFH | EG |
| Atxn7l3 | ABCDFH | EG |
| Gbp4 | ABCDFH | EG |
| Smn1 | ABCDFH | EG |
| Thbs1 | ABCDFH | EG |
| 1700027J05Rik | ABCDFH | EG |
| Cyp7b1 | ABCDFH | EG |
| Map3k14 | ABCDFH | EG |
| Cd244 | ABCDFH | EG |
| NAP019557-001 | ABCDFH | EG |
| 5-Mar | ABCDFH | EG |
| Lrp1 | ABCDFH | EG |
| Itga4 | ABCDFH | EG |
| AK039806 | ABCDFH | EG |
| Ctdp1 | ABCDFH | EG |
| Yrdc | ABCDFH | EG |
| Gtf2f2 | ABCDFH | EG |
| Tox4 | ABCDFH | EG |
| Nfam1 | ABCDFH | EG |
| Rbm18 | ABCDFH | EG |
| ORF5 | ABCDFH | EG |
| Parp14 | ABCDFH | EG |
| TC1717672 | ABCDFH | EG |
| Zfp364 | ABCDFH | EG |
| Irgm | ABCDFH | EG |
| Bean | ABCDFH | EG |
| Kpna3 | ABCDFH | EG |
| 6330578E17Rik | ABCDFH | EG |
| Dr1 | ABCDFH | EG |
| 2010106G01Rik | ABCDFH | EG |
| Irgm | ABCDFH | EG |
| Tyrobp | ABCDFH | EG |
| Seh1l | ABCDFH | EG |
| Srm | ABCDFH | EG |
| BC023892 | ABCDFH | EG |
| Snx8 | ABCDFH | EG |
| Pitpna | ABCDFH | EG |
| Maf | ABCDFH | EG |
| Ubap1 | ABCDFH | EG |
| Stam2 | ABCDFH | EG |
| C230075M21Rik | ABCDFH | EG |
| Mobkl2a | ABCDFH | EG |
| Ifnar2 | ABCDFH | EG |
| Trim21 | ABCDFH | EG |
| Psmd3 | ABCDFH | EG |
| AK037475 | ABCDFH | EG |
| AA407452 | ABCDFH | EG |
| Plaur | ABCDFH | EG |
| Pik3cd | ABCDFH | EG |
| Brd2 | ABCDFH | EG |
| 1300018I05Rik | ABCDFH | EG |
| Fbxo42 | ABCDFH | EG |
| Epha2 | ABCDFH | EG |
| Trim30 | ABCDFH | EG |
| Mt1 | ABCDFH | EG |
| Samd9l | ABCDFH | EG |
| Sos1 | ABCDFH | EG |
| Edem1 | ABCDFH | EG |
| Mdk | ABCDFH | EG |
| NAP102462-1 | ABCDFH | EG |
| TC1677080 | ABCDFH | EG |
| Dpy19l1 | ABCDFH | EG |
| Plcg2 | ABCDFH | EG |
| Mapk7 | ABCDFH | EG |
| Ftsj3 | ABCDFH | EG |
| Pwp2 | ABCDFH | EG |
| Gnl3 | ABCDFH | EG |
| Coro7 | ABCDFH | EG |
| AK012844 | ABCDFH | EG |
| Tbrg4 | ABCDFH | EG |
| Adam21 | ABCDFH | EG |
| Smg7 | ABCDFH | EG |
| Ogfrl1 | ABCDFH | EG |
| D10Ertd438e | ABCDFH | EG |
| EG382448 | ABCDFH | EG |
| Baz1a | ABCDFH | EG |
| Tial1 | ABCDFH | EG |
| 1110032O16Rik | ABCDFH | EG |
| 1110018G07Rik | ABCDFH | EG |
| Lair1 | ABCDFH | EG |
| Psmb10 | ABCDFH | EG |
| Surf4 | ABCDFH | EG |
| Cul2 | ABCDFH | EG |
| Rassf1 | ABCDFH | EG |
| Cyba | ABCDFH | EG |
| Pfn1 | ABCDFH | EG |
| Ppp4c | ABCDFH | EG |
| Ptpn22 | ABCDFH | EG |
| Nol10 | ABCDFH | EG |
| Prkx | ABCDFH | EG |
| Olfr741 | ABCDFH | EG |
| 9530028C05 | ABCDFH | EG |
| Zfp384 | ABCDFH | EG |
| Atp8b2 | ABCDFH | EG |
| Gmppb | ABCDFH | EG |
| Arpc4 | ABCDFH | EG |
| Sema4c | ABCDFH | EG |
| AI481105 | ABCDFH | EG |
| BC027231 | ABCDFH | EG |
| H2-T22 | ABCDFH | EG |
| Gfpt2 | ABCDFH | EG |
| Midn | ABCDFH | EG |
| Stat2 | ABCDFH | EG |
| Zfp513 | ABCDFH | EG |
| 3110005G23Rik | ABCDFH | EG |
| Fos | ABCDFH | EG |
| Nol5 | ABCDFH | EG |
| Atp7a | ABCDFH | EG |
| A_51_P462771 | ABCDFH | EG |
| NAP007796-001 | ABCDFH | EG |
| Nip7 | ABCDFH | EG |
| Otud5 | ABCDFH | EG |
| Pus1 | ABCDFH | EG |
| Zfp668 | ABCDFH | EG |
| Clic1 | ABCDFH | EG |
| Thbs1 | ABCDFH | EG |
| BC069963 | ABCDFH | EG |
| Pwp2 | ABCDFH | EG |
| Gtf2f2 | ABCDFH | EG |
| BC051230 | ABCDFH | EG |
| Psma4 | ABCDFH | EG |
| Oaf | ABCDFH | EG |
| Eif3s7 | ABCDFH | EG |
| Tmem106a | ABCDFH | EG |
| Cebpb | ABCDFH | EG |
| Lrrc59 | ABCDFH | EG |
| Lrrc4 | ABCDFH | EG |
| Akp2 | ABCDFH | EG |
| Slc30a1 | ABCDFH | EG |
| BC013672 | ABCDFH | EG |
| Dtx4 | ABCDFH | EG |
| Kpnb1 | ABCDFH | EG |
| Herc5 | ABCDFH | EG |
| B230217C12Rik | ABCDFH | EG |
| Pnpt1 | ABCDFH | EG |
| Cyr61 | ABCDFH | EG |
| Nup50 | ABCDFH | EG |
| Azi2 | ABCDFH | EG |
| Pols | ABCDFH | EG |
| Mospd4 | ABCDFH | EG |
| Rnf213 | ABCDFH | EG |
| Ncf1 | ABCDFH | EG |
| Cflar | ABCDFH | EG |
| Siglec1 | ABCDFH | EG |
| 6820401H01Rik | ABCDFH | EG |
| Zkscan6 | ABCDFH | EG |
| NAP025806-1 | ABCDFH | EG |
| B2m | ABCDFH | EG |
| Dimt1 | ABCDFH | EG |
| 2010003J03Rik | ABCDFH | EG |
| Isg20 | ABCDFH | EG |
| Unc93b1 | ABCDFH | EG |
| AK084024 | ABCDFH | EG |
| Scyl3 | ABCDFH | EG |
| Axl | ABCDFH | EG |
| Itga4 | ABCDFH | EG |
| 2010305A19Rik | ABCDFH | EG |
| Ddx58 | ABCDFH | EG |
| Plod3 | ABCDFH | EG |
| Creb5 | ABCDFH | EG |
| Smndc1 | ABCDFH | EG |
| Vps54 | ABCDFH | EG |
| Dennd1a | ABCDFH | EG |
| Ppp2r2a | ABCDFH | EG |
| Ube2q1 | ABCDFH | EG |
| Zfand3 | ABCDFH | EG |
| Yars | ABCDFH | EG |
| AK041551 | ABCDFH | EG |
| Ilf3 | ABCDFH | EG |
| Lyl1 | ABCDFH | EG |
| 2410001C21Rik | ABCDFH | EG |
| Tspo | ABCDFH | EG |
| Rnase6 | ABCDFH | EG |
| Hnrpd | ABCDFH | EG |
| Spata5 | ABCDFH | EG |
| Prdm2 | ABCDFH | EG |
| Apobec1 | ABCDFH | EG |
| Ascc3 | ABCDFH | EG |
| Igf2bp2 | ABCDFH | EG |
| 4933426M11Rik | ABCDFH | EG |
| Ugcg | ABCDFH | EG |
| ENSMUST00000101511 | ABCDFH | EG |
| Ppa1 | ABCDFH | EG |
| Emp3 | ABCDFH | EG |
| Wdsof1 | ABCDFH | EG |
| Ddx54 | ABCDFH | EG |
| Atf6 | ABCDFH | EG |
| Cars | ABCDFH | EG |
| Cxcl16 | ABCDFH | EG |
| D4Ertd22e | ABCDFH | EG |
| Dnmt3a | ABCDFH | EG |
| LOC667370 | ABCDFH | EG |
| BC011248 | ABCDFH | EG |
| St3gal4 | ABCDFH | EG |
| Med10 | ABCDFH | EG |
| Aars | ABCDFH | EG |
| Phlda1 | ABCDFH | EG |
| Sdcbp2 | ABCDFH | EG |
| Stat1 | ABCDFH | EG |
| Itgb4bp | ABCDFH | EG |
| 4732496O08Rik | ABCDFH | EG |
| AK169992 | ABCDFH | EG |
| Hdh | ABCDFH | EG |
| Xrn2 | ABCDFH | EG |
| Rab11fip5 | ABCDFH | EG |
| Garnl4 | ABCDFH | EG |
| Gars | ABCDFH | EG |
| Pld4 | ABCDFH | EG |
| EG634650 | ABCDFH | EG |
| Adar | ABCDFH | EG |
| Cd68 | ABCDFH | EG |
| Il18bp | ABCDFH | EG |
| Asb13 | ABCDFH | EG |
| Tlk2 | ABCDFH | EG |
| Sirpa | ABCDFH | EG |
| Pop7 | ABCDFH | EG |
| Cebpb | ABCDFH | EG |
| Ifrg15 | ABCDFH | EG |
| Prrx1 | ABCDFH | EG |
| Psmc4 | ABCDFH | EG |
| Has1 | ABCDFH | EG |
| Ipo4 | ABCDFH | EG |
| Phf11 | ABCDFH | EG |
| H3f3b | ABCDFH | EG |
| Tspo | ABCDFH | EG |
| Kpna3 | ABCDFH | EG |
| Snx5 | ABCDFH | EG |
| Ugcg | ABCDFH | EG |
| Hsh2d | ABCDFH | EG |
| Flnb | ABCDFH | EG |
| Plekho1 | ABCDFH | EG |
| Sco1 | ABCDFH | EG |
| Psmd14 | ABCDFH | EG |
| Lilrb4 | ABCDFH | EG |
| Tle3 | ABCDFH | EG |
| Taf7 | ABCDFH | EG |
| Rab43 | ABCDFH | EG |
| Prtn3 | ABCDFH | EG |
| Atf4 | ABCDFH | EG |
| Dnajc2 | ABCDFH | EG |
| Wdr40c | ABCDFH | EG |
| Il24 | ABCDFH | EG |
| NAP113293-1 | ABCDFH | EG |
| Il18bp | ABCDFH | EG |
| Tbc1d9 | ABCDFH | EG |
| Rbm43 | ABCDFH | EG |
| Dnttip2 | ABCDFH | EG |
| Zc3h7a | ABCDFH | EG |
| Furin | ABCDFH | EG |
| Nmt1 | ABCDFH | EG |
| Tsr1 | ABCDFH | EG |
| Thoc6 | ABCDFH | EG |
| Cdc42ep4 | ABCDFH | EG |
| Fasl | ABCDFH | EG |
| Isy1 | ABCDFH | EG |
| NAP000727-001 | ABCDFH | EG |
| Pfkp | ABCDFH | EG |
| Pilrb1 | ABCDFH | EG |
| Ttc9c | ABCDFH | EG |
| Crtc2 | ABCDFH | EG |
| Tdrd7 | ABCDFH | EG |
| Tnfaip3 | ABCDH | EFG |
| Gadd45b | ABCDH | EFG |
| Il1r2 | ABCDH | EFG |
| B430306N03Rik | ABCDH | EFG |
| Gadd45b | ABCDH | EFG |
| Sod2 | ABCDH | EFG |
| Zfp281 | ABCDH | EFG |
| Nfkbiz | ABCDH | EFG |
| Rod1 | ABCDH | EFG |
| Morc3 | ABCDH | EFG |
| Ch25h | ABCDH | EFG |
| Fpr-rs2 | ABCDH | EFG |
| Fcgr4 | ABCDH | EFG |
| Pdss1 | ABCDH | EFG |
| Snx10 | ABCDH | EFG |
| Ier3 | ABCDH | EFG |
| 5830416P10Rik | ABCDH | EFG |
| 2210403K04Rik | ABCDH | EFG |
| Ncoa3 | ABCDH | EFG |
| Ripk1 | ABCDH | EFG |
| Il18rap | ABCDH | EFG |
| Rab8b | ABCDH | EFG |
| Relb | ABCDH | EFG |
| Sema6b | ABCDH | EFG |
| Prdx5 | ABCDH | EFG |
| Spata20 | ABCDH | EFG |
| Mapk6 | ABCDH | EFG |
| Zbtb7a | ABCDH | EFG |
| Socs3 | ABCDH | EFG |
| AK139506 | ABCDH | EFG |
| AK082068 | ABCDH | EFG |
| A630082K20Rik | ABCDH | EFG |
| Spic | ABCDH | EFG |
| Asprv1 | ABCDH | EFG |
| Rrbp1 | ABCDH | EFG |
| Arid3a | ABCDH | EFG |
| Basp1 | ABCDH | EFG |
| EG435337 | ABCDH | EFG |
| Ptk2b | ABCDH | EFG |
| Ifi204 | ABCDH | EFG |
| Taf7 | ABCDH | EFG |
| Parp14 | ABCDH | EFG |
| Mllt1 | ABCDH | EFG |
| Eea1 | ABCDH | EFG |
| Gadd45g | ABCDH | EFG |
| Skil | ABCDH | EFG |
| Phca | ABCDH | EFG |
| Fndc3a | ABCDH | EFG |
| Slc16a6 | ABCDH | EFG |
| Eif5 | ABCDH | EFG |
| Nras | ABCDH | EFG |
| Cd14 | ABCDH | EFG |
| Crlz1 | ABCDH | EFG |
| Ihpk1 | ABCDH | EFG |
| Ccl12 | ABCDH | EFG |
| Ddx21 | ABCDH | EFG |
| Nol12 | ABCDH | EFG |
| Irf1 | ABCDH | EFG |
| Wibg | ABCDH | EFG |
| Il20rb | ABCDH | EFG |
| Retnlg | ABCDH | EFG |
| 2010109K11Rik | ABCDH | EFG |
| Fbxl5 | ABCDH | EFG |
| Thrap1 | ABCDH | EFG |
| Taf4b | ABCDH | EFG |
| Mdm4 | ABCDH | EFG |
| Pla1a | ABCDH | EFG |
| Larp1 | ABCDH | EFG |
| Lsg1 | ABCDH | EFG |
| 3010026O09Rik | ABCDH | EFG |
| Lass6 | ABCDH | EFG |
| Ihpk1 | ABCDH | EFG |
| Centa2 | ABCDH | EFG |
| Wwp2 | ABCDH | EFG |
| Ube1x | ABCDH | EFG |
| OTTMUSG00000015282 | ABCDH | EFG |
| Kpna4 | ABCDH | EFG |
| Tex10 | ABCDH | EFG |
| EG240327 | ABCDH | EFG |
| Plaa | ABCDH | EFG |
| Btg1 | ABCDH | EFG |
| Saa1 | ABCDH | EFG |
| Zfp296 | ABCDH | EFG |
| 1810054D07Rik | ABCDH | EFG |
| Nrbp1 | ABCDH | EFG |
| Mrgprg | ABCDH | EFG |
| BC033915 | ABCDH | EFG |
| Ptprc | ABCDH | EFG |
| Thbs1 | ABCDH | EFG |
| Nufip1 | ABCDH | EFG |
| Osgin1 | ABCDH | EFG |
| Wwp2 | ABCDH | EFG |
| Adrbk1 | ABCDH | EFG |
| Cxcl13 | ABCDH | EFG |
| Sphk1 | ABCDH | EFG |
| Znrf1 | ABCDH | EFG |
| Mthfd2 | ABCDH | EFG |
| NAP061805-1 | ABCDH | EFG |
| Sat1 | ABCDH | EFG |
| 5031439G07Rik | ABCDH | EFG |
| AI451617 | ABCDH | EFG |
| Saa3 | ABCDH | EFG |
| Ptpn12 | ABCDH | EFG |
| Gtf2h1 | ABCDH | EFG |
| Pfkfb3 | ABCDH | EFG |
| Crk | ABCDH | EFG |
| Dnajc2 | ABCDH | EFG |
| Sphk1 | ABCDH | EFG |
| Wbp4 | ABCDH | EFG |
| Ifng | ABCDH | EFG |
| AK035139 | ABCDH | EFG |
| Zfp143 | ABCDH | EFG |
| Arih1 | ABCDH | EFG |
| Znrf1 | ABCDH | EFG |
| Bcl2a1b | ABCDH | EFG |
| TC1665442 | ABCDH | EFG |
| Vps37c | ABCDH | EFG |
| Gosr1 | ABCDH | EFG |
| Atp8b2 | ABCDH | EFG |
| Csnk1d | ABCDH | EFG |
| Mansc1 | ABCEFG | DH |
| Tomm7 | ABCEFG | DH |
| Ly6g6d | ABCEFGH | D |
| C230078M08Rik | ABCEFGH | D |
| BC038613 | ABCEFGH | D |
| Ndufb10 | ABCEFGH | D |
| Fahd1 | ABCEFGH | D |
| Kif26b | ABCEFGH | D |
| Lyrm4 | ABCEFGH | D |
| Aprin | ABCEFGH | D |
| Ddit4l | ABCEFGH | D |
| Mett5d1 | ABCEFGH | D |
| Synj2 | ABCEFGH | D |
| Ipo8 | ABCEFGH | D |
| Pcyox1 | ABCEFGH | D |
| Angptl6 | ABCEFGH | D |
| Ube2e2 | ABCEFGH | D |
| Gart | ABCEFGH | D |
| 2410091C18Rik | ABCEFGH | D |
| Tada2l | ABCEFGH | D |
| Peg12 | ABCEFGH | D |
| Lrpprc | ABCEFGH | D |
| Kifap3 | ABCEFGH | D |
| Ccdc34 | ABCEFGH | D |
| Spg3a | ABCEFGH | D |
| Mapk11 | ABCH | DEFG |
| Tekt4 | ABDEFG | CH |
| Col8a2 | ABDEFG | CH |
| Mtap7 | ABDEFG | CH |
| Lamb3 | ABDEFG | CH |
| Tbcel | ABDEFG | CH |
| Trp53inp2 | ABDEFGH | C |
| TC1610785 | ABDEFGH | C |
| AU040829 | ABDEFGH | C |
| BC038167 | ABDEFGH | C |
| Rabgap1l | ABDEFGH | C |
| Gm114 | ABDEFGH | C |
| Aldh1a7 | ABDEFGH | C |
| Cyp2s1 | ABDEFGH | C |
| Sec14l3 | ABDEFGH | C |
| 3110004L20Rik | ABDEFGH | C |
| Eps8l1 | ABDEFGH | C |
| 4930562C15Rik | ABDEFGH | C |
| 2010300C02Rik | ABDEFGH | C |
| Slc25a36 | ABDEFGH | C |
| Kifap3 | ABDEFGH | C |
| D130043K22Rik | ABDEFGH | C |
| Cyp2a5 | ABDEFGH | C |
| ENSMUST00000035915 | ABDEFGH | C |
| 1700088E04Rik | ABDEFGH | C |
| Cyp2a4 | ABDEFGH | C |
| Krtcap3 | ABDEFGH | C |
| Ctxn1 | ABDEFGH | C |
| Osbpl5 | ABDEFGH | C |
| C77370 | ABDEFGH | C |
| Calcoco1 | ABDEFGH | C |
| 1700027N10Rik | ABDEFGH | C |
| Mapre2 | ABDEFGH | C |
| Fgfr2 | ABDEFGH | C |
| 1700021K14Rik | ABDEFGH | C |
| Pcnt | ABDEFGH | C |
| Mamdc2 | ABDEFGH | C |
| Akap9 | ABDEFGH | C |
| Cbr2 | ABDEFGH | C |
| AK076876 | ABDEFGH | C |
| Spa17 | ABDEFGH | C |
| Klhdc7a | ABDEFGH | C |
| 2610301F02Rik | ABDEFGH | C |
| Irs1 | ABDEFGH | C |
| Tfdp2 | ABDEFGH | C |
| 4-Mar | ABDEFGH | C |
| Grlf1 | ABDEFGH | C |
| Atp1b1 | ABDEFGH | C |
| Dag1 | ABDEFGH | C |
| C230094B09Rik | ABDEFGH | C |
| Ager | ABDEFGH | C |
| Dag1 | ABDEFGH | C |
| Traf3ip1 | ABDEFGH | C |
| Mlf1 | ABDEFGH | C |
| Tacc2 | ABDEFGH | C |
| Nfib | ABDEFGH | C |
| Herpud1 | ABDEFGH | C |
| Ptpn3 | ABDEFGH | C |
| Lamb2 | ABDEFGH | C |
| Sdc2 | ABDEFGH | C |
| Tmem110 | ABDEFGH | C |
| Magi1 | ABDEFGH | C |
| Pcyt2 | ABDEFGH | C |
| 4833401D15Rik | ABDEFGH | C |
| Timp2 | ABDEFGH | C |
| Ablim3 | ABDEFGH | C |
| Col4a6 | ABDEFGH | C |
| Col4a3bp | ABDEFGH | C |
| Vegfb | ABDEFGH | C |
| Tln2 | ABDEFGH | C |
| Cdc14a | ABDEFGH | C |
| 1600029D21Rik | ABDEFGH | C |
| Sh3d19 | ABDEFGH | C |
| Wwp1 | ABDEFGH | C |
| Arhgef18 | ABDEFGH | C |
| Spint1 | ABDEFGH | C |
| Cyb5r3 | ABDEFGH | C |
| Arrdc1 | ABDEFGH | C |
| Zfp704 | ABDEFGH | C |
| Mylk | ABDEFGH | C |
| Mlc1 | ABDEFGH | C |
| Scnn1b | ABDEFGH | C |
| Pik3c2a | ABDH | CEFG |
| Ccnl1 | ABDH | CEFG |
| Brd7 | ABEFG | CDH |
| Ttc3 | ABEFG | CDH |
| Atxn1 | ABEFG | CDH |
| Pcdh12 | ABEFG | CDH |
| Lsm14b | ABEFG | CDH |
| Pls3 | ABEFG | CDH |
| Exoc8 | ABEFG | CDH |
| Sytl4 | ABEFGH | CD |
| Egflam | ABEFGH | CD |
| Pigp | ABEGH | CDF |
| Cbr3 | ACDEFGH | B |
| Glcci1 | ACDEFGH | B |
| Jarid2 | ACDH | BEFG |
| 5730458M16Rik | ACDH | BEFG |
| Mr1 | ACEFG | BDH |
| Ciao1 | ACEFGH | BD |
| Tcp11l2 | ADEFG | BCH |
| Plekha1 | ADEFG | BCH |
| Iqca | AEFG | BCDH |
| Dynlrb2 | AEFG | BCDH |
| 4930430E16Rik | AEFG | BCDH |
| Dynlrb2 | AEFG | BCDH |
| Akap14 | AEFG | BCDH |
| Mgat3 | AEFG | BCDH |
| B3gnt4 | AEFG | BCDH |
| Bphl | AEFG | BCDH |
| Dyx1c1 | AEFG | BCDH |
| 2610015P09Rik | AEFG | BCDH |
| 1700094D03Rik | AEFG | BCDH |
| Hpn | AEFG | BCDH |
| Lrrc56 | AEFG | BCDH |
| 1700009P17Rik | AEFG | BCDH |
| Efcab1 | AEFG | BCDH |
| Ift88 | AEFG | BCDH |
| 4930455F23Rik | AEFG | BCDH |
| Kndc1 | AEFG | BCDH |
| Capsl | AEFG | BCDH |
| D330050I23Rik | AEFG | BCDH |
| Ccdc108 | AEFG | BCDH |
| Gstt3 | AEFG | BCDH |
| 9330101J02Rik | AEFG | BCDH |
| Fbxo25 | AEFG | BCDH |
| 6820408C15Rik | AEFG | BCDH |
| C730043O17 | AEFG | BCDH |
| Dnahc6 | AEFG | BCDH |
| Wdr63 | AEFG | BCDH |
| Dnahc2 | AEFG | BCDH |
| D430015B01Rik | AEFG | BCDH |
| 4933404M02Rik | AEFG | BCDH |
| Ccdc40 | AEFG | BCDH |
| Trp73 | AEFG | BCDH |
| Aim1l | AEFG | BCDH |
| Iqcg | AEFG | BCDH |
| Spag17 | AEFG | BCDH |
| EG432995 | AEFG | BCDH |
| Dnahc11 | AEFG | BCDH |
| Dnahc9 | AEFG | BCDH |
| Pih1d2 | AEFG | BCDH |
| Traf3ip1 | AEFG | BCDH |
| BC051019 | AEFG | BCDH |
| Dync2h1 | AEFG | BCDH |
| TC1671899 | AEFG | BCDH |
| Sigirr | AEFG | BCDH |
| 1700003M02Rik | AEFG | BCDH |
| Ppfibp2 | AEFG | BCDH |
| Nme5 | AEFG | BCDH |
| 3300002A11Rik | AEFG | BCDH |
| Paxip1 | AEFG | BCDH |
| Cep70 | AEFG | BCDH |
| Suv420h2 | AEFG | BCDH |
| Mgst2 | AEFG | BCDH |
| Spef1 | AEFG | BCDH |
| 1700123D08Rik | AEFG | BCDH |
| Spag16 | AEFG | BCDH |
| Gle1l | AEFG | BCDH |
| Myb | AEFG | BCDH |
| Gramd1c | AEFG | BCDH |
| A730055C05Rik | AEFG | BCDH |
| 9630019K15Rik | AEFG | BCDH |
| Fign | AEFG | BCDH |
| Ppp3ca | AEFG | BCDH |
| Ttc12 | AEFG | BCDH |
| Agbl2 | AEFG | BCDH |
| Map3k12 | AEFG | BCDH |
| Glcci1 | AEFG | BCDH |
| BC060267 | AEFG | BCDH |
| Ttc18 | AEFG | BCDH |
| Dbndd1 | AEFG | BCDH |
| Krtcap3 | AEFG | BCDH |
| Cat | AEFG | BCDH |
| Cluap1 | AEFG | BCDH |
| Nme5 | AEFG | BCDH |
| Numa1 | AEFG | BCDH |
| D14Ertd500e | AEFG | BCDH |
| Rpgr | AEFG | BCDH |
| 4933430H15Rik | AEFG | BCDH |
| Pvrl3 | AEFG | BCDH |
| AK079732 | AEFG | BCDH |
| Ankrd42 | AEFG | BCDH |
| Rere | AEFG | BCDH |
| Ccdc14 | AEFG | BCDH |
| Lrrc51 | AEFG | BCDH |
| Nek1 | AEFG | BCDH |
| 1700026L06Rik | AEFG | BCDH |
| Ankrd25 | AEFG | BCDH |
| Gtlf3b | AEFG | BCDH |
| Nsmce4a | AEFG | BCDH |
| Dab2ip | AEFG | BCDH |
| Dnajc12 | AEFG | BCDH |
| Cpsf6 | AEFG | BCDH |
| Scrn3 | AEFG | BCDH |
| D14Ertd436e | AEFG | BCDH |
| Sap30l | AEFG | BCDH |
| 2810488G03Rik | AEFG | BCDH |
| Casp2 | AEFG | BCDH |
| D19Ertd652e | AEFG | BCDH |
| Mxra8 | AEFG | BCDH |
| Bace1 | AEFG | BCDH |
| ENSMUST00000094652 | AEFG | BCDH |
| 3300001P08Rik | AEFG | BCDH |
| Endod1 | AEFG | BCDH |
| Prpf18 | AEFG | BCDH |
| Dzip1 | AEFG | BCDH |
| Ttll7 | AEFG | BCDH |
| Wdr52 | AEFG | BCDH |
| Osbpl10 | AEFG | BCDH |
| BC062650 | AEFG | BCDH |
| 4933404M02Rik | AEFG | BCDH |
| Rbmx | AEFG | BCDH |
| Grem2 | AEFG | BCDH |
| Usp33 | AEFG | BCDH |
| Prkce | AEFG | BCDH |
| Gphn | AEG | BCDFH |
| 2310030G06Rik | AEG | BCDFH |
| 9130221D24Rik | AEG | BCDFH |
| Irx2 | AEG | BCDFH |
| Otub2 | BCD | AEFGH |
| Lrch4 | BCDFH | AEG |
| Arpc2 | BCDFH | AEG |
| 5830482F20Rik | BCDFH | AEG |
| Nxn | BCDFH | AEG |
| NAP037326-1 | BCDFH | AEG |
| Surf4 | BCDFH | AEG |
| Rgs19 | BCDFH | AEG |
| Zc3hc1 | BCDFH | AEG |
| Il1r2 | BCDH | AEFG |
| S100a9 | BCDH | AEFG |
| Ccdc86 | BCDH | AEFG |
| Cirh1a | BCDH | AEFG |
| Fgd3 | BCDH | AEFG |
| Nfil3 | BCDH | AEFG |
| 2010002N04Rik | BCDH | AEFG |
| Nfil3 | BCDH | AEFG |
| Tpd52 | BCDH | AEFG |
| Mybbp1a | BCDH | AEFG |
| Cish | BCDH | AEFG |
| Dhrs9 | BCDH | AEFG |
| Eif4e2 | BCDH | AEFG |
| Samd8 | BCDH | AEFG |
| Prkaa1 | BCDH | AEFG |
| Tmem167 | BCDH | AEFG |
| Selplg | BCDH | AEFG |
| Creb3l1 | BCDH | AEFG |
| Rffl | BCDH | AEFG |
| TC1628823 | BCDH | AEFG |
| Cln8 | BCDH | AEFG |
| Sirpa | BCDH | AEFG |
| Zfp36l2 | BCDH | AEFG |
| Ptpn6 | BCDH | AEFG |
| ENSMUST00000077435 | BCDH | AEFG |
| Uck2 | BCDH | AEFG |
| Arrb2 | BCDH | AEFG |
| Prrc1 | BCDH | AEFG |
| EG619750 | BCDH | AEFG |
| Ankrd57 | BCDH | AEFG |
| 9030425E11Rik | BCDH | AEFG |
| Surf5 | BCDH | AEFG |
| Sgpl1 | BCDH | AEFG |
| TC1646163 | BCDH | AEFG |
| Mrpl52 | BCDH | AEFG |
| 5133401H06Rik | BCDH | AEFG |
| Runx1 | BCFGH | ADE |
| Gpr124 | BCFH | ADEG |
| Bop1 | BCH | ADEFG |
| NAP070973-1 | BCH | ADEFG |
| Timm10 | BCH | ADEFG |
| Dusp16 | BD | ACEFGH |
| Coq7 | BEFG | ACDH |
| Slc25a11 | BEFG | ACDH |
| Coq7 | BEFG | ACDH |
| Snrpn | BEFG | ACDH |
| Synj1 | C | ABDEFGH |
| Ahrr | C | ABDEFGH |
| Dbr1 | C | ABDEFGH |
| Josd3 | C | ABDEFGH |
| Cnnm4 | C | ABDEFGH |
| Tbrg4 | C | ABDEFGH |
| Zbtb6 | C | ABDEFGH |
| LOC219106 | C | ABDEFGH |
| Apaf1 | C | ABDEFGH |
| Isy1 | C | ABDEFGH |
| Msh3 | C | ABDEFGH |
| Trim27 | C | ABDEFGH |
| Ak2 | C | ABDEFGH |
| 1110054O05Rik | C | ABDEFGH |
| Rpl7l1 | C | ABDEFGH |
| Tomm70a | C | ABDEFGH |
| NAP057030-1 | C | ABDEFGH |
| Cul5 | C | ABDEFGH |
| Abl2 | C | ABDEFGH |
| Klhl15 | C | ABDEFGH |
| Ddx28 | C | ABDEFGH |
| Nrip3 | C | ABDEFGH |
| Adprh | C | ABDEFGH |
| Mtf1 | C | ABDEFGH |
| Ppp2r2a | C | ABDEFGH |
| AK041801 | C | ABDEFGH |
| Denr | C | ABDEFGH |
| Farsa | C | ABDEFGH |
| ENSMUST00000074789 | C | ABDEFGH |
| Sufu | C | ABDEFGH |
| Snx2 | C | ABDEFGH |
| Crlz1 | C | ABDEFGH |
| 1110007C09Rik | C | ABDEFGH |
| Atg5 | C | ABDEFGH |
| Yrdc | C | ABDEFGH |
| Umps | C | ABDEFGH |
| Zc3h12c | CD | ABEFGH |
| Cln3 | CD | ABEFGH |
| Inpp5d | CDH | ABEFG |
| 5730508B09Rik | CDH | ABEFG |
| Arsi | CDH | ABEFG |
| Col2a1 | CDH | ABEFG |
| Nup98 | CDH | ABEFG |
| Zfp385 | CDH | ABEFG |
| Vps33a | CDH | ABEFG |
| 4933439F18Rik | CEFG | ABDH |
| Mpzl1 | CEFG | ABDH |
| 0610007C21Rik | CEFG | ABDH |
| Nedd8 | CEFG | ABDH |
| Pfpl | CH | ABDEFG |
| 3110082I17Rik | CH | ABDEFG |
| Hpxn | D | ABCEFGH |
| Fbxw11 | D | ABCEFGH |
| BC033915 | D | ABCEFGH |
| AK047447 | D | ABCEFGH |
| Rragc | D | ABCEFGH |
| Pdlim5 | D | ABCEFGH |
| Zfp364 | D | ABCEFGH |
| Pbxip1 | DEFG | ABCH |
| AK035243 | DH | ABCEFG |
| Itfg1 | E | ABCDFGH |
| Tex264 | E | ABCDFGH |
| Prkcz | E | ABCDFGH |
| Sclt1 | E | ABCDFGH |
| Paqr4 | E | ABCDFGH |
| 2810432L12Rik | E | ABCDFGH |
| Lrrc8e | E | ABCDFGH |
| Ddx26b | E | ABCDFGH |
| Ncoa2 | E | ABCDFGH |
| Tmem64 | E | ABCDFGH |
| 2410005O16Rik | E | ABCDFGH |
| Rbl2 | E | ABCDFGH |
| Clic5 | E | ABCDFGH |
| BC022687 | E | ABCDFGH |
| Slc2a8 | E | ABCDFGH |
| Sos2 | E | ABCDFGH |
| Cacnb2 | E | ABCDFGH |
| Ptch1 | E | ABCDFGH |
| Snai2 | E | ABCDFGH |
| Rab3d | E | ABCDFGH |
| Ubr1 | E | ABCDFGH |
| E130308A19Rik | EF | ABCDGH |
| Hhip | EF | ABCDGH |
| Thbs3 | EFG | ABCDH |
| Fbxo36 | EFG | ABCDH |
| 0610011F06Rik | EFG | ABCDH |
| Bcas1 | EFG | ABCDH |
| 2310007A19Rik | EFG | ABCDH |
| Tmem50b | EFG | ABCDH |
| Zmynd10 | EFG | ABCDH |
| Mrpl14 | EFG | ABCDH |
| Cetn2 | EFG | ABCDH |
| Cetn2 | EFG | ABCDH |
| Crip2 | EFG | ABCDH |
| 1700026D08Rik | EFG | ABCDH |
| Epha1 | EFG | ABCDH |
| Wdr6 | EFG | ABCDH |
| Tyro3 | EFG | ABCDH |
| 1700010A17Rik | EFG | ABCDH |
| Zmym3 | EFG | ABCDH |
| Ccdc56 | EFG | ABCDH |
| 4921509J17Rik | EFG | ABCDH |
| Tmem107 | EFG | ABCDH |
| Sgsh | EFG | ABCDH |
| Ss18 | EFG | ABCDH |
| BC010787 | EFG | ABCDH |
| 1110004E09Rik | EFG | ABCDH |
| 1700025K23Rik | EFG | ABCDH |
| Zfp637 | EFG | ABCDH |
| Tmem59 | EFG | ABCDH |
| Abca2 | EFG | ABCDH |
| Nme3 | EFG | ABCDH |
| 2610003J06Rik | EFG | ABCDH |
| Strbp | EFG | ABCDH |
| Fzd2 | EFG | ABCDH |
| Antxr2 | EFG | ABCDH |
| Tmem4 | EFG | ABCDH |
| Mum1 | EFG | ABCDH |
| Ctf1 | EFG | ABCDH |
| 2510003E04Rik | EFG | ABCDH |
| Pigq | EFG | ABCDH |
| Ctnnb1 | EFG | ABCDH |
| Cbx6 | EFG | ABCDH |
| Chd6 | EFG | ABCDH |
| Chd6 | EFG | ABCDH |
| Grcc10 | EFG | ABCDH |
| Ccdc52 | EFG | ABCDH |
| Mtap7 | EFG | ABCDH |
| Hmgcll1 | EFG | ABCDH |
| Ganab | EFG | ABCDH |
| Tmem16a | EFG | ABCDH |
| 4930431B11Rik | EFG | ABCDH |
| Morn3 | EFG | ABCDH |
| Gpd1l | EFG | ABCDH |
| Btbd6 | EFG | ABCDH |
| Tm9sf2 | EFG | ABCDH |
| Zfp367 | EFG | ABCDH |
| Vps28 | EFG | ABCDH |
| Tmem14a | EFG | ABCDH |
| 1200009O22Rik | EFG | ABCDH |
| Rtn3 | EFG | ABCDH |
| Vkorc1 | EFG | ABCDH |
| Stk16 | EFG | ABCDH |
| Hdac8 | EFG | ABCDH |
| 1110051M20Rik | EFG | ABCDH |
| 1810037C20Rik | EFG | ABCDH |
| Skp1a | EFG | ABCDH |
| Ncapd3 | EFG | ABCDH |
| Pltp | EFG | ABCDH |
| Cbx6 | EFG | ABCDH |
| Hltf | EFG | ABCDH |
| Slc38a5 | EFG | ABCDH |
| Six5 | EFG | ABCDH |
| Anapc11 | EFG | ABCDH |
| Als2cr2 | EFG | ABCDH |
| Aox1 | EFG | ABCDH |
| Ggcx | EFG | ABCDH |
| Prss36 | EFG | ABCDH |
| Dstn | EFG | ABCDH |
| Itga8 | EFG | ABCDH |
| Gpr30 | EFG | ABCDH |
| 2410187C16Rik | EFG | ABCDH |
| Zc3h14 | EFG | ABCDH |
| 1700021K19Rik | EFG | ABCDH |
| Ubqln1 | EFG | ABCDH |
| Tbc1d8b | EFG | ABCDH |
| Ulk2 | EFG | ABCDH |
| Cog6 | EFG | ABCDH |
| Arl3 | EFG | ABCDH |
| Nipa1 | EFG | ABCDH |
| Sec14l1 | EFG | ABCDH |
| Btbd2 | EFG | ABCDH |
| Ankrd46 | EFG | ABCDH |
| 2700094K13Rik | EFG | ABCDH |
| 4732479N06Rik | EFG | ABCDH |
| Klhdc8b | EFG | ABCDH |
| Sh3md4 | EFG | ABCDH |
| D15Ertd621e | EFG | ABCDH |
| Ecm2 | EFG | ABCDH |
| Tns3 | EFG | ABCDH |
| Pde8b | EFG | ABCDH |
| Lrba | EFG | ABCDH |
| Hes6 | EFG | ABCDH |
| 3732413I11Rik | EFG | ABCDH |
| 2700049A03Rik | EFG | ABCDH |
| Cbx6 | EFG | ABCDH |
| Wnt3a | EFG | ABCDH |
| Ttc28 | EFG | ABCDH |
| Tmem175 | EFG | ABCDH |
| Rbm10 | EFG | ABCDH |
| Park7 | EFG | ABCDH |
| Setd3 | EFG | ABCDH |
| Uhrf2 | EFG | ABCDH |
| D6Wsu176e | EFG | ABCDH |
| Ebp | EFG | ABCDH |
| Thap11 | EFG | ABCDH |
| Ogn | EFG | ABCDH |
| Mapk8ip1 | EFG | ABCDH |
| Wwtr1 | EFG | ABCDH |
| Hirip3 | EFG | ABCDH |
| RP23-195K8.6 | EFG | ABCDH |
| AU020772 | EFG | ABCDH |
| Gmcl1 | EFG | ABCDH |
| Ltbp3 | EFG | ABCDH |
| B930008K04Rik | EFG | ABCDH |
| BC054438 | EFG | ABCDH |
| Itfg1 | EFG | ABCDH |
| Tusc3 | EFG | ABCDH |
| Wwtr1 | EFG | ABCDH |
| Rbp1 | EFG | ABCDH |
| Ndufb2 | EFG | ABCDH |
| Fchsd2 | EFG | ABCDH |
| 1810034K20Rik | EFG | ABCDH |
| Slc39a13 | EFG | ABCDH |
| E130308A19Rik | EFG | ABCDH |
| Eif4a2 | EFG | ABCDH |
| Mpv17l | EFG | ABCDH |
| Vgll4 | EFG | ABCDH |
| Cep164 | EFG | ABCDH |
| 4732415M23Rik | EFG | ABCDH |
| Fbxo3 | EFG | ABCDH |
| Nfia | EFG | ABCDH |
| Kdelc2 | EFG | ABCDH |
| B230333C21Rik | EFG | ABCDH |
| Tpcn1 | EFG | ABCDH |
| Pdcd6ip | EFG | ABCDH |
| Foxo6 | EFG | ABCDH |
| Smc1a | EFG | ABCDH |
| Caprin2 | EFG | ABCDH |
| 1110031B06Rik | EFG | ABCDH |
| Pdgfd | EFG | ABCDH |
| C1qtnf2 | EFG | ABCDH |
| Bloc1s1 | EFG | ABCDH |
| Pvrl3 | EFG | ABCDH |
| Bpgm | EFG | ABCDH |
| 2310016C16Rik | EFG | ABCDH |
| Ccdc117 | EFG | ABCDH |
| Anapc5 | EFG | ABCDH |
| Atg16l2 | EFG | ABCDH |
| Ap1m2 | EFG | ABCDH |
| Mphosph9 | EFG | ABCDH |
| Tmem9 | EFG | ABCDH |
| Nbr1 | EFG | ABCDH |
| Sh3glb1 | EFG | ABCDH |
| Vpreb3 | EFG | ABCDH |
| Plekha6 | EFG | ABCDH |
| Nme7 | EFG | ABCDH |
| Capn7 | EFG | ABCDH |
| Kitl | EFG | ABCDH |
| Cbfa2t3h | EFG | ABCDH |
| Azi1 | EFG | ABCDH |
| 2310033P09Rik | EFG | ABCDH |
| Pts | EFG | ABCDH |
| AK034355 | EFG | ABCDH |
| Afap1l1 | EFG | ABCDH |
| Kdelc2 | EFG | ABCDH |
| Id4 | EFG | ABCDH |
| Aga | EFG | ABCDH |
| Slc2a13 | EFG | ABCDH |
| Dnalc4 | EFG | ABCDH |
| NAP026388-1 | EFG | ABCDH |
| 2510048L02Rik | EFG | ABCDH |
| Scap | EFG | ABCDH |
| Pias2 | EFG | ABCDH |
| Tmem64 | EFG | ABCDH |
| 1110003E01Rik | EFG | ABCDH |
| Ptprd | EFG | ABCDH |
| Dennd2a | EFG | ABCDH |
| Lass5 | EFG | ABCDH |
| ENSMUSG00000052439 | EFG | ABCDH |
| Tmc4 | EG | ABCDFH |
| Rnf186 | EG | ABCDFH |
| Bbs9 | EG | ABCDFH |
| Ccdc92 | EG | ABCDFH |
| 6330439K17Rik | EG | ABCDFH |
| Apoa1bp | EG | ABCDFH |
| 4930430E16Rik | EG | ABCDFH |
| 6330505N24Rik | EG | ABCDFH |
| Wbp1 | EG | ABCDFH |
| 2610028H24Rik | EG | ABCDFH |
| D4Bwg0951e | EG | ABCDFH |
| Pamci | EG | ABCDFH |
| Hspa4l | EG | ABCDFH |
| AK036787 | EG | ABCDFH |
| Slc25a10 | EG | ABCDFH |
| Dync2h1 | EG | ABCDFH |
| Stk36 | EG | ABCDFH |
| Wdr35 | EG | ABCDFH |
| Tekt2 | EG | ABCDFH |
| 5330417C22Rik | EG | ABCDFH |
| Slc7a4 | EG | ABCDFH |
| Lrrc23 | EG | ABCDFH |
| Faah | EG | ABCDFH |
| BE650457 | EG | ABCDFH |
| Hspa4l | EG | ABCDFH |
| Hspa4l | EG | ABCDFH |
| ENSMUST00000050829 | EG | ABCDFH |
| Gm166 | EG | ABCDFH |
| Pop5 | EG | ABCDFH |
| Ptplad1 | EG | ABCDFH |
| Pkp2 | EG | ABCDFH |
| Abcd3 | EG | ABCDFH |
| Usp33 | EG | ABCDFH |
| Usp33 | EG | ABCDFH |
| Gtl3 | EG | ABCDFH |
| Wdr13 | EG | ABCDFH |
| Sec14l2 | EG | ABCDFH |
| 1700040I03Rik | EG | ABCDFH |
| ENSMUST00000030142 | EG | ABCDFH |
| Pcnt | EG | ABCDFH |
| Fhad1 | EG | ABCDFH |
| Stx19 | EG | ABCDFH |
| Ank3 | EG | ABCDFH |
| Tlcd1 | EG | ABCDFH |
| Arhgef4 | EG | ABCDFH |
| Ank3 | EG | ABCDFH |
| Lnx1 | EG | ABCDFH |
| D630039A03Rik | EG | ABCDFH |
| Adk | EG | ABCDFH |
| 4932425I24Rik | EG | ABCDFH |
| Lrba | EG | ABCDFH |
| Snx25 | EG | ABCDFH |
| Abcd3 | EG | ABCDFH |
| Sec14l3 | EG | ABCDFH |
| Atpif1 | EG | ABCDFH |
| Zfp219 | EG | ABCDFH |
| Lrp2 | EG | ABCDFH |
| Ovol2 | EG | ABCDFH |
| 2900041A09Rik | EG | ABCDFH |
| Ptprf | EG | ABCDFH |
| Oaz2 | EG | ABCDFH |
| 2310057J16Rik | EG | ABCDFH |
| Cryl1 | EG | ABCDFH |
| Lztr1 | EG | ABCDFH |
| Mir16 | EG | ABCDFH |
| Lrp2 | EG | ABCDFH |
| Wdr90 | EG | ABCDFH |
| Ak7 | EG | ABCDFH |
| Ropn1l | EG | ABCDFH |
| Dnaja4 | EG | ABCDFH |
| Tspan12 | EG | ABCDFH |
| BC059842 | EG | ABCDFH |
| Alcam | EG | ABCDFH |
| Cobll1 | EG | ABCDFH |
| Slc23a1 | EG | ABCDFH |
| Dpp4 | EG | ABCDFH |
| Anapc11 | EG | ABCDFH |
| Ppp1r16a | EG | ABCDFH |
| Tekt1 | EG | ABCDFH |
| Enpep | EG | ABCDFH |
| Akap9 | EG | ABCDFH |
| Rad21 | EG | ABCDFH |
| Csad | EG | ABCDFH |
| Phf17 | EG | ABCDFH |
| Rassf7 | EG | ABCDFH |
| Cdc25b | EG | ABCDFH |
| Ppm1b | EG | ABCDFH |
| Mycbp | EG | ABCDFH |
| Heca | EG | ABCDFH |
| Rnf43 | EG | ABCDFH |
| Tmem109 | EG | ABCDFH |
| Ptov1 | EG | ABCDFH |
| Dixdc1 | EG | ABCDFH |
| Lnx1 | EG | ABCDFH |
| D230025D16Rik | EG | ABCDFH |
| AW061290 | EG | ABCDFH |
| Ptprf | EG | ABCDFH |
| Oxct1 | EG | ABCDFH |
| 3110050N22Rik | EG | ABCDFH |
| AV249152 | EG | ABCDFH |
| 1700001L19Rik | EG | ABCDFH |
| 1110017D15Rik | EG | ABCDFH |
| Hcfc1r1 | EG | ABCDFH |
| Sema3b | EG | ABCDFH |
| Dpp4 | EG | ABCDFH |
| Rnf32 | EG | ABCDFH |
| Ptprf | EG | ABCDFH |
| AV249152 | EG | ABCDFH |
| Ripk4 | EG | ABCDFH |
| 3110004L20Rik | EG | ABCDFH |
| Gtf3c1 | EG | ABCDFH |
| Gtf2i | EG | ABCDFH |
| Glb1l | EG | ABCDFH |
| Plekhb1 | EG | ABCDFH |
| AK048091 | EG | ABCDFH |
| Hey1 | EG | ABCDFH |
| Tbcel | EG | ABCDFH |
| Pgm2l1 | EG | ABCDFH |
| Faah | EG | ABCDFH |
| Pja1 | EG | ABCDFH |
| Pop5 | EG | ABCDFH |
| Med25 | EG | ABCDFH |
| Tle6 | EG | ABCDFH |
| Prepl | EG | ABCDFH |
| Sec14l3 | EG | ABCDFH |
| Gstt2 | EG | ABCDFH |
| AK087708 | EG | ABCDFH |
| Serinc5 | EG | ABCDFH |
| Oaz1 | EG | ABCDFH |
| Sccpdh | EG | ABCDFH |
| Celsr1 | EG | ABCDFH |
| Plch2 | EG | ABCDFH |
| Irs1 | EG | ABCDFH |
| 5830434P21Rik | EG | ABCDFH |
| Wdr35 | EG | ABCDFH |
| Rbm35b | EG | ABCDFH |
| Fasn | EG | ABCDFH |
| Trp53bp2 | EG | ABCDFH |
| Il17re | EG | ABCDFH |
| Phf3 | EG | ABCDFH |
| Mid1ip1 | EG | ABCDFH |
| Mmp11 | EG | ABCDFH |
| Abcd3 | EG | ABCDFH |
| Smarca2 | EG | ABCDFH |
| Ift81 | EG | ABCDFH |
| Ppid | EG | ABCDFH |
| Hnrph3 | EG | ABCDFH |
| Fhl1 | EG | ABCDFH |
| Ift80 | EG | ABCDFH |
| Dock7 | EG | ABCDFH |
| Bola1 | EG | ABCDFH |
| C77370 | EG | ABCDFH |
| Ivd | EG | ABCDFH |
| 1810019J16Rik | EG | ABCDFH |
| Foxp2 | EG | ABCDFH |
| Thrb | EG | ABCDFH |
| Ces3 | EG | ABCDFH |
| Tmem42 | EG | ABCDFH |
| Fgf1 | EG | ABCDFH |
| Pik3ca | EG | ABCDFH |
| 2900010J23Rik | EG | ABCDFH |
| Sort1 | EG | ABCDFH |
| Ankrd44 | EG | ABCDFH |
| Lamp1 | EG | ABCDFH |
| A930008G19Rik | EG | ABCDFH |
| Elovl1 | EG | ABCDFH |
| 5330417C22Rik | EG | ABCDFH |
| 1810007P19Rik | EG | ABCDFH |
| Ift81 | EG | ABCDFH |
| 2810410M20Rik | EG | ABCDFH |
| Cyb5b | EG | ABCDFH |
| Trp53bp2 | EG | ABCDFH |
| 4921511K06Rik | EG | ABCDFH |
| Cobl | EG | ABCDFH |
| Ppfia1 | EG | ABCDFH |
| AK082505 | EG | ABCDFH |
| Mcee | EG | ABCDFH |
| Ypel1 | EG | ABCDFH |
| AW061290 | EG | ABCDFH |
| Dusp14 | EG | ABCDFH |
| Rnf32 | EG | ABCDFH |
| Slc25a36 | EG | ABCDFH |
| Hsd17b4 | EG | ABCDFH |
| Lamp3 | EG | ABCDFH |
| Gnpat | EG | ABCDFH |
| Muc1 | EG | ABCDFH |
| Btbd3 | EG | ABCDFH |
| Cpm | EG | ABCDFH |
| Usp46 | EG | ABCDFH |
| B230317C12Rik | EG | ABCDFH |
| Mthfd1 | EG | ABCDFH |
| Pcx | EG | ABCDFH |
| Cplx2 | EG | ABCDFH |
| Csad | EG | ABCDFH |
| Paip2 | EG | ABCDFH |
| Hipk3 | EG | ABCDFH |
| 1190002J23Rik | EG | ABCDFH |
| Gss | EG | ABCDFH |
| Pcmtd2 | EG | ABCDFH |
| Spnb2 | EG | ABCDFH |
| Tmem63b | EG | ABCDFH |
| AK199677 | EG | ABCDFH |
| Cadm1 | EG | ABCDFH |
| Fzd3 | EG | ABCDFH |
| Tinag | EG | ABCDFH |
| Stard10 | EG | ABCDFH |
| Klhl8 | EG | ABCDFH |
| Cars2 | EG | ABCDFH |
| Cox6c | EG | ABCDFH |
| Gkap1 | EG | ABCDFH |
| Vamp5 | EG | ABCDFH |
| AK052002 | EG | ABCDFH |
| Etv1 | EG | ABCDFH |
| Sort1 | EG | ABCDFH |
| Acvr2b | EG | ABCDFH |
| Rab11fip3 | EG | ABCDFH |
| Pcsk6 | EG | ABCDFH |
| Klc3 | EG | ABCDFH |
| Phkb | EG | ABCDFH |
| Mtch1 | EG | ABCDFH |
| Cldn3 | EG | ABCDFH |
| 2310045A20Rik | EG | ABCDFH |
| Deb1 | EG | ABCDFH |
| Sort1 | EG | ABCDFH |
| 2010003O02Rik | EG | ABCDFH |
| Nucb1 | EG | ABCDFH |
| Atrn | EG | ABCDFH |
| Plxdc2 | EG | ABCDFH |
| Mbtps1 | EG | ABCDFH |
| Rap1gap | EG | ABCDFH |
| E330009J07Rik | EG | ABCDFH |
| Tle2 | EG | ABCDFH |
| 2900024O10Rik | EG | ABCDFH |
| Mobkl2b | EG | ABCDFH |
| 1110032A03Rik | EG | ABCDFH |
| Srebf1 | EG | ABCDFH |
| Unc84a | EG | ABCDFH |
| Sema3e | EG | ABCDFH |
| Alas1 | EG | ABCDFH |
| Sdpr | EG | ABCDFH |
| Mrpl49 | EG | ABCDFH |
| Xpo7 | EG | ABCDFH |
| Cnot6 | EG | ABCDFH |
| Tmem41a | EG | ABCDFH |
| Ccpg1 | EG | ABCDFH |
| Tspan3 | EG | ABCDFH |
| Idh2 | EG | ABCDFH |
| Gpx4 | EG | ABCDFH |
| 2210013O21Rik | EG | ABCDFH |
| Muc1 | EG | ABCDFH |
| Xpc | EG | ABCDFH |
| Tmem106b | EG | ABCDFH |
| 2900024O10Rik | EG | ABCDFH |
| Mme | EG | ABCDFH |
| Tspan9 | EG | ABCDFH |
| Fsip1 | EG | ABCDFH |
| Aldh3b1 | EG | ABCDFH |
| Crip3 | EG | ABCDFH |
| Pxmp4 | EG | ABCDFH |
| Irx1 | EG | ABCDFH |
| Anks1 | EG | ABCDFH |
| Arhgef12 | EG | ABCDFH |
| Spry1 | EG | ABCDFH |
| Strbp | EG | ABCDFH |
| Fuk | EG | ABCDFH |
| Rmnd5a | EG | ABCDFH |
| Mid2 | EG | ABCDFH |
| Ankrd5 | EG | ABCDFH |
| Ndst1 | EG | ABCDFH |
| Map1lc3a | EG | ABCDFH |
| Pbx3 | EG | ABCDFH |
| Rmnd5a | EG | ABCDFH |
| Dyrk1b | EG | ABCDFH |
| 2700078K21Rik | EG | ABCDFH |
| Sp5 | EG | ABCDFH |
| BC031353 | EG | ABCDFH |
| Slc34a2 | EG | ABCDFH |
| Lamp3 | EG | ABCDFH |
| Dad1 | EG | ABCDFH |
| Myo6 | EG | ABCDFH |
| 1700020I14Rik | EG | ABCDFH |
| Sel1l | EG | ABCDFH |
| Nek9 | EG | ABCDFH |
| Zfp612 | EG | ABCDFH |
| Cd9 | EG | ABCDFH |
| Dmn | EG | ABCDFH |
| Dynlt3 | EG | ABCDFH |
| Scrn3 | EG | ABCDFH |
| Btbd3 | EG | ABCDFH |
| 1810021J13Rik | EG | ABCDFH |
| 1190002N15Rik | EG | ABCDFH |
| Clcn3 | EG | ABCDFH |
| Cdadc1 | EG | ABCDFH |
| Cadm1 | EG | ABCDFH |
| Hrsp12 | EG | ABCDFH |
| Cab39l | EG | ABCDFH |
| Myo1d | EG | ABCDFH |
| Setx | EG | ABCDFH |
| Eif2c4 | EG | ABCDFH |
| Hmcn1 | EG | ABCDFH |
| Nrbp2 | EG | ABCDFH |
| Twsg1 | EG | ABCDFH |
| 2210018M11Rik | EG | ABCDFH |
| Phtf1 | EG | ABCDFH |
| Arid2 | EG | ABCDFH |
| Cox8a | EG | ABCDFH |
| Slc24a3 | EG | ABCDFH |
| Rev3l | EG | ABCDFH |
| Zrsr1 | EG | ABCDFH |
| Helz | EG | ABCDFH |
| 4631427C17Rik | EG | ABCDFH |
| Slc9a6 | EG | ABCDFH |
| Yipf2 | EG | ABCDFH |
| Bnip3l | EG | ABCDFH |
| Map3k13 | EG | ABCDFH |
| Ccs | EG | ABCDFH |
| Acsl5 | EG | ABCDFH |
| Macf1 | EG | ABCDFH |
| Lamp3 | EG | ABCDFH |
| Magi3 | EG | ABCDFH |
| Myh7 | EG | ABCDFH |
| Wdr45 | EG | ABCDFH |
| AK078885 | EG | ABCDFH |
| Ica1 | EG | ABCDFH |
| BF642829 | EG | ABCDFH |
| AI464131 | EG | ABCDFH |
| Nsbp1 | EG | ABCDFH |
| Tnfrsf19 | EG | ABCDFH |
| Cobll1 | EG | ABCDFH |
| Bptf | EG | ABCDFH |
| Daam2 | EG | ABCDFH |
| Ndfip1 | EG | ABCDFH |
| 2210011C24Rik | EG | ABCDFH |
| Abca3 | EG | ABCDFH |
| Pmp22 | EG | ABCDFH |
| 2700049A03Rik | EG | ABCDFH |
| Itga8 | EG | ABCDFH |
| Ptrf | EG | ABCDFH |
| Fmo5 | EG | ABCDFH |
| Plcg1 | EG | ABCDFH |
| Tmc4 | EG | ABCDFH |
| Ndufa6 | EG | ABCDFH |
| Psd3 | EG | ABCDFH |
| Npr3 | EG | ABCDFH |
| Gpr108 | EG | ABCDFH |
| Asb1 | EG | ABCDFH |
| D730040F13Rik | EG | ABCDFH |
| Ttbk2 | EG | ABCDFH |
| Nbeal1 | EG | ABCDFH |
| Pard3 | EG | ABCDFH |
| Ypel3 | EG | ABCDFH |
| Emp2 | EG | ABCDFH |
| Ift140 | EG | ABCDFH |
| Fancb | EG | ABCDFH |
| Map1lc3b | EG | ABCDFH |
| Etv5 | EG | ABCDFH |
| Brd3 | EG | ABCDFH |
| Zfp101 | EG | ABCDFH |
| Mtss1 | EG | ABCDFH |
| Mdh1b | EG | ABCDFH |
| Rbm35a | EG | ABCDFH |
| Acox1 | EG | ABCDFH |
| Elovl5 | EG | ABCDFH |
| Clic3 | EG | ABCDFH |
| Megf9 | EG | ABCDFH |
| Zbtb4 | EG | ABCDFH |
| Parva | EG | ABCDFH |
| Mib1 | EG | ABCDFH |
| Zfp2 | EG | ABCDFH |
| Cav1 | EG | ABCDFH |
| Eif2c1 | EG | ABCDFH |
| Aldh3a2 | EG | ABCDFH |
| Usp34 | EG | ABCDFH |
| Vps13b | EG | ABCDFH |
| Acaa1a | EG | ABCDFH |
| Ccni | EG | ABCDFH |
| Dnm3 | EG | ABCDFH |
| Tspan2 | EG | ABCDFH |
| LOC627873 | EG | ABCDFH |
| Abca3 | EG | ABCDFH |
| Ptprd | EG | ABCDFH |
| Fat4 | EG | ABCDFH |
| Ctdspl | EG | ABCDFH |
| Park2 | EG | ABCDFH |
| Top2b | EG | ABCDFH |
| Sftpa1 | EG | ABCDFH |
| Arhgap12 | EG | ABCDFH |
| Specc1l | EG | ABCDFH |
| Mme | EG | ABCDFH |
| Frat1 | EG | ABCDFH |
| 1500011H22Rik | EG | ABCDFH |
| D930005D10Rik | EG | ABCDFH |
| Cybrd1 | EG | ABCDFH |
| 5730601F06Rik | EG | ABCDFH |
| Huwe1 | EG | ABCDFH |
| 9630058J23Rik | EG | ABCDFH |
| Apc | EG | ABCDFH |
| Ppm1b | EG | ABCDFH |
| Nfia | EG | ABCDFH |
| D12Ertd647e | EG | ABCDFH |
| Thbd | EG | ABCDFH |
| Pnpla8 | EG | ABCDFH |
| Mxi1 | EG | ABCDFH |
| Purg | EG | ABCDFH |
| Opa1 | EG | ABCDFH |
| Smurf2 | EG | ABCDFH |
| Sf3b2 | EG | ABCDFH |
| Pcolce2 | EG | ABCDFH |
| Aktip | EG | ABCDFH |
| Dync1li2 | EG | ABCDFH |
| Mgst1 | EG | ABCDFH |
| Setd7 | EG | ABCDFH |
| Nbeal1 | EG | ABCDFH |
| Pxmp4 | EG | ABCDFH |
| Atxn2 | EG | ABCDFH |
| 1700022C21Rik | EG | ABCDFH |
| Epm2aip1 | EG | ABCDFH |
| Uqcrh | EG | ABCDFH |
| Prkaca | EG | ABCDFH |
| Cdc42bpa | EG | ABCDFH |
| Klhl17 | EG | ABCDFH |
| Cbx7 | EG | ABCDFH |
| Klhl7 | EG | ABCDFH |
| Fgfr4 | EG | ABCDFH |
| BC039093 | EG | ABCDFH |
| Ttc28 | EG | ABCDFH |
| Chchd6 | EG | ABCDFH |
| 1110059E24Rik | EG | ABCDFH |
| Ankrd5 | EG | ABCDFH |
| 1810008I18Rik | EG | ABCDFH |
| Hisppd2a | EG | ABCDFH |
| Tbc1d19 | EG | ABCDFH |
| Ift20 | EG | ABCDFH |
| Rnf187 | EG | ABCDFH |
| 4-Sep | EG | ABCDFH |
| Slc1a4 | EG | ABCDFH |
| Acp6 | EG | ABCDFH |
| Cd81 | EG | ABCDFH |
| Rb1cc1 | EG | ABCDFH |
| Aox3 | EG | ABCDFH |
| Arhgap21 | EG | ABCDFH |
| Slc34a2 | EG | ABCDFH |
| Nbl1 | EG | ABCDFH |
| Gm684 | EG | ABCDFH |
| Prkaa2 | EG | ABCDFH |
| Zmym2 | EG | ABCDFH |
| Atp5j2 | EG | ABCDFH |
| Phactr1 | EG | ABCDFH |
| Cebpa | EG | ABCDFH |
| Kif16b | EG | ABCDFH |
| Tspan18 | EG | ABCDFH |
| Myh10 | EG | ABCDFH |
| Zfand6 | EG | ABCDFH |
| Cd97 | EG | ABCDFH |
| Sox18 | EG | ABCDFH |
| Pik3ca | EG | ABCDFH |
| Fat1 | EG | ABCDFH |
| Zmat3 | EG | ABCDFH |
| Xpc | EG | ABCDFH |
| Hint2 | EG | ABCDFH |
| Ccpg1 | EG | ABCDFH |
| Rutbc1 | EG | ABCDFH |
| Mpp5 | EG | ABCDFH |
| 4933404M19Rik | EG | ABCDFH |
| Wwp1 | EG | ABCDFH |
| AI462493 | EG | ABCDFH |
| Ddc | EG | ABCDFH |
| Gcat | EG | ABCDFH |
| Cugbp2 | EG | ABCDFH |
| Uqcr | EG | ABCDFH |
| Epas1 | EG | ABCDFH |
| Pftk1 | EG | ABCDFH |
| Chd3 | EG | ABCDFH |
| Gna11 | EG | ABCDFH |
| Myo1b | EG | ABCDFH |
| Sned1 | EG | ABCDFH |
| Cyp39a1 | EG | ABCDFH |
| Rab25 | EG | ABCDFH |
| Tmem29 | EG | ABCDFH |
| Mapk15 | EG | ABCDFH |
| Igf1r | EG | ABCDFH |
| Ptgfr | EG | ABCDFH |
| Vps13d | EG | ABCDFH |
| Rnase4 | EG | ABCDFH |
| 4931406C07Rik | EG | ABCDFH |
| Atp5h | EG | ABCDFH |
| Zbtb20 | EG | ABCDFH |
| Pkd1 | EG | ABCDFH |
| Hmg20a | EG | ABCDFH |
| Mapk1ip1 | EG | ABCDFH |
| Adcy9 | EG | ABCDFH |
| 2900009J20Rik | EG | ABCDFH |
| Npr3 | EG | ABCDFH |
| Lsmd1 | EG | ABCDFH |
| Pxmp2 | EG | ABCDFH |
| Tcp11l2 | EG | ABCDFH |
| Gcap14 | EG | ABCDFH |
| Setd7 | EG | ABCDFH |
| Zfp474 | EG | ABCDFH |
| Dynll2 | EG | ABCDFH |
| Eif2c1 | EG | ABCDFH |
| Cep63 | EG | ABCDFH |
| Dnajc3a | EG | ABCDFH |
| Serinc1 | EG | ABCDFH |
| Dexi | EG | ABCDFH |
| Cttnbp2 | EG | ABCDFH |
| Yy1 | EG | ABCDFH |
| Fzd7 | EG | ABCDFH |
| Klhl24 | EG | ABCDFH |
| Akap11 | EG | ABCDFH |
| Arhgef12 | EG | ABCDFH |
| Serpina9 | EG | ABCDFH |
| Dsp | EG | ABCDFH |
| Arid4b | EG | ABCDFH |
| AK086814 | EG | ABCDFH |
| Ttc23 | EG | ABCDFH |
| Phactr1 | EG | ABCDFH |
| Por | EG | ABCDFH |
| Slitrk6 | EG | ABCDFH |
| Hdac7a | EG | ABCDFH |
| Scamp1 | EG | ABCDFH |
| AI597479 | EG | ABCDFH |
| Mosc2 | EG | ABCDFH |
| Erbb3 | EG | ABCDFH |
| Ociad2 | EG | ABCDFH |
| AU042671 | EG | ABCDFH |
| Prkcn | EG | ABCDFH |
| Ppp2r5c | EG | ABCDFH |
| Abi2 | EG | ABCDFH |
| D430042O09Rik | EG | ABCDFH |
| Sftpa1 | EG | ABCDFH |
| Smurf2 | EG | ABCDFH |
| Dhrs13 | EG | ABCDFH |
| Wwp1 | EG | ABCDFH |
| C030015A19Rik | EG | ABCDFH |
| Heca | EG | ABCDFH |
| Tmem165 | EG | ABCDFH |
| Ankrd54 | EG | ABCDFH |
| Prickle1 | EG | ABCDFH |
| Tyro3 | EG | ABCDFH |
| Ndst1 | EG | ABCDFH |
| Rnase4 | EG | ABCDFH |
| Fdx1 | EG | ABCDFH |
| Bcas3 | EG | ABCDFH |
| Ulk1 | EG | ABCDFH |
| Homez | EG | ABCDFH |
| Yipf2 | EG | ABCDFH |
| LOC671029 | EG | ABCDFH |
| Cutl1 | EG | ABCDFH |
| Zfp524 | EG | ABCDFH |
| Dnajc3a | EG | ABCDFH |
| Aldh2 | EG | ABCDFH |
| Kif21a | EG | ABCDFH |
| Clock | EG | ABCDFH |
| Ncoa6 | EG | ABCDFH |
| Cldn7 | EG | ABCDFH |
| Sod1 | EG | ABCDFH |
| Ccdc46 | EG | ABCDFH |
| Gm166 | EG | ABCDFH |
| Scube2 | EG | ABCDFH |
| D1Ertd161e | EG | ABCDFH |
| D130059P03Rik | EG | ABCDFH |
| Zbtb20 | EG | ABCDFH |
| 2410127L17Rik | EG | ABCDFH |
| 4933439F18Rik | EG | ABCDFH |
| Aldh6a1 | EG | ABCDFH |
| Gpr175 | EG | ABCDFH |
| BC043118 | EG | ABCDFH |
| Cbfa2t3h | EG | ABCDFH |
| Cds2 | EG | ABCDFH |
| Fbln1 | EG | ABCDFH |
| Slc5a3 | EG | ABCDFH |
| Lonp2 | EG | ABCDFH |
| Syne1 | EG | ABCDFH |
| Ogt | EG | ABCDFH |
| Upf3b | EG | ABCDFH |
| Clec14a | EG | ABCDFH |
| Lims2 | EG | ABCDFH |
| Specc1l | EG | ABCDFH |
| AK054054 | EG | ABCDFH |
| Man2c1 | EG | ABCDFH |
| Atrnl1 | EG | ABCDFH |
| 2310067B10Rik | EG | ABCDFH |
| Spna2 | EG | ABCDFH |
| Xpr1 | EG | ABCDFH |
| Unc50 | EG | ABCDFH |
| Tgoln1 | EG | ABCDFH |
| Taf4a | EG | ABCDFH |
| Deb1 | EG | ABCDFH |
| Fbxl17 | EG | ABCDFH |
| 1200016B10Rik | EG | ABCDFH |
| Ica1 | EG | ABCDFH |
| Tmem100 | EG | ABCDFH |
| Tnrc6b | EG | ABCDFH |
| 6720456B07Rik | EG | ABCDFH |
| Hlcs | F | ABCDEGH |
| AK050788 | F | ABCDEGH |
| 1190002A17Rik | G | ABCDEFH |
| Ldhb | G | ABCDEFH |
| Ngef | G | ABCDEFH |
| Stk36 | G | ABCDEFH |
| C230082I21Rik | G | ABCDEFH |
| Lrrfip2 | G | ABCDEFH |
| Ndufb11 | G | ABCDEFH |
| Pcp4l1 | G | ABCDEFH |
| 2900046G09Rik | G | ABCDEFH |
| 2010007H06Rik | G | ABCDEFH |
| Ttc18 | G | ABCDEFH |
| Cds1 | G | ABCDEFH |
| AK033818 | G | ABCDEFH |
| Reep6 | G | ABCDEFH |
| Oaz2 | G | ABCDEFH |
| Dact2 | G | ABCDEFH |
| 9830169C18Rik | G | ABCDEFH |
| Itpr1 | G | ABCDEFH |
| Wrb | G | ABCDEFH |
| Mtus1 | G | ABCDEFH |
| C85492 | G | ABCDEFH |
| Rftn2 | G | ABCDEFH |
| Tcea3 | G | ABCDEFH |
| Dalrd3 | G | ABCDEFH |
| Wdr78 | G | ABCDEFH |
| Mapre3 | G | ABCDEFH |
| Dhrs3 | G | ABCDEFH |
| Acss2 | G | ABCDEFH |
| 5330431N19Rik | G | ABCDEFH |
| 2900034E22Rik | G | ABCDEFH |
| 2600009E05Rik | G | ABCDEFH |
| Ppfibp2 | G | ABCDEFH |
| Zmynd12 | G | ABCDEFH |
| E230008N13Rik | G | ABCDEFH |
| Efhb | G | ABCDEFH |
| 4833436C18Rik | G | ABCDEFH |
| Syne2 | G | ABCDEFH |
| 4930535E21Rik | G | ABCDEFH |
| Lama3 | G | ABCDEFH |
| Stim1 | G | ABCDEFH |
| Ptpn13 | G | ABCDEFH |
| Sh3rf1 | G | ABCDEFH |
| ENSMUST00000032357 | G | ABCDEFH |
| Dcun1d2 | G | ABCDEFH |
| 1700019E19Rik | G | ABCDEFH |
| Mapbpip | G | ABCDEFH |
| Bace2 | G | ABCDEFH |
| Gstz1 | G | ABCDEFH |
| Zfp219 | G | ABCDEFH |
| Map3k4 | G | ABCDEFH |
| Pick1 | G | ABCDEFH |
| Crip1 | G | ABCDEFH |
| S100a1 | G | ABCDEFH |
| Pir | G | ABCDEFH |
| Lrrc51 | G | ABCDEFH |
| 2810002I04Rik | G | ABCDEFH |
| Tom1l1 | G | ABCDEFH |
| BC004853 | G | ABCDEFH |
| AK045702 | G | ABCDEFH |
| Mgst1 | G | ABCDEFH |
| Tusc2 | G | ABCDEFH |
| Trim3 | G | ABCDEFH |
| Fem1b | G | ABCDEFH |
| Efemp1 | G | ABCDEFH |
| Mtmr4 | G | ABCDEFH |
| Adrbk2 | G | ABCDEFH |
| Cspp1 | G | ABCDEFH |
| Arhgap18 | G | ABCDEFH |
| Mccc2 | G | ABCDEFH |
| Klhl13 | G | ABCDEFH |
| Phlda2 | G | ABCDEFH |
| Vegfa | G | ABCDEFH |
| Npal2 | G | ABCDEFH |
| Lrig1 | G | ABCDEFH |
| Cxcl15 | G | ABCDEFH |
| Aes | G | ABCDEFH |
| 4933406E20Rik | G | ABCDEFH |
| Es22 | G | ABCDEFH |
| 2810423A18Rik | G | ABCDEFH |
| Gpsn2 | G | ABCDEFH |
| AK042559 | G | ABCDEFH |
| Ankrd15 | G | ABCDEFH |
| Ube2h | G | ABCDEFH |
| Dnase2a | G | ABCDEFH |
| Aktip | G | ABCDEFH |
| Foxj1 | G | ABCDEFH |
| Atp2c2 | G | ABCDEFH |
| A830007P12Rik | G | ABCDEFH |
| Slc12a2 | G | ABCDEFH |
| Eya1 | G | ABCDEFH |
| Lpin1 | G | ABCDEFH |
| Pygb | G | ABCDEFH |
| Tsc22d1 | G | ABCDEFH |
| Zfp664 | G | ABCDEFH |
| Rnf5 | G | ABCDEFH |
| 4932442K08Rik | G | ABCDEFH |
| Ubr2 | G | ABCDEFH |
| Bbs5 | G | ABCDEFH |
| A_51_P486046 | G | ABCDEFH |
| Akap8l | G | ABCDEFH |
| Gprasp1 | G | ABCDEFH |
| 2410091C18Rik | G | ABCDEFH |
| Met | G | ABCDEFH |
| Cdc14a | G | ABCDEFH |
| Alkbh7 | G | ABCDEFH |
| B230339M05Rik | G | ABCDEFH |
| Rragd | G | ABCDEFH |
| Arhgdig | G | ABCDEFH |
| Suclg1 | G | ABCDEFH |
| Hmgn3 | G | ABCDEFH |
| Tmem93 | G | ABCDEFH |
| Garnl1 | G | ABCDEFH |
| Pdcd4 | G | ABCDEFH |
| Eml1 | G | ABCDEFH |
| 2810002I04Rik | G | ABCDEFH |
| 4732418C07Rik | G | ABCDEFH |
| 1110054M08Rik | G | ABCDEFH |
| Ccdc13 | G | ABCDEFH |
| Add1 | G | ABCDEFH |
| Pex26 | G | ABCDEFH |
| Prpf8 | G | ABCDEFH |
| 1810048J11Rik | G | ABCDEFH |
| B230219D22Rik | G | ABCDEFH |
| Dtnb | G | ABCDEFH |
| Mpv17 | G | ABCDEFH |
| Trim37 | G | ABCDEFH |
| Sms | G | ABCDEFH |
| Prpf40b | G | ABCDEFH |
| Tom1l2 | G | ABCDEFH |
| Ric8b | G | ABCDEFH |
| Ttll1 | G | ABCDEFH |
| Ppp6c | G | ABCDEFH |
| Dcun1d2 | G | ABCDEFH |
| Rage | G | ABCDEFH |
| Dlg1 | G | ABCDEFH |
| Bbs5 | G | ABCDEFH |
| 4732418C07Rik | G | ABCDEFH |
| Sord | G | ABCDEFH |
| Mmp28 | G | ABCDEFH |
| 4930444P10Rik | G | ABCDEFH |
| Ndufc1 | G | ABCDEFH |
| Acad11 | G | ABCDEFH |
| 2310028O11Rik | G | ABCDEFH |
| Pcca | G | ABCDEFH |
| AK142427 | G | ABCDEFH |
| Nr2f6 | G | ABCDEFH |

Allele groupings were determined by grouping alleles based on the largest differences between groups. A=A/J, B=C57BL/6J, C=129S1/SvImJ, D=NOD/ShiLtJ, E=NZO/HILtJ, F=CAST/EiJ, G=PWK/PhJ, H=WSB/EiJ
