# Supplementary material for: Modeling Host Genetic Regulation of Influenza Pathogenesis in the Collaborative Cross
Source: PLoS Pathog. 2013 Feb 28;9(2):e1003196. doi: 10.1371/journal.ppat.1003196 (PMC3585141; doi:10.1371/journal.ppat.1003196)
Supplement: Table S8 — GO-Term enrichment of eQTL that differentiate the CAST/EiJ Mx1 allele's effects. (DOCX) [file ppat.1003196.s014.docx]

| **Table S8. GO Term enrichment of eQTL that differentiate the CAST/EiJ *Mx1* allele's effects** | | | | |  |
| --- | --- | --- | --- | --- | --- |
| **Clustering** | **High Alleles** | **GO Term** | **BY FDR** |  |  |
| CAST/EiJ resistant | ABCDH | cellular response to cytokine stimulus | 0.005545 |  |  |
| CAST/EiJ resistant | ABCDH | response to cytokine stimulus | 0.005545 |  |  |
| CAST/EiJ resistant | ABCDH | inflammatory response | 0.016782 |  |  |
| CAST/EiJ resistant | ABCDH | alpha-beta T cell activation | 0.033248 |  |  |
| CAST/EiJ resistant | ABCDH | regulation of macromolecule metabolic process | 0.033248 |  |  |
| CAST/EiJ resistant | ABCDH | regulation of metabolic process | 0.035601 |  |  |
| CAST/EiJ resistant | ABCDH | regulation of primary metabolic process | 0.035601 |  |  |
| CAST/EiJ resistant | ABCDH | defense response | 0.035601 |  |  |
| CAST/EiJ resistant | ABCDH | regulation of cellular metabolic process | 0.035601 |  |  |
| CAST/EiJ resistant | ABCDH | regulation of gene expression | 0.035601 |  |  |
| CAST/EiJ susceptible | EG | cellular component organization at cellular level | 0.009829 |  |  |
| CAST/EiJ susceptible | EG | cellular component organization or biogenesis at cellular level | 0.019432 |  |  |
| CAST/EiJ susceptible | EG | regulation of cholesterol metabolic process | 0.024606 |  |  |
| CAST/EiJ susceptible | EG | positive regulation of cholesterol biosynthetic process | 0.027833 |  |  |
| CAST/EiJ susceptible | EG | positive regulation of cholesterol metabolic process | 0.027833 |  |  |
| CAST/EiJ susceptible | EG | nervous system development | 0.027833 |  |  |
| CAST/EiJ susceptible | EG | regulation of cholesterol biosynthetic process | 0.028491 |  |  |
| CAST/EiJ susceptible | ABCDFH | immune system process | 3.40E-18 |  |  |
| CAST/EiJ susceptible | ABCDFH | immune response | 9.35E-11 |  |  |
| CAST/EiJ susceptible | ABCDFH | cell activation | 7.28E-10 |  |  |
| CAST/EiJ susceptible | ABCDFH | leukocyte activation | 3.23E-08 |  |  |
| CAST/EiJ susceptible | ABCDFH | immune system development | 4.11E-08 |  |  |
| CAST/EiJ susceptible | ABCDFH | hemopoietic or lymphoid organ development | 9.47E-08 |  |  |
| CAST/EiJ susceptible | ABCDFH | hemopoiesis | 1.06E-07 |  |  |
| CAST/EiJ susceptible | ABCDFH | defense response | 1.06E-07 |  |  |
| CAST/EiJ susceptible | ABCDFH | response to other organism | 2.65E-07 |  |  |
| CAST/EiJ susceptible | ABCDFH | positive regulation of biological process | 3.60E-07 |  |  |
| CAST/EiJ susceptible | ABCDFH | response to biotic stimulus | 4.38E-07 |  |  |
| CAST/EiJ susceptible | ABCDFH | regulation of response to stimulus | 4.52E-07 |  |  |
| CAST/EiJ susceptible | ABCDFH | response to cytokine stimulus | 5.79E-07 |  |  |
| CAST/EiJ susceptible | ABCDFH | positive regulation of cellular process | 6.26E-07 |  |  |
| CAST/EiJ susceptible | ABCDFH | immune effector process | 1.01E-06 |  |  |
| CAST/EiJ susceptible | ABCDFH | regulation of immune system process | 2.22E-06 |  |  |
| CAST/EiJ susceptible | ABCDFH | innate immune response | 2.26E-06 |  |  |
| CAST/EiJ susceptible | ABCDFH | lymphocyte activation | 2.82E-06 |  |  |
| CAST/EiJ susceptible | ABCDFH | leukocyte differentiation | 6.35E-06 |  |  |
| CAST/EiJ susceptible | ABCDFH | cytokine production | 6.35E-06 |  |  |
| CAST/EiJ susceptible | ABCDFH | multi-organism process | 1.20E-05 |  |  |
| CAST/EiJ susceptible | ABCDFH | phagocytosis | 1.42E-05 |  |  |
| CAST/EiJ susceptible | ABCDFH | regulation of cytokine production | 1.88E-05 |  |  |
| CAST/EiJ susceptible | ABCDFH | cellular response to chemical stimulus | 2.77E-05 |  |  |
| CAST/EiJ susceptible | ABCDFH | cellular response to cytokine stimulus | 3.54E-05 |  |  |
| CAST/EiJ susceptible | ABCDFH | cellular process | 4.61E-05 |  |  |
| CAST/EiJ susceptible | ABCDFH | cell proliferation | 5.83E-05 |  |  |
| CAST/EiJ susceptible | ABCDFH | regulation of cell activation | 5.84E-05 |  |  |
| CAST/EiJ susceptible | ABCDFH | response to interferon-gamma | 9.24E-05 |  |  |
| CAST/EiJ susceptible | ABCDFH | cell migration | 0.000107 |  |  |
| CAST/EiJ susceptible | ABCDFH | inflammatory response | 0.000132 |  |  |
| CAST/EiJ susceptible | ABCDFH | response to stress | 0.000168 |  |  |
| CAST/EiJ susceptible | ABCDFH | response to bacterium | 0.000237 |  |  |
| CAST/EiJ susceptible | ABCDFH | intracellular signal transduction | 0.000273 |  |  |
| CAST/EiJ susceptible | ABCDFH | cell motility | 0.000301 |  |  |
| CAST/EiJ susceptible | ABCDFH | localization of cell | 0.000301 |  |  |
| CAST/EiJ susceptible | ABCDFH | response to organic substance | 0.000314 |  |  |
| CAST/EiJ susceptible | ABCDFH | myeloid leukocyte activation | 0.000326 |  |  |
| CAST/EiJ susceptible | ABCDFH | regulation of cell motility | 0.000358 |  |  |
| CAST/EiJ susceptible | ABCDFH | response to wounding | 0.00042 |  |  |
| CAST/EiJ susceptible | ABCDFH | biological regulation | 0.00042 |  |  |
| CAST/EiJ susceptible | ABCDFH | neutrophil activation involved in immune response | 0.000498 |  |  |
| CAST/EiJ susceptible | ABCDFH | regulation of programmed cell death | 0.000498 |  |  |
| CAST/EiJ susceptible | ABCDFH | regulation of cell migration | 0.000508 |  |  |
| CAST/EiJ susceptible | ABCDFH | regulation of leukocyte activation | 0.000521 |  |  |
| CAST/EiJ susceptible | ABCDFH | cellular component movement | 0.000521 |  |  |
| CAST/EiJ susceptible | ABCDFH | regulation of immune effector process | 0.000526 |  |  |
| CAST/EiJ susceptible | ABCDFH | neutrophil activation | 0.000558 |  |  |
| CAST/EiJ susceptible | ABCDFH | regulation of response to stress | 0.000589 |  |  |
| CAST/EiJ susceptible | ABCDFH | regulation of apoptotic process | 0.000708 |  |  |
| CAST/EiJ susceptible | ABCDFH | intracellular protein kinase cascade | 0.000747 |  |  |
| CAST/EiJ susceptible | ABCDFH | regulation of cellular component movement | 0.000874 |  |  |
| CAST/EiJ susceptible | ABCDFH | programmed cell death | 0.001065 |  |  |
| CAST/EiJ susceptible | ABCDFH | response to molecule of bacterial origin | 0.001101 |  |  |
| CAST/EiJ susceptible | ABCDFH | regulation of cell death | 0.001101 |  |  |
| CAST/EiJ susceptible | ABCDFH | regulation of locomotion | 0.001214 |  |  |
| CAST/EiJ susceptible | ABCDFH | cell death | 0.001286 |  |  |
| CAST/EiJ susceptible | ABCDFH | apoptotic process | 0.00133 |  |  |
| CAST/EiJ susceptible | ABCDFH | regulation of biological process | 0.001335 |  |  |
| CAST/EiJ susceptible | ABCDFH | death | 0.001376 |  |  |
| CAST/EiJ susceptible | ABCDFH | cell activation involved in immune response | 0.001376 |  |  |
| CAST/EiJ susceptible | ABCDFH | leukocyte activation involved in immune response | 0.001376 |  |  |
| CAST/EiJ susceptible | ABCDFH | induction of apoptosis | 0.001376 |  |  |
| CAST/EiJ susceptible | ABCDFH | myeloid cell activation involved in immune response | 0.001379 |  |  |
| CAST/EiJ susceptible | ABCDFH | induction of programmed cell death | 0.001414 |  |  |
| CAST/EiJ susceptible | ABCDFH | lymphocyte differentiation | 0.001682 |  |  |
| CAST/EiJ susceptible | ABCDFH | locomotion | 0.001748 |  |  |
| CAST/EiJ susceptible | ABCDFH | regulation of cellular process | 0.001814 |  |  |
| CAST/EiJ susceptible | ABCDFH | positive regulation of cell migration | 0.001976 |  |  |
| CAST/EiJ susceptible | ABCDFH | macromolecule metabolic process | 0.001976 |  |  |
| CAST/EiJ susceptible | ABCDFH | T cell activation | 0.002321 |  |  |
| CAST/EiJ susceptible | ABCDFH | gene expression | 0.002405 |  |  |
| CAST/EiJ susceptible | ABCDFH | positive regulation of immune system process | 0.002411 |  |  |
| CAST/EiJ susceptible | ABCDFH | positive regulation of cell motility | 0.002424 |  |  |
| CAST/EiJ susceptible | ABCDFH | cellular macromolecule metabolic process | 0.002769 |  |  |
| CAST/EiJ susceptible | ABCDFH | platelet degranulation | 0.002922 |  |  |
| CAST/EiJ susceptible | ABCDFH | negative regulation of biological process | 0.003182 |  |  |
| CAST/EiJ susceptible | ABCDFH | response to lipopolysaccharide | 0.003481 |  |  |
| CAST/EiJ susceptible | ABCDFH | positive regulation of cellular component movement | 0.003591 |  |  |
| CAST/EiJ susceptible | ABCDFH | regulation of defense response | 0.003668 |  |  |
| CAST/EiJ susceptible | ABCDFH | positive regulation of response to stimulus | 0.003917 |  |  |
| CAST/EiJ susceptible | ABCDFH | regulation of multicellular organismal process | 0.004081 |  |  |
| CAST/EiJ susceptible | ABCDFH | positive regulation of locomotion | 0.004134 |  |  |
| CAST/EiJ susceptible | ABCDFH | regulation of cell proliferation | 0.004197 |  |  |
| CAST/EiJ susceptible | ABCDFH | myeloid leukocyte mediated immunity | 0.00461 |  |  |
| CAST/EiJ susceptible | ABCDFH | regulation of leukocyte degranulation | 0.004793 |  |  |
| CAST/EiJ susceptible | ABCDFH | regulation of signal transduction | 0.004793 |  |  |
| CAST/EiJ susceptible | ABCDFH | blood vessel morphogenesis | 0.005524 |  |  |
| CAST/EiJ susceptible | ABCDFH | regulation of immune response | 0.006768 |  |  |
| CAST/EiJ susceptible | ABCDFH | positive regulation of cytokine production | 0.006842 |  |  |
| CAST/EiJ susceptible | ABCDFH | cell chemotaxis | 0.006948 |  |  |
| CAST/EiJ susceptible | ABCDFH | positive regulation of cell activation | 0.007082 |  |  |
| CAST/EiJ susceptible | ABCDFH | leukocyte degranulation | 0.007082 |  |  |
| CAST/EiJ susceptible | ABCDFH | regulation of phagocytosis | 0.0071 |  |  |
| CAST/EiJ susceptible | ABCDFH | response to virus | 0.007938 |  |  |
| CAST/EiJ susceptible | ABCDFH | blood vessel development | 0.007947 |  |  |
| CAST/EiJ susceptible | ABCDFH | vasculature development | 0.00809 |  |  |
| CAST/EiJ susceptible | ABCDFH | negative regulation of cellular process | 0.009219 |  |  |
| CAST/EiJ susceptible | ABCDFH | endocytosis | 0.010185 |  |  |
| CAST/EiJ susceptible | ABCDFH | membrane invagination | 0.010185 |  |  |
| CAST/EiJ susceptible | ABCDFH | cellular response to stimulus | 0.010219 |  |  |
| CAST/EiJ susceptible | ABCDFH | positive regulation of cell proliferation | 0.010287 |  |  |
| CAST/EiJ susceptible | ABCDFH | positive regulation of apoptotic process | 0.010449 |  |  |
| CAST/EiJ susceptible | ABCDFH | regulation of developmental process | 0.010938 |  |  |
| CAST/EiJ susceptible | ABCDFH | myeloid cell differentiation | 0.011414 |  |  |
| CAST/EiJ susceptible | ABCDFH | cellular response to organic substance | 0.011414 |  |  |
| CAST/EiJ susceptible | ABCDFH | regulation of myeloid leukocyte mediated immunity | 0.011414 |  |  |
| CAST/EiJ susceptible | ABCDFH | cellular response to interferon-gamma | 0.011414 |  |  |
| CAST/EiJ susceptible | ABCDFH | regulation of lymphocyte activation | 0.011462 |  |  |
| CAST/EiJ susceptible | ABCDFH | regulation of blood coagulation | 0.011462 |  |  |
| CAST/EiJ susceptible | ABCDFH | regulation of hemostasis | 0.011462 |  |  |
| CAST/EiJ susceptible | ABCDFH | positive regulation of programmed cell death | 0.011535 |  |  |
| CAST/EiJ susceptible | ABCDFH | response to stimulus | 0.012311 |  |  |
| CAST/EiJ susceptible | ABCDFH | regulated secretory pathway | 0.012701 |  |  |
| CAST/EiJ susceptible | ABCDFH | leukocyte chemotaxis | 0.013063 |  |  |
| CAST/EiJ susceptible | ABCDFH | cellular developmental process | 0.013635 |  |  |
| CAST/EiJ susceptible | ABCDFH | RNA modification | 0.014533 |  |  |
| CAST/EiJ susceptible | ABCDFH | response to tumor necrosis factor | 0.014533 |  |  |
| CAST/EiJ susceptible | ABCDFH | regulation of localization | 0.015194 |  |  |
| CAST/EiJ susceptible | ABCDFH | integrin-mediated signaling pathway | 0.01544 |  |  |
| CAST/EiJ susceptible | ABCDFH | regulation of mast cell degranulation | 0.015848 |  |  |
| CAST/EiJ susceptible | ABCDFH | cellular response to biotic stimulus | 0.015848 |  |  |
| CAST/EiJ susceptible | ABCDFH | regulation of cell adhesion mediated by integrin | 0.016012 |  |  |
| CAST/EiJ susceptible | ABCDFH | regulation of metabolic process | 0.01604 |  |  |
| CAST/EiJ susceptible | ABCDFH | B cell activation | 0.01604 |  |  |
| CAST/EiJ susceptible | ABCDFH | positive regulation of cell death | 0.017416 |  |  |
| CAST/EiJ susceptible | ABCDFH | establishment of protein localization | 0.018437 |  |  |
| CAST/EiJ susceptible | ABCDFH | regulation of mast cell activation involved in immune response | 0.020452 |  |  |
| CAST/EiJ susceptible | ABCDFH | regulation of cellular metabolic process | 0.024165 |  |  |
| CAST/EiJ susceptible | ABCDFH | positive regulation of immune effector process | 0.024165 |  |  |
| CAST/EiJ susceptible | ABCDFH | leukocyte mediated immunity | 0.025806 |  |  |
| CAST/EiJ susceptible | ABCDFH | regulation of coagulation | 0.025806 |  |  |
| CAST/EiJ susceptible | ABCDFH | antigen processing/presentation of peptide/polysaccharide  via MHC class II | 0.025992 |  |  |
| CAST/EiJ susceptible | ABCDFH | cellular metabolic process | 0.025992 |  |  |
| CAST/EiJ susceptible | ABCDFH | regulation of signaling | 0.025992 |  |  |
| CAST/EiJ susceptible | ABCDFH | vesicle-mediated transport | 0.027253 |  |  |
| CAST/EiJ susceptible | ABCDFH | cation homeostasis | 0.02874 |  |  |
| CAST/EiJ susceptible | ABCDFH | cytokine-mediated signaling pathway | 0.030539 |  |  |
| CAST/EiJ susceptible | ABCDFH | copper ion homeostasis | 0.031609 |  |  |
| CAST/EiJ susceptible | ABCDFH | RNA metabolic process | 0.032127 |  |  |
| CAST/EiJ susceptible | ABCDFH | regulation of platelet activation | 0.032152 |  |  |
| CAST/EiJ susceptible | ABCDFH | regulation of cell communication | 0.033486 |  |  |
| CAST/EiJ susceptible | ABCDFH | regulation of primary metabolic process | 0.034796 |  |  |
| CAST/EiJ susceptible | ABCDFH | cellular membrane organization | 0.035918 |  |  |
| CAST/EiJ susceptible | ABCDFH | cell differentiation | 0.037582 |  |  |
| CAST/EiJ susceptible | ABCDFH | membrane organization | 0.037727 |  |  |
| CAST/EiJ susceptible | ABCDFH | neutrophil chemotaxis | 0.04154 |  |  |
| CAST/EiJ susceptible | ABCDFH | ribosome biogenesis | 0.04154 |  |  |
| CAST/EiJ susceptible | ABCDFH | cell adhesion mediated by integrin | 0.043212 |  |  |
| CAST/EiJ susceptible | ABCDFH | protein transport | 0.04476 |  |  |
| CAST/EiJ susceptible | ABCDFH | macromolecule localization | 0.044828 |  |  |
| CAST/EiJ susceptible | ABCDFH | developmental process | 0.048995 |  |  |
| CAST/EiJ susceptible | ABCDFH | angiogenesis | 0.049283 |  |  |
| CAST/EiJ susceptible | ABCDFH | primary metabolic process | 0.049283 |  |  |
| CAST/EiJ susceptible | ABCDFH | blood coagulation | 0.049798 |  |  |

Allele groupings were determined by grouping alleles based on the largest differences between groups. A=A/J, B=C57BL/6J, C=129S1/SvImJ, D=NOD/ShiLtJ, E=NZO/HILtJ, F=CAST/EiJ, G=PWK/PhJ, H=WSB/EiJ

X
