# Supplementary material for: Modeling Host Genetic Regulation of Influenza Pathogenesis in the Collaborative Cross
Source: PLoS Pathog. 2013 Feb 28;9(2):e1003196. doi: 10.1371/journal.ppat.1003196 (PMC3585141; doi:10.1371/journal.ppat.1003196)
Supplement: Table S9 — Phenotypic mean (ranges) between entire pre-CC population and the Mx1 -/- subpopulation. (DOCX) [file ppat.1003196.s015.docx]

| **Table S9. Phenotypic Mean (Ranges) between entire pre-CC population, and the *Mx1*-/- subpopulation** | | | | | | | | | | | | | | | | |  | |
| --- | --- | --- | --- | --- | --- | --- | --- | --- | --- | --- | --- | --- | --- | --- | --- | --- | --- | --- |
|  | Clinical Disease | | | | Viral replication | | Inflammatory cell infiltrates | | | | | | | Pathology | | | | |
|  | D4 weight | D4 clinical | Hemorrhage | Gross  Edema | Log titer | IHC  score | Airway  inflam | Airway neut | Airway mono | Vascular inflam | Vascular neut | Vascular mono | Alveolar  Inflam | Airway damage | Alveolar damage | Pulmonary edema | | Fibrin |
| Entire  pop. | 89.9  (75.0-109.1) | 1.4  (0-3.5) | 0.26 (0-2) | 0.61 (0-3) | 4.18 (BDL^#^-6.34) | 2.94 (0-5) | 1.48  (0-2.9) | 0.95  (0-3) | 1.38  (0.5-2.5) | 1.58  (0-3) | 0.62  (0-3) | 1.31  (0-2.5) | 0.83  (0-3) | 1.41  (0-3) | 0.5  (0-2) | 1.08  (0-3) | | 0.87  (0-3) |
| *Mx1*-/- pop. | 86.3  (75.2-101.3) | 1.73  (0-3.5) | 0.3  (0-2) | 0.66  (0-2) | 4.74 (BDL-6.34) | 3.46  (0-5) | 1.67  (0-2.9) | 1.18  (0-3) | 1.44 (0.5-2.5) | 1.69  (0-3) | 0.75  (0-3) | 1.37  (0-2.5) | 1.05  (0-2.5) | 1.8  (0-3) | 0.42  (0-2) | 1.2  (0-3) | | 0.9  (0-3) |
| ^#^BDL=Below Detectable limit of Log_10_=1.87. | | | | | | | | | | | | | | | | | |  |
