# Supplementary material for: Modeling Host Genetic Regulation of Influenza Pathogenesis in the Collaborative Cross
Source: PLoS Pathog. 2013 Feb 28;9(2):e1003196. doi: 10.1371/journal.ppat.1003196 (PMC3585141; doi:10.1371/journal.ppat.1003196)
Supplement: Table S10 — Phenotypic correlations across the Mx1 -/- pre-CC subpopulation. (DOCX) [file ppat.1003196.s016.docx]

| **Table S10. Phenotypic correlations across the *Mx1*-/- pre-CC subpopluation** | | | | | | | | | | | | | | | | |
| --- | --- | --- | --- | --- | --- | --- | --- | --- | --- | --- | --- | --- | --- | --- | --- | --- |
|  | Clinical Disease | | | | Viral replication | | Inflammatory cell infiltrates | | | | | | | Pathology | | |
|  | D4 weight | D4 clinical | Hem | Gross Edema | Log titer | IHC score | Airway inflam | Airway neut | Airway mono | Vasc inflam | Vasc neut | Vasc mono | Alve inflam | Airway damage | Alve damage | Pulmonary edema |
| Fibrin | -0.03 | -0.03 | 0.05 | -0.03 | 0.15 | 0.17 | 0.08 | 0.14 | 0.13 | 0.1 | 0.01 | 0.15 | 0.08 | 0.01 | 0.07 | 0.19 |
| Pulm Edema | 0.02 | -0.06 | 0.04 | -0.05 | 0 | 0 | 0.06 | 0.23 | 0.18 | 0.11 | 0.16 | 0.19 | 0.12 | 0.11 | 0.07 |  |
| Alve Damage | -0.02 | 0.01 | -0.17 | 0.04 | -0.14 | 0.06 | 0.17 | 0.11 | 0.05 | 0.21 | 0.22 | 0.02 | 0.4 | 0.08 |  |  |
| Airway Damage | -0.28 | 0.13 | -0.11 | 0.11 | 0.16 | 0.21 | 0.45 | 0.31 | 0.47 | 0.31 | 0.26 | 0.42 | 0.08 |  |  |  |
| Alve Inflam | -0.06 | 0.03 | 0.19 | -0.06 | 0 | 0.15 | 0.22 | 0.27 | 0.29 | 0.21 | 0.2 | 0.24 |  |  |  |  |
| Vasc Mono | -0.12 | 0.09 | -0.04 | -0.02 | 0.23 | 0.19 | 0.4 | 0.38 | 0.9 | 0.22 | 0.35 |  |  |  |  |  |
| Vasc Neut | -0.1 | 0.07 | -0.12 | -0.03 | 0 | 0.04 | 0.33 | 0.68 | 0.39 | 0.22 |  |  |  |  |  |  |
| Vasc Inflam | -0.14 | -0.05 | 0.07 | -0.11 | -0.05 | 0.14 | 0.39 | 0.22 | 0.21 |  |  |  |  |  |  |  |
| Airway Mono | -0.14 | 0.04 | -0.06 | 0.07 | 0.21 | 0.2 | 0.47 | 0.39 |  |  |  |  |  |  |  |  |
| Airway Neut | -0.16 | 0.05 | -0.15 | -0.01 | 0.04 | 0.09 | 0.39 |  |  |  |  |  |  |  |  |  |
| Airway inflam | -0.2 | 0.12 | 0 | 0.1 | 0.12 | 0.27 |  |  |  |  |  |  |  |  |  |  |
| IHC score | -0.39 | 0.36 | 0.06 | 0.14 | 0.36 |  |  |  |  |  |  |  |  |  |  |  |
| Log titer | -0.26 | 0.35 | 0.07 | 0.03 |  |  |  |  |  |  |  |  |  |  |  |  |
| Gross Edema | -0.09 | 0.05 | -0.04 |  |  |  |  |  |  |  |  |  |  |  |  |  |
| Hem | 0 | 0.09 |  |  |  |  |  |  |  |  |  |  |  |  |  |  |
| D4 clinical | -0.44 |  |  |  |  |  |  |  |  |  |  |  |  |  |  |  |
| Correlations are Spearman’s rho. Blue shading indicate significance, lt. blue p<0.05, med blue p<0.001, dk blue p<0.00001 Abbreviations: inflam=inflammatory cell infiltrates, neut = neutrophils, mono = monocytes, pulm=pulmonary, vasc=vascular, alve=alveolar | | | | | | | | | | | | | | | | |
