# Supplementary material for: Modeling Host Genetic Regulation of Influenza Pathogenesis in the Collaborative Cross
Source: PLoS Pathog. 2013 Feb 28;9(2):e1003196. doi: 10.1371/journal.ppat.1003196 (PMC3585141; doi:10.1371/journal.ppat.1003196)
Supplement: Table S11 — Transcripts within modules in the Mx1 -/- subpopulation. (DOCX) [file ppat.1003196.s017.docx]

| **Table S11. Transcripts within modules in the *Mx1*-/- subpopulation** | |
| --- | --- |
| **Module** | **Transcript** |
| M | 1110007M04Rik |
| M | 1110007M04Rik |
| M | 1110038B12Rik |
| M | 1500012F01Rik |
| M | 1500034J01Rik |
| M | 1700012H17Rik |
| M | 1700017B05Rik |
| M | 1810009K13Rik |
| M | 2010002N04Rik |
| M | 2010003J03Rik |
| M | 2010109K11Rik |
| M | 2010204K13Rik |
| M | 2010305A19Rik |
| M | 2010310D06Rik |
| M | 2310008H09Rik |
| M | 2310011J03Rik |
| M | 2410012H22Rik |
| M | 2610028A01Rik |
| M | 2700007P21Rik |
| M | 2700094F01Rik |
| M | 3110001I22Rik |
| M | 3200002M19Rik |
| M | 3200002M19Rik |
| M | 4833422F24Rik |
| M | 4833446K15Rik |
| M | 4833446K15Rik |
| M | 4933403F05Rik |
| M | 5133401H06Rik |
| M | 5530401N12Rik |
| M | 5730528L13Rik |
| M | 5830416P10Rik |
| M | 9430008C03Rik |
| M | 9430038I01Rik |
| M | A030007L17Rik |
| M | AA960436 |
| M | Aars |
| M | Aars |
| M | Acvr1b |
| M | Adm |
| M | AK012844 |
| M | AK038388 |
| M | AK041801 |
| M | AK043151 |
| M | AK048793 |
| M | AK160276 |
| M | AK169742 |
| M | Ak2 |
| M | Alkbh3 |
| M | Ap4b1 |
| M | Apex1 |
| M | Arf5 |
| M | Arid5a |
| M | Ascl3 |
| M | Asprv1 |
| M | Atf1 |
| M | Atf3 |
| M | Axud1 |
| M | B430306N03Rik |
| M | BC051230 |
| M | Bcar1 |
| M | Bcl2l2 |
| M | Bcl3 |
| M | Bean |
| M | Birc3 |
| M | Blvra |
| M | C1qbp |
| M | C330023M02Rik |
| M | Camk2n2 |
| M | Cant1 |
| M | Car13 |
| M | Cars |
| M | Cars |
| M | Cbwd1 |
| M | Ccdc86 |
| M | Ccl2 |
| M | Ccl20 |
| M | Cct6b |
| M | Cd14 |
| M | Cd3eap |
| M | Cd44 |
| M | Cdc42ep4 |
| M | Ch25h |
| M | Chac1 |
| M | Cish |
| M | Cldn4 |
| M | Clec5a |
| M | Cnnm4 |
| M | Col4a2 |
| M | Cox10 |
| M | Csf1 |
| M | Csf3r |
| M | Ctdp1 |
| M | Cul2 |
| M | Cyp4f18 |
| M | Cyr61 |
| M | D10Ertd438e |
| M | D11Ertd497e |
| M | D13Wsu177e |
| M | Dbn1 |
| M | Ddx28 |
| M | Dhx38 |
| M | Dlgap4 |
| M | Dnajb9 |
| M | Dscr1 |
| M | Dus4l |
| M | Dusp4 |
| M | E2f3 |
| M | Eaf1 |
| M | Eef1g |
| M | EG382448 |
| M | EG435337 |
| M | Egr3 |
| M | Eif4a1 |
| M | Emilin2 |
| M | Emilin2 |
| M | Enc1 |
| M | ENSMUST00000042143 |
| M | ENSMUST00000070019 |
| M | Ercc1 |
| M | Exosc2 |
| M | Fbxl5 |
| M | Fbxo42 |
| M | Fkbp5 |
| M | Fos |
| M | Fosl1 |
| M | Fpgs |
| M | Fpr-rs2 |
| M | Fst |
| M | Ftsj3 |
| M | Gadd45b |
| M | Gadd45g |
| M | Gars |
| M | Gcnt2 |
| M | Glce |
| M | Glipr2 |
| M | Gm22 |
| M | Gmfb |
| M | Gosr2 |
| M | Gtf2f2 |
| M | Has1 |
| M | Hbegf |
| M | Hif1a |
| M | Hmgcr |
| M | Hnrpa1 |
| M | Hpdl |
| M | Ier2 |
| M | Ier5 |
| M | Ier5l |
| M | Il1a |
| M | Il1r2 |
| M | Il1r2 |
| M | Il4ra |
| M | Il6 |
| M | Inhba |
| M | Inhbb |
| M | Ipo4 |
| M | Itga3 |
| M | Itgav |
| M | Itpkc |
| M | Jmjd3 |
| M | Josd3 |
| M | Josd3 |
| M | Josd3 |
| M | Junb |
| M | Kcne4 |
| M | Kpna3 |
| M | Litaf |
| M | Lmna |
| M | Lmna |
| M | Lmna |
| M | LOC219106 |
| M | Loh12cr1 |
| M | Lrig2 |
| M | Ltb4r1 |
| M | Mad2l2 |
| M | Maff |
| M | Map2k3 |
| M | Map3k15 |
| M | Mapk6 |
| M | Mars |
| M | Matk |
| M | Mcl1 |
| M | Mizf |
| M | Mki67ip |
| M | Morc2a |
| M | Mphosph10 |
| M | Mrto4 |
| M | Mybbp1a |
| M | Myc |
| M | Myc |
| M | Myc |
| M | NAP107236-1 |
| M | NAP113293-1 |
| M | NAP122876-1 |
| M | Ncl |
| M | Ndel1 |
| M | Nek6 |
| M | Nfam1 |
| M | Nfil3 |
| M | Nfil3 |
| M | Nfkb2 |
| M | Nip7 |
| M | Nipa2 |
| M | Nkiras2 |
| M | Nle1 |
| M | Noc3l |
| M | Nol12 |
| M | Nol5 |
| M | Nola2 |
| M | Npl |
| M | Nrg1 |
| M | Nrg1 |
| M | Nrip3 |
| M | Nubp1 |
| M | Nufip1 |
| M | Nup50 |
| M | Nup54 |
| M | Nvl |
| M | Oraov1 |
| M | Ovol1 |
| M | Oxsm |
| M | Pappa |
| M | Pdlim7 |
| M | Pdlim7 |
| M | Phca |
| M | Pkm2 |
| M | Pla1a |
| M | Plat |
| M | Plat |
| M | Plaur |
| M | Plaur |
| M | Plk3 |
| M | Pno1 |
| M | Ppa1 |
| M | Ppp1r2 |
| M | Ppp1r2 |
| M | Ppp3cc |
| M | Pram1 |
| M | Prtn3 |
| M | Psmd8 |
| M | Ptgir |
| M | Ptpn1 |
| M | Ptpn2 |
| M | Ptpn22 |
| M | Pus1 |
| M | Pus7 |
| M | Pvr |
| M | Pvr |
| M | Pvr |
| M | Pxn |
| M | Rab5c |
| M | Rabl3 |
| M | Ranbp5 |
| M | Rars |
| M | Rassf1 |
| M | Rbm13 |
| M | Rbm9 |
| M | Relb |
| M | Rg9mtd2 |
| M | Rhoc |
| M | Ripk3 |
| M | Rnd3 |
| M | Rnd3 |
| M | Rpl7l1 |
| M | Rpp38 |
| M | Rras2 |
| M | Rras2 |
| M | Rrbp1 |
| M | Rrp9 |
| M | Samd8 |
| M | Sbno2 |
| M | Sdad1 |
| M | Sdcbp2 |
| M | Selp |
| M | Sema4c |
| M | Sema7a |
| M | Serpine1 |
| M | Sertad1 |
| M | Sfrs2 |
| M | Sgms2 |
| M | Sh2d5 |
| M | Slc16a6 |
| M | Slc25a20 |
| M | Slc25a25 |
| M | Slc25a25 |
| M | Slc25a30 |
| M | Slc25a37 |
| M | Slc2a1 |
| M | Slc30a1 |
| M | Slc30a7 |
| M | Slc35d2 |
| M | Slc39a14 |
| M | Slc3a2 |
| M | Slc7a6 |
| M | Smn1 |
| M | Snag1 |
| M | Snx8 |
| M | Socs3 |
| M | Spata2 |
| M | Spata20 |
| M | Spata20 |
| M | Sphk1 |
| M | Sphk1 |
| M | Spry2 |
| M | Spry4 |
| M | Spsb1 |
| M | Srfbp1 |
| M | Srgn |
| M | Srm |
| M | Srxn1 |
| M | St6galnac4 |
| M | St7 |
| M | Steap2 |
| M | Stk19 |
| M | Stk19 |
| M | Stk19 |
| M | Stk40 |
| M | Syncrip |
| M | Taf9 |
| M | Tbl3 |
| M | Tbrg4 |
| M | TC1628823 |
| M | TC1681528 |
| M | Tcfec |
| M | Thbs1 |
| M | Thumpd3 |
| M | Timm10 |
| M | Tle3 |
| M | Tlk2 |
| M | Tmem127 |
| M | Tmem185b |
| M | Tmem38b |
| M | Tmem49 |
| M | Tnc |
| M | Tnfaip3 |
| M | Tnfrsf12a |
| M | Tnpo2 |
| M | Tomm20 |
| M | Tomm70a |
| M | Tpcn2 |
| M | Trem3 |
| M | Trib1 |
| M | Tsc22d2 |
| M | Tsc22d2 |
| M | Tubb6 |
| M | U11274 |
| M | Ubap1 |
| M | Ubap2l |
| M | Ubqln1 |
| M | Ubtd1 |
| M | Uck2 |
| M | Umps |
| M | Utp18 |
| M | Vasp |
| M | Vasp |
| M | Vcan |
| M | W15861 |
| M | Wdr12 |
| M | Wdr4 |
| M | Wsb1 |
| M | Yars |
| M | Ykt6 |
| M | Yrdc |
| M | Yrdc |
| M | Zc3h12a |
| M | Zc3h12c |
| M | Zfand5 |
| M | Zfp143 |
| M | Zfp296 |
| M | Zfp36 |
| M | Zfp36l2 |
| M | Zfp384 |
| M | Zwint |
| N | 1-Mar |
| N | 5-Mar |
| N | 6-Mar |
| N | 0610010F05Rik |
| N | 0910001A06Rik |
| N | 0910001A06Rik |
| N | 1100001G20Rik |
| N | 1110018G07Rik |
| N | 1110032O16Rik |
| N | 1200011I18Rik |
| N | 1200015F23Rik |
| N | 1600014C10Rik |
| N | 1600027N09Rik |
| N | 1700001K19Rik |
| N | 1700027J05Rik |
| N | 1700037C18Rik |
| N | 1700041G16Rik |
| N | 1700049L16Rik |
| N | 1700056N10Rik |
| N | 1810054D07Rik |
| N | 2010012C16Rik |
| N | 2010316F05Rik |
| N | 2210008N01Rik |
| N | 2210403K04Rik |
| N | 2310014H01Rik |
| N | 2310022A10Rik |
| N | 2410001C21Rik |
| N | 2410127E18Rik |
| N | 2700007P21Rik |
| N | 2700019D07Rik |
| N | 2810403A07Rik |
| N | 2810439F02Rik |
| N | 2810457I06Rik |
| N | 2810474O19Rik |
| N | 3110003A17Rik |
| N | 3110003A17Rik |
| N | 3110005G23Rik |
| N | 4632434I11Rik |
| N | 4732429D16Rik |
| N | 4921520G13Rik |
| N | 4930471M23Rik |
| N | 4930503L19Rik |
| N | 4933440N22Rik |
| N | 5031414D18Rik |
| N | 5730442P18Rik |
| N | 5730458M16Rik |
| N | 5730508B09Rik |
| N | 5730559C18Rik |
| N | 5830400J07Rik |
| N | 5830443L24Rik |
| N | 6330578E17Rik |
| N | 6430511F03 |
| N | 8030431J09Rik |
| N | 9030611O19Rik |
| N | 9030611O19Rik |
| N | 9130017C17Rik |
| N | 9130017N09Rik |
| N | 9330175E14Rik |
| N | 9430034N14Rik |
| N | 9430098F02Rik |
| N | 9930023K05Rik |
| N | 9930023K05Rik |
| N | A_52_P981179 |
| N | A230097K15Rik |
| N | A430084P05Rik |
| N | A530032D15Rik |
| N | A530098C11Rik |
| N | A630077B13Rik |
| N | A730011C13Rik |
| N | AA407452 |
| N | Abhd2 |
| N | Abhd2 |
| N | Abhd8 |
| N | Abtb2 |
| N | Acp2 |
| N | Acp2 |
| N | Adam21 |
| N | Adar |
| N | Adora3 |
| N | Adprh |
| N | Adrbk1 |
| N | Adrbk1 |
| N | Aftph |
| N | Ahrr |
| N | AI447904 |
| N | AI447904 |
| N | AI451617 |
| N | AI481105 |
| N | Aif1 |
| N | AK008862 |
| N | AK013113 |
| N | AK013903 |
| N | AK030647 |
| N | AK032625 |
| N | AK034974 |
| N | AK035243 |
| N | AK037919 |
| N | AK039806 |
| N | AK040628 |
| N | AK041551 |
| N | AK042092 |
| N | AK046811 |
| N | AK050867 |
| N | AK051012 |
| N | AK053561 |
| N | AK054187 |
| N | AK054376 |
| N | AK076900 |
| N | AK079230 |
| N | AK079475 |
| N | AK079518 |
| N | AK079803 |
| N | AK081327 |
| N | AK081338 |
| N | AK083709 |
| N | AK084024 |
| N | AK084420 |
| N | AK087356 |
| N | AK087429 |
| N | AK089714 |
| N | AK089832 |
| N | AK090164 |
| N | AK134580 |
| N | AK149247 |
| N | Aloxe3 |
| N | Als2 |
| N | Amica1 |
| N | Ankrd57 |
| N | Aoah |
| N | Aoc2 |
| N | Ap1s2 |
| N | Ap3m2 |
| N | Apob48r |
| N | Apobec1 |
| N | Apof |
| N | Apool |
| N | Areg |
| N | Arf6 |
| N | Arf6 |
| N | Arhgap30 |
| N | Arhgap30 |
| N | Arid4a |
| N | Arl5c |
| N | Armc7 |
| N | Arpc4 |
| N | Asb13 |
| N | Asb13 |
| N | Asb13 |
| N | Asb4 |
| N | Atp11c |
| N | Atp1a3 |
| N | Atp6v1b2 |
| N | Atp7a |
| N | Atxn7l1 |
| N | AU042671 |
| N | Auh |
| N | AW108044 |
| N | AW112010 |
| N | AW552889 |
| N | Axl |
| N | AY078069 |
| N | B230217C12Rik |
| N | B230312A22Rik |
| N | Bai1 |
| N | Bak1 |
| N | Basp1 |
| N | BC004022 |
| N | BC004022 |
| N | BC013712 |
| N | BC023892 |
| N | BC027057 |
| N | BC032204 |
| N | BC033915 |
| N | BC033915 |
| N | BC033915 |
| N | BC117090 |
| N | BC117090 |
| N | BC117090 |
| N | Bcl2a1b |
| N | Bcl2l1 |
| N | Bcl2l11 |
| N | Bcl9 |
| N | Birc2 |
| N | Bloc1s2 |
| N | Bri3bp |
| N | Btg1 |
| N | BX529199 |
| N | C130021H21Rik |
| N | C130032J12Rik |
| N | C230075M21Rik |
| N | C230081A13Rik |
| N | C330006P03Rik |
| N | C330023M02Rik |
| N | C330023M02Rik |
| N | C3ar1 |
| N | C3ar1 |
| N | C530007A02Rik |
| N | Cacnb4 |
| N | Calm3 |
| N | Capg |
| N | Casp8 |
| N | Ccdc59 |
| N | Ccdc88 |
| N | Ccdc90a |
| N | Ccl12 |
| N | Ccl4 |
| N | Ccl5 |
| N | Ccnd2 |
| N | Ccr2 |
| N | Ccr7 |
| N | Ccrl2 |
| N | Cd180 |
| N | Cd300lf |
| N | Cd37 |
| N | Cd52 |
| N | Cd68 |
| N | Cd86 |
| N | Cdk5r1 |
| N | Cdt1 |
| N | Cebpb |
| N | Cebpb |
| N | Centa2 |
| N | CF747846 |
| N | Chic2 |
| N | Clcn5 |
| N | Clcn6 |
| N | Clip2 |
| N | Cln3 |
| N | Cln8 |
| N | Cndp2 |
| N | Col23a1 |
| N | Coq5 |
| N | Coro1a |
| N | Coro1a |
| N | Coro7 |
| N | Cotl1 |
| N | Cpsf4 |
| N | Crem |
| N | Crem |
| N | Crem |
| N | Crem |
| N | Crem |
| N | Crispld2 |
| N | Crk |
| N | Cryba4 |
| N | Csf2ra |
| N | Csf2rb2 |
| N | Csk |
| N | Csnk1d |
| N | Ctsz |
| N | Cttnbp2nl |
| N | Cxcl16 |
| N | Cyba |
| N | Cyfip2 |
| N | Cyp7b1 |
| N | Cysltr1 |
| N | Cysltr1 |
| N | D15Wsu75e |
| N | D630023B12Rik |
| N | Darc |
| N | Daxx |
| N | Dcakd |
| N | Dclre1c |
| N | Dcp2 |
| N | Dcp2 |
| N | Dennd1a |
| N | Dennd1a |
| N | Dennd1c |
| N | Dgkz |
| N | Dhfr |
| N | Dhrs9 |
| N | Disc1 |
| N | Dnmt3a |
| N | Dnmt3l |
| N | Dock2 |
| N | Dok1 |
| N | Dok3 |
| N | Dph2 |
| N | Dusp10 |
| N | Dusp2 |
| N | E030010A14Rik |
| N | E430028B21Rik |
| N | Ebi3 |
| N | Ecm1 |
| N | Edem1 |
| N | Efcbp2 |
| N | Efhd2 |
| N | EG240327 |
| N | EG432555 |
| N | EG433016 |
| N | EG545306 |
| N | EG634650 |
| N | EG668139 |
| N | Ehbp1l1 |
| N | Eif2ak1 |
| N | Eif2ak2 |
| N | Eif2s2 |
| N | Elf4 |
| N | Emr1 |
| N | Endogl1 |
| N | ENSMUSG00000071525 |
| N | ENSMUST00000038890 |
| N | ENSMUST00000057926 |
| N | ENSMUST00000073088 |
| N | ENSMUST00000077110 |
| N | ENSMUST00000101511 |
| N | Entpd7 |
| N | Epha2 |
| N | Ergic1 |
| N | Esrra |
| N | Etv6 |
| N | Evi2a |
| N | Evl |
| N | F10 |
| N | F7 |
| N | Fbxl14 |
| N | Fbxo6 |
| N | Fbxw11 |
| N | Fbxw17 |
| N | Fcer1g |
| N | Fcgr2b |
| N | Fcgr4 |
| N | Fem1c |
| N | Fes |
| N | Fgl2 |
| N | Fgr |
| N | Fmn1 |
| N | Fmnl1 |
| N | Fmnl1 |
| N | Fndc3a |
| N | Fosb |
| N | Foxk1 |
| N | Frmd4b |
| N | Fus |
| N | Gab2 |
| N | Gadd45b |
| N | Galns |
| N | Galnt6 |
| N | Galnt7 |
| N | Garnl1 |
| N | Gas2l3 |
| N | Gas7 |
| N | Gba |
| N | Gbp4 |
| N | Gca |
| N | Gch1 |
| N | Gch1 |
| N | Ggps1 |
| N | Gimap5 |
| N | Gimap9 |
| N | Gla |
| N | Glod5 |
| N | Gm1966 |
| N | Gmip |
| N | Gmppb |
| N | Gna13 |
| N | Gng12 |
| N | Gng2 |
| N | Gnrh1 |
| N | Golph3 |
| N | Golt1b |
| N | Gpr160 |
| N | Gpr64 |
| N | Gpr65 |
| N | Grap2 |
| N | Grina |
| N | Grpel2 |
| N | Gtpbp2 |
| N | Gtpbp2 |
| N | Gzmd |
| N | H2afy |
| N | H2-M3 |
| N | H3f3b |
| N | Havcr2 |
| N | Hbp1 |
| N | Hck |
| N | Hcls1 |
| N | Hcst |
| N | Hdh |
| N | Herc5 |
| N | Herc5 |
| N | Hhex |
| N | Hhex |
| N | Hipk1 |
| N | Hipk2 |
| N | Hpse |
| N | Hsf5 |
| N | Hsh2d |
| N | Htra4 |
| N | Ibrdc1 |
| N | Ibrdc3 |
| N | Icam5 |
| N | Id2 |
| N | Ifi203 |
| N | Ifi204 |
| N | Ifi204 |
| N | Ifi30 |
| N | Ifi35 |
| N | Ifi44 |
| N | Ifit2 |
| N | Ifitm6 |
| N | Ifna12 |
| N | Ifnb1 |
| N | Ifng |
| N | Ifngr2 |
| N | Ifrg15 |
| N | Igsf6 |
| N | Ihpk1 |
| N | Ihpk1 |
| N | Il12rb1 |
| N | Il13ra1 |
| N | Il15 |
| N | Il15ra |
| N | Il17ra |
| N | Il17ra |
| N | Il18bp |
| N | Il18bp |
| N | Il18rap |
| N | Il1f9 |
| N | Il1rn |
| N | Il20rb |
| N | Il24 |
| N | Il6ra |
| N | Il6ra |
| N | Il7r |
| N | Inpp5d |
| N | Irf1 |
| N | Irf2 |
| N | Irf5 |
| N | Irgb10 |
| N | Irgm |
| N | Irgm |
| N | Isg20 |
| N | Itga4 |
| N | Itga4 |
| N | Itgax |
| N | Itpa |
| N | Jak2 |
| N | Katna1 |
| N | Kbtbd2 |
| N | Klhl15 |
| N | Kpna3 |
| N | Krt18 |
| N | Krt85 |
| N | Lair1 |
| N | Larp1 |
| N | Lasp1 |
| N | Lcp2 |
| N | Lfng |
| N | Lgals1 |
| N | Lgals3bp |
| N | Lgals8 |
| N | Lipg |
| N | Lnpep |
| N | LOC545342 |
| N | Lonrf3 |
| N | Lrch1 |
| N | Lrch4 |
| N | Lrp1 |
| N | Lrp12 |
| N | Lrrc4 |
| N | Lrrc61 |
| N | Lsm12 |
| N | Lyl1 |
| N | Lyn |
| N | Mafb |
| N | Mafk |
| N | Malt1 |
| N | Malt1 |
| N | Map4k4 |
| N | Mapkapk3 |
| N | Marcksl1 |
| N | Marcksl1 |
| N | Mbc2 |
| N | Mbd1 |
| N | Mbd1 |
| N | Mdk |
| N | Mdm4 |
| N | Mef2d |
| N | Mefv |
| N | Mgat1 |
| N | Mgat4a |
| N | Midn |
| N | Mlkl |
| N | Mmp19 |
| N | Mms19l |
| N | Mobkl2a |
| N | Morc3 |
| N | Mrgprg |
| N | Mrpl27 |
| N | Ms4a4b |
| N | Ms4a4d |
| N | Ms4a6c |
| N | Msh3 |
| N | Mt1 |
| N | Mx1 |
| N | Mx1 |
| N | Mxd1 |
| N | Mylip |
| N | Myo1f |
| N | NAP007796-001 |
| N | NAP019557-001 |
| N | NAP037326-1 |
| N | NAP046244-1 |
| N | NAP050738-1 |
| N | NAP055718-1 |
| N | NAP055900-1 |
| N | NAP058998-1 |
| N | NAP061805-1 |
| N | NAP066348-1 |
| N | NAP097934-001 |
| N | NAP102462-1 |
| N | Nat5 |
| N | Ncf1 |
| N | Ncf4 |
| N | Ncoa7 |
| N | Neu1 |
| N | Neud4 |
| N | Nfam1 |
| N | Nfe2 |
| N | Nfs1 |
| N | Nktr |
| N | Noc4l |
| N | Nt5c3 |
| N | Nudcd1 |
| N | Nupr1 |
| N | Oaf |
| N | Oas1a |
| N | Oas2 |
| N | Oas2 |
| N | Oas3 |
| N | Oasl2 |
| N | Odf2 |
| N | Ogfr |
| N | Ogfrl1 |
| N | Olfr433 |
| N | Olfr920 |
| N | Osgin1 |
| N | Osmr |
| N | Osr1 |
| N | Otof |
| N | OTTMUSG00000016644 |
| N | OTTMUSG00000016644 |
| N | Otub1 |
| N | Oxsr1 |
| N | P2ry14 |
| N | P2ry6 |
| N | Parp14 |
| N | Pbef1 |
| N | Pbef1 |
| N | Pcgf5 |
| N | Pdcd1 |
| N | Pdss1 |
| N | Pdzrn3 |
| N | Pfdn4 |
| N | Pfkp |
| N | Pfn1 |
| N | Phf11 |
| N | Phf15 |
| N | Phf15 |
| N | Pik3ap1 |
| N | Pik3cd |
| N | Pim1 |
| N | Pip5k1a |
| N | Pira3 |
| N | Pkn1 |
| N | Pkn3 |
| N | Pla2g4c |
| N | Plcg2 |
| N | Pld4 |
| N | Plec1 |
| N | Plek |
| N | Plek |
| N | Plekho1 |
| N | Plekhq1 |
| N | Plekhq1 |
| N | Pnp |
| N | Pou3f1 |
| N | Ppif |
| N | Ppil3 |
| N | Ppp1r12c |
| N | Ppp1r15b |
| N | Ppp2r1b |
| N | Ppp3cc |
| N | Prdx5 |
| N | Prkcd |
| N | Pscd4 |
| N | Psd4 |
| N | Psmb10 |
| N | Psmb8 |
| N | Psmd7 |
| N | Pstpip1 |
| N | Ptafr |
| N | Ptp4a1 |
| N | Ptpn14 |
| N | Ptpn6 |
| N | Ptpn6 |
| N | Ptpro |
| N | Pvrl2 |
| N | Pvrl4 |
| N | Pycard |
| N | Qscn6 |
| N | Qscn6 |
| N | Rab20 |
| N | Rab22a |
| N | Rab37 |
| N | Rab43 |
| N | Rab43 |
| N | Rap2b |
| N | Rasgef1b |
| N | Rassf5 |
| N | Rbl1 |
| N | Rbm38 |
| N | Rbm43 |
| N | Rcc2 |
| N | Retnlg |
| N | Rffl |
| N | Rgl1 |
| N | Rhbdf2 |
| N | Rhbdf2 |
| N | Rhcg |
| N | Rhoh |
| N | Rhou |
| N | Riok3 |
| N | Rnase6 |
| N | Rnaseh2b |
| N | Rnf135 |
| N | Rnf149 |
| N | Rnf149 |
| N | Rnf157 |
| N | Rnf213 |
| N | Rnf213 |
| N | Rod1 |
| N | Rpl27a |
| N | Rsad2 |
| N | Rsad2 |
| N | Rufy3 |
| N | Rufy3 |
| N | S100a14 |
| N | S100a9 |
| N | Saa1 |
| N | Saa3 |
| N | Samd1 |
| N | Samd9l |
| N | Samhd1 |
| N | Samhd1 |
| N | Sap30 |
| N | Sat1 |
| N | Scamp2 |
| N | Sco1 |
| N | Sct |
| N | Sdc3 |
| N | Sectm1a |
| N | Sell |
| N | Senp5 |
| N | Sgk3 |
| N | Sgpl1 |
| N | Shb |
| N | Siglec1 |
| N | Sirpa |
| N | Slamf6 |
| N | Slamf7 |
| N | Slc11a1 |
| N | Slc12a9 |
| N | Slc16a10 |
| N | Slc26a2 |
| N | Slc43a2 |
| N | Slc6a13 |
| N | Slfn2 |
| N | Slfn5 |
| N | Smap1l |
| N | Smcr8 |
| N | Smek1 |
| N | Smurf1 |
| N | Snap29 |
| N | Snd1 |
| N | Snf8 |
| N | Snx10 |
| N | Socs1 |
| N | Sp100 |
| N | Spic |
| N | Srcrb4d |
| N | Stat3 |
| N | Stfa2l1 |
| N | Stoml1 |
| N | Stoml1 |
| N | Stx3 |
| N | Stxbp5 |
| N | Sv2a |
| N | Syk |
| N | Taf6l |
| N | Tbc1d1 |
| N | Tbc1d9 |
| N | Tbl1xr1 |
| N | TC1634227 |
| N | TC1645771 |
| N | TC1653266 |
| N | TC1658167 |
| N | Tceb3 |
| N | Tcirg1 |
| N | Thrap2 |
| N | Timeless |
| N | Tiparp |
| N | Tlr3 |
| N | Tlr7 |
| N | Tmem106a |
| N | Tmem40 |
| N | Tmem67 |
| N | Tmem87a |
| N | Tmepai |
| N | Tnf |
| N | Tnfaip8l2 |
| N | Tnfrsf14 |
| N | Tnfrsf1b |
| N | Tnfsf10 |
| N | Tnip1 |
| N | Tor1aip1 |
| N | Trafd1 |
| N | Trafd1 |
| N | Tram1 |
| N | Trex1 |
| N | Trim16 |
| N | Trim21 |
| N | Trim27 |
| N | Trim30 |
| N | Tspan4 |
| N | Ttll11 |
| N | Tuft1 |
| N | Txndc2 |
| N | Txnrd1 |
| N | Tyki |
| N | Tyrobp |
| N | Ubap1 |
| N | Ubd |
| N | Ube2f |
| N | Ube2l6 |
| N | Uck2 |
| N | Ucp2 |
| N | Ugcg |
| N | Ugcg |
| N | Uimc1 |
| N | Unc93b1 |
| N | Usp16 |
| N | Usp25 |
| N | Usp42 |
| N | Vav1 |
| N | Vav1 |
| N | Vcan |
| N | Vcpip1 |
| N | Vcpip1 |
| N | Vps33a |
| N | Vps37b |
| N | Vps54 |
| N | Vta1 |
| N | Wars |
| N | Was |
| N | Was |
| N | Wdr40c |
| N | Wdr62 |
| N | Wdr73 |
| N | Wibg |
| N | Wnt6 |
| N | Wnt7b |
| N | Xdh |
| N | Ypel5 |
| N | Zbtb7b |
| N | Zc3h15 |
| N | Zcchc2 |
| N | Zfp26 |
| N | Zfp26 |
| N | Zfp36 |
| N | Zfp385 |
| N | Zfp709 |
| O | 4-Sep |
| O | 0610011F06Rik |
| O | 0610040J01Rik |
| O | 1110031B06Rik |
| O | 1110031B06Rik |
| O | 1110051M20Rik |
| O | 1110069O07Rik |
| O | 1190002N15Rik |
| O | 1200009O22Rik |
| O | 1700020I14Rik |
| O | 1700040I03Rik |
| O | 1700055M20Rik |
| O | 1810011O10Rik |
| O | 1810019J16Rik |
| O | 2210011C24Rik |
| O | 2210408K08 |
| O | 2310014D11Rik |
| O | 2310022B05Rik |
| O | 2310043N10Rik |
| O | 2310046K01Rik |
| O | 2310057J16Rik |
| O | 2410081M15Rik |
| O | 2610019F03Rik |
| O | 2610301F02Rik |
| O | 2700089E24Rik |
| O | 2810025M15Rik |
| O | 2900009J20Rik |
| O | 2900041A09Rik |
| O | 3110004L20Rik |
| O | 3110004L20Rik |
| O | 3526401B18Rik |
| O | 4631427C17Rik |
| O | 4921533L14Rik |
| O | 4930570C03Rik |
| O | 4933406E20Rik |
| O | 4933413A10Rik |
| O | 4933439F18Rik |
| O | 5330417C22Rik |
| O | 5330417C22Rik |
| O | 5330421C15Rik |
| O | 5730403B10Rik |
| O | 5730446D14Rik |
| O | 5830434P21Rik |
| O | 5830454D03Rik |
| O | 6330505N24Rik |
| O | 8430408O14 |
| O | 8430415E04Rik |
| O | 9030607L17Rik |
| O | 9130221D24Rik |
| O | 9430020K01Rik |
| O | 9430079B08Rik |
| O | 9630058J23Rik |
| O | A_52_P549754 |
| O | A730055C05Rik |
| O | Aass |
| O | Abca2 |
| O | Abca3 |
| O | Abca3 |
| O | Abca3 |
| O | Abca5 |
| O | Abcd3 |
| O | Abcd3 |
| O | Ablim1 |
| O | Ablim1 |
| O | Ablim3 |
| O | Acbd4 |
| O | Acbd5 |
| O | Acot1 |
| O | Acss2 |
| O | Acvr2b |
| O | Acvrl1 |
| O | Adam19 |
| O | Adamtsl5 |
| O | Adcy3 |
| O | Adcy9 |
| O | Add3 |
| O | Adk |
| O | Adrbk2 |
| O | Afap1l1 |
| O | Ager |
| O | Agrn |
| O | Agtr1a |
| O | Agtr1a |
| O | Agtrl1 |
| O | Ahnak |
| O | AI464131 |
| O | AK020422 |
| O | AK028004 |
| O | AK031258 |
| O | AK034355 |
| O | AK043317 |
| O | AK045702 |
| O | AK048091 |
| O | AK051672 |
| O | AK052002 |
| O | AK054507 |
| O | AK085773 |
| O | AK086814 |
| O | AK087708 |
| O | Akap11 |
| O | Akap8l |
| O | Aktip |
| O | Aktip |
| O | Aldh1a7 |
| O | Aldh2 |
| O | Aldh3a2 |
| O | Aldh6a1 |
| O | Ank3 |
| O | Ank3 |
| O | Ankrd12 |
| O | Ankrd25 |
| O | Ankrd25 |
| O | Ankrd40 |
| O | Ankrd44 |
| O | Ankrd47 |
| O | Antxr2 |
| O | Aox1 |
| O | Aox3 |
| O | Aplp2 |
| O | Apoa1bp |
| O | Appl1 |
| O | Aqp1 |
| O | Araf |
| O | Arhgap21 |
| O | Arhgap29 |
| O | Arhgef12 |
| O | Arhgef15 |
| O | Arhgef18 |
| O | Arrb1 |
| O | Arvcf |
| O | Atbf1 |
| O | Atp9a |
| O | Atrn |
| O | Atrnl1 |
| O | AU040829 |
| O | AV154513 |
| O | AW061290 |
| O | AW061290 |
| O | AW215868 |
| O | AW456874 |
| O | Axin2 |
| O | Aytl2 |
| O | B230380D07Rik |
| O | B430201A12Rik |
| O | B930008K04Rik |
| O | B930037P14Rik |
| O | Bahcc1 |
| O | Bambi |
| O | Bbs9 |
| O | BC004853 |
| O | BC021381 |
| O | BC031353 |
| O | BC039093 |
| O | BC043118 |
| O | BC054438 |
| O | BC054438 |
| O | BC059842 |
| O | BE650457 |
| O | Bex4 |
| O | Bmf |
| O | Bmpr1a |
| O | Bnip3l |
| O | Bpgm |
| O | Braf |
| O | Btbd3 |
| O | Btbd3 |
| O | BU530502 |
| O | BU531328 |
| O | BU554808 |
| O | BU557601 |
| O | C030015A19Rik |
| O | C1qtnf2 |
| O | C230082I21Rik |
| O | C230091D08Rik |
| O | C230094B09Rik |
| O | C77370 |
| O | C77370 |
| O | Cacnb2 |
| O | Cadm1 |
| O | Cadm1 |
| O | Calcoco1 |
| O | Calcrl |
| O | Calcrl |
| O | Calm1 |
| O | Calm1 |
| O | Cand1 |
| O | Car4 |
| O | Card10 |
| O | Cat |
| O | Cav1 |
| O | Cav2 |
| O | Cbfa2t3h |
| O | Cbfa2t3h |
| O | Cbr2 |
| O | Cbx7 |
| O | Ccdc92 |
| O | Ccpg1 |
| O | Ccpg1 |
| O | Ccpg1 |
| O | Cd36 |
| O | Cd81 |
| O | Cd9 |
| O | Cd97 |
| O | Cdc25b |
| O | Cdc42bpa |
| O | Cds2 |
| O | Centb2 |
| O | Centd3 |
| O | Cep70 |
| O | Cep70 |
| O | Ces3 |
| O | Chd3 |
| O | Chd6 |
| O | Clic3 |
| O | Clstn1 |
| O | Cobl |
| O | Cobll1 |
| O | Col4a4 |
| O | Cox4i2 |
| O | Cpm |
| O | Crbn |
| O | Crebl2 |
| O | Crip2 |
| O | Csad |
| O | Csad |
| O | Cst3 |
| O | Ctdspl |
| O | Ctnnd1 |
| O | Cugbp2 |
| O | Cxcl15 |
| O | Cyb5b |
| O | Cyb5r3 |
| O | Cybrd1 |
| O | Cyp2b9 |
| O | Cyp2b9 |
| O | Cyp2j9 |
| O | Cyp39a1 |
| O | D10Ertd610e |
| O | D19Wsu12e |
| O | D1Ertd161e |
| O | D230025D16Rik |
| O | D330050I23Rik |
| O | D430015B01Rik |
| O | D4Bwg0951e |
| O | D630014A15Rik |
| O | D630039A03Rik |
| O | D730040F13Rik |
| O | D930005D10Rik |
| O | Daam2 |
| O | Dab2ip |
| O | Dach1 |
| O | Dcbld2 |
| O | Dcun1d2 |
| O | Ddc |
| O | Ddr2 |
| O | Dgka |
| O | Dhrs1 |
| O | Dhrs3 |
| O | Dixdc1 |
| O | Dmn |
| O | Dnm3 |
| O | Dock6 |
| O | Dpp4 |
| O | Dpp4 |
| O | Dtna |
| O | Dync1li2 |
| O | Dynlt3 |
| O | E130308A19Rik |
| O | E330009J07Rik |
| O | Ecm2 |
| O | Eda |
| O | Ednrb |
| O | Efemp1 |
| O | EG432995 |
| O | EG639396 |
| O | EG639426 |
| O | Egflam |
| O | Ehbp1 |
| O | Ehd4 |
| O | Eif2c1 |
| O | Eif2c4 |
| O | Eif2c4 |
| O | Elovl1 |
| O | Elovl5 |
| O | Emp2 |
| O | Enpep |
| O | ENSMUSG00000052439 |
| O | ENSMUST00000030142 |
| O | ENSMUST00000032357 |
| O | ENSMUST00000035300 |
| O | ENSMUST00000094652 |
| O | Epas1 |
| O | Epb4.1l4a |
| O | Epb4.1l5 |
| O | Epha1 |
| O | Ephb4 |
| O | Epn2 |
| O | Eps8l1 |
| O | Erg |
| O | Es22 |
| O | Etv1 |
| O | Evi1 |
| O | Faah |
| O | Fads1 |
| O | Fasn |
| O | Fat4 |
| O | Fbxl16 |
| O | Fbxl7 |
| O | Fbxo3 |
| O | Fbxo8 |
| O | Fcgbp |
| O | Fchsd2 |
| O | Fcmd |
| O | Fech |
| O | Fgd5 |
| O | Fgf1 |
| O | Fgf18 |
| O | Fgfr2 |
| O | Fgfr3 |
| O | Fgfr3 |
| O | Fgfr4 |
| O | Fgfr4 |
| O | Fhl1 |
| O | Figf |
| O | Fign |
| O | Fmo3 |
| O | Fmo5 |
| O | Fnta |
| O | Foxo1 |
| O | Foxp1 |
| O | Foxp2 |
| O | Frmd6 |
| O | Fzd2 |
| O | Fzd7 |
| O | Gab1 |
| O | Gabarapl1 |
| O | Galntl4 |
| O | Gdpd1 |
| O | Glb1l |
| O | Gm114 |
| O | Gnmt |
| O | Gpr125 |
| O | Gpr126 |
| O | Gpr30 |
| O | Gprasp1 |
| O | Gpsn2 |
| O | Grem2 |
| O | Grtp1 |
| O | Gstt3 |
| O | Gtf2i |
| O | Gucy1a3 |
| O | Gucy1b3 |
| O | Gucy1b3 |
| O | Gulp1 |
| O | Hcfc1r1 |
| O | Heca |
| O | Heca |
| O | Helz |
| O | Herpud1 |
| O | Hey1 |
| O | Hhip |
| O | Higd1b |
| O | Hkdc1 |
| O | Hmbox1 |
| O | Hmcn1 |
| O | Hnrph3 |
| O | Hoxa2 |
| O | Hpn |
| O | Hs2st1 |
| O | Hsd11b1 |
| O | Ica1 |
| O | Ica1 |
| O | Icam2 |
| O | Il17re |
| O | Irs1 |
| O | Itfg1 |
| O | Itga8 |
| O | Itga8 |
| O | Itm2b |
| O | Jak1 |
| O | Kbtbd3 |
| O | Kctd7 |
| O | Kif13a |
| O | Kif16b |
| O | Kif26a |
| O | Kitl |
| O | Klf9 |
| O | Klhdc8b |
| O | Klhl17 |
| O | Klhl24 |
| O | Klhl8 |
| O | Lace1 |
| O | Lama3 |
| O | Lamp3 |
| O | Lamp3 |
| O | Lamp3 |
| O | Lbx2 |
| O | Ldhb |
| O | Lims2 |
| O | Lmbr1 |
| O | Lpin1 |
| O | Lrp2 |
| O | Lrp2 |
| O | Lrp5 |
| O | Lrrk2 |
| O | Ltbp3 |
| O | Macf1 |
| O | Mageh1 |
| O | Magi3 |
| O | Mamdc2 |
| O | Map1lc3b |
| O | Map4k2 |
| O | Mapk1ip1 |
| O | Mapre2 |
| O | Mapt |
| O | Mapt |
| O | Mbip |
| O | Megf9 |
| O | Meox2 |
| O | Met |
| O | Mett5d1 |
| O | Mgst2 |
| O | Mid1ip1 |
| O | Mid2 |
| O | Mir16 |
| O | Mlc1 |
| O | Mllt6 |
| O | Mme |
| O | Mmp11 |
| O | Mobkl2b |
| O | Mospd2 |
| O | Mpp5 |
| O | Mtac2d1 |
| O | Mtus1 |
| O | Mtx3 |
| O | Muc1 |
| O | Muc1 |
| O | Mxra8 |
| O | Myh10 |
| O | Myh7 |
| O | Mylk |
| O | Mylk |
| O | Myo1d |
| O | Myo6 |
| O | Myo9a |
| O | NAP122883-1 |
| O | NAP123201-1 |
| O | Nap1l1 |
| O | Naprt1 |
| O | Napsa |
| O | Nbeal1 |
| O | Nbeal1 |
| O | Nck1 |
| O | Ndst1 |
| O | Nebl |
| O | Nebl |
| O | Nfia |
| O | Nfib |
| O | Nfib |
| O | Nhlrc2 |
| O | Nipa1 |
| O | Nisch |
| O | Nkd1 |
| O | Nme3 |
| O | Nostrin |
| O | Nox4 |
| O | Npnt |
| O | Npnt |
| O | Nrbp2 |
| O | Nrp1 |
| O | Numa1 |
| O | Numb |
| O | Oaz2 |
| O | Oaz2 |
| O | Ogn |
| O | Ogt |
| O | Omd |
| O | Osbpl5 |
| O | Osgep |
| O | OTTMUSG00000003947 |
| O | Oxr1 |
| O | Paip2 |
| O | Palmd |
| O | Pamci |
| O | Pard6g |
| O | Parva |
| O | Patz1 |
| O | Pcdh18 |
| O | Pcdha6 |
| O | Pcdha9 |
| O | Pcdha9 |
| O | Pcmtd2 |
| O | Pcolce2 |
| O | Pcyox1 |
| O | Pcyt2 |
| O | Pde4d |
| O | Pde8b |
| O | Pdlim2 |
| O | Pftk1 |
| O | Pgm2l1 |
| O | Phactr1 |
| O | Phactr1 |
| O | Phf17 |
| O | Phkb |
| O | Pias2 |
| O | Pigp |
| O | S1 |
| O | Pitpnc1 |
| O | Pitpnc1 |
| O | Pkia |
| O | Pkp2 |
| O | Plcg1 |
| O | Plekhb1 |
| O | Pltp |
| O | Pmp22 |
| O | Pomt2 |
| O | Pon1 |
| O | Postn |
| O | Ppfibp2 |
| O | Ppfibp2 |
| O | Ppid |
| O | Ppm1l |
| O | Ppp1r16a |
| O | pPtp4a3 |
| O | Prelp |
| O | Prickle1 |
| O | Prickle2 |
| O | Prkaa2 |
| O | Prkg1 |
| O | Prr8 |
| O | Pscd3 |
| O | Psd3 |
| O | Psd3 |
| O | Ptch1 |
| O | Ptplad1 |
| O | Ptprd |
| O | Ptprd |
| O | Ptprf |
| O | Ptprf |
| O | Ptprf |
| O | Ptprm |
| O | Ptprr |
| O | Ptprs |
| O | Ptrf |
| O | Ptrf |
| O | Purg |
| O | Pvrl3 |
| O | Pvrl3 |
| O | Pxmp4 |
| O | Qser1 |
| O | Rab11fip3 |
| O | Rab2b |
| O | Rabgap1l |
| O | Rabgap1l |
| O | Rad1 |
| O | Ramp2 |
| O | Rap1gap |
| O | Rasgrp2 |
| O | Rasl11a |
| O | Rasl12 |
| O | Rb1cc1 |
| O | Rbbp6 |
| O | Rbl2 |
| O | Rbm35b |
| O | Rbm35b |
| O | Rbpms |
| O | Reck |
| O | Reep6 |
| O | Rftn2 |
| O | Rgs3 |
| O | Rmnd5a |
| O | Rnase4 |
| O | Rnase4 |
| O | Rnf141 |
| O | Rnf144 |
| O | Rnf43 |
| O | Rora |
| O | Rora |
| O | Rsu1 |
| O | Rtn4rl1 |
| O | Sash1 |
| O | Scgb1a1 |
| O | Scnn1b |
| O | Scrn3 |
| O | Scrn3 |
| O | Scube2 |
| O | Scx |
| O | Sdpr |
| O | Sec14l2 |
| O | Sec14l3 |
| O | Sec14l3 |
| O | Sec14l3 |
| O | Sec63 |
| O | Sema3b |
| O | Sepp1 |
| O | Serinc5 |
| O | Sestd1 |
| O | Sft2d2 |
| O | Sftpa1 |
| O | Sftpa1 |
| O | Sftpb |
| O | Sgce |
| O | Sgsh |
| O | Sh3md4 |
| O | Sh3tc2 |
| O | Shroom1 |
| O | Sipa1l2 |
| O | Slc13a4 |
| O | Slc16a9 |
| O | Slc23a1 |
| O | Slc24a3 |
| O | Slc25a10 |
| O | Slc25a35 |
| O | Slc29a1 |
| O | Slc31a1 |
| O | Slc34a2 |
| O | Slc34a2 |
| O | Slc38a5 |
| O | Slc7a4 |
| O | Smarca2 |
| O | Smpd2 |
| O | Snai2 |
| O | Sned1 |
| O | Snx13 |
| O | Snx21 |
| O | Snx25 |
| O | Sod1 |
| O | Sort1 |
| O | Sort1 |
| O | Sox18 |
| O | Specc1l |
| O | Spna2 |
| O | Spnb2 |
| O | Spnb2 |
| O | Spock2 |
| O | Spock2 |
| O | Spry1 |
| O | Srpx |
| O | St5 |
| O | Stau2 |
| O | Stk36 |
| O | Stmn2 |
| O | Strn |
| O | Stx19 |
| O | Suv420h2 |
| O | Svep1 |
| O | Syne1 |
| O | Syne2 |
| O | Taf9b |
| O | Tbcel |
| O | Tbx3 |
| O | Tbx6 |
| O | TC1605426 |
| O | Tcn2 |
| O | Tcp11l2 |
| O | Tcp11l2 |
| O | Tek |
| O | Tenc1 |
| O | Tgfb3 |
| O | Thbd |
| O | Thbs3 |
| O | Thrb |
| O | Tie1 |
| O | Tinag |
| O | Tjp1 |
| O | Tle2 |
| O | Tle6 |
| O | Tmcc2 |
| O | Tmeff1 |
| O | Tmem100 |
| O | Tmem106b |
| O | Tmem109 |
| O | Tmem110 |
| O | Tmem123 |
| O | Tmem150 |
| O | Tmem16a |
| O | Tmem32 |
| O | Tmem41a |
| O | Tmem42 |
| O | Tmem50b |
| O | Tmem59 |
| O | Tmod1 |
| O | Tnfrsf19 |
| O | Tnfsf13b |
| O | Tnik |
| O | Tns1 |
| O | Tns3 |
| O | Tpcn1 |
| O | Trib2 |
| O | Trim2 |
| O | Trp53bp2 |
| O | Trp53inp2 |
| O | Tspan12 |
| O | Tspan13 |
| O | Tspan18 |
| O | Tspan2 |
| O | Tspan7 |
| O | Ttc28 |
| O | Ttc28 |
| O | Ttll1 |
| O | Twsg1 |
| O | Ube2h |
| O | Ubr1 |
| O | Ubr1 |
| O | Ulk2 |
| O | Usp33 |
| O | Usp46 |
| O | Vamp4 |
| O | Vamp5 |
| O | Vegfa |
| O | Vegfa |
| O | Vgll4 |
| O | Vldlr |
| O | Vps13a |
| O | Vsnl1 |
| O | Vtn |
| O | Vwf |
| O | Wbp1 |
| O | Wdfy3 |
| O | Wdr45 |
| O | Wdr6 |
| O | Wnt3a |
| O | Wscd1 |
| O | Wwp1 |
| O | Wwp1 |
| O | Xpc |
| O | Ypel3 |
| O | Zbtb10 |
| O | Zbtb20 |
| O | Zeb1 |
| O | Zfp2 |
| O | Zfp277 |
| O | Zfp532 |
| O | Zfp579 |
| O | Zfp592 |
| O | Zfp612 |
| O | Zfp647 |
| O | Zfp652 |
| O | Zhx1 |
| O | Zrsr1 |
| P | 1110013L07Rik |
| P | 1110018J18Rik |
| P | 1190007I07Rik |
| P | 1700034H14Rik |
| P | 1810020D17Rik |
| P | 2210010B09Rik |
| P | 2210021J22Rik |
| P | 2310001A20Rik |
| P | 2310016C16Rik |
| P | 2310045A20Rik |
| P | 2310045N01Rik |
| P | 2510048L02Rik |
| P | 2510048L02Rik |
| P | 2610003J06Rik |
| P | 2610027H17Rik |
| P | 2610036F08Rik |
| P | 2810417J12Rik |
| P | 3110001I20Rik |
| P | 3110009E18Rik |
| P | 3110050N22Rik |
| P | 3321401G04Rik |
| P | 4631416L12Rik |
| P | 4631426J05Rik |
| P | 4732474O15Rik |
| P | 4833428M15Rik |
| P | 4833442J19Rik |
| P | 4930502E18Rik |
| P | 4930504E06Rik |
| P | 4930588G05Rik |
| P | 4932442K08Rik |
| P | 4933404M19Rik |
| P | 5730406M06Rik |
| P | 5730593F17Rik |
| P | 5830404H04Rik |
| P | 5830428H23Rik |
| P | 6330403L08Rik |
| P | 6330416L11Rik |
| P | 6720489N17Rik |
| P | 8430416G17Rik |
| P | 9630023C09Rik |
| P | A130022F02Rik |
| P | A230062I15Rik |
| P | A230092J17Rik |
| P | Aasdhppt |
| P | Abca1 |
| P | Abcc5 |
| P | Abtb1 |
| P | Acaa1b |
| P | Acp6 |
| P | Adamts10 |
| P | Adcy2 |
| P | Add3 |
| P | Agbl3 |
| P | Agbl3 |
| P | Ahnak |
| P | AI851716 |
| P | AJ237917 |
| P | AK011803 |
| P | AK031552 |
| P | AK031632 |
| P | AK032764 |
| P | AK033365 |
| P | AK034320 |
| P | AK035307 |
| P | AK035337 |
| P | AK043743 |
| P | AK044272 |
| P | AK044903 |
| P | AK046895 |
| P | AK047616 |
| P | AK047905 |
| P | AK048657 |
| P | AK050084 |
| P | AK051949 |
| P | AK053596 |
| P | AK054073 |
| P | AK078322 |
| P | AK078794 |
| P | AK079264 |
| P | AK079806 |
| P | AK084144 |
| P | AK084349 |
| P | AK085332 |
| P | AK085706 |
| P | AK085783 |
| P | AK087326 |
| P | AK089858 |
| P | AK090299 |
| P | Akr1b3 |
| P | Aldh1l1 |
| P | Amph |
| P | Anapc1 |
| P | Ang1 |
| P | Ang2 |
| P | Ang4 |
| P | Ankrd24 |
| P | Ankrd50 |
| P | Apln |
| P | App |
| P | Aprin |
| P | Arl3 |
| P | Atbf1 |
| P | Atg16l2 |
| P | Atp6v0a4 |
| P | AW986112 |
| P | B230333C21Rik |
| P | Bace2 |
| P | BB333095 |
| P | BC010787 |
| P | BC020535 |
| P | BC060632 |
| P | Bcl2 |
| P | Bhlhb9 |
| P | Bmp1 |
| P | Bmp5 |
| P | Brwd1 |
| P | C030044C12Rik |
| P | C230078M08Rik |
| P | C330016O10Rik |
| P | C85492 |
| P | Canx |
| P | Caprin2 |
| P | Car14 |
| P | Carkl |
| P | CB845695 |
| P | Cbx6 |
| P | Cbx6 |
| P | Cbx6 |
| P | Ccdc46 |
| P | Ccdc52 |
| P | Ccdc85b |
| P | Ccni |
| P | Ccnt2 |
| P | Ccny |
| P | Ccr6 |
| P | Cd59a |
| P | Cd59b |
| P | Cdc14b |
| P | Cdc2l6 |
| P | Cdh11 |
| P | Cdh11 |
| P | Cdkn1b |
| P | Cdkn2c |
| P | Cdkn2c |
| P | Cdr2l |
| P | Cebpa |
| P | Cep70 |
| P | Cetn2 |
| P | Chd6 |
| P | Cnnm2 |
| P | Col16a1 |
| P | Col4a6 |
| P | Colec12 |
| P | Colec12 |
| P | Coq7 |
| P | Coq7 |
| P | Ctnna1 |
| P | Ctnnb1 |
| P | Cxx1c |
| P | Cyp26b1 |
| P | D030011O10Rik |
| P | D14Ertd500e |
| P | D16Bwg1494e |
| P | D18Ertd653e |
| P | D430007A19Rik |
| P | D4Wsu53e |
| P | D830024N08Rik |
| P | Dapk1 |
| P | Dbp |
| P | Dchs1 |
| P | Ddx5 |
| P | Dock1 |
| P | Dock4 |
| P | Dock4 |
| P | Dohh |
| P | Dus1l |
| P | Dyrk1b |
| P | E030026E10Rik |
| P | Ebf1 |
| P | Ece1 |
| P | Efnb1 |
| P | EG665123 |
| P | Eif4a2 |
| P | Eml1 |
| P | ENSMUSG00000052188 |
| P | ENSMUSG00000073981 |
| P | ENSMUSG00000075516 |
| P | ENSMUST00000035340 |
| P | ENSMUST00000054524 |
| P | Epb4.1 |
| P | Exosc7 |
| P | F2r |
| P | Fads3 |
| P | Fads3 |
| P | Fancb |
| P | Fat1 |
| P | Fbln1 |
| P | Fcgrt |
| P | Fdx1 |
| P | Fdx1 |
| P | Fech |
| P | Fhod1 |
| P | Fibp |
| P | Flt1 |
| P | Fnbp1l |
| P | Foxo6 |
| P | Fzd3 |
| P | Fzd8 |
| P | Gata2 |
| P | Gata2 |
| P | Gcc2 |
| P | Gdap5 |
| P | Gdpd1 |
| P | Gfod1 |
| P | Ggcx |
| P | Glul |
| P | Gpm6a |
| P | Gpm6a |
| P | Hectd3 |
| P | Hint2 |
| P | Hlf |
| P | Hmgcll1 |
| P | Hmha1 |
| P | Hoxa7 |
| P | Hrsp12 |
| P | Hsd17b4 |
| P | Hsp90b1 |
| P | Hspa4 |
| P | Ick |
| P | Id4 |
| P | Id4 |
| P | Idh1 |
| P | Ift80 |
| P | Igf1r |
| P | Igsf3 |
| P | Il11ra1 |
| P | Il11ra1 |
| P | Impdh1 |
| P | Iqsec1 |
| P | Iqsec1 |
| P | Irf2bp2 |
| P | Itga6 |
| P | Kcnb1 |
| P | Kcne2 |
| P | Kctd1 |
| P | Kctd15 |
| P | Kdelc2 |
| P | Kdelc2 |
| P | Kif26b |
| P | Klf9 |
| P | Klhl13 |
| P | Lama4 |
| P | Lbh |
| P | LOC636537 |
| P | Lphn1 |
| P | Lphn1 |
| P | Lpin2 |
| P | Ltbp3 |
| P | Lycat |
| P | M77174 |
| P | Mapkbp1 |
| P | Mark3 |
| P | Mcee |
| P | Meis1 |
| P | Mllt3 |
| P | Mmd |
| P | Mmp28 |
| P | Morn2 |
| P | Mpdz |
| P | Mpp7 |
| P | Mpp7 |
| P | Mpv17 |
| P | Mpv17l |
| P | Mrc2 |
| P | Mrg1 |
| P | Mrg1 |
| P | Mtap6 |
| P | Mtap6 |
| P | Mxd4 |
| P | Mxd4 |
| P | NAP046120-1 |
| P | NAP101459-1 |
| P | Nap1l3 |
| P | Ndufb2 |
| P | Nedd4l |
| P | Nek1 |
| P | Nfib |
| P | Nipsnap1 |
| P | Nisch |
| P | Notch4 |
| P | Npal2 |
| P | Npal2 |
| P | Nr1d1 |
| P | Nr1d2 |
| P | Nup210 |
| P | Osbpl2 |
| P | Otud1 |
| P | Pabpn1 |
| P | Paqr4 |
| P | Pbx1 |
| P | Pcdhb6 |
| P | Pcdhga7 |
| P | Pcdhgb4 |
| P | Pdcd4 |
| P | Pdcd6ip |
| P | Pdgfa |
| P | Pdlim3 |
| P | Pdlim3 |
| P | Pecam1 |
| P | Pex10 |
| P | Phpt1 |
| P | Plcl1 |
| P | Plekha6 |
| P | Pnpla6 |
| P | Pon3 |
| P | Pon3 |
| P | Ppic |
| P | Prdx6 |
| P | Prdx6 |
| P | Prelp |
| P | Prickle1 |
| P | Prmt8 |
| P | Prps2 |
| P | Prss36 |
| P | Ptgis |
| P | Ptk2 |
| P | Ptpn14 |
| P | Rapgef4 |
| P | Rarg |
| P | Rasal2 |
| P | Rbp1 |
| P | Rcn3 |
| P | Rcn3 |
| P | Recql |
| P | Rgl2 |
| P | Rgs12 |
| P | Rnf141 |
| P | S100a1 |
| P | Sall2 |
| P | Saps3 |
| P | Scnn1g |
| P | Scrn2 |
| P | Sdc2 |
| P | Sema3g |
| P | Sgms1 |
| P | Shank3 |
| P | Sirt4 |
| P | Six5 |
| P | Slc1a5 |
| P | Slc2a13 |
| P | Slc43a3 |
| P | Slc44a1 |
| P | Slc44a2 |
| P | Slc46a1 |
| P | Slc46a3 |
| P | Slc6a2 |
| P | Slc6a9 |
| P | Slit2 |
| P | Smo |
| P | Snca |
| P | Snx22 |
| P | Sorbs3 |
| P | Sort1 |
| P | Sox17 |
| P | Sox17 |
| P | Sparcl1 |
| P | Sparcl1 |
| P | Spnb2 |
| P | Ssbp3 |
| P | St8sia4 |
| P | St8sia4 |
| P | Stard13 |
| P | Stard9 |
| P | Stard9 |
| P | Syn2 |
| P | Synj2 |
| P | Synj2bp |
| P | Taok2 |
| P | Tbx3 |
| P | TC1605825 |
| P | TC1614162 |
| P | TC1638968 |
| P | TC1643662 |
| P | TC1663189 |
| P | TC1677116 |
| P | TC1697186 |
| P | Tcta |
| P | Tead2 |
| P | Tef |
| P | Terf2ip |
| P | Tfdp2 |
| P | Tmco1 |
| P | Tmco3 |
| P | Tmem14a |
| P | Tmem16k |
| P | Tmem176a |
| P | Tmem176a |
| P | Tmem176b |
| P | Tmem29 |
| P | Tmem4 |
| P | Tmem9 |
| P | Tmem98 |
| P | Tmtc2 |
| P | Tns1 |
| P | Tns1 |
| P | Trim24 |
| P | Trim37 |
| P | Trpt1 |
| P | Tspan6 |
| P | Tspan6 |
| P | Ttc3 |
| P | Ube2b |
| P | Ubr1 |
| P | Usp54 |
| P | Vamp1 |
| P | Vegfb |
| P | Vegfc |
| P | Vkorc1 |
| P | Wdfy3 |
| P | X12807 |
| P | X99384 |
| P | Xpot |
| P | Zdhhc2 |
| P | Zdhhc2 |
| P | Zdhhc3 |
| P | Zfp26 |
| P | Zfp704 |
| P | Zhx3 |
| P | Zmym6 |
| Q | 2-Mar |
| Q | 6-Mar |
| Q | 6-Sep |
| Q | 0610007C21Rik |
| Q | 1110021J02Rik |
| Q | 1110028C15Rik |
| Q | 1190002H23Rik |
| Q | 1300007F04Rik |
| Q | 1600020E01Rik |
| Q | 2210010C04Rik |
| Q | 2310002J21Rik |
| Q | 2310005E10Rik |
| Q | 2310010J17Rik |
| Q | 2310016C08Rik |
| Q | 2310016C08Rik |
| Q | 2310061J03Rik |
| Q | 2400001E08Rik |
| Q | 2410014A08Rik |
| Q | 2410014A08Rik |
| Q | 2410017P07Rik |
| Q | 2410187C16Rik |
| Q | 2510003E04Rik |
| Q | 2600005O03Rik |
| Q | 2600005O03Rik |
| Q | 2610002D18Rik |
| Q | 2610020H08Rik |
| Q | 2610029G23Rik |
| Q | 2610036L11Rik |
| Q | 2610110G12Rik |
| Q | 2610110G12Rik |
| Q | 2700038N03Rik |
| Q | 2700049P18Rik |
| Q | 2700094K13Rik |
| Q | 2700097O09Rik |
| Q | 2810055G20Rik |
| Q | 2810417H13Rik |
| Q | 2810453I06Rik |
| Q | 2810459M11Rik |
| Q | 2810482I07Rik |
| Q | 3110009E18Rik |
| Q | 3110043A19Rik |
| Q | 4631427C17Rik |
| Q | 4732460I02Rik |
| Q | 4921505C17Rik |
| Q | 4921517D21Rik |
| Q | 4930431P19Rik |
| Q | 4930509H03Rik |
| Q | 4930523C07Rik |
| Q | 4930523C11Rik |
| Q | 4930535E21Rik |
| Q | 4930567H12Rik |
| Q | 4930570C03Rik |
| Q | 4931406C07Rik |
| Q | 4932442K08Rik |
| Q | 4933400C05Rik |
| Q | 4933400E14Rik |
| Q | 4933403F05Rik |
| Q | 4933407H18Rik |
| Q | 5830418K08Rik |
| Q | 5830469G19Rik |
| Q | 6330409N04Rik |
| Q | 6720467C03Rik |
| Q | 6720473M08Rik |
| Q | 9030419F21Rik |
| Q | A_51_P327496 |
| Q | A_52_P1197466 |
| Q | A430107D22Rik |
| Q | A430107O13Rik |
| Q | A730081D07Rik |
| Q | A930025D01Rik |
| Q | AA438147 |
| Q | Abca7 |
| Q | Abi3 |
| Q | Acta2 |
| Q | Adam15 |
| Q | Adamts12 |
| Q | Adamtsl3 |
| Q | Adrb2 |
| Q | Aga |
| Q | Agmat |
| Q | AI449175 |
| Q | Air |
| Q | AK012436 |
| Q | AK028719 |
| Q | AK031703 |
| Q | AK032178 |
| Q | AK033087 |
| Q | AK035359 |
| Q | AK035407 |
| Q | AK036075 |
| Q | AK037381 |
| Q | AK037470 |
| Q | AK039370 |
| Q | AK042636 |
| Q | AK045222 |
| Q | AK045982 |
| Q | AK047023 |
| Q | AK047281 |
| Q | AK047447 |
| Q | AK048147 |
| Q | AK048514 |
| Q | AK049913 |
| Q | AK051621 |
| Q | AK051961 |
| Q | AK051988 |
| Q | AK052617 |
| Q | AK076350 |
| Q | AK078992 |
| Q | AK080372 |
| Q | AK082620 |
| Q | AK083880 |
| Q | AK084575 |
| Q | AK084593 |
| Q | AK084634 |
| Q | AK084897 |
| Q | AK087246 |
| Q | AK136420 |
| Q | Akap2 |
| Q | Akp2 |
| Q | Akt3 |
| Q | Anln |
| Q | Anxa3 |
| Q | Arl4a |
| Q | Arl4a |
| Q | Arl4d |
| Q | Armcx2 |
| Q | Armcx5 |
| Q | Asf1b |
| Q | B230208H17Rik |
| Q | B430006D22Rik |
| Q | B930095M22Rik |
| Q | Bace2 |
| Q | Baiap2 |
| Q | Banp |
| Q | BB146404 |
| Q | Bbs4 |
| Q | Bbs5 |
| Q | Bbs5 |
| Q | BC003993 |
| Q | BC011209 |
| Q | BC019943 |
| Q | BC024479 |
| Q | BC024694 |
| Q | BC031426 |
| Q | BC038822 |
| Q | BC050196 |
| Q | BC051227 |
| Q | BC051628 |
| Q | Bckdhb |
| Q | Bckdhb |
| Q | Bcl11a |
| Q | Bcl11b |
| Q | Bcl11b |
| Q | Bcl6b |
| Q | Bcs1l |
| Q | BF642829 |
| Q | Birc5 |
| Q | Brca1 |
| Q | Brdt |
| Q | Bsg |
| Q | Btbd7 |
| Q | Bub1b |
| Q | C1r |
| Q | C2 |
| Q | C230094A16Rik |
| Q | C230096C10Rik |
| Q | Cacybp |
| Q | Caprin1 |
| Q | Casc5 |
| Q | Cbara1 |
| Q | Cbx5 |
| Q | Ccdc117 |
| Q | Ccdc34 |
| Q | Ccdc9 |
| Q | Ccna2 |
| Q | Ccnb1 |
| Q | Ccnl2 |
| Q | Ccnl2 |
| Q | Ccnl2 |
| Q | Ccnt1 |
| Q | Cd164l2 |
| Q | Cd244 |
| Q | Cd244 |
| Q | Cd247 |
| Q | Cd247 |
| Q | Cd248 |
| Q | Cd248 |
| Q | Cd47 |
| Q | Cd47 |
| Q | Cd96 |
| Q | Cdc20 |
| Q | Cdc42ep1 |
| Q | Cdca2 |
| Q | Cdca3 |
| Q | Cdca5 |
| Q | Cdca8 |
| Q | Cdkn3 |
| Q | Cenpa |
| Q | Cenpe |
| Q | Cenph |
| Q | Cenpj |
| Q | Cep55 |
| Q | Chaf1a |
| Q | Chchd8 |
| Q | Chid1 |
| Q | Chid1 |
| Q | Chka |
| Q | Chka |
| Q | Chordc1 |
| Q | Chrm4 |
| Q | Chrna7 |
| Q | Chst11 |
| Q | Ciita |
| Q | Cirbp |
| Q | Cited2 |
| Q | Cks1b |
| Q | Cnih2 |
| Q | Cnn1 |
| Q | Col1a1 |
| Q | Col3a1 |
| Q | Col5a1 |
| Q | Col5a2 |
| Q | Col6a1 |
| Q | Cpt1c |
| Q | Creb3 |
| Q | Creb3l3 |
| Q | Crebbp |
| Q | Cs |
| Q | Csf1r |
| Q | Ctsw |
| Q | Cttnbp2nl |
| Q | Cxcr3 |
| Q | D16H22S680E |
| Q | D17Wsu104e |
| Q | D19Bwg1357e |
| Q | D330028D13Rik |
| Q | D6Wsu176e |
| Q | Daglb |
| Q | Dcp1b |
| Q | Dennd3 |
| Q | Dhh |
| Q | Dhx29 |
| Q | Dhx33 |
| Q | Dkkl1 |
| Q | Dleu2 |
| Q | Dll1 |
| Q | Dnaja1 |
| Q | Dnaja1 |
| Q | Dnajb1 |
| Q | Dnajc15 |
| Q | Dnm1 |
| Q | Dock10 |
| Q | Dock8 |
| Q | Dph1 |
| Q | Dpp7 |
| Q | Dtl |
| Q | Dtymk |
| Q | Dut |
| Q | Dut |
| Q | DV651670 |
| Q | E130106K03Rik |
| Q | E130306D19Rik |
| Q | E130311K13Rik |
| Q | E2f7 |
| Q | Ecgf1 |
| Q | Ecm1 |
| Q | Ect2 |
| Q | Efs |
| Q | EG619750 |
| Q | EG621431 |
| Q | Elf4 |
| Q | Elmo1 |
| Q | Eme1 |
| Q | Enpp4 |
| Q | ENSMUSG00000050599 |
| Q | ENSMUST00000039827 |
| Q | ENSMUST00000084306 |
| Q | ENSMUST00000100305 |
| Q | ENSMUST00000100870 |
| Q | ENSMUST00000103291 |
| Q | ENSMUST00000103507 |
| Q | Eny2 |
| Q | Epc1 |
| Q | Eps15l1 |
| Q | Ercc6l |
| Q | Errfi1 |
| Q | Eya3 |
| Q | F730002C09Rik |
| Q | Fasl |
| Q | Fbxl12 |
| Q | Fbxo5 |
| Q | Fcho1 |
| Q | Fkbp1b |
| Q | Fnbp1 |
| Q | Fzd7 |
| Q | Fzd8 |
| Q | G0s2 |
| Q | G6pdx |
| Q | Gabbr2 |
| Q | Gabrb2 |
| Q | Gadd45gip1 |
| Q | Gart |
| Q | Gats |
| Q | Gcnt1 |
| Q | Gcnt1 |
| Q | Gfi1 |
| Q | Gga2 |
| Q | Ggps1 |
| Q | Ghr |
| Q | Ghr |
| Q | Gimap1 |
| Q | Git2 |
| Q | Gja7 |
| Q | Gja7 |
| Q | Glipr1 |
| Q | Gm1418 |
| Q | Gm189 |
| Q | Gm2a |
| Q | Gnai1 |
| Q | Gpr124 |
| Q | Gpx7 |
| Q | Grap |
| Q | Grb10 |
| Q | Grin3b |
| Q | Grit |
| Q | Gspt1 |
| Q | Gtl2 |
| Q | Gtl2 |
| Q | Gusb |
| Q | Gzmb |
| Q | H2afx |
| Q | Hells |
| Q | Hexim1 |
| Q | Hist1h1a |
| Q | Hist1h1b |
| Q | Hist1h2aa |
| Q | Hist3h2a |
| Q | Hmga2 |
| Q | Hoxb5 |
| Q | Hpcal1 |
| Q | Hsdl2 |
| Q | Hsp110 |
| Q | Hsp90ab1 |
| Q | Hspa1a |
| Q | Hspa1a |
| Q | Hspb1 |
| Q | Hspbap1 |
| Q | Id1 |
| Q | Id1 |
| Q | Igf1 |
| Q | Igh-6 |
| Q | Ikbkg |
| Q | Ikbkg |
| Q | Ikzf1 |
| Q | Ikzf4 |
| Q | Il27ra |
| Q | Il2rb |
| Q | Isyna1 |
| Q | Ivns1abp |
| Q | Jub |
| Q | Jup |
| Q | Kalrn |
| Q | Kcnt1 |
| Q | Kif1c |
| Q | Kif20a |
| Q | Kif22 |
| Q | Kif23 |
| Q | Kif4 |
| Q | Kifc1 |
| Q | Klc3 |
| Q | Klf13 |
| Q | Klf2 |
| Q | Klf4 |
| Q | Klf7 |
| Q | Klf7 |
| Q | Klhl2 |
| Q | Kmo |
| Q | Kptn |
| Q | Krt80 |
| Q | L01776 |
| Q | L01776 |
| Q | l7Rn6 |
| Q | Lck |
| Q | Ldb2 |
| Q | Lef1 |
| Q | Lmcd1 |
| Q | LOC546100 |
| Q | Lphn3 |
| Q | Lrch1 |
| Q | Lrrc8a |
| Q | Lrtm2 |
| Q | Lxn |
| Q | Mad2l1 |
| Q | Map3k6 |
| Q | Map4k1 |
| Q | Mapkapk2 |
| Q | Mastl |
| Q | Mccc2 |
| Q | Mcm5 |
| Q | Me2 |
| Q | Met |
| Q | Mfap2 |
| Q | Mib2 |
| Q | Mif |
| Q | Mitd1 |
| Q | Mki67 |
| Q | Mki67 |
| Q | Mlh3 |
| Q | Mmab |
| Q | Mmp2 |
| Q | Mrpl35 |
| Q | Mrps25 |
| Q | Mrps6 |
| Q | Mrps6 |
| Q | Mtch1 |
| Q | Mtch2 |
| Q | Mtf1 |
| Q | Mthfd2 |
| Q | Mxra7 |
| Q | Myh11 |
| Q | Myl9 |
| Q | Myo1c |
| Q | Myrip |
| Q | NAP027922-1 |
| Q | NAP028620-1 |
| Q | NAP029297-1 |
| Q | NAP034624-1 |
| Q | NAP047434-1 |
| Q | NAP056427-1 |
| Q | NAP058291-1 |
| Q | NAP091715-1 |
| Q | NAP103523-1 |
| Q | NAP105744-1 |
| Q | NAP120816-001 |
| Q | Ncaph |
| Q | Ndufa3 |
| Q | Ndufab1 |
| Q | Nes |
| Q | Nfe2l2 |
| Q | Nfrkb |
| Q | Nfya |
| Q | Nid2 |
| Q | Nisch |
| Q | Nkg7 |
| Q | Nkg7 |
| Q | Nnt |
| Q | Nos3 |
| Q | Notch1 |
| Q | NP064425 |
| Q | Nphp3 |
| Q | Nrarp |
| Q | Nsl1 |
| Q | Ntn2l |
| Q | Ntrk1 |
| Q | Numb |
| Q | Nusap1 |
| Q | Nxn |
| Q | Ocln |
| Q | Odz4 |
| Q | Olfr569 |
| Q | Olfr821 |
| Q | P4ha1 |
| Q | Paf1 |
| Q | Pard6b |
| Q | Pard6b |
| Q | Parva |
| Q | Pcaf |
| Q | Pcdhb12 |
| Q | Pcp2 |
| Q | Pcsk1n |
| Q | Pde1a |
| Q | Pde1a |
| Q | Pde9a |
| Q | Pdgfra |
| Q | Pdia4 |
| Q | Pdia6 |
| Q | Pdlim2 |
| Q | Per1 |
| Q | Phf16 |
| Q | Phf19 |
| Q | Pias3 |
| Q | Pik3r3 |
| Q | Pitpnb |
| Q | Plekhf1 |
| Q | Plk1 |
| Q | Plk4 |
| Q | Plxna2 |
| Q | Plxna2 |
| Q | Plxna2 |
| Q | Plxnc1 |
| Q | Pmf1 |
| Q | Pnpla2 |
| Q | Podxl |
| Q | Pofut2 |
| Q | Pofut2 |
| Q | Pola1 |
| Q | Pon2 |
| Q | Porcn |
| Q | Ppm1a |
| Q | Ppp1r13b |
| Q | Ppt1 |
| Q | Prcp |
| Q | Prdx4 |
| Q | Prf1 |
| Q | Prim1 |
| Q | Prkch |
| Q | Psap |
| Q | Ptplad2 |
| Q | Ptprcap |
| Q | Ptprk |
| Q | Ptprk |
| Q | Rab3il1 |
| Q | Rab40c |
| Q | Racgap1 |
| Q | Rad51 |
| Q | Rad51l3 |
| Q | Rad54l |
| Q | Raf1 |
| Q | Rai14 |
| Q | Rai14 |
| Q | Ralgps1 |
| Q | Ralgps2 |
| Q | Ranbp3 |
| Q | Rcn1 |
| Q | Rin3 |
| Q | RP23-136K12.4 |
| Q | Rpap1 |
| Q | Rrp1b |
| Q | Rtbdn |
| Q | S100a3 |
| Q | Sbds |
| Q | Sema6d |
| Q | Senp7 |
| Q | Serpinb1a |
| Q | Serpinf1 |
| Q | Serpinh1 |
| Q | Sfxn1 |
| Q | Sgk |
| Q | Sh2d3c |
| Q | Shcbp1 |
| Q | Shroom2 |
| Q | Slc10a6 |
| Q | Slc14a2 |
| Q | Slc25a35 |
| Q | Slc30a6 |
| Q | Slc35d1 |
| Q | Slc5a3 |
| Q | Slc6a14 |
| Q | Slc6a14 |
| Q | Smc2 |
| Q | Snhg8 |
| Q | Snrpn |
| Q | Spag5 |
| Q | Spag9 |
| Q | Spata21 |
| Q | Spata7 |
| Q | Ss18l1 |
| Q | Ssbp2 |
| Q | St6galnac5 |
| Q | St6galnac6 |
| Q | Stip1 |
| Q | Stk3 |
| Q | Stx16 |
| Q | Stxbp3a |
| Q | Stxbp5 |
| Q | Sulf1 |
| Q | Susd3 |
| Q | Syce2 |
| Q | Tagln |
| Q | Tagln |
| Q | Tanc1 |
| Q | Taok2 |
| Q | Tbc1d9b |
| Q | Tbkbp1 |
| Q | Tbx4 |
| Q | Tbx4 |
| Q | TC1633516 |
| Q | TC1651824 |
| Q | TC1675885 |
| Q | TC1676032 |
| Q | TC1682954 |
| Q | TC1683047 |
| Q | TC1704634 |
| Q | TC1708930 |
| Q | TC1708996 |
| Q | TC1779078 |
| Q | Tceb3 |
| Q | Tcrb-V13 |
| Q | Tcte2 |
| Q | Terc |
| Q | Tert |
| Q | Tesk2 |
| Q | Tfpi |
| Q | Thoc1 |
| Q | Thrap2 |
| Q | Tiam1 |
| Q | Timp3 |
| Q | Tinagl |
| Q | Tlcd1 |
| Q | Tm6sf1 |
| Q | Tmbim1 |
| Q | Tmbim1 |
| Q | Tmem112 |
| Q | Tmem128 |
| Q | Tmem134 |
| Q | Tmem37 |
| Q | Tmem48 |
| Q | Tmod3 |
| Q | Tnfrsf13c |
| Q | Tnks1bp1 |
| Q | Tnrc15 |
| Q | Tnrc6b |
| Q | Tomm40l |
| Q | Top1mt |
| Q | Top2a |
| Q | Tpm2 |
| Q | Trem2 |
| Q | Trem2 |
| Q | Trim59 |
| Q | Trip13 |
| Q | Trp53inp1 |
| Q | Trpc2 |
| Q | Tsc1 |
| Q | Tsc22d3 |
| Q | Tsc22d3 |
| Q | Tsga10 |
| Q | Tspan32 |
| Q | Ttc19 |
| Q | Ttc21b |
| Q | Tubb5 |
| Q | Tubb5 |
| Q | Tusc5 |
| Q | Tyms |
| Q | Tyms |
| Q | Tyms-ps |
| Q | Tyro3 |
| Q | Ube2n |
| Q | Ubfd1 |
| Q | Ugt2b34 |
| Q | Uhrf2 |
| Q | Ung |
| Q | Uros |
| Q | Vars2 |
| Q | Vps29 |
| Q | Vps37d |
| Q | Wdfy4 |
| Q | Wdhd1 |
| Q | Wdr32 |
| Q | Wdr67 |
| Q | Wisp2 |
| Q | Wnk1 |
| Q | Wtip |
| Q | Wwox |
| Q | X83328 |
| Q | Xcl1 |
| Q | Xlkd1 |
| Q | Yipf6 |
| Q | Zc3h6 |
| Q | Zfp142 |
| Q | Zfp383 |
| Q | Zfp606 |
| Q | Zfp650 |
| Q | Znf512b |
| Q | Zxdc |
| R | 4-Mar |
| R | 1110004E09Rik |
| R | 1110017D15Rik |
| R | 1110032A03Rik |
| R | 1190002A17Rik |
| R | 1190002J23Rik |
| R | 1190007F08Rik |
| R | 1600029D21Rik |
| R | 1700001L19Rik |
| R | 1700003M02Rik |
| R | 1700009P17Rik |
| R | 1700010A17Rik |
| R | 1700016K19Rik |
| R | 1700021K14Rik |
| R | 1700025K23Rik |
| R | 1700026D08Rik |
| R | 1700026L06Rik |
| R | 1700027N10Rik |
| R | 1700030J22Rik |
| R | 1700088E04Rik |
| R | 1700094D03Rik |
| R | 1700123D08Rik |
| R | 1810007P19Rik |
| R | 1810008I18Rik |
| R | 1810048J11Rik |
| R | 2010007H06Rik |
| R | 2010300C02Rik |
| R | 2310007A19Rik |
| R | 2610015P09Rik |
| R | 2610028H24Rik |
| R | 2810002I04Rik |
| R | 2900046G09Rik |
| R | 3300002A11Rik |
| R | 4732415M23Rik |
| R | 4833401D15Rik |
| R | 4833436C18Rik |
| R | 4921509J17Rik |
| R | 4930430E16Rik |
| R | 4930430E16Rik |
| R | 4930431B11Rik |
| R | 4930444P10Rik |
| R | 4930455F23Rik |
| R | 4930535E21Rik |
| R | 4930562C15Rik |
| R | 4932425I24Rik |
| R | 4933404M02Rik |
| R | 4933404M02Rik |
| R | 4933430H15Rik |
| R | 4933430H15Rik |
| R | 5430414B12Rik |
| R | 6330439K17Rik |
| R | 6430537H07Rik |
| R | 6820408C15Rik |
| R | 9330101J02Rik |
| R | 9630019K15Rik |
| R | 9830169C18Rik |
| R | A530050D06Rik |
| R | A530088I07Rik |
| R | Acpl2 |
| R | Acsl3 |
| R | AF143539 |
| R | Agbl2 |
| R | Agr2 |
| R | Aim1l |
| R | AK033818 |
| R | Ak7 |
| R | Akap14 |
| R | Ankrd42 |
| R | Ankrd5 |
| R | Ankrd5 |
| R | Arhgap18 |
| R | Arhgdig |
| R | Arhgef4 |
| R | Atp2c2 |
| R | AV249152 |
| R | AV249152 |
| R | B3gnt4 |
| R | BC007180 |
| R | BC038167 |
| R | BC051019 |
| R | BC060267 |
| R | BC062650 |
| R | Bcas1 |
| R | Bphl |
| R | Bzrap1 |
| R | C730043O17 |
| R | Capsl |
| R | Cblc |
| R | Ccdc108 |
| R | Ccdc13 |
| R | Ccdc40 |
| R | Ccdc67 |
| R | Cd24a |
| R | Cdc14a |
| R | Celsr1 |
| R | Cldn7 |
| R | Col8a2 |
| R | Crip3 |
| R | Ctxn1 |
| R | Cyp2s1 |
| R | D10Bwg1070e |
| R | D130043K22Rik |
| R | D19Ertd652e |
| R | D230014K01Rik |
| R | D430042O09Rik |
| R | D630040G17Rik |
| R | Dalrd3 |
| R | Dalrd3 |
| R | Dbndd1 |
| R | Dmrt2 |
| R | Dnahc11 |
| R | Dnahc2 |
| R | Dnahc6 |
| R | Dnahc9 |
| R | Dnaja4 |
| R | Dnajc12 |
| R | Dusp14 |
| R | Dync2h1 |
| R | Dync2h1 |
| R | Dync2h1 |
| R | Dynlrb2 |
| R | Dynlrb2 |
| R | Dyrk3 |
| R | Dyx1c1 |
| R | Dzip1 |
| R | E230008N13Rik |
| R | Efcab1 |
| R | Efhb |
| R | ENSMUST00000035915 |
| R | ENSMUST00000036576 |
| R | ENSMUST00000050829 |
| R | Eppb9 |
| R | Faah |
| R | Fbxo36 |
| R | Fhad1 |
| R | Foxj1 |
| R | Fsip1 |
| R | Galnt3 |
| R | Ggt6 |
| R | Gm1060 |
| R | Gm166 |
| R | Gm969 |
| R | Golph2 |
| R | Gpx2 |
| R | Hist1h2ba |
| R | Hmgn3 |
| R | Hspa4l |
| R | Hspa4l |
| R | Hspa4l |
| R | Ift140 |
| R | Ift81 |
| R | Ift81 |
| R | Ift88 |
| R | Iqca |
| R | Iqcg |
| R | Klhdc7a |
| R | Kndc1 |
| R | Kremen1 |
| R | Liph |
| R | Lnx1 |
| R | Lnx1 |
| R | LOC546840 |
| R | Lrig1 |
| R | Lrrc23 |
| R | Lrrc51 |
| R | Lrrc51 |
| R | Lrrc56 |
| R | Mak |
| R | Mansc1 |
| R | Mapk15 |
| R | Mdh1b |
| R | Mgat3 |
| R | Mlf1 |
| R | Morn3 |
| R | Mtap7 |
| R | Muc4 |
| R | Muc4 |
| R | Muc5ac |
| R | Myb |
| R | NAP021083-001 |
| R | NAP029500-1 |
| R | Neurl |
| R | Ngef |
| R | Nme5 |
| R | Nme5 |
| R | Oit1 |
| R | Osbpl10 |
| R | Ovol2 |
| R | Pcp4l1 |
| R | Pcsk6 |
| R | Pex11c |
| R | Pih1d2 |
| R | Plcb3 |
| R | Plch2 |
| R | Plekhg6 |
| R | Ptpn13 |
| R | Rabl2a |
| R | Rage |
| R | Rage |
| R | Rarb |
| R | Rassf6 |
| R | Retnla |
| R | Ribc1 |
| R | Ribc2 |
| R | Rnf186 |
| R | Rnf32 |
| R | Rnf32 |
| R | Ropn1l |
| R | Rpgr |
| R | Serpina9 |
| R | Six1 |
| R | Slc39a4 |
| R | Spa17 |
| R | Spag16 |
| R | Spag17 |
| R | Spef1 |
| R | Spint1 |
| R | Spint1 |
| R | Spnb3 |
| R | Stk36 |
| R | Strbp |
| R | Strbp |
| R | TC1610785 |
| R | TC1631866 |
| R | TC1671899 |
| R | Tcea3 |
| R | Tcf2 |
| R | Tekt1 |
| R | Tekt2 |
| R | Tekt4 |
| R | Tjp3 |
| R | Tmc5 |
| R | Tmem107 |
| R | Tmem54 |
| R | Traf3ip1 |
| R | Traf3ip1 |
| R | Trp73 |
| R | Tspan33 |
| R | Ttc18 |
| R | Ttc18 |
| R | Ubxd5 |
| R | Vpreb3 |
| R | Wdr35 |
| R | Wdr35 |
| R | Wdr52 |
| R | Wdr54 |
| R | Wdr60 |
| R | Wdr63 |
| R | Wdr78 |
| R | Wdr90 |
| R | Yipf2 |
| R | Yipf2 |
| R | Yipf2 |
| R | Zfp474 |
| R | Zfp750 |
| R | Zmynd10 |
| R | Zmynd12 |
| S | 11-Sep |
| S | 1110054M08Rik |
| S | 1500041B16Rik |
| S | 1700012H05Rik |
| S | 1810021J13Rik |
| S | 1810026B05Rik |
| S | 2010003O02Rik |
| S | 2310040A07Rik |
| S | 2610034N15Rik |
| S | 2810432L12Rik |
| S | 2900024O10Rik |
| S | 2900024O10Rik |
| S | 3110001I20Rik |
| S | 3110001I20Rik |
| S | 4732457N14 |
| S | 4732479N06Rik |
| S | 4921511K06Rik |
| S | 5730601F06Rik |
| S | 6720401G13Rik |
| S | 6720458D17Rik |
| S | 8430408J07Rik |
| S | 9430028L06Rik |
| S | 9530004P13Rik |
| S | 9630050M13Rik |
| S | 9830163H01Rik |
| S | 9930024M15Rik |
| S | A430090L17Rik |
| S | A930001N09Rik |
| S | Abcb9 |
| S | Abcc5 |
| S | Abcd3 |
| S | Ablim2 |
| S | Acrbp |
| S | Acsl4 |
| S | Adarb1 |
| S | Adarb1 |
| S | Aes |
| S | AK031532 |
| S | AK036787 |
| S | AK038076 |
| S | AK041221 |
| S | AK041851 |
| S | AK042559 |
| S | AK048751 |
| S | AK049403 |
| S | AK078885 |
| S | AK079732 |
| S | AK080164 |
| S | AK142427 |
| S | Alas1 |
| S | Alcam |
| S | Als2cr13 |
| S | Angptl2 |
| S | Ap1m2 |
| S | Armc9 |
| S | Arrdc3 |
| S | Atad4 |
| S | Atp1b1 |
| S | Atp1b2 |
| S | Atpif1 |
| S | Atxn1 |
| S | AU021092 |
| S | AW124722 |
| S | Bace1 |
| S | BB128963 |
| S | BC021608 |
| S | BC037121 |
| S | BC038156 |
| S | BC038613 |
| S | BC051142 |
| S | Bcam |
| S | Bdnf |
| S | Bmpr2 |
| S | Bmpr2 |
| S | Btbd6 |
| S | C1qtnf7 |
| S | Casd1 |
| S | Casz1 |
| S | Ccpg1 |
| S | Cd300lg |
| S | Cds1 |
| S | Cfh |
| S | Cfh |
| S | Cfh |
| S | Cfh |
| S | Cgnl1 |
| S | Chst8 |
| S | Cldn3 |
| S | Clec14a |
| S | Clec14a |
| S | Clic5 |
| S | Clstn2 |
| S | Cobll1 |
| S | Col8a1 |
| S | Crebl2 |
| S | Creg1 |
| S | Creg1 |
| S | Crim1 |
| S | Crim1 |
| S | Ctf1 |
| S | Cttnbp2 |
| S | Cxx1b |
| S | Cyhr1 |
| S | Cyp2d22 |
| S | Cys1 |
| S | D030011O10Rik |
| S | D130061D10Rik |
| S | D14Ertd436e |
| S | D1Ertd161e |
| S | D730040F13Rik |
| S | Dag1 |
| S | Dag1 |
| S | Dennd2a |
| S | Dennd2d |
| S | Dmd |
| S | Dnajb4 |
| S | Dnase2a |
| S | Dnase2a |
| S | Dpyd |
| S | Dstn |
| S | Dusp14 |
| S | Dynll2 |
| S | Dynll2 |
| S | E130319B15Rik |
| S | Eif2c1 |
| S | Eif2c4 |
| S | Eif4ebp2 |
| S | ENSMUST00000050697 |
| S | ENSMUST00000069187 |
| S | Epn2 |
| S | Eps8l2 |
| S | Erbb3 |
| S | Etl4 |
| S | Etv5 |
| S | Evc2 |
| S | Eya1 |
| S | F730031O20Rik |
| S | Fbxo10 |
| S | Fbxw8 |
| S | Foxp1 |
| S | Fyco1 |
| S | Gata3 |
| S | Gcat |
| S | Gfra2 |
| S | Glcci1 |
| S | Glcci1 |
| S | Glis2 |
| S | Glt28d2 |
| S | Gm684 |
| S | Gng11 |
| S | Gramd1c |
| S | Grhl2 |
| S | Grin2c |
| S | Gstt2 |
| S | Hdac7a |
| S | Hdac8 |
| S | Helz |
| S | Hfe |
| S | Hgf |
| S | Hisppd2a |
| S | Igf1r |
| S | Igfbp2 |
| S | Ildr1 |
| S | Inpp4b |
| S | Irs1 |
| S | Irx1 |
| S | Irx1 |
| S | Itga1 |
| S | Itsn1 |
| S | Jmy |
| S | Kcna2 |
| S | Kdr |
| S | Klhl26 |
| S | Krtcap3 |
| S | Krtcap3 |
| S | Lama4 |
| S | Lamb2 |
| S | Lamb3 |
| S | Lifr |
| S | Ly6g6d |
| S | Magi1 |
| S | Maml2 |
| S | Maml2 |
| S | Mcf2l |
| S | Mgll |
| S | Mgll |
| S | Mgst1 |
| S | Mgst1 |
| S | Mllt3 |
| S | Mllt3 |
| S | Mme |
| S | Mpped2 |
| S | Mtap7 |
| S | Mthfd1 |
| S | Mtss1 |
| S | Mxi1 |
| S | Myo1b |
| S | Myo1b |
| S | Myo1b |
| S | NAP014312-001 |
| S | Nbeal1 |
| S | Nbl1 |
| S | Ndst1 |
| S | Nebl |
| S | Neo1 |
| S | Nfia |
| S | Nfib |
| S | Ngfrap1 |
| S | Nphp3 |
| S | Npr3 |
| S | Npr3 |
| S | Nuak1 |
| S | Pard3b |
| S | Park2 |
| S | Pcdh12 |
| S | Pcnt |
| S | Pcnt |
| S | Pcx |
| S | Pde7a |
| S | Pea15a |
| S | Pfkfb4 |
| S | Pgm5 |
| S | Pir |
| S | Pkd1 |
| S | Pla2g4f |
| S | Plagl1 |
| S | Plcb1 |
| S | Plxdc2 |
| S | Por |
| S | Ppp1r12b |
| S | Ppp3ca |
| S | Ptch1 |
| S | Ptgs1 |
| S | Ptpn3 |
| S | Pxmp2 |
| S | Pxmp4 |
| S | Rab25 |
| S | Rab6b |
| S | Rabgap1l |
| S | Rad51l3 |
| S | Rasgef1a |
| S | Rassf7 |
| S | Rbm35a |
| S | Rbms3 |
| S | Rbms3 |
| S | Reps2 |
| S | Rhebl1 |
| S | Ripk4 |
| S | Ripk5 |
| S | Rmnd5a |
| S | Rnf144 |
| S | Robo2 |
| S | Rora |
| S | RP23-136K12.4 |
| S | RP23-157O10.7 |
| S | Rpl22 |
| S | Rprm |
| S | Rprm |
| S | Rragd |
| S | Rtn1 |
| S | Rtn1 |
| S | Rusc2 |
| S | Rutbc1 |
| S | Rutbc3 |
| S | Scarf2 |
| S | Scn7a |
| S | Scrn1 |
| S | Sdha |
| S | Selenbp1 |
| S | Sema3e |
| S | Sema6a |
| S | Sesn1 |
| S | Setbp1 |
| S | Sh3d19 |
| S | Sigirr |
| S | Siglecf |
| S | Sil1 |
| S | Sirt5 |
| S | Slc12a2 |
| S | Slc16a2 |
| S | Slc1a4 |
| S | Slc25a36 |
| S | Slc25a36 |
| S | Slc30a9 |
| S | Slc9a3r2 |
| S | Slitrk6 |
| S | Smarca2 |
| S | Sntb1 |
| S | Sord |
| S | Sox13 |
| S | Sp5 |
| S | Specc1l |
| S | Spg20 |
| S | Spg3a |
| S | Spnb2 |
| S | Sstr4 |
| S | Stard9 |
| S | Sytl4 |
| S | Tacc2 |
| S | TC1632790 |
| S | TC1672661 |
| S | Tcf3 |
| S | Tfdp2 |
| S | Tgoln1 |
| S | Thsd4 |
| S | Timp2 |
| S | Tlcd1 |
| S | Tln2 |
| S | Tmc4 |
| S | Tmc4 |
| S | Tmcc3 |
| S | Tmeff2 |
| S | Tmem117 |
| S | Tmem63b |
| S | Tmem64 |
| S | Tmem64 |
| S | Tom1l1 |
| S | Trim3 |
| S | Trim3 |
| S | Trp53bp2 |
| S | Tsc22d1 |
| S | Tspan15 |
| S | Txnrd3 |
| S | Usp20 |
| S | Usp33 |
| S | Utrn |
| S | Vegfa |
| S | Vsig2 |
| S | Wnk4 |
| S | Wwp1 |
| S | Ypel1 |
| S | Zbtb20 |
| S | Zbtb4 |
| S | Zfp354a |
| S | Zfyve21 |
| S | Zkscan14 |
| T | 1110001J03Rik |
| T | 1110012N22Rik |
| T | 1300010F03Rik |
| T | 1500032D16Rik |
| T | 2310076L09Rik |
| T | 2900062L11Rik |
| T | 9530058B02Rik |
| T | Abcb8 |
| T | Acad10 |
| T | Acad11 |
| T | Acads |
| T | Acadvl |
| T | Acat1 |
| T | Aco2 |
| T | Acox1 |
| T | Acsl1 |
| T | Acsl1 |
| T | Actc1 |
| T | Actn2 |
| T | Adprhl1 |
| T | Ahsg |
| T | AK161939 |
| T | AK199677 |
| T | Akap6 |
| T | Aldh5a1 |
| T | Ank |
| T | Ank1 |
| T | Ankrd15 |
| T | Ankrd23 |
| T | Ankrd23 |
| T | Aoc3 |
| T | Apobec2 |
| T | Art3 |
| T | Asb2 |
| T | Atp2a2 |
| T | Auh |
| T | AW822216 |
| T | B230317C12Rik |
| T | BI100969 |
| T | BY439412 |
| T | C030006K11Rik |
| T | Camta1 |
| T | Cdh13 |
| T | Chpt1 |
| T | Clybl |
| T | Cox7a1 |
| T | Cox8b |
| T | Csrp3 |
| T | Cyc1 |
| T | D10Jhu81e |
| T | D12Ertd647e |
| T | D930010J01Rik |
| T | Dci |
| T | Ddhd2 |
| T | Decr1 |
| T | Dgat2 |
| T | Dmpk |
| T | Doc2g |
| T | Dsp |
| T | Echdc3 |
| T | Eno3 |
| T | ENSMUSG00000071543 |
| T | Etfdh |
| T | Fabp3 |
| T | Fahd1 |
| T | Fahd2a |
| T | Fhl2 |
| T | Fhod3 |
| T | Frat1 |
| T | Frmd5 |
| T | Fxyd1 |
| T | Gmpr |
| T | Gnpat |
| T | Gpd1 |
| T | Gpd1 |
| T | Gpsm1 |
| T | Gstz1 |
| T | Hhatl |
| T | Htra3 |
| T | Idh2 |
| T | Idh3a |
| T | Itgb1bp2 |
| T | Ivd |
| T | Kcna1 |
| T | Kcnk3 |
| T | Kif1c |
| T | Lmod3 |
| T | Lpl |
| T | Lynx1 |
| T | Map1lc3a |
| T | Mapk10 |
| T | Mb |
| T | Mccc1 |
| T | Mccc1 |
| T | Mdh1 |
| T | Mdh1 |
| T | Mfn2 |
| T | Mlxipl |
| T | Mrvi1 |
| T | Myl4 |
| T | Myo18b |
| T | Myom2 |
| T | Myoz2 |
| T | NAP006594-001 |
| T | Ncam1 |
| T | Ncam1 |
| T | Ndufa10 |
| T | Ndufb10 |
| T | Ndufs2 |
| T | Nexn |
| T | Ociad2 |
| T | Ogdh |
| T | Ogdh |
| T | Oplah |
| T | Ormdl3 |
| T | Oxct1 |
| T | Pacsin3 |
| T | Pcca |
| T | Pde4dip |
| T | Pdha1 |
| T | Pdlim5 |
| T | Pfkm |
| T | Pgam2 |
| T | Ppargc1a |
| T | Ppargc1a |
| T | Ppargc1b |
| T | Ppargc1b |
| T | Ppargc1b |
| T | Prkag2 |
| T | Ptgfr |
| T | Ptn |
| T | Pygb |
| T | Pygm |
| T | Rbpms2 |
| T | Rhobtb1 |
| T | Rxrg |
| T | S100b |
| T | S3-12 |
| T | Sec31b |
| T | Sgcg |
| T | Slc22a1 |
| T | Slc25a19 |
| T | Slc25a3 |
| T | Slc25a4 |
| T | Slc25a4 |
| T | Slc27a1 |
| T | Slc4a3 |
| T | Slc4a3 |
| T | Sln |
| T | Smarcd3 |
| T | Sobp |
| T | Stard10 |
| T | Suclg1 |
| T | TC1646108 |
| T | Tcap |
| T | Tmem38a |
| T | Tnni3 |
| T | Tnnt2 |
| T | Tpm1 |
| T | Tpm1 |
| T | Ttn |
| T | Ttn |
| T | Ttn |
| T | Yipf7 |
| T | Zfp106 |
| U | 1110006O24Rik |
| U | 1110014J01Rik |
| U | 1200015N20Rik |
| U | 1700019G17Rik |
| U | 1700020C07Rik |
| U | 1700027J05Rik |
| U | 1700041E20Rik |
| U | 1700049J03Rik |
| U | 1700105P06Rik |
| U | 1810049H19Rik |
| U | 2310058D17Rik |
| U | 2410004P03Rik |
| U | 2610028A01Rik |
| U | 2810408B13Rik |
| U | 2900083I11Rik |
| U | 3110048L19Rik |
| U | 4921524J17Rik |
| U | 4921525H12Rik |
| U | 4921530G04Rik |
| U | 4930539N22Rik |
| U | 4930571C24Rik |
| U | 4932418E24Rik |
| U | 6330419J24Rik |
| U | 6720457D02Rik |
| U | 8430426H19Rik |
| U | 9030025P20Rik |
| U | 9430010O03Rik |
| U | A_51_P451508 |
| U | A_52_P1013432 |
| U | A030003K02Rik |
| U | A230042K10Rik |
| U | A230058J24Rik |
| U | A630055G03Rik |
| U | A630089N07Rik |
| U | AA987161 |
| U | Aak1 |
| U | Adra1d |
| U | Aff4 |
| U | Ahdc1 |
| U | AI118078 |
| U | AI987944 |
| U | AK006309 |
| U | AK006604 |
| U | AK034319 |
| U | AK035470 |
| U | AK043486 |
| U | AK050700 |
| U | AK052999 |
| U | AK082070 |
| U | AK082839 |
| U | AK084225 |
| U | AK085302 |
| U | Aldoa-ps1 |
| U | Ankrd11 |
| U | Apbb2 |
| U | Apc2 |
| U | Aqp2 |
| U | Aqp4 |
| U | Asgr1 |
| U | Atp7b |
| U | AU020772 |
| U | AW536275 |
| U | B230315N10Rik |
| U | B430319H21Rik |
| U | Bat2d |
| U | BC002059 |
| U | BC024139 |
| U | BC029127 |
| U | BC029127 |
| U | BC029127 |
| U | BC031441 |
| U | BC048507 |
| U | BC051212 |
| U | BC068233 |
| U | BG084650 |
| U | Bloc1s3 |
| U | Braf |
| U | Bzrpl1 |
| U | C1rl |
| U | C330007P06Rik |
| U | C330013J21Rik |
| U | C77080 |
| U | C87436 |
| U | Cacna2d2 |
| U | Calm3 |
| U | Camk1g |
| U | Card14 |
| U | Cask |
| U | Cbx8 |
| U | Ccdc131 |
| U | Cd3e |
| U | Cdk5r2 |
| U | Celsr3 |
| U | Chd6 |
| U | Chrnd |
| U | Cited1 |
| U | Cmtm1 |
| U | Cntnap1 |
| U | Coro2b |
| U | Cplx2 |
| U | Cpne5 |
| U | Crim2 |
| U | Csnk1a1 |
| U | Cyp17a1 |
| U | Cyp46a1 |
| U | D17H6S56E-3 |
| U | D330037H05Rik |
| U | D5Ertd135e |
| U | Daf2 |
| U | Ddx25 |
| U | Ddx42 |
| U | Dgcr8 |
| U | Dhrs7b |
| U | Dirc2 |
| U | Dis3l |
| U | Dlk1 |
| U | Dlx6as |
| U | Dnalc1 |
| U | Dot1l |
| U | Drd3 |
| U | Dyrk1b |
| U | Dyrk1b |
| U | E130119H09Rik |
| U | E130304I02Rik |
| U | E230015B07Rik |
| U | E2f4 |
| U | Ecel1 |
| U | EG240038 |
| U | EG382161 |
| U | EG434179 |
| U | EG434459 |
| U | EG435336 |
| U | EG435337 |
| U | EG435366 |
| U | EG435970 |
| U | EG546361 |
| U | EG627782 |
| U | EG629581 |
| U | EG629591 |
| U | EG630882 |
| U | EG631624 |
| U | EG664907 |
| U | EG666606 |
| U | ENSMUSG00000044227 |
| U | ENSMUSG00000060603 |
| U | ENSMUSG00000069586 |
| U | ENSMUSG00000070586 |
| U | ENSMUST00000040810 |
| U | ENSMUST00000074313 |
| U | ENSMUST00000075032 |
| U | ENSMUST00000099744 |
| U | Esrrg |
| U | Fbs1 |
| U | Fbxo2 |
| U | Fbxo2 |
| U | Fbxo21 |
| U | Fchsd1 |
| U | Fkbp11 |
| U | Foxh1 |
| U | Fthl17 |
| U | G630014P10Rik |
| U | Gabbr2 |
| U | Gal |
| U | Gdap1l1 |
| U | Glp2r |
| U | Gnai1 |
| U | Gpr173 |
| U | Gpr37l1 |
| U | Gpr44 |
| U | Gprc5d |
| U | Grin2d |
| U | Gscl |
| U | Gtrgeo22 |
| U | Hcn4 |
| U | Hes2 |
| U | Hhat |
| U | Hip1 |
| U | Hist1h1t |
| U | Ifrd2 |
| U | Ifrg15 |
| U | Ikzf3 |
| U | Ing5 |
| U | Inhbb |
| U | Inhbe |
| U | Iqcf1 |
| U | Irs4 |
| U | Jph4 |
| U | Kcnc4 |
| U | Kif26a |
| U | Klf8 |
| U | Klk4 |
| U | Krtap16-10 |
| U | Krtap6-3 |
| U | L3mbtl2 |
| U | Lalba |
| U | Lama3 |
| U | Lce1l |
| U | Lcn10 |
| U | Lhb |
| U | Lhfpl4 |
| U | Lhx4 |
| U | Lifr |
| U | Lmnb2 |
| U | Lmtk2 |
| U | LOC383707 |
| U | LOC619975 |
| U | LOC621290 |
| U | LOC632900 |
| U | LOC638058 |
| U | LOC670626 |
| U | LOC671973 |
| U | LOC675815 |
| U | Lrdd |
| U | Ly6g5b |
| U | Ly6g5c |
| U | Ly6g6c |
| U | Ly6g6d |
| U | M17518 |
| U | Mapk8ip2 |
| U | Mc4r |
| U | Mchr1 |
| U | Mdga2 |
| U | MGC117846 |
| U | Mier1 |
| U | Mitf |
| U | Mocs1 |
| U | Mov10 |
| U | Nab2 |
| U | NAP000001-064 |
| U | NAP005803-002 |
| U | NAP018688-001 |
| U | NAP020770-001 |
| U | NAP020861-001 |
| U | NAP022641-001 |
| U | NAP025478-001 |
| U | NAP026611-1 |
| U | NAP026710-1 |
| U | NAP027922-1 |
| U | NAP030950-1 |
| U | NAP039199-1 |
| U | NAP044463-1 |
| U | NAP045501-1 |
| U | NAP048481-1 |
| U | NAP059572-1 |
| U | NAP062835-1 |
| U | NAP067058-1 |
| U | NAP070792-1 |
| U | NAP070939-1 |
| U | NAP071006-1 |
| U | NAP071025-1 |
| U | NAP071064-1 |
| U | NAP096498-001 |
| U | NAP102079-1 |
| U | NAP102091-1 |
| U | NAP102441-1 |
| U | NAP102548-1 |
| U | NAP102683-1 |
| U | NAP103757-1 |
| U | NAP108144-1 |
| U | NAP113018-1 |
| U | NAP113722-1 |
| U | NAP115505-1 |
| U | NAP121938-001 |
| U | NAP123498-1 |
| U | Nap1l4 |
| U | Nfatc2 |
| U | Nfe2l3 |
| U | Ngb |
| U | Npal3 |
| U | Nr1d1 |
| U | Nr1i3 |
| U | Nr3c2 |
| U | Odf4 |
| U | Og9x |
| U | Olfr1030 |
| U | Olfr1170 |
| U | Olfr124 |
| U | Olfr1263 |
| U | Olfr1344 |
| U | Olfr1350 |
| U | Olfr1393 |
| U | Olfr1444 |
| U | Olfr1459 |
| U | Olfr313 |
| U | Olfr32 |
| U | Olfr414 |
| U | Olfr469 |
| U | Olfr48 |
| U | Olfr53 |
| U | Olfr744 |
| U | Olfr933 |
| U | Osbpl7 |
| U | Otos |
| U | OTTMUSG00000005065 |
| U | OTTMUSG00000008584 |
| U | OTTMUSG00000015730 |
| U | OTTMUSG00000015743 |
| U | OTTMUSG00000015743 |
| U | Palld |
| U | Pcbd2 |
| U | Pcbp1 |
| U | Pdcl3 |
| U | Pdpk1 |
| U | Pknox2 |
| U | Plcg1 |
| U | Plcl1 |
| U | Plec1 |
| U | Pou4f1 |
| U | Ppp1r13l |
| U | Ppp1r1b |
| U | Ppp3r1 |
| U | Ppp3r2 |
| U | Ppp5c |
| U | Prdm2 |
| U | Prkcc |
| U | Prlr |
| U | Prp2 |
| U | Prrt1 |
| U | Prss3 |
| U | Psd |
| U | Ptk7 |
| U | Pyy |
| U | Raver2 |
| U | Rb1cc1 |
| U | Rbak |
| U | Rdhs |
| U | Rec8L1 |
| U | Rex2 |
| U | Rhbdl3 |
| U | RP23-406N5.2 |
| U | Rps21 |
| U | Rps21 |
| U | Rusc1 |
| U | Samd10 |
| U | Sars2 |
| U | Scn10a |
| U | Scrt2 |
| U | Sdk2 |
| U | Sema5b |
| U | Serpinb6c |
| U | Set |
| U | Shc2 |
| U | Slc17a2 |
| U | Slc1a2 |
| U | Slc20a1 |
| U | Slc22a12 |
| U | Slc22a17 |
| U | Slc22a8 |
| U | Slit1 |
| U | Slmo1 |
| U | Sox15 |
| U | Speg |
| U | Spint2 |
| U | Sprn |
| U | Sprr2g |
| U | Ssb |
| U | Stab2 |
| U | Stox2 |
| U | Stra6 |
| U | Strbp |
| U | Syn1 |
| U | Synpo |
| U | Taar4 |
| U | Tacr1 |
| U | Tbrg4 |
| U | TC1625496 |
| U | TC1631533 |
| U | TC1637123 |
| U | TC1661973 |
| U | TC1665684 |
| U | TC1666853 |
| U | TC1672155 |
| U | TC1679528 |
| U | TC1686545 |
| U | TC1707343 |
| U | TC1708273 |
| U | TC1721634 |
| U | Tdh |
| U | Tex21 |
| U | Tgm5 |
| U | Tle3 |
| U | Tlx2 |
| U | Tmco7 |
| U | Tmem165 |
| U | Tmem16h |
| U | Tmem174 |
| U | Tnk2 |
| U | Trim45 |
| U | Ttbk1 |
| U | Tulp1 |
| U | Ubxd2 |
| U | Usp29 |
| U | Uts2r |
| U | V1ra4 |
| U | V1ri6 |
| U | Vav2 |
| U | Wasf1 |
| U | Wdr25 |
| U | Ybx2 |
| U | Zfp179 |
| U | Zfp248 |
| U | Zfp273 |
| U | Zfp335 |
| U | Zfp51 |
| U | Zfp74 |
| U | Zfp759 |
| V | 7-Mar |
| V | 9-Sep |
| V | 10-Sep |
| V | 0610007P08Rik |
| V | 0610030E20Rik |
| V | 1110033M05Rik |
| V | 1110037F02Rik |
| V | 1110059G10Rik |
| V | 1500002O20Rik |
| V | 1600016N20Rik |
| V | 1700001J11Rik |
| V | 1700003O08Rik |
| V | 1700019N12Rik |
| V | 1700020I14Rik |
| V | 1700030E15Rik |
| V | 1700081L11Rik |
| V | 1700081L11Rik |
| V | 1700124K17Rik |
| V | 2010106G01Rik |
| V | 2210408I21Rik |
| V | 2210412D01Rik |
| V | 2210417D09Rik |
| V | 2310003C23Rik |
| V | 2310047C04Rik |
| V | 2410042D21Rik |
| V | 2610020O08Rik |
| V | 2610024E20Rik |
| V | 2610101N10Rik |
| V | 2610203E10Rik |
| V | 2610207I05Rik |
| V | 2610207I05Rik |
| V | 2610208M17Rik |
| V | 2610507B11Rik |
| V | 2610507B11Rik |
| V | 2810002O09Rik |
| V | 2810006K23Rik |
| V | 2810055G22Rik |
| V | 2900053A13Rik |
| V | 3110001A13Rik |
| V | 3110031B13Rik |
| V | 4121402D02Rik |
| V | 4632433K11Rik |
| V | 4921505C17Rik |
| V | 4930402H24Rik |
| V | 4930422G04Rik |
| V | 4930422G04Rik |
| V | 4930431B09Rik |
| V | 4930432O21Rik |
| V | 4930506M07Rik |
| V | 4930535B03Rik |
| V | 4930546H06Rik |
| V | 4932438A13Rik |
| V | 4933426M11Rik |
| V | 4933440H19Rik |
| V | 5031439G07Rik |
| V | 5530601H04Rik |
| V | 5730596K20Rik |
| V | 5830415L20Rik |
| V | 5830482F20Rik |
| V | 6030465E24Rik |
| V | 6330411E07Rik |
| V | 6720456H09Rik |
| V | 9030409G11Rik |
| V | 9030416H16Rik |
| V | 9030425P06Rik |
| V | 9130023H24Rik |
| V | 9430025N12Rik |
| V | 9430025N12Rik |
| V | 9530028C05 |
| V | 9830124H08Rik |
| V | A_51_P462771 |
| V | A_51_P502068 |
| V | A_52_P771912 |
| V | A_52_P860487 |
| V | A230046K03Rik |
| V | A430041B07Rik |
| V | A430041B07Rik |
| V | A430057L12Rik |
| V | A530054K11Rik |
| V | A530054K11Rik |
| V | A530082C11Rik |
| V | A630018P17Rik |
| V | A630026N12Rik |
| V | A630047E20Rik |
| V | A630082K20Rik |
| V | A830080L01Rik |
| V | A930001N09Rik |
| V | AA409316 |
| V | AA536743 |
| V | Abcc1 |
| V | Abcc3 |
| V | Abce1 |
| V | Abl2 |
| V | Abl2 |
| V | Ablim1 |
| V | Abt1 |
| V | Acaca |
| V | Acaca |
| V | Acbd3 |
| V | Acbd3 |
| V | Acin1 |
| V | Acly |
| V | Actb |
| V | Adam17 |
| V | Adamts1 |
| V | Adcy6 |
| V | AF315352 |
| V | Aff1 |
| V | Agpat6 |
| V | Ahnak |
| V | Ahnak |
| V | AI450540 |
| V | AI504432 |
| V | AI848100 |
| V | AJ237917 |
| V | AK008871 |
| V | AK013239 |
| V | AK021069 |
| V | AK032729 |
| V | AK032810 |
| V | AK035139 |
| V | AK036484 |
| V | AK036874 |
| V | AK037061 |
| V | AK037107 |
| V | AK038845 |
| V | AK040092 |
| V | AK040351 |
| V | AK040891 |
| V | AK041471 |
| V | AK041476 |
| V | AK041753 |
| V | AK041988 |
| V | AK042078 |
| V | AK042230 |
| V | AK044019 |
| V | AK044844 |
| V | AK045158 |
| V | AK045163 |
| V | AK045692 |
| V | AK046873 |
| V | AK047822 |
| V | AK049591 |
| V | AK050569 |
| V | AK050842 |
| V | AK051007 |
| V | AK051319 |
| V | AK051522 |
| V | AK054451 |
| V | AK080115 |
| V | AK081910 |
| V | AK082068 |
| V | AK082409 |
| V | AK082505 |
| V | AK084172 |
| V | AK085328 |
| V | AK085547 |
| V | AK087406 |
| V | AK087421 |
| V | AK087625 |
| V | AK087943 |
| V | AK088646 |
| V | AK169992 |
| V | Akap10 |
| V | Akap13 |
| V | Akap13 |
| V | Akt1 |
| V | Als2cr13 |
| V | Als2cr13 |
| V | Amdhd2 |
| V | Ankib1 |
| V | Ankrd15 |
| V | Anp32e |
| V | Anxa4 |
| V | Ap4e1 |
| V | Apbb1ip |
| V | Apc |
| V | Apex2 |
| V | App |
| V | Arfgef1 |
| V | Arhgap10 |
| V | Arhgap12 |
| V | Arhgap26 |
| V | Arhgap29 |
| V | Arhgef10 |
| V | Arhgef12 |
| V | Arhgef6 |
| V | Arhgef6 |
| V | Arid3a |
| V | Arl13b |
| V | Arl4c |
| V | Arl5b |
| V | Arl6ip2 |
| V | Arl6ip6 |
| V | Atad2b |
| V | Atad2b |
| V | Atbf1 |
| V | Atf6 |
| V | Atf7 |
| V | Atf7ip |
| V | Atg12 |
| V | Atp11b |
| V | Atp13a3 |
| V | Atp2b1 |
| V | Atp2b1 |
| V | Atp6v0a1 |
| V | Atp8b1 |
| V | Atp8b2 |
| V | Atp8b4 |
| V | Atrn |
| V | Atrn |
| V | Atrx |
| V | Atxn1 |
| V | Atxn7 |
| V | Atxn7l3 |
| V | AU017455 |
| V | AU040320 |
| V | AW555464 |
| V | AY036118 |
| V | Azin1 |
| V | B230219D22Rik |
| V | B230325K18Rik |
| V | B230369F24Rik |
| V | B2m |
| V | B3galt4 |
| V | B930006L02Rik |
| V | Baz1b |
| V | Baz2a |
| V | Bbx |
| V | Bbx |
| V | BC005537 |
| V | BC005537 |
| V | BC013672 |
| V | BC018371 |
| V | BC024659 |
| V | BC037034 |
| V | BC038700 |
| V | BC040101 |
| V | BC071254 |
| V | BC082312 |
| V | Bcl2l1 |
| V | Bcl2l1 |
| V | Bcl7c |
| V | Bclaf1 |
| V | Bcr |
| V | BF228116 |
| V | BG296545 |
| V | Bhlhb8 |
| V | BI738048 |
| V | Blzf1 |
| V | Blzf1 |
| V | Braf |
| V | Brd3 |
| V | Brd4 |
| V | Brp16 |
| V | Brwd1 |
| V | Btg2 |
| V | Btg2 |
| V | Bzw1 |
| V | C030010B13Rik |
| V | C030046E11Rik |
| V | C030048B08Rik |
| V | C030048B08Rik |
| V | C130022K22Rik |
| V | C130039O16Rik |
| V | C130057D09Rik |
| V | C1galt1 |
| V | C230029D21Rik |
| V | C230081A13Rik |
| V | C430003P19Rik |
| V | C730024G19Rik |
| V | C80913 |
| V | C920006C10Rik |
| V | C920006C10Rik |
| V | Cabin1 |
| V | Cacna1d |
| V | Cald1 |
| V | Calu |
| V | Camk2d |
| V | Camk2d |
| V | Canx |
| V | Capn5 |
| V | Cast |
| V | Ccdc100 |
| V | Ccdc117 |
| V | Ccdc25 |
| V | Ccdc55 |
| V | Ccdc66 |
| V | Ccdc85a |
| V | Ccdc85a |
| V | Ccng1 |
| V | Ccnk |
| V | Ccnl1 |
| V | Cd164 |
| V | Cd2ap |
| V | Cd44 |
| V | Cd47 |
| V | Cd93 |
| V | Cd99l2 |
| V | Cdc14a |
| V | Cdc27 |
| V | Cdc2l1 |
| V | Cdc2l5 |
| V | Cdc37l1 |
| V | Cdc42bpa |
| V | Cdk5rap2 |
| V | Cdk8 |
| V | Cdkl3 |
| V | Cebpg |
| V | Cebpz |
| V | Centb2 |
| V | Centb2 |
| V | Centg3 |
| V | Cep192 |
| V | Cep250 |
| V | Cflar |
| V | Cfp |
| V | Cgn |
| V | Chd1 |
| V | Chd7 |
| V | Chd9 |
| V | Ches1 |
| V | Chic1 |
| V | Chit1 |
| V | Chm |
| V | Chmp4b |
| V | Chmp4c |
| V | Chordc1 |
| V | Clasp1 |
| V | Clasp1 |
| V | Cldnd1 |
| V | Clic4 |
| V | Clip1 |
| V | Clip4 |
| V | Cnot3 |
| V | Cobll1 |
| V | Col1a1 |
| V | Col5a1 |
| V | Col7a1 |
| V | Cpeb3 |
| V | Cpeb4 |
| V | Creb5 |
| V | Crebzf |
| V | Crkrs |
| V | Crkrs |
| V | Csde1 |
| V | Csnk2a1 |
| V | Cugbp1 |
| V | Cwf19l1 |
| V | Cxxc5 |
| V | Cyld |
| V | D030011O10Rik |
| V | D030022P06Rik |
| V | D030074E01Rik |
| V | D12Ertd551e |
| V | D14Abb1e |
| V | D14Ertd436e |
| V | D17Wsu92e |
| V | D1Pas1 |
| V | D330037H05Rik |
| V | D4Wsu114e |
| V | D5Ertd579e |
| V | D5Wsu178e |
| V | Daam1 |
| V | Dars |
| V | Dcun1d3 |
| V | Dcun1d4 |
| V | Ddhd1 |
| V | Ddx1 |
| V | Ddx10 |
| V | Ddx19b |
| V | Ddx19b |
| V | Ddx21 |
| V | Ddx23 |
| V | Ddx24 |
| V | Ddx3x |
| V | Ddx46 |
| V | Ddx58 |
| V | Ddx6 |
| V | Dek |
| V | Dgke |
| V | Dis3 |
| V | Disc1 |
| V | Dlg1 |
| V | Dnajc10 |
| V | Dnajc10 |
| V | Dnajc2 |
| V | Dnajc2 |
| V | Dnajc5 |
| V | Dnm2 |
| V | Dnmt3a |
| V | Dock4 |
| V | Dph3 |
| V | Dpy19l1 |
| V | Dpysl2 |
| V | Drctnnb1a |
| V | Dsp |
| V | Dst |
| V | Dtx4 |
| V | Dusp16 |
| V | Dusp3 |
| V | Dym |
| V | Dync2h1 |
| V | Dzip1l |
| V | E030024N20Rik |
| V | E130014J05Rik |
| V | E130102H24Rik |
| V | E130112N10Rik |
| V | E2f3 |
| V | E330009E22Rik |
| V | E430025E21Rik |
| V | Eea1 |
| V | Eea1 |
| V | Eea1 |
| V | Efna5 |
| V | EG245436 |
| V | EG272350 |
| V | EG382421 |
| V | EG385454 |
| V | Eif2ak1 |
| V | Eif3s1 |
| V | Eif3s6 |
| V | Eif3s8 |
| V | Eif3s8 |
| V | Eif4a2 |
| V | Eif4b |
| V | Eif4e3 |
| V | Eif4enif1 |
| V | Eif4g1 |
| V | Eif4g2 |
| V | Eif5b |
| V | Ell |
| V | Elmo1 |
| V | Elmod2 |
| V | Emb |
| V | Enpp4 |
| V | Ensa |
| V | ENSMUSG00000054178 |
| V | ENSMUST00000015595 |
| V | ENSMUST00000024982 |
| V | ENSMUST00000055843 |
| V | ENSMUST00000078779 |
| V | ENSMUST00000079391 |
| V | ENSMUST00000088251 |
| V | ENSMUST00000095565 |
| V | ENSMUST00000096411 |
| V | ENSMUST00000098637 |
| V | ENSMUST00000103201 |
| V | Ep300 |
| V | Ep400 |
| V | Epc1 |
| V | Epm2aip1 |
| V | Epn1 |
| V | Eps15 |
| V | Erbb2ip |
| V | Erbb3 |
| V | Erbb3 |
| V | Erg |
| V | Esf1 |
| V | Etnk1 |
| V | Etnk1 |
| V | Exoc3 |
| V | Exoc6b |
| V | Fat1 |
| V | Fblim1 |
| V | Fbxl5 |
| V | Fcho2 |
| V | Fcmd |
| V | Fgd3 |
| V | Fgd4 |
| V | Fgf2 |
| V | Fhl3 |
| V | Fkbp15 |
| V | Fkbp5 |
| V | Flnb |
| V | Flnb |
| V | Flywch1 |
| V | Fnbp1l |
| V | Fndc3a |
| V | Fnip1 |
| V | Foxg1 |
| V | Foxj2 |
| V | Frmd4b |
| V | Frmd6 |
| V | Frmd6 |
| V | Fry |
| V | Fryl |
| V | Furin |
| V | Fus |
| V | Fyco1 |
| V | Fzd4 |
| V | Fzd5 |
| V | Gabbr1 |
| V | Gabpb1 |
| V | Galnt1 |
| V | Galnt10 |
| V | Gan |
| V | Ganab |
| V | Gas7 |
| V | Gata4 |
| V | Gbf1 |
| V | Gcc2 |
| V | Gcnt2 |
| V | Gimap4 |
| V | Git1 |
| V | Glis2 |
| V | Gls |
| V | Gltscr2 |
| V | Gmps |
| V | Gnao1 |
| V | Gnas |
| V | Gnptab |
| V | Golgb1 |
| V | Golph4 |
| V | Gosr1 |
| V | Gpatch2 |
| V | Gpbp1 |
| V | Gpbp1l1 |
| V | Gpr119 |
| V | Gpr177 |
| V | Gpr89 |
| V | Gprk6 |
| V | Gramd1b |
| V | Gripap1 |
| V | Gtf2h1 |
| V | Gtpbp4 |
| V | Gyk |
| V | Gzf1 |
| V | H2afy |
| V | H2afz |
| V | Hand2 |
| V | Hbs1l |
| V | Heatr1 |
| V | Heatr5a |
| V | Hel308 |
| V | Herc1 |
| V | Herc4 |
| V | Hipk1 |
| V | Hira |
| V | Hist1h1e |
| V | Hivep3 |
| V | Hk1 |
| V | Hk1 |
| V | Hlx1 |
| V | Hmbox1 |
| V | Hmg20b |
| V | Hmgb1-rs17 |
| V | Hnrpdl |
| V | Hnrpul1 |
| V | Hnrpul2 |
| V | Hook3 |
| V | Hs1bp3 |
| V | Hspa12a |
| V | Hspa4 |
| V | Hspa9 |
| V | Htatsf1 |
| V | Huwe1 |
| V | Huwe1 |
| V | Ifnz |
| V | Igf1r |
| V | Igf2bp2 |
| V | Igfbp4 |
| V | Igfbp5 |
| V | Il1rl2 |
| V | Il28ra |
| V | Ilf3 |
| V | Ing4 |
| V | Inoc1 |
| V | Inpp4a |
| V | Inpp5f |
| V | Ints6 |
| V | Irx2 |
| V | Itga3 |
| V | Itga3 |
| V | Itgb1 |
| V | Itgb7 |
| V | Ivns1abp |
| V | Jak2 |
| V | Jarid1b |
| V | Jarid2 |
| V | Jarid2 |
| V | Jmjd1a |
| V | Kdelc1 |
| V | Keap1 |
| V | Khdrbs1 |
| V | Kif13a |
| V | Kif13b |
| V | Kif16b |
| V | Kif1b |
| V | Kif1b |
| V | Kif1b |
| V | Kif2a |
| V | Kitl |
| V | Klf3 |
| V | Klf6 |
| V | Klhl18 |
| V | Klhl9 |
| V | Kpna4 |
| V | Kras |
| V | Kremen1 |
| V | Lair1 |
| V | Lamp2 |
| V | Larp1 |
| V | Larp5 |
| V | Lars |
| V | Las1l |
| V | Lass4 |
| V | Lass6 |
| V | Lats1 |
| V | Ldhal6b |
| V | Lemd3 |
| V | Leng8 |
| V | Lgtn |
| V | Lims1 |
| V | Lincr |
| V | Lmbrd2 |
| V | Lmnb1 |
| V | Lmo7 |
| V | Lmo7 |
| V | LOC236598 |
| V | Lonp2 |
| V | Lpp |
| V | Lrba |
| V | Lrfn4 |
| V | Lrig3 |
| V | Lrrc29 |
| V | Lrrc8b |
| V | Lrrk1 |
| V | Lypla1 |
| V | Maf |
| V | Magi2 |
| V | Maml2 |
| V | Man2a2 |
| V | Map4k3 |
| V | Mapk6 |
| V | Mapk9 |
| V | Mark3 |
| V | Mbd6 |
| V | Mbnl1 |
| V | Mbtps1 |
| V | Mdm4 |
| V | Mdn1 |
| V | Mef2c |
| V | Megf9 |
| V | Memo1 |
| V | Mgea6 |
| V | Micall1 |
| V | Mier1 |
| V | Mier1 |
| V | Mkl2 |
| V | Mkln1 |
| V | Mll2 |
| V | Mll3 |
| V | Mll3 |
| V | Mllt10 |
| V | Mllt3 |
| V | Mllt6 |
| V | Mnt |
| V | Mobkl1a |
| V | Morf4l1 |
| V | Mpeg1 |
| V | Mpo |
| V | Mpp1 |
| V | Mpp6 |
| V | Msn |
| V | Msrb3 |
| V | Mtap4 |
| V | Mtpn |
| V | Mtpn |
| V | Mtr |
| V | Mtus1 |
| V | Myadm |
| V | Myo10 |
| V | Myo9a |
| V | Myo9b |
| V | NAP000727-001 |
| V | NAP025806-1 |
| V | NAP028427-1 |
| V | NAP042178-1 |
| V | NAP042348-1 |
| V | NAP045236-1 |
| V | NAP052136-1 |
| V | NAP054603-1 |
| V | NAP060490-1 |
| V | NAP061200-1 |
| V | NAP068884-1 |
| V | NAP099819-001 |
| V | NAP102845-1 |
| V | NAP103155-1 |
| V | NAP107478-1 |
| V | NAP112463-1 |
| V | Ncoa6 |
| V | Nedd1 |
| V | Nedd9 |
| V | Nek1 |
| V | Nf1 |
| V | Nfat5 |
| V | Nfatc1 |
| V | Nfatc3 |
| V | Nfic |
| V | Nfix |
| V | Nfkbil1 |
| V | Nfxl1 |
| V | Nfyb |
| V | Nipa2 |
| V | Nlrp3 |
| V | Nol10 |
| V | Nol8 |
| V | Npat |
| V | Nqo2 |
| V | Nr2c2 |
| V | Nr2f2 |
| V | Nr3c1 |
| V | Nrbp1 |
| V | Nrg1 |
| V | Nsbp1 |
| V | Nsmaf |
| V | Nudt1 |
| V | Nudt4 |
| V | Nufip2 |
| V | Nup153 |
| V | Nup50 |
| V | Nup98 |
| V | Nupl1 |
| V | Nupl1 |
| V | Olfm1 |
| V | Olfr741 |
| V | Osbpl1a |
| V | Osbpl8 |
| V | OTTMUSG00000015282 |
| V | Otud3 |
| V | Otud7b |
| V | Otud7b |
| V | Oxsr1 |
| V | Pa2g4 |
| V | Pabpc2 |
| V | Paip2b |
| V | Panx1 |
| V | Papd5 |
| V | Pard6b |
| V | Parp14 |
| V | Pax6 |
| V | Pbx2 |
| V | Pcdhga7 |
| V | Pcf11 |
| V | Pctk2 |
| V | Pcyt1a |
| V | Pdcd11 |
| V | Pdcl |
| V | Pde2a |
| V | Pde3b |
| V | Pde4a |
| V | Pde4d |
| V | Pde4d |
| V | Pdlim5 |
| V | Pdpk1 |
| V | Pdxk |
| V | Pecam1 |
| V | Peli1 |
| V | Perq1 |
| V | Pex11a |
| V | Pfkfb2 |
| V | Pfkfb3 |
| V | Pfkfb3 |
| V | Pftk1 |
| V | Phf20 |
| V | Phf3 |
| V | Phf3 |
| V | Phf6 |
| V | Phip |
| V | Phip |
| V | Phka2 |
| V | Phka2 |
| V | Phldb2 |
| V | Phr1 |
| V | Picalm |
| V | Piga |
| V | Pign |
| V | Pik3c2a |
| V | Pik3cb |
| V | Pik3cd |
| V | Pik3r1 |
| V | Pip5k2b |
| V | Pitpnm2 |
| V | Pitpnm2 |
| V | Pitpnm2 |
| V | Pkp3 |
| V | Pla2g12a |
| V | Pla2g2f |
| V | Plcb1 |
| V | Plcb1 |
| V | Plec1 |
| V | Plekha1 |
| V | Plekha7 |
| V | Plscr2 |
| V | Plscr2 |
| V | Plxnb2 |
| V | Plxnc1 |
| V | Pmm2 |
| V | Pnpt1 |
| V | Podxl |
| V | Polr3g |
| V | Pom121 |
| V | Pom121 |
| V | Pou2f1 |
| V | Ppm1d |
| V | Ppm1k |
| V | Ppme1 |
| V | Ppp1r12a |
| V | Ppp1r12b |
| V | Ppp2cb |
| V | Prcc |
| V | Prdm4 |
| V | Prickle2 |
| V | Prkab2 |
| V | Prkacb |
| V | Prkrir |
| V | Prpf38b |
| V | Prpf40a |
| V | Prpf4b |
| V | Prr8 |
| V | Prrc1 |
| V | Prrx1 |
| V | Prss22 |
| V | Pscd3 |
| V | Pscdbp |
| V | Psme4 |
| V | Ptcd3 |
| V | Ptk2b |
| V | Ptms |
| V | Ptpn11 |
| V | Ptpn21 |
| V | Ptpn7 |
| V | Ptprc |
| V | Ptprz1 |
| V | Ptrf |
| V | Pum2 |
| V | Pus3 |
| V | R3hdm1 |
| V | Rab11fip5 |
| V | Rab12 |
| V | Rab22a |
| V | Rab2b |
| V | Rab43 |
| V | Rab8b |
| V | Rad21 |
| V | Rai14 |
| V | Ramp2 |
| V | Ranbp6 |
| V | Rassf4 |
| V | Rbm16 |
| V | Rbm24 |
| V | Rbm25 |
| V | Rbm26 |
| V | Rbm27 |
| V | Rcor3 |
| V | Rffl |
| V | Rffl |
| V | Rfxdc2 |
| V | Rgs4 |
| V | Rin2 |
| V | Rin2 |
| V | Riok1 |
| V | Ripk1 |
| V | Rkhd2 |
| V | Rmnd5a |
| V | Rmrp |
| V | Rnf103 |
| V | Rnf111 |
| V | Rnf12 |
| V | Rnf139 |
| V | Rnf146 |
| V | Rnf183 |
| V | Rnf19 |
| V | Rnf214 |
| V | Rnf214 |
| V | Rnf24 |
| V | Rnf38 |
| V | Rnf4 |
| V | Rnf6 |
| V | Rock1 |
| V | Rock2 |
| V | Rp2h |
| V | Rrbp1 |
| V | Rrp12 |
| V | Rrp1b |
| V | Rsad2 |
| V | Rsl1d1 |
| V | Rsrc1 |
| V | Rtn4 |
| V | Rufy1 |
| V | Runx3 |
| V | Sat1 |
| V | Sbf2 |
| V | Sbno1 |
| V | Sbno1 |
| V | Scamp2 |
| V | Sclt1 |
| V | Sdccag3 |
| V | Sema6b |
| V | Senp2 |
| V | Sesn2 |
| V | Setd8 |
| V | Setd8 |
| V | Setx |
| V | Sf3b3 |
| V | Sfmbt1 |
| V | Sfrs10 |
| V | Sfrs11 |
| V | Sfrs12 |
| V | Sfrs15 |
| V | Sfrs3 |
| V | Sgms1 |
| V | Sh3d19 |
| V | Sh3glb1 |
| V | Sh3kbp1 |
| V | Sh3rf1 |
| V | Shprh |
| V | Shroom4 |
| V | Sirpa |
| V | Sirpa |
| V | Skil |
| V | Sla |
| V | Slamf9 |
| V | Slc10a7 |
| V | Slc16a3 |
| V | Slc27a6 |
| V | Slc35a3 |
| V | Slc35a5 |
| V | Slc38a2 |
| V | Slc45a3 |
| V | Slc4a7 |
| V | Slc4a8 |
| V | Slc6a6 |
| V | Slmap |
| V | Smarca1 |
| V | Smarca5 |
| V | Smarcc2 |
| V | Smc2 |
| V | Smg7 |
| V | Smg7 |
| V | Snap23 |
| V | Snap23 |
| V | Snapc3 |
| V | Snrp70 |
| V | Soat1 |
| V | Socs7 |
| V | Sp100 |
| V | Sp100 |
| V | Sp4 |
| V | Sp4 |
| V | Spata6 |
| V | Spats2 |
| V | Spna2 |
| V | Spnb2 |
| V | Spop |
| V | SpU1 |
| V | SpU1 |
| V | Spty2d1 |
| V | Srbd1 |
| V | Srgap1 |
| V | Ssr3 |
| V | Stat1 |
| V | Stat2 |
| V | Stat3 |
| V | Stat6 |
| V | Stau2 |
| V | Stk3 |
| V | Strn3 |
| V | Stx2 |
| V | Suhw4 |
| V | Suv420h1 |
| V | Suz12 |
| V | Svil |
| V | Synj1 |
| V | Syt11 |
| V | Tacc2 |
| V | Taf1 |
| V | Taf1 |
| V | Taf4b |
| V | Taf7 |
| V | Taf7 |
| V | Tal1 |
| V | Tbc1d23 |
| V | Tbc1d9 |
| V | Tbc1d9b |
| V | Tbcel |
| V | Tbcel |
| V | Tbl1xr1 |
| V | TC1602578 |
| V | TC1614082 |
| V | TC1619269 |
| V | TC1620718 |
| V | TC1659888 |
| V | TC1676807 |
| V | TC1677080 |
| V | TC1688109 |
| V | TC1707826 |
| V | TC1709674 |
| V | TC1717672 |
| V | TC1735696 |
| V | Tead1 |
| V | Tep1 |
| V | Terf2 |
| V | Terf2ip |
| V | Tgfb2 |
| V | Tgfbr1 |
| V | Tgoln2 |
| V | Tgs1 |
| V | Thap6 |
| V | Thbs1 |
| V | Thbs1 |
| V | Thex1 |
| V | Thrap1 |
| V | Tjp1 |
| V | Tle1 |
| V | Tmbim4 |
| V | Tmem161b |
| V | Tmem80 |
| V | Tnks |
| V | Tnks1bp1 |
| V | Tnks2 |
| V | Tnrc15 |
| V | Tnrc6a |
| V | Tnrc6b |
| V | Tob1 |
| V | Tob2 |
| V | Tob2 |
| V | Tpd52 |
| V | Tpr |
| V | Trabd |
| V | Traf4 |
| V | Trak2 |
| V | Tram2 |
| V | Trim14 |
| V | Trrap |
| V | Trrap |
| V | Tspyl4 |
| V | Ttbk2 |
| V | Ttc9c |
| V | Ttl |
| V | Ttll7 |
| V | Ttyh2 |
| V | Txndc5 |
| V | Ubap2l |
| V | Ube1x |
| V | Ube2e2 |
| V | Ube2h |
| V | Ubxd8 |
| V | Unc5b |
| V | Unc84b |
| V | Upf3b |
| V | Usp12 |
| V | Usp14 |
| V | Usp15 |
| V | Usp33 |
| V | Usp36 |
| V | Usp38 |
| V | Usp42 |
| V | Usp47 |
| V | Usp47 |
| V | Utp14a |
| V | Utrn |
| V | Utrn |
| V | Utrn |
| V | Vamp2 |
| V | Vamp2 |
| V | Vcl |
| V | Vcpip1 |
| V | Vps13c |
| V | Vps35 |
| V | Vps54 |
| V | Wac |
| V | Wapal |
| V | Wdfy2 |
| V | Wdr33 |
| V | Wdr37 |
| V | Wdr43 |
| V | Wdr60 |
| V | Wsb1 |
| V | Wwp2 |
| V | Wwp2 |
| V | Xpo4 |
| V | Xpr1 |
| V | Zbtb11 |
| V | Zbtb7a |
| V | Zc3h13 |
| V | Zc3h13 |
| V | Zc3h6 |
| V | Zc3h6 |
| V | Zc3h7a |
| V | Zc3hav1 |
| V | Zcchc11 |
| V | Zcchc2 |
| V | Zcchc6 |
| V | Zcchc8 |
| V | Zeb2 |
| V | Zfml |
| V | Zfp106 |
| V | Zfp146 |
| V | Zfp148 |
| V | Zfp236 |
| V | Zfp292 |
| V | Zfp318 |
| V | Zfp397 |
| V | Zfp398 |
| V | Zfp40 |
| V | Zfp426 |
| V | Zfp444 |
| V | Zfp451 |
| V | Zfp462 |
| V | Zfp462 |
| V | Zfp597 |
| V | Zfp609 |
| V | Zfp629 |
| V | Zfp644 |
| V | Zfp668 |
| V | Zfp688 |
| V | Zfp697 |
| V | Zfp748 |
| V | Zfp75 |
| V | Zfp91 |
| V | Zfr |
| V | Zfr |
| V | Zfy1 |
| V | Zfy2 |
| V | Zfyve26 |
| V | Zhx1 |
| V | Zhx1 |
| V | Zmynd17 |
| V | Znhit1 |
| V | Zranb2 |
| V | Zswim4 |
| V | Zubr1 |
| W | 3-Mar |
| W | 0610038F07Rik |
| W | 1110007C09Rik |
| W | 1110049F12Rik |
| W | 1110054O05Rik |
| W | 1500003O03Rik |
| W | 1700020O03Rik |
| W | 1700029F09Rik |
| W | 1700029F09Rik |
| W | 1700037H04Rik |
| W | 1700084J12Rik |
| W | 2010106G01Rik |
| W | 2010209O12Rik |
| W | 2010305A19Rik |
| W | 2310004I24Rik |
| W | 2310008M10Rik |
| W | 2310008M10Rik |
| W | 2310047M10Rik |
| W | 2310047O13Rik |
| W | 2410001C21Rik |
| W | 2410018C17Rik |
| W | 2410019A14Rik |
| W | 2410129H14Rik |
| W | 2610018G03Rik |
| W | 2610019A05Rik |
| W | 2610024G14Rik |
| W | 2610036D13Rik |
| W | 2610039C10Rik |
| W | 2610203C20Rik |
| W | 2610206B13Rik |
| W | 2810452K22Rik |
| W | 2900009I07Rik |
| W | 2900024C23Rik |
| W | 3110001K24Rik |
| W | 3110002H16Rik |
| W | 3110082I17Rik |
| W | 3300001M20Rik |
| W | 4921537I17Rik |
| W | 4930486L24Rik |
| W | 4930486L24Rik |
| W | 4933425L03Rik |
| W | 5730437N04Rik |
| W | 5730494M16Rik |
| W | 5730557B15Rik |
| W | 5830433M19Rik |
| W | 5830472M02Rik |
| W | 6030446N20Rik |
| W | 6530404N21Rik |
| W | 9630042H07Rik |
| W | A_51_P305350 |
| W | A230046K03Rik |
| W | A530064D06Rik |
| W | A830080D01Rik |
| W | AA408296 |
| W | AA536717 |
| W | Abcb1b |
| W | Abcb7 |
| W | Abcd1 |
| W | Abcd1 |
| W | Abcg2 |
| W | Abcg2 |
| W | Abhd10 |
| W | Abhd12 |
| W | Abr |
| W | Acn9 |
| W | Acp1 |
| W | Adal |
| W | Adora2b |
| W | Adsl |
| W | Agpat5 |
| W | AI314976 |
| W | AI413782 |
| W | AI467606 |
| W | AI480556 |
| W | AI504432 |
| W | AI848100 |
| W | Aifm1 |
| W | AK039920 |
| W | AK042960 |
| W | AK052777 |
| W | AK076354 |
| W | AK077405 |
| W | AK089957 |
| W | AK162362 |
| W | Akt3 |
| W | Aldh9a1 |
| W | Alg13 |
| W | Alg2 |
| W | Alg9 |
| W | Anapc4 |
| W | Anp32e |
| W | Ap3s1 |
| W | Ap4e1 |
| W | Apaf1 |
| W | Apbb1ip |
| W | Arcn1 |
| W | Arg2 |
| W | Arid4a |
| W | Arl4c |
| W | Arl6 |
| W | Arl6 |
| W | Armet |
| W | Arsi |
| W | Ash2l |
| W | Atf2 |
| W | Atf4 |
| W | Atg3 |
| W | Atpbd1c |
| W | Aven |
| W | Azin1 |
| W | B3gnt2 |
| W | B3gnt2 |
| W | B4galt2 |
| W | B4galt2 |
| W | Bat3 |
| W | Bat5 |
| W | Bax |
| W | Bbs12 |
| W | Bbs7 |
| W | BC016423 |
| W | BC020077 |
| W | BC025546 |
| W | BC027231 |
| W | BC035295 |
| W | BC043301 |
| W | BC048507 |
| W | BC049807 |
| W | BC067068 |
| W | Bet1 |
| W | Bgn |
| W | Bhlhb5 |
| W | Blcap |
| W | Bmper |
| W | Bmper |
| W | Brms1 |
| W | Bst1 |
| W | Btaf1 |
| W | Btbd4 |
| W | Btbd9 |
| W | Bxdc2 |
| W | C87436 |
| W | Cad |
| W | Cad |
| W | Cad |
| W | Calca |
| W | Calm2 |
| W | Camk1 |
| W | Camk1 |
| W | Capns1 |
| W | Cbwd1 |
| W | Ccdc109b |
| W | Ccdc49 |
| W | Ccdc94 |
| W | Ccdc98 |
| W | Ccnc |
| W | Cct3 |
| W | Cd302 |
| W | Cd8a |
| W | Cdc34 |
| W | Cdc42ep5 |
| W | Cdc42ep5 |
| W | Cdc45l |
| W | Cdc91l1 |
| W | Cdk4 |
| W | Cdk7 |
| W | Cdr2 |
| W | Cenpc1 |
| W | Cenpq |
| W | Cept1 |
| W | Cfl1 |
| W | Chac2 |
| W | Chd4 |
| W | Chd7 |
| W | Chmp6 |
| W | Chn1 |
| W | CJ236955 |
| W | Cklf |
| W | Cklf |
| W | Clcc1 |
| W | Cnih4 |
| W | Cnr2 |
| W | Cog2 |
| W | Cog8 |
| W | Col2a1 |
| W | Commd1 |
| W | Cops3 |
| W | Coq10b |
| W | Coro1b |
| W | Coro1c |
| W | Cpeb2 |
| W | Cpeb4 |
| W | Creb3l1 |
| W | Crebl1 |
| W | Crlf3 |
| W | Crsp8 |
| W | Cry1 |
| W | Csk |
| W | Cspp1 |
| W | Ctdspl2 |
| W | Ctsc |
| W | Ctsl |
| W | Cutc |
| W | Cwf19l1 |
| W | Cxcl13 |
| W | Cxcr7 |
| W | Cyb5b |
| W | D14Ertd581e |
| W | D1Ertd622e |
| W | D230004J03Rik |
| W | D230037D09Rik |
| W | D830012I24Rik |
| W | D930043N17Rik |
| W | D9Ertd402e |
| W | Dapk3 |
| W | Dars |
| W | Dbndd2 |
| W | Dbnl |
| W | Dclre1a |
| W | Dclre1a |
| W | Ddah1 |
| W | Ddx19a |
| W | Depdc7 |
| W | Derl1 |
| W | Dgat1 |
| W | Dhrs4 |
| W | Dhx15 |
| W | Dhx15 |
| W | Dhx40 |
| W | Dkc1 |
| W | Dlg5 |
| W | Dnajb9 |
| W | Dock11 |
| W | Donson |
| W | Drr1 |
| W | Dtnbp1 |
| W | Dtwd1 |
| W | E2f5 |
| W | Ecd |
| W | Edg5 |
| W | Edg5 |
| W | Edg6 |
| W | Eea1 |
| W | Eed |
| W | EG238395 |
| W | EG634588 |
| W | Egfr |
| W | Eid2 |
| W | Eif2s1 |
| W | Eif3s2 |
| W | Eif4enif1 |
| W | Ell2 |
| W | Emp3 |
| W | Enoph1 |
| W | Enoph1 |
| W | ENSMUST00000056717 |
| W | ENSMUST00000063037 |
| W | ENSMUST00000074789 |
| W | ENSMUST00000075235 |
| W | ENSMUST00000085365 |
| W | ENSMUST00000085410 |
| W | ENSMUST00000092045 |
| W | Eprs |
| W | Ergic2 |
| W | Ergic3 |
| W | Ethe1 |
| W | Evi2b |
| W | Evi2b |
| W | Exdl2 |
| W | Exosc1 |
| W | Exosc1 |
| W | Farsa |
| W | Farsb |
| W | Fastkd1 |
| W | Fbn1 |
| W | Fbn1 |
| W | Fbxl5 |
| W | Fbxo45 |
| W | Fbxw2 |
| W | Fdps |
| W | Fgfr1 |
| W | Fkbp1a |
| W | Flnb |
| W | Fosl2 |
| W | Foxk2 |
| W | Foxp4 |
| W | Fubp1 |
| W | Fusip1 |
| W | Fzd1 |
| W | Garnl4 |
| W | Gatad1 |
| W | Gatad1 |
| W | Gfpt2 |
| W | Ghitm |
| W | Gle1l |
| W | Glmn |
| W | Gmfg |
| W | Gmps |
| W | Gnas |
| W | Gnb1 |
| W | Gnb4 |
| W | Gnl3 |
| W | Gnl3l |
| W | Gorasp2 |
| W | Gpaa1 |
| W | Gpr19 |
| W | Gpr4 |
| W | Gprk5 |
| W | Gprk6 |
| W | Gtf2a2 |
| W | Gtpbp8 |
| W | H1f0 |
| W | H2-D1 |
| W | H2-K1 |
| W | H2-T22 |
| W | Hace1 |
| W | Has2 |
| W | Has2 |
| W | Hat1 |
| W | Hax1 |
| W | Hes1 |
| W | Heyl |
| W | Hic1 |
| W | Hipk2 |
| W | Hisppd1 |
| W | Hnrpc |
| W | Hoxa1 |
| W | Hoxa9 |
| W | Hoxb3 |
| W | Iars |
| W | Icam1 |
| W | Id3 |
| W | Ifitm1 |
| W | Ifitm2 |
| W | Ifitm2 |
| W | Ifitm7 |
| W | Il2rg |
| W | Ilf3 |
| W | Ing3 |
| W | Ints4 |
| W | Itfg2 |
| W | Itgb2 |
| W | Itgb4bp |
| W | Iws1 |
| W | Jazf1 |
| W | Jmjd6 |
| W | Josd1 |
| W | Kcnip3 |
| W | Kcnmb1 |
| W | Kctd12 |
| W | Kdelr3 |
| W | Kif18a |
| W | Kif21a |
| W | Klf3 |
| W | Kpna4 |
| W | Ksr1 |
| W | Lactb |
| W | Lancl1 |
| W | Laptm4a |
| W | Laptm4b |
| W | Lepre1 |
| W | Lhfpl2 |
| W | Lilrb4 |
| W | Lmbrd1 |
| W | Lmo2 |
| W | Lmo4 |
| W | LOC194985 |
| W | LOC627563 |
| W | LOC670106 |
| W | LOC674229 |
| W | Lonrf1 |
| W | Loxl1 |
| W | Lrrc3 |
| W | Lrrc40 |
| W | Lrrc59 |
| W | Lsm2 |
| W | Lsm2 |
| W | Lypla1 |
| W | Maged2 |
| W | Map2k1 |
| W | Map3k3 |
| W | Mare |
| W | Mars |
| W | Mars2 |
| W | Matr3 |
| W | Mbd2 |
| W | Mbd2 |
| W | Mbtps2 |
| W | Mcm8 |
| W | Mcm9 |
| W | Me2 |
| W | Med18 |
| W | Med8 |
| W | Mesdc2 |
| W | Mfn1 |
| W | Mfsd8 |
| W | Mical2 |
| W | Mkrn1 |
| W | Mllt11 |
| W | Mlx |
| W | Moap1 |
| W | Mobkl2c |
| W | Mphosph9 |
| W | Mrpl15 |
| W | Mrpl3 |
| W | Mrpl37 |
| W | Mrpl40 |
| W | Mrpl40 |
| W | Mrpl44 |
| W | Mrpl9 |
| W | Mrps18b |
| W | Msn |
| W | Mst1r |
| W | Mtm1 |
| W | Mtx2 |
| W | Mum1l1 |
| W | Mutyh |
| W | Mvk |
| W | Myo5a |
| W | Myohd1 |
| W | NAP000001-074 |
| W | NAP025822-1 |
| W | NAP026555-1 |
| W | NAP029862-1 |
| W | NAP043943-1 |
| W | NAP057019-1 |
| W | NAP057020-1 |
| W | NAP093832-001 |
| W | NAP103789-1 |
| W | NAP104272-1 |
| W | NAP123115-1 |
| W | Napg |
| W | Nat12 |
| W | Ndfip2 |
| W | Ndufa12 |
| W | Ndufa12 |
| W | Ndufv2 |
| W | Nfkbiz |
| W | Nfu1 |
| W | Nkrf |
| W | Nol14 |
| W | Nola1 |
| W | Npm1 |
| W | Nsdhl |
| W | Nsdhl |
| W | Nsg1 |
| W | Nsmce2 |
| W | Nt5c2 |
| W | Nt5c3l |
| W | Ntan1 |
| W | Nucb2 |
| W | Nudt5 |
| W | Nudt6 |
| W | Nup153 |
| W | Nup205 |
| W | Nup37 |
| W | Nup43 |
| W | Nupl2 |
| W | Obfc1 |
| W | Obfc2a |
| W | Olfml3 |
| W | ORF61 |
| W | Otud6b |
| W | Pacs1 |
| W | Parp2 |
| W | Pcdhb22 |
| W | Pdcd6ip |
| W | Pde8a |
| W | Pemt |
| W | Pex11c |
| W | Pex3 |
| W | Pfpl |
| W | Pgd |
| W | Pgm1 |
| W | Phf23 |
| W | Phgdhl1 |
| W | Phlda1 |
| W | Pias1 |
| W | Pigg |
| W | Pigl |
| W | Pigx |
| W | Pim2 |
| W | Pla2g12a |
| W | Pla2g4a |
| W | Plaa |
| W | Pldn |
| W | Plekhb2 |
| W | Plekhf2 |
| W | Plk2 |
| W | Plod1 |
| W | Plrg1 |
| W | Pnpla7 |
| W | Pnpt1 |
| W | Pola2 |
| W | Pold2 |
| W | Pole4 |
| W | Polh |
| W | Polh |
| W | Polr3d |
| W | Polr3k |
| W | Ppap2a |
| W | Ppef2 |
| W | Ppp2r1b |
| W | Ppp2r5c |
| W | Ppwd1 |
| W | Pqlc3 |
| W | Praf2 |
| W | Prdm2 |
| W | Prkag1 |
| W | Prmt1 |
| W | Prmt3 |
| W | Prpf31 |
| W | Prpf4b |
| W | Prpf8 |
| W | Prr16 |
| W | Psma3 |
| W | Psmb2 |
| W | Psmd14 |
| W | Psme4 |
| W | Psmf1 |
| W | Ptcd1 |
| W | Ptgs2 |
| W | Ptplb |
| W | Pus3 |
| W | Pycr1 |
| W | Qtrtd1 |
| W | Rab11a |
| W | Rab34 |
| W | Rab5a |
| W | Rabepk |
| W | Rabggtb |
| W | Rabggtb |
| W | Rabif |
| W | Rap1b |
| W | Rars2 |
| W | Raver1 |
| W | Rbm22 |
| W | Rbm22 |
| W | Rcl1 |
| W | Rcl1 |
| W | Reep3 |
| W | Rg9mtd1 |
| W | Rgs19 |
| W | Rgs3 |
| W | Ripk1 |
| W | Rnh1 |
| W | Rogdi |
| W | Rp2h |
| W | Rpa3 |
| W | Rpl29 |
| W | Rpl29 |
| W | Rpl29 |
| W | Rpusd3 |
| W | Rtn4 |
| W | Runx2 |
| W | Ryk |
| W | S100a16 |
| W | Saal1 |
| W | Scamp4 |
| W | Scotin |
| W | Sec11c |
| W | Sec13 |
| W | Sec13 |
| W | Sec24a |
| W | Sec61a2 |
| W | Seh1l |
| W | Seh1l |
| W | Selplg |
| W | Sfn |
| W | Sh2b3 |
| W | Sh2b3 |
| W | Shq1 |
| W | Siah1a |
| W | Slc16a10 |
| W | Slc20a2 |
| W | Slc24a5 |
| W | Slc25a39 |
| W | Slc25a43 |
| W | Slc25a43 |
| W | Slc35a5 |
| W | Slc38a2 |
| W | Slc5a6 |
| W | Slc6a6 |
| W | Smad3 |
| W | Smarca5 |
| W | Smndc1 |
| W | Smpdl3b |
| W | Smu1 |
| W | Snai1 |
| W | Snapc1 |
| W | Snrpa |
| W | Snrpa1 |
| W | Snx12 |
| W | Snx27 |
| W | Socs4 |
| W | Socs4 |
| W | Sod2 |
| W | Spata6 |
| W | Spire1 |
| W | Srd5a2l |
| W | Srfbp1 |
| W | Srp9 |
| W | Srprb |
| W | St3gal3 |
| W | Stab1 |
| W | Steap4 |
| W | Stoml2 |
| W | Strn4 |
| W | Stxbp2 |
| W | Surf5 |
| W | Taf5 |
| W | Taf5 |
| W | Tardbp |
| W | Tbc1d20 |
| W | Tbc1d7 |
| W | Tbk1 |
| W | TC1615264 |
| W | TC1638264 |
| W | TC1648248 |
| W | TC1660970 |
| W | Tcea2 |
| W | Tcerg1 |
| W | Tec |
| W | Tex261 |
| W | Tex261 |
| W | Tfdp1 |
| W | Tgfbr1 |
| W | Timm9 |
| W | Tjap1 |
| W | Tln1 |
| W | Tm2d3 |
| W | Tmem142a |
| W | Tmem177 |
| W | Tmem186 |
| W | Tmem33 |
| W | Tmem39b |
| W | Tmem5 |
| W | Tmem55b |
| W | Tmem62 |
| W | Tmem70 |
| W | Tmem77 |
| W | Tnfrsf21 |
| W | Tollip |
| W | Tpm3 |
| W | Tprkb |
| W | Tra2a |
| W | Trappc2 |
| W | Trappc3 |
| W | Trim27 |
| W | Trmt5 |
| W | Trnt1 |
| W | Trpc2 |
| W | Trpc4ap |
| W | Tspo |
| W | Tspo |
| W | Tsr1 |
| W | Ttc1 |
| W | Ttc32 |
| W | Ttc7 |
| W | Ttc9c |
| W | Tubb2c |
| W | Tusc3 |
| W | Twistnb |
| W | Txndc14 |
| W | Tyk2 |
| W | U2af1 |
| W | U2af2 |
| W | Ube2a |
| W | Ube2d1 |
| W | Ube2d3 |
| W | Ube2d3 |
| W | Ube2f |
| W | Ube2g2 |
| W | Ube2j2 |
| W | Ube2m |
| W | Ubl7 |
| W | Ubox5 |
| W | Ubtd2 |
| W | Uchl4 |
| W | Uchl4 |
| W | Ufd1l |
| W | Unc119 |
| W | Unk |
| W | Usp6nl |
| W | Utp6 |
| W | Vac14 |
| W | Vav3 |
| W | Vbp1 |
| W | Vps26b |
| W | Vrk2 |
| W | Wdr18 |
| W | Wdr70 |
| W | Wdr77 |
| W | Wdr77 |
| W | Wdr8 |
| W | Wnk1 |
| W | Wtap |
| W | Xbp1 |
| W | Xrcc4 |
| W | Yars2 |
| W | Yipf1 |
| W | Yipf1 |
| W | Yipf3 |
| W | Yme1l1 |
| W | Ywhae |
| W | Zcchc11 |
| W | Zdhhc13 |
| W | Zdhhc7 |
| W | Zeb2 |
| W | Zfp37 |
| W | Zfp420 |
| W | Zfp53 |
| W | Zfp53 |
| W | Zfp54 |
| W | Zfp609 |
| W | Zfp655 |
| W | Zfp68 |
| W | Zfp75 |
| W | Zfp817 |
| W | Zfp9 |
| W | Zfr |
| W | Zik1 |
| W | Zkscan5 |
| W | Zswim3 |
