# Supplementary material for: Modeling Host Genetic Regulation of Influenza Pathogenesis in the Collaborative Cross
Source: PLoS Pathog. 2013 Feb 28;9(2):e1003196. doi: 10.1371/journal.ppat.1003196 (PMC3585141; doi:10.1371/journal.ppat.1003196)
Supplement: Table S12 — GO-term enrichment by module in Mx1 -/- subpopulation. (DOCX) [file ppat.1003196.s018.docx]

| **Table S12. GO-term enrichment by module in *Mx1*-/- subpopulation** | | |
| --- | --- | --- |
| **Module** | **GO Term** | **BY FDR** |
| M | ncRNA metabolic process | 0.000124 |
|  | ribosome biogenesis | 0.002461 |
|  | ncRNA processing | 0.003119 |
|  | ribonucleoprotein complex biogenesis | 0.01382 |
|  | tRNA metabolic process | 0.037418 |
|  | T cell differentiation during immune response | 0.038899 |
|  | alpha-beta T cell differentiation during immune response | 0.038899 |
|  | CD4-positive, alpha-beta T cell differentiation during immune response | 0.038899 |
|  | T-helper cell differentiation | 0.038899 |
| N | immune system process | 1.10E-09 |
|  | immune response | 3.88E-08 |
|  | defense response | 5.74E-08 |
|  | cell activation | 7.81E-08 |
|  | leukocyte activation | 1.35E-06 |
|  | lymphocyte activation | 1.07E-05 |
|  | regulation of response to stimulus | 1.46E-05 |
|  | response to stress | 0.000309 |
|  | response to stimulus | 0.000402 |
|  | regulation of immune system process | 0.000909 |
|  | response to wounding | 0.001117 |
|  | response to other organism | 0.001525 |
|  | response to external stimulus | 0.002028 |
|  | regulation of immune response | 0.00213 |
|  | mononuclear cell proliferation | 0.002346 |
|  | lymphocyte proliferation | 0.002346 |
|  | regulation of response to stress | 0.002572 |
|  | positive regulation of response to stimulus | 0.002677 |
|  | inflammatory response | 0.002994 |
|  | leukocyte proliferation | 0.003177 |
|  | T cell activation | 0.003282 |
|  | response to biotic stimulus | 0.007784 |
|  | regulation of cell activation | 0.014293 |
|  | immune response-regulating signaling pathway | 0.015032 |
|  | defense response to bacterium | 0.01524 |
|  | positive regulation of immune system process | 0.015784 |
|  | locomotory behavior | 0.015784 |
|  | immune effector process | 0.016988 |
|  | response to bacterium | 0.020697 |
|  | innate immune response | 0.023119 |
|  | regulation of leukocyte activation | 0.024575 |
|  | B cell activation | 0.024796 |
|  | regulation of immune effector process | 0.026209 |
|  | immune system development | 0.030397 |
|  | leukocyte differentiation | 0.034404 |
|  | T cell proliferation | 0.038899 |
|  | response to chemical stimulus | 0.039748 |
|  | multi-organism process | 0.041169 |
|  | chemotaxis | 0.041362 |
|  | taxis | 0.041362 |
|  | regulation of lymphocyte activation | 0.043375 |
|  | immune response-activating signal transduction | 0.043861 |
|  | hemopoiesis | 0.046151 |
|  | regulation of defense response | 0.047834 |
| O | enzyme linked receptor protein signaling pathway | 0.002816 |
|  | cell communication | 0.017829 |
|  | fibroblast growth factor receptor signaling pathway | 0.04037 |
| Q | M phase | 6.88E-08 |
|  | cell cycle | 7.70E-06 |
|  | cell cycle phase | 1.46E-05 |
|  | M phase of mitotic cell cycle | 1.67E-05 |
|  | nuclear division | 1.67E-05 |
|  | mitosis | 1.67E-05 |
|  | cell cycle process | 2.24E-05 |
|  | organelle fission | 3.28E-05 |
|  | cell division | 8.39E-05 |
|  | mitotic cell cycle | 0.003226 |
|  | cellular component organization | 0.007266 |
|  | organelle organization | 0.008008 |
|  | DNA conformation change | 0.014968 |
|  | DNA metabolic process | 0.031005 |
|  | chromosome segregation | 0.032336 |
|  | chromosome organization | 0.03327 |
|  | DNA packaging | 0.042511 |
| T | generation of precursor metabolites and energy | 3.91E-11 |
|  | small molecule catabolic process | 1.19E-09 |
|  | oxidation reduction | 6.88E-08 |
|  | cellular carbohydrate catabolic process | 1.46E-05 |
|  | small molecule metabolic process | 1.65E-05 |
|  | carbohydrate catabolic process | 7.20E-05 |
|  | glucose metabolic process | 7.83E-05 |
|  | alcohol catabolic process | 0.000124 |
|  | carboxylic acid metabolic process | 0.000144 |
|  | oxoacid metabolic process | 0.000144 |
|  | organic acid metabolic process | 0.000149 |
|  | hexose metabolic process | 0.000167 |
|  | monosaccharide metabolic process | 0.000212 |
|  | cellular ketone metabolic process | 0.000229 |
|  | fatty acid metabolic process | 0.000262 |
|  | monocarboxylic acid metabolic process | 0.000402 |
|  | energy derivation by oxidation of organic compounds | 0.000534 |
|  | glycolysis | 0.000682 |
|  | glucose catabolic process | 0.001057 |
|  | hexose catabolic process | 0.001057 |
|  | monosaccharide catabolic process | 0.001057 |
|  | electron transport chain | 0.001361 |
|  | cellular respiration | 0.001559 |
|  | cellular carbohydrate metabolic process | 0.001876 |
|  | cellular lipid metabolic process | 0.001876 |
|  | catabolic process | 0.003085 |
|  | cardiac muscle tissue development | 0.005488 |
|  | tricarboxylic acid cycle | 0.006067 |
|  | acetyl-CoA catabolic process | 0.006067 |
|  | alcohol metabolic process | 0.006246 |
|  | muscle contraction | 0.016026 |
|  | acetyl-CoA metabolic process | 0.016988 |
|  | aerobic respiration | 0.016988 |
|  | coenzyme catabolic process | 0.016988 |
|  | organic acid catabolic process | 0.016988 |
|  | carboxylic acid catabolic process | 0.016988 |
|  | muscle system process | 0.019156 |
|  | carbohydrate metabolic process | 0.025783 |
|  | cofactor catabolic process | 0.025783 |
|  | lipid metabolic process | 0.027109 |
|  | cellular component assembly involved in morphogenesis | 0.037418 |
|  | myofibril assembly | 0.038385 |
| U | sensory perception of smell | 2.43E-10 |
|  | sensory perception of chemical stimulus | 2.64E-10 |
|  | G-protein coupled receptor protein signaling pathway | 6.88E-08 |
|  | sensory perception | 4.66E-07 |
|  | cognition | 1.67E-05 |
|  | neurological system process | 0.000149 |
|  | system process | 0.002653 |
|  | cell surface receptor linked signaling pathway | 0.03129 |
| V | cellular macromolecule metabolic process | 7.70E-06 |
|  | gene expression | 3.54E-05 |
|  | regulation of gene expression | 5.43E-05 |
|  | macromolecule metabolic process | 7.83E-05 |
|  | regulation of macromolecule biosynthetic process | 0.00011 |
|  | regulation of cellular biosynthetic process | 0.000137 |
|  | regulation of nitrogen compound metabolic process | 0.000147 |
|  | regulation of nucleobase, nucleoside, nucleotide and nucleic acid metabolic process | 0.000149 |
|  | regulation of biosynthetic process | 0.00015 |
|  | regulation of transcription | 0.000167 |
|  | regulation of primary metabolic process | 0.000402 |
|  | regulation of macromolecule metabolic process | 0.000403 |
|  | cellular macromolecule biosynthetic process | 0.000415 |
|  | transcription | 0.000453 |
|  | macromolecule biosynthetic process | 0.001361 |
|  | regulation of cellular metabolic process | 0.001876 |
|  | nucleobase, nucleoside, nucleotide and nucleic acid metabolic process | 0.00213 |
|  | primary metabolic process | 0.00256 |
|  | regulation of metabolic process | 0.002806 |
|  | chromatin modification | 0.003043 |
|  | cellular metabolic process | 0.008008 |
|  | chromatin organization | 0.012208 |
|  | chromosome organization | 0.016026 |
|  | RNA metabolic process | 0.016988 |
|  | regulation of cellular process | 0.019156 |
|  | regulation of biological process | 0.020697 |
|  | nitrogen compound metabolic process | 0.021411 |
|  | cellular biosynthetic process | 0.037418 |
| W | RNA processing | 0.000402 |
|  | ncRNA metabolic process | 0.002613 |
|  | RNA splicing | 0.003286 |
|  | tRNA metabolic process | 0.00682 |
|  | cellular macromolecule metabolic process | 0.016605 |
|  | cellular metabolic process | 0.033379 |
|  | nitrogen compound metabolic process | 0.047834 |

Both the Gene Ontology term (GO Term) and the Benjamini and Yekutieli false discovery rate (BY FDR) are shown for modules that had significant representation in GO-terms. 3 modules (P, R, and S) were not significantly enriched for any GO terms.
