# Supplementary material for: Modeling Host Genetic Regulation of Influenza Pathogenesis in the Collaborative Cross
Source: PLoS Pathog. 2013 Feb 28;9(2):e1003196. doi: 10.1371/journal.ppat.1003196 (PMC3585141; doi:10.1371/journal.ppat.1003196)
Supplement: Table S13 — Transcriptional module correlates with influenza phenotypes in the Mx1 -/- subpopulation. (DOCX) [file ppat.1003196.s019.docx]

| **Table S13. Transcriptional module correlates with influenza phenotypes in the *Mx1*-/- subpopulation** | | | | | | | | | | | | |
| --- | --- | --- | --- | --- | --- | --- | --- | --- | --- | --- | --- | --- |
|  | | Mod W | Mod M | Mod N | Mod U | Mod V | Mod T | Mod R | Mod P | Mod O | Mod S | Mod Q |
| Clinical Disease | D4 weight | -0.13 | -0.24 | -0.12 | -0.04 | -0.07 | 0.18 | 0.27 | 0.21 | 0.22 | 0.21 | -0.05 |
|  | D4 clinical | 0.14 | 0.24 | 0.23 | 0.02 | 0.01 | -0.09 | -0.28 | -0.2 | -0.19 | -0.21 | -0.18 |
|  | Hemorrhage | 0.06 | 0.06 | 0.14 | 0.05 | -0.08 | -0.04 | -0.05 | -0.09 | -0.07 | -0.11 | 0.0 |
|  | Gross Edema | 0.12 | 0.14 | 0.07 | -0.01 | -0.01 | -0.04 | -0.12 | -0.07 | -0.06 | -0.06 | -0.05 |
| Viral Replication | Log titer | 0.24 | 0.11 | 0.14 | -0.07 | -0.15 | -0.04 | -0.27 | -0.09 | -0.07 | -0.14 | -0.1 |
|  | IHC score | 0.23 | 0.38 | 0.31 | -0.02 | 0.03 | -0.16 | -0.33 | -0.31 | -0.33 | -0.37 | -0.03 |
| Inflammatory Cell Infiltration | Airway inflam | 0.14 | 0.23 | 0.18 | -0.04 | 0.1 | -0.05 | -0.37 | -0.18 | -0.24 | -0.28 | 0.02 |
|  | Airway mono | 0.2 | 0.08 | 0.03 | -0.17 | -0.1 | 0.02 | -0.12 | 0.03 | -0.04 | -0.13 | 0.12 |
|  | Airway neut | 0.11 | 0.09 | -0.01 | -0.02 | -0.02 | -0.1 | -0.14 | 0.04 | -0.08 | -0.13 | 0.04 |
|  | Vascular inflam | 0.03 | 0.1 | 0.08 | 0.02 | 0.11 | -0.07 | -0.14 | -0.14 | -0.15 | -0.15 | 0.09 |
|  | Vasc mono | 0.21 | 0.05 | 0.03 | -0.15 | -0.14 | 0.02 | -0.1 | 0.04 | -0.01 | -0.12 | 0.11 |
|  | Vasc neut | 0.06 | 0.06 | -0.09 | 0 | 0.04 | 0.01 | -0.11 | 0.03 | -0.04 | -0.08 | -0.02 |
|  | Alveolar inflam | 0.16 | 0.07 | 0.11 | -0.01 | -0.05 | 0.03 | -0.04 | -0.07 | -0.11 | -0.18 | 0.19 |
| Pathology | Airway damage | 0 | 0.15 | 0.02 | 0.01 | 0.12 | -0.04 | -0.21 | -0.12 | -0.18 | -0.2 | 0.02 |
|  | Alveolar damage | 0.02 | 0.08 | 0.08 | 0.01 | 0.04 | 0.09 | 0.07 | -0.12 | -0.16 | -0.15 | 0.15 |
|  | Pulmonary Edema | -0.16 | -0.08 | -0.17 | -0.11 | 0.1 | -0.14 | 0.2 | 0.17 | 0.11 | 0.11 | 0.03 |
|  | Fibrin | 0.1 | 0.16 | 0.03 | 0 | -0.03 | -0.05 | -0.16 | -0.04 | -0.07 | -0.15 | -0.02 |
| Correlations are Spearman’s Rho. Blue shading indicate significance, lt. blue p<0.05, med blue p<0.001, dk blue p<0.00001 Abbreviations: inflam=inflammatory cell infiltrates, neut = neutrophils, mono = monocytes | | | | | | | | | | | | |
