# Supplementary material for: Modeling Host Genetic Regulation of Influenza Pathogenesis in the Collaborative Cross
Source: PLoS Pathog. 2013 Feb 28;9(2):e1003196. doi: 10.1371/journal.ppat.1003196 (PMC3585141; doi:10.1371/journal.ppat.1003196)
Supplement: Table S14 — Primers used in sequencing Mx1 . (DOCX) [file ppat.1003196.s020.docx]

| **Table S14. Primers used in sequencing *Mx1*** | |  |
| --- | --- | --- |
| **Primer ID** | **Primer Sequence** |  |
| Mx1-Exon-1-Forward | TTCCCAACCTCAGTACCAAGCCAA | |
| Mx1-Exon-1-Reverse | CAAAGCAGGCTCCTGTCTGCAC |  |
| Mx1-Exon-2-Forward | GTTTGCAGACAGAACCCAGTGCTT | |
| Mx1-Exon-2-Reverse | CCAAGCAAAGTAAGCAGCTGACCA | |
| Mx1-Exon-3-Forward | TGGAGCATGACCTCACTCTTGCTT | |
| Mx1-Exon-3-Reverse | GCTATGTCTCCAAACTGGGAAGG | |
| Mx1-Exon-4,5-Forward | GTGAAGGTGTGGCCCCATATTGG | |
| Mx1-Exon-4,5-Reverse | CTGTCTCCATCAAACATGTGGTAAAGCC | |
| Mx1-Exon-6-Forward | GGCTTTACCACATGTTTGATGGAGACAG | |
| Mx1-Exon-6-Reverse | TGCCCTCATTTGGATCCTGAGCTT | |
| Mx1-Exon-7-Forward | GTGCTGCTTTCTGGTTTCCTGCTT |  |
| Mx1-Exon-7-Reverse | CCTACTGGAATTAATGCGCTGGTC | |
| Mx1-Exon-8,9,10-Forward | ACTACCCAGCACAAACCCACTTCT | |
| Mx1-Exon-8,9,10-Reverse | AGGATGCTCTGGGAGAACCACAAT | |
| Mx1-Exon-10,11,12,13-Forward | TGATGCAGAGCCCTTAAGTCCCAA | |
| Mx1-Exon-10,11,12,13-Reverse | TCCTGTGGAGTGTTCAGAGTAAGG | |
| Mx1-Exon-14-Forward | TCCCAGGTGATACCTGTGTTTGGT | |
| Mx1-Exon-14-Reverse | TCCAAACCTGCCTGAGCCATAGAA | |
| Mx1-Exon-15A-Forward | TTGGAAATCTGGACCCTTCTGGGA | |
| Mx1-Exon-15A-Reverse | TTTCTGCCTGTGGAAAGGACTGGA | |
| Mx1-Exon-15B-Forward | GTGGAAGCAAGCAAGCCCTTTGTA | |
| Mx1-Exon-15B-Reverse | TTGTCCTGGGAACTTGGACAGGAA | |
| Mx1-InsertTest-Forward | TTCTTCTGGAAGATGGGAAGGCCA | |
| Mx1-InsertTest-Reverse | TGGCCTTCCCATCTTCCAGAAGAA | |
| Mx1-cDNA-Ex1-Forward | TTGATTCTCCCTGGGAAGGAGTGA | |
| Mx1-cDNA-Ex7-Forward | AAGGCAAGGTCTTGGATGTGATGC | |
| Mx1-cDNA-Ex7-Reverse | GCATCACATCCAAGACCTTGCCTT | |
| Mx1-cDNA-Ex15-Reverse | ACTACTTGTGAGGTTCCTGCAGCA | |
| MX1-InsertSeq-1 | TGAAGCTTGCTCCCAAGTTCTG |  |
| Mx1-Insertion2-Forward | GCTCTTCTTCCCCTGTGTTCAAAGTC | |
| Mx1-Insertion2-Reverse | GGTCAGAACATACTGAATTCCTGAACACC | |
| Mx1-Insert1.5-Forward | GAGTTGTCAAACCTCCTTGAGGACTGG | |
| Mx1-Insertion1.5-Reverse | GACTCTGTCTTCTGACAGCAAATCCC | |
| MX1-InsertSeq-4-Forward | TCACTGCCACTATTGGAAGATCA |  |
| MX1-InsertSeq-4-Reverse | TCAAATAAATAGCAGTCATCAGAGTGC | |
| MX1-InsertSeq-5-Forward | ACCAGAAGATGACAGAACGAGG | |
| MX1-InsertSeq-5-Reverse | GAACGAGGATGTCCTTTCTGGTG | |
| MX1-InsertSeq-6-Forward | TCAGTGCCTTCAATAGGAATATCATGA | |
| MX1-InsertSeq-7-Forward | CTTGCTTGGGATGATCATATTGAGG | |
| MX1-InsertSeq-8-Forward | GATTCTCCTGAGGTACAAAGCAAG | |
| MX1-InsertSeq-8-Reverse | GAAAATCAGTATCGTGGCCGGG |  |
| Mx1-Ex8-Insertion-Reverse | TTGAGATGCAAACTCCACCAAGCC | |
| MX1-InsertSeq-7F-Comp-R | CCTCAATATGATCATCCCAAGCAAG | |
